# Origin of the α‐Effect in S_N_2 Reactions

**DOI:** 10.1002/anie.202106053

**Published:** 2021-07-26

**Authors:** Thomas Hansen, Pascal Vermeeren, F. Matthias Bickelhaupt, Trevor A. Hamlin

**Affiliations:** ^1^ Department of Theoretical Chemistry Amsterdam Institute of Molecular and Life Sciences (AIMMS) Amsterdam Center for Multiscale Modeling (ACMM) Vrije Universiteit Amsterdam De Boelelaan 1083 1081 HV Amsterdam The Netherlands; ^2^ Leiden Institute of Chemistry Leiden University Einsteinweg 55 2333 CC Leiden The Netherlands; ^3^ Institute for Molecules and Materials Radboud University Heyendaalseweg 135 6525 AJ Nijmegen The Netherlands

**Keywords:** activation strain model, basicity, density functional calculations, nucleophilicity, α-effect

## Abstract

The α‐effect is a term used to explain the dramatically enhanced reactivity of α‐nucleophiles (R−Y−X:^−^) compared to their parent normal nucleophile (R−X:^−^) by deviating from the classical Brønsted‐type reactivity‐basicity relationship. The exact origin of this effect is, however, still heavily under debate. In this work, we have quantum chemically analyzed the α‐effect of a set of anionic nucleophiles, including *O*‐, *N*‐ and *S*‐based normal and α‐nucleophiles, participating in an S_N_2 reaction with ethyl chloride using relativistic density functional theory at ZORA‐OLYP/QZ4P. Our activation strain and Kohn–Sham molecular orbital analyses identified two criteria an α‐nucleophile needs to fulfill in order to show α‐effect: (i) a small HOMO lobe on the nucleophilic center, pointing towards the substrate, to reduce the repulsive occupied–occupied orbital overlap and hence (steric) Pauli repulsion with the substrate; and (ii) a sufficiently high energy HOMO to overcome the loss of favorable HOMO–LUMO orbital overlap with the substrate, as a consequence of the first criterion, by reducing the HOMO–LUMO orbital energy gap. If one of these two criteria is not fulfilled, one can expect no α‐effect or inverse α‐effect.

## Graphical abstract

The α‐effect explained! Quantum chemical activation strain analyses reveal the intrinsic factors that α‐nucleophiles need to exhibit α‐effect. The origin is traced back to a small HOMO lobe to reduce the destabilizing Pauli repulsion, and a sufficiently high energy HOMO to overcome the loss of favorable orbital overlap.

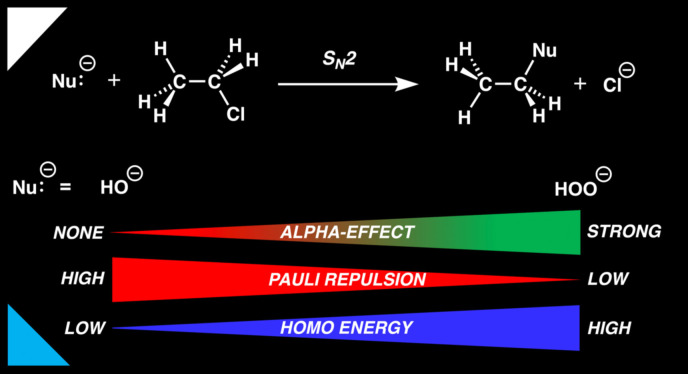

## Introduction

**Figure 1:**
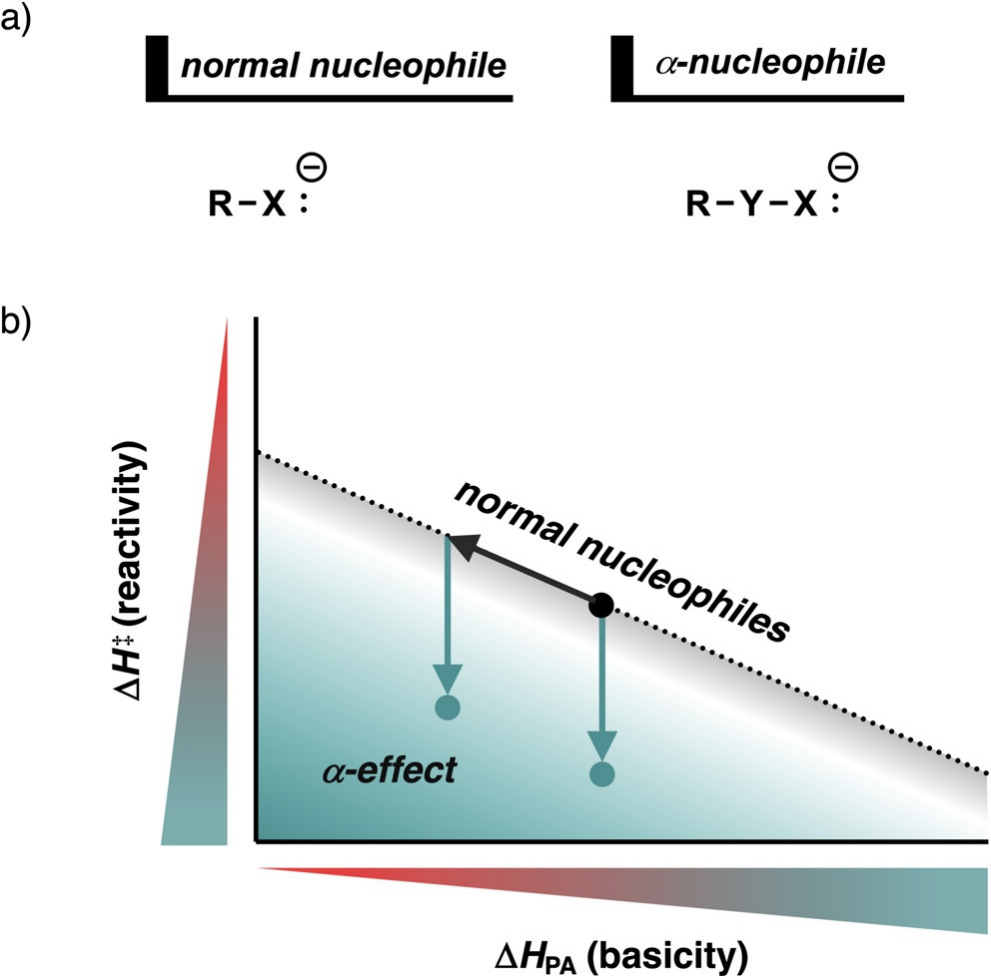
a) Normal and α‐nucleophiles; b) overview of the Brønsted‐type correlation between the reactivity and basicity (black dotted line), in which α‐nucleophiles with α‐effect have a downward deviation from this trend (teal arrow), showing greater reactivity than would be expected based on their basicity (i.e., α‐effect).

The α‐effect is a fundamental phenomenon in organic chemistry that refers to the dramatically enhanced reactivity of a nucleophile featuring a lone pair‐bearing heteroatom adjacent to the nucleophilic center (i.e., the α‐position).^[1]^ In 1962, Pearson and Edwards introduced the term α‐effect^[1b]^ to denote a downward deviation from the Brønsted‐type correlation (reaction barrier versus proton affinity, see Figure 1) found for normal nucleophiles.^[2]^ The α‐effect has been found in a myriad of reactions in which the magnitude of this effect is highly dependent on both the studied class of reaction and type of α‐nucleophiles.^[3]^

Despite extensive experimental and computational studies, the origin of the α‐effect remains elusive and under debate.^[4]^ Based on the intrinsic properties of the α‐nucleophile several theories on the origin of the α‐effect have been proposed, such as ground state destabilization, transition state stabilization, or thermodynamic product stability.[^3f^, ^3g^, ^3h^, ^5^] Next to that, the α‐effect is also ascribed to external factors, like solvent‐induced effects.^[2a]^ The ground state destabilization theory proposes that the electron‐electron repulsion between the lone pair electrons of the nucleophilic center and the α‐atom result in a higher HOMO energy of the α‐nucleophile, making it more reactive. This explains the higher reactivity of the α‐nucleophile; however, this electronic mechanism should also increase the basicity and, therefore, cannot fully explain the α‐effect. Several explanations, which fall in the class of the transition state stabilization, have been proposed, e.g., secondary orbital interactions, electron transfer contribution, tighter transition state, and polarizability of α‐nucleophiles. The secondary orbital interactions theory argues that the second lone‐pair‐bearing heteroatom of α‐nucleophiles can engage in an additional orbital interaction with the substrate and thereby stabilizing the TS.[^4m^, ^5b^] The electron transfer contribution model suggests that α‐nucleophiles exhibit some single electron transfer character in the S_N_2 transition state, which can be stabilized by the adjacent lone‐pair bearing atom.[^5c^, ^5d^, ^5e^] Many also ascribe the α‐effect to a tighter transition state of the α‐nucleophiles.^[3j]^ Lastly, the higher polarizability of α‐nucleophiles also has been proposed as the driving force behind the α‐effect.^[5f]^ Although these theories share the requirements, rendering the system to be more reactive than one would expect based on their basicity, only a limited amount of quantitative data is available on these explanations, which ultimately hampers pinpointing the exact underlying mechanism which is responsible for the α‐effect.

**Scheme 1:**
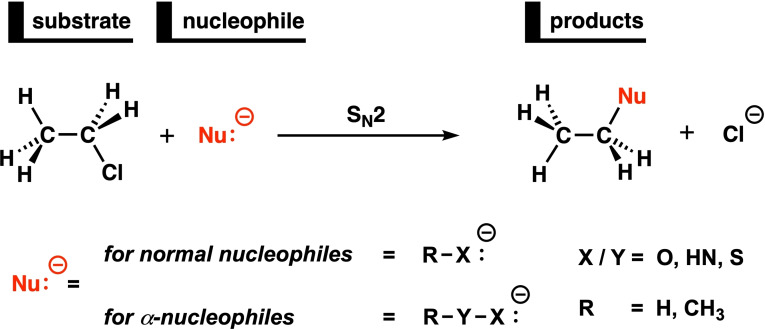
Schematic overview of the computationally analyzed S_N_2 reactions of normal and α‐nucleophiles with C_2_H_5_Cl.

With the aim of providing a unified framework with which to understand the α‐effect in terms of the intrinsic properties of the α‐nucleophile, we disentangled the physical mechanisms and identified causal structure–reactivity relationships controlling S_N_2 reactions involving normal and α‐nucleophiles. We have explored and analyzed the potential energy surface of Nu:^−^ + C_2_H_5_Cl, with Nu:^−^ being a set of anionic nucleophiles including *O*‐, *N*‐ and *S*‐based normal and α‐nucleophiles, by using relativistic density functional theory (DFT) at ZORA‐OLYP/QZ4P (Scheme 1). The nucleophile (Nu:^−^) is, for the parent normal nucleophiles, defined by R−X:^−^ while for α‐nucleophiles by R−Y−X:^−^, in which X, Y=O, HN, S and R=H, CH_3_. The activation strain model (ASM)^[6]^ of reactivity in combination with Kohn–Sham molecular orbital (KS‐MO) theory^[7a]^ and the matching energy decomposition analysis (EDA)[^7b^, ^7c^] were employed to provide quantitative insight into the factors that are responsible for the α‐effect. This methodological approach enables the analysis of the potential energy surface and, more importantly, the reaction barrier, by decomposing the total energy of the system into physically meaningful and chemically intuitive terms, and has shown to be valuable for understanding the reactivity of, amongst others, nucleophilic substitution reactions.^[8]^

## Results and Discussion

### Main Trends in Reactivity

**Figure 2:**
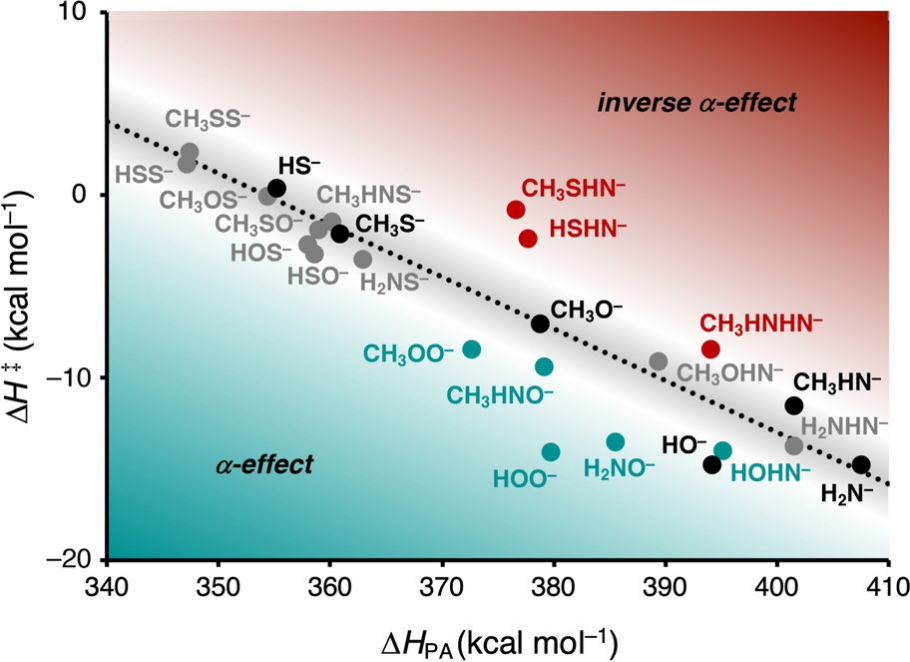
Brønsted‐type correlation between the reaction barrier (i.e., Δ*H* ^≠^; kinetics) and the basicity (i.e., Δ*H*_PA_; thermodynamics) for the S_N_2 reaction of Nu:^−^ + C_2_H_5_Cl. The normal nucleophiles (i.e., R−Y:^−^) are indicated in black. The α‐nucleophiles (i.e., R−X−Y:^−^) have three distinct classes: class I, exhibiting α‐effect, teal dots; class II, having a minor degree or no α‐effect, grey dots; class III, showing inverse α‐effect, red dots, where X, Y=O, HN, S and R=H, CH_3_. The linear trend line (black dotted line; R^2^=0.93) is fitted to the normal nucleophile data set. Computed at ZORA‐OLYP/QZ4P.

The first step was to quantify which of the α‐nucleophiles exhibit α‐effect in the S_N_2 reaction with C_2_H_5_Cl. Figure 2 shows the Brønsted‐type correlation diagram of the analyzed S_N_2 reactions, in which the reaction barrier (i.e., Δ*H* ^≠^) is plotted as a function of the basicity (i.e., Δ*H*_PA_). See SI Tables S1 and S2 for all complete reaction profiles. In line with previous studies,[^4j^, ^4k^, ^4l^] a good correlation emerges between the reaction barrier and the basicity of the six normal nucleophiles (R−X:^−^=HO^−^, CH_3_O^−^, H_2_N^−^, CH_3_HN^−^, CH_3_S^−^, HS^−^), which are indicated by black dots (Figure 2). For the α‐nucleophiles (R−Y−X:^−^), the introduction of the electron‐withdrawing heteroatom Y (i.e., O, HN, S) adjacent to the nucleophilic center (i.e., X:^−^) leads in most cases to a less basic and reactive nucleophile. In order words, most α‐nucleophiles shift left‐upwards, following the Brønsted‐type correlation diagram of the normal nucleophiles (Figure 1, black arrow on the dotted correlation line). However, some of these α‐nucleophiles also deviate in a downward trend from the Brønsted‐type correlation, making them more reactive than one would expect based on their basicity (Figure 1, teal arrow below the dotted correlation line). These α‐nucleophiles, therefore, exhibit α‐effect.

By analyzing the computed reaction barriers and basicity's, three distinct classes of α‐nucleophiles (i.e., R−Y−X:^−^) can be discerned from the Brønsted‐type correlation diagram (Figure 2): (i) α‐nucleophiles with an apparent downward deviation from the Brønsted‐type correlation and hence exhibiting α‐effect, such as HOO^−^, CH_3_OO^−^, H_2_NO^−^, CH_3_HNO^−^, HOHN^−^ (teal dots); (ii) α‐nucleophiles, mainly *S*‐, *N*‐based, but also *O*‐based with an adjacent sulfur atom, showing a minor degree or no α‐effect and thus behaving like their parent normal analog (grey dots); (iii) α‐nucleophiles featuring a degree of inverse α‐effect, making them less reactive than would be expected based on their basicity and show an upward deviation from the Brønsted‐type correlation, such as HSHN^−^ and CH_3_SHN^−^ (red dots).^[9]^ In addition, we have computed the Brønsted‐type correlation diagrams of the S_N_2 reactions between the herein studied normal and α‐nucleophiles with substrates (i.e., electrophiles) varying in size and leaving group: R−Y, where R=Me, Et, *i*‐Pr; Y=F, Cl (SI Figures S1–S3 and S9, and Tables S3 and S11). We are able to derive the same conclusions as for ethyl chloride, which shows that the origin of the α‐effect in S_N_2 reactions is independent of the studied substrate (see below). Notably, we found, like Ren and Yamataka,^4l^ that the α‐effect becomes more apparent as the size of the substrate increases.

### Origin of Reactivity

**Figure 3:**
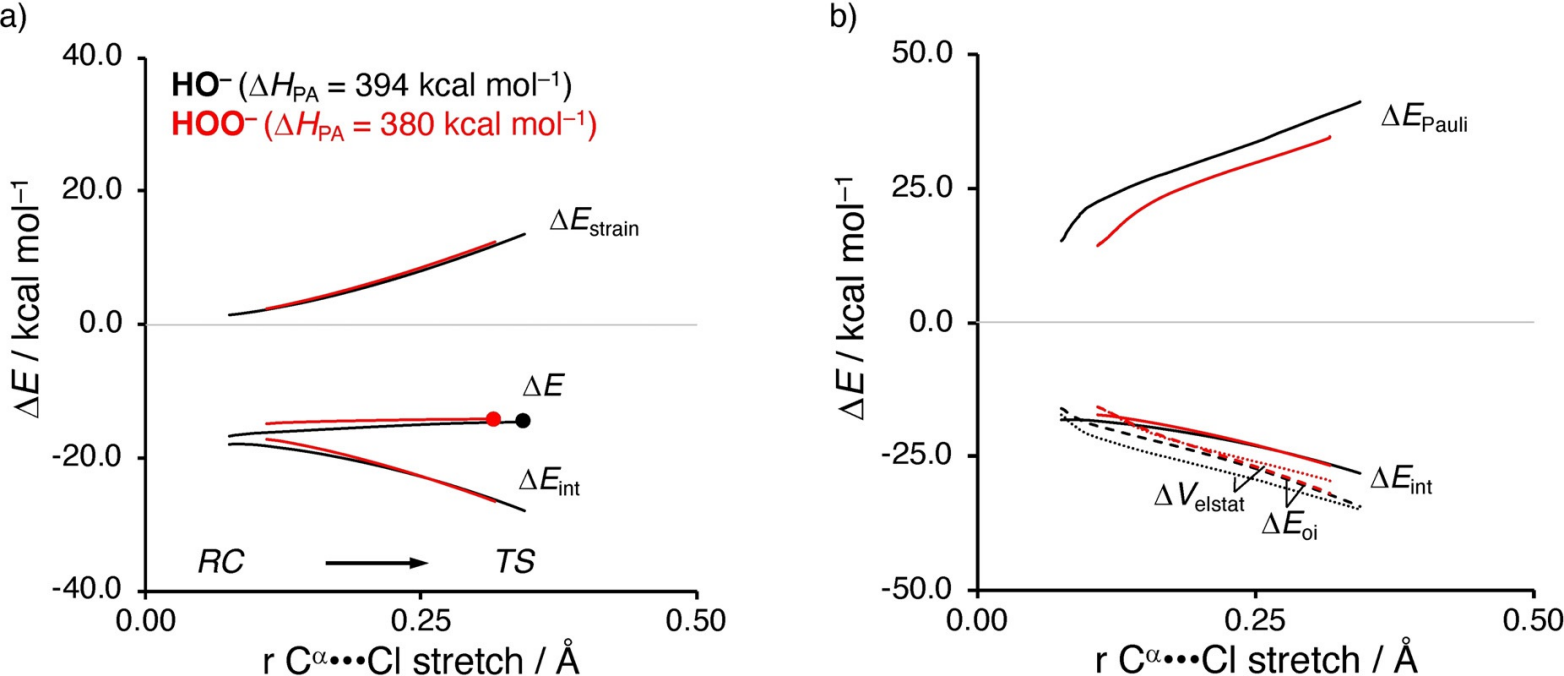
a) Activation strain analysis; and b) energy decomposition analysis of the S_N_2 reactions between HO^−^ (black; normal nucleophile) and HOO^−^ (red; α‐nucleophile) + C_2_H_5_Cl, along the IRC projected on the C^α^⋅⋅⋅Cl bond stretch. Computed at ZORA‐OLYP/QZ4P.

In order to gain quantitative insight into the physical factors behind the α‐effect, we turn to the activation strain model (ASM) of reactivity.^[6]^ The ASM decomposes the electronic energy (Δ*E*) into two distinct energy terms, namely, the strain energy (Δ*E*_strain_) and the interaction energy (Δ*E*_int_). The strain energy results from the deformation of the individual reactants and the interaction energy consists of all mutual interactions between the deformed reactants along the reaction coordinate, defined in this case as the IRC projection onto the C^α^⋅⋅⋅Cl distance.[^8^, ^10^] Figure 3 a shows the activation strain diagrams (ASDs) of HO^−^ and HOO^−^ + C_2_H_5_Cl, which are the most representative and well‐known models for a normal and α‐nucleophile that exhibits strong α‐effect. We found that all other α‐nucleophiles that show α‐effect are governed by the same underlying physical mechanism (see below). The ASDs of all other S_N_2 reactions involving *O*‐nucleophiles are provided in the Supporting Information (see SI Figures S7 and S8). As already shown in Figure 2, HO^−^ and HOO^−^ have similar reactivity (i.e., reaction barriers), but have vastly differing basicity (Δ*H*_PA_=394 and 380 kcal mol^−1^ for HO^−^ and HOO^−^, respectively), which was also experimentally found by Bierbaum and co‐workers.[^4a^, ^4b^] Thus, HOO^−^ exhibits a strong α‐effect by having a significantly lower basicity while being equally reactive compared to the corresponding parent normal nucleophile HO^−^ and hence having a downward deviation from the Brønsted‐type correlation. The origin of the similarity in reaction barriers, in terms of electronic energy (trends in Δ*E* ^≠^ and Δ*H* ^≠^ are identical), can be traced back, by using the ASM, to a nearly identical Δ*E*_strain_ and Δ*E*_int_ for both nucleophiles. Thus, despite the significantly lower basicity of the α‐nucleophile HOO^−^, the interaction with the substrate (i.e., Δ*E*_int_) is maintained equivalent to that of the normal nucleophile HO^−^.^[9]^

To understand why both nucleophiles interact with the substrate with equal interaction energy, we employ the canonical energy decomposition analysis (EDA).[^7b^, ^7c^] Our canonical EDA decomposes the Δ*E*_int_ between the reactants into the following three physically meaningful energy terms: electrostatic interactions (Δ*V*_elstat_), (steric) Pauli repulsion (Δ*E*_Pauli_), and orbital interaction (Δ*E*_oi_). Herein, Δ*V*_elstat_ is the classical electrostatic interaction between the unperturbed charge distributions of the (deformed) reactants. The (steric) Pauli repulsion, Δ*E*_Pauli_, includes the destabilizing interaction between the occupied orbitals of both fragments, due to the Pauli's exclusion principle, and is a measure for steric repulsion. The orbital interaction energy, Δ*E*_oi_, accounts for, amongst others, charge transfer between the fragments, such as HOMO–LUMO interactions. We found that, despite the nearly equivalent Δ*E*_int_ for both nucleophiles, the individual energy terms (i.e., Δ*V*_elstat_ and Δ*E*_Pauli_) are vastly different, in which the (steric) Pauli repulsion is significantly less destabilizing for the α‐nucleophile compared to the parent normal nucleophile (Figure 3 b; see SI Tables S8–S9 and S14 for the ASM/EDA data on consistent geometries, which render identical trends as our initial ASM/EDA data). In contrast, the orbital interactions of the normal nucleophile are almost identical to those of the α‐nucleophile, while the electrostatic interaction is significantly more stabilizing for the normal nucleophile. Thus, it can be concluded that the significant reduction in destabilizing (steric) Pauli repulsion effectively offsets the loss of stabilizing electrostatic interactions and equips the α‐nucleophiles with the α‐effect. In all, the above‐mentioned features compose an α‐nucleophile with a strong α‐effect. Later, we will discuss how this gives rise to a downward deviation from the classical Brønsted‐type reactivity‐basicity relationship.^[9]^

**Figure 4:**
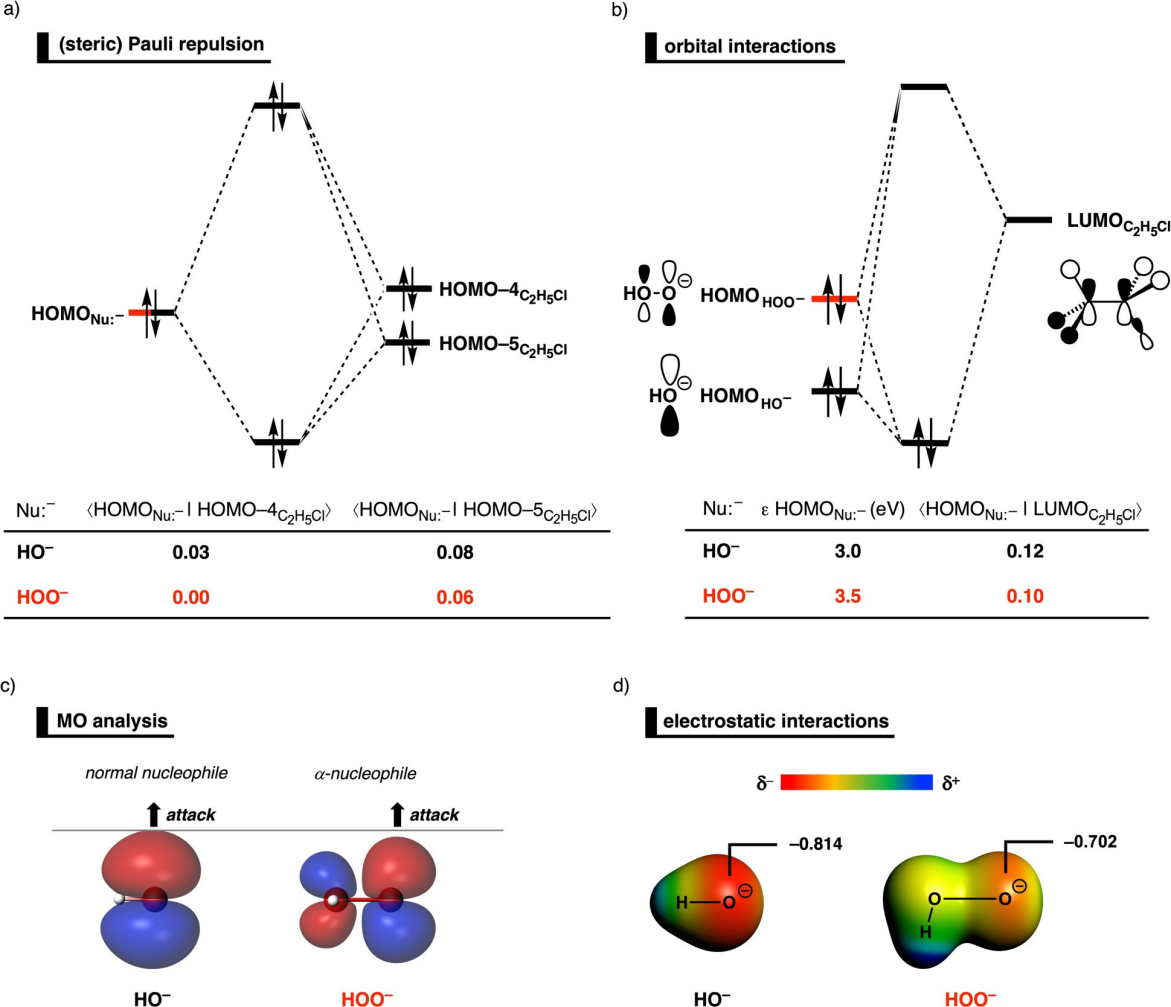
a) Molecular orbital diagram of the most important occupied–occupied orbital overlaps of the S_N_2 reaction between HO^−^ (black; normal nucleophile) and HOO^−^ (red; α‐nucleophile) + C_2_H_5_Cl; b) molecular orbital diagram of the most important donor–acceptor interaction between the HOMO of the nucleophile and the LUMO of C_2_H_5_Cl computed at consistent geometries with a C^α^⋅⋅⋅Cl bond stretch of 0.30 Å; c) representation of the DFT HOMO${}_{\mathrm{Nu}:{}^{-}}$ (isovalue=0.03 Bohr^−3/2^), where the gray horizontal line indicates the maximum spatial extent of the HOMO${}_{\mathrm{HO}{}^{-}}$ ; and d) molecular electrostatic potential maps (at 0.03 Bohr^−3/2^) from −0.4 (red) to 0.0 (blue) Hartree e^−1^ and the Voronoi deformation density^[11]^ of the nucleophilic center at their equilibrium geometries. Computed at ZORA‐OLYP/QZ4P.

In order to find the origin of the less destabilizing (steric) Pauli repulsion for the α‐nucleophile HOO^−^ compared to its parent normal analog HO^−^, we perform a Kohn–Sham molecular orbital analysis.^[7a]^ The occupied orbitals of Nu:^−^ and C_2_H_5_Cl, for both HO^−^ and HOO^−^, were quantified at transition state‐like, consistent geometries with the C^α^⋅⋅⋅Cl bond stretch of 0.30 Å (Figure 4 a). Analysis at this point on the reaction coordinate (near all transition states), rather than the transition state alone, ensured that the results are not skewed by the position of the transition state (i.e., early or late transition state).^[6b]^ Of all possible computed occupied–occupied orbital overlaps, the most important occupied molecular orbitals (MOs) that dictate the trend in (steric) Pauli repulsion, that is, the occupied orbitals responsible for the differences in steric repulsion between the normal and α‐nucleophile and the substrate, are the HOMO${}_{\mathrm{Nu}:{}^{-}}$ of the (α‐)nucleophile and HOMO−4${}_{C{}_{2}H{}_{5}\mathrm{Cl}}$ and HOMO−5${}_{C{}_{2}H{}_{5}\mathrm{Cl}}$ of the substrate C_2_H_5_Cl. The HOMO${}_{\mathrm{Nu}:{}^{-}}$ is the lone‐pair orbital predominantly located on the nucleophilic center, whereas HOMO−4${}_{C{}_{2}H{}_{5}\mathrm{Cl}}$ and HOMO−5${}_{C{}_{2}H{}_{5}\mathrm{Cl}}$ are the filled C−H and C−C σ‐bonding orbitals that are delocalized over the substrate. Note, that the overlap between HOMO${}_{\mathrm{Nu}:{}^{-}}$ and other filled σ‐orbitals on the substrate (e.g., HOMO−3 and HOMO−6) also contribute, although less prominently, to the observed trend in (steric) Pauli repulsion (see SI Table S10). The higher‐lying filled orbitals of the substrate, on the contrary, are the lone‐pair orbitals on the chloride atom (i.e., HOMO and HOMO−1) or form the C−Cl bond σ‐orbital (i.e., HOMO−2), and, therefore, have no occupied–occupied orbital repulsion with the incoming nucleophile. The α‐nucleophile HOO^−^ engages in less occupied–occupied orbital overlap (*S=*0.00 and *S=*0.06), and hence experiences less repulsion, compared to the normal nucleophile HO^−^ (*S=*0.03 and *S=*0.08). The difference in (steric) Pauli repulsion can be rationalized when comparing the spatial extent of the HOMO${}_{\mathrm{Nu}:{}^{-}}$ of the normal and α‐nucleophile (Figure 4 c). The lobe of the filled orbital on the nucleophilic center of the α‐nucleophile HOO^−^ is significantly smaller than the analogous lobe of the normal nucleophile HO^−^, due to the more electronegative oxygen atom adjacent to the nucleophilic center, which, in turn, polarizes orbital density away from the nucleophilic center (see below for a detailed analysis). This ultimately results in less repulsive overlap with the HOMO−4${}_{C{}_{2}H{}_{5}\mathrm{Cl}}$ and HOMO−5${}_{C{}_{2}H{}_{5}\mathrm{Cl}}$ of the substrate and, therefore, a less destabilizing (steric) Pauli repulsion compared to the parent normal nucleophile.

Despite the significantly smaller HOMO${}_{\mathrm{Nu}:{}^{-}}$ lobe of the α‐nucleophile HOO^−^, it can engage in similar orbital interactions (Δ*E*_oi_) with the substrate (i.e., C_2_H_5_Cl) as its parent normal nucleophile HO^−^. This initially counterintuitive observation can be understood by the fact that, while the smaller HOMO${}_{\mathrm{Nu}:{}^{-}}$ lobe leads to less overlap with the LUMO${}_{C{}_{2}H{}_{5}\mathrm{Cl}}$ of the substrate (Figure 4 b), it is effectively compensated by the higher‐lying HOMO of the α‐nucleophile HOO^−^. Now, why is it that HOO^−^ has a higher‐lying HOMO than HO^−^? This can be traced back to the repulsive occupied–occupied orbital interaction between the filled 2*p_z_* atomic orbitals (AOs) of the two neighboring oxygen atoms of HOO^−^ (see below), which, in turn, pushes the HOMO${}_{\mathrm{HOO}{}^{-}}$ up in energy and ultimately results in a smaller HOMO${}_{\mathrm{Nu}:{}^{-}}$ –LUMO${}_{C{}_{2}H{}_{5}\mathrm{Cl}}$ orbital energy gap compared to the S_N_2 reaction involving OH^−^. Furthermore_,_ the electrostatic interaction (Δ*V*_elstat_) is less stabilizing for the α‐nucleophile, which can be directly related to the electron‐withdrawing character of adjacent heteroatom at the α‐position that diminishes the negative charge on the nucleophilic center (Figure 4 d; see SI Table S13 for the VDD atomic charges of all nucleophiles). By performing a numerical experiment where we artificially constrained the HOO^−^⋅⋅⋅C^α^ bond length of the reaction involving HOO^−^ to the bond length of HO^−^⋅⋅⋅C^α^ while keeping the C^α^⋅⋅⋅Cl bond stretch at 0.30 Å (see SI Tables S3 and S4), we can conclude that the obtained interaction energy terms shown in Figure 3 b are not skewed by the difference in Nu:^−^⋅⋅⋅C^α^ distances.

The α‐effect can also manifest in α‐nucleophiles by having a similar basicity but a lower reaction barrier than its normal parent nucleophile, hence having a downward deviation from the Brønsted‐type correlation.^[12]^ For example, CH_3_O^−^ and CH_3_HNO^−^ are equally basic (Δ*H*_PA_=379 kcal mol^−1^), but the reaction barrier of CH_3_HNO^−^ is lower than of CH_3_O^−^ (ΔΔ*H* ^≠^=2.4 kcal mol^−1^; see SI Table S2). This enhanced reactivity of the α‐nucleophile can again be traced back to the less destabilizing (steric) Pauli repulsion (see SI Figures S8c,d). The lobe of the filled orbital on the nucleophilic center of the α‐nucleophile H_3_CHNO^−^ is significantly smaller than the analogous lobe of the normal nucleophile H_3_CO^−^ (see below), which, in turn, undergoes less repulsive overlap with the filled orbitals of the substrate (i.e., C_2_H_5_Cl). This significant reduction of destabilizing (steric) Pauli repulsion effectively overcomes the loss of stabilizing electrostatic interaction, making CH_3_HNO^−^ engage in a stronger interaction with the substrate than CH_3_O^−^. Note that the reaction involving CH_3_HNO^−^ also experiences less destabilizing strain energy, along the entire reaction pathway, compared to the reaction with CH_3_O^−^. This difference in strain energy results from the deformation of the (α‐)nucleophile to accommodate the newly formed covalent bond with the substrate. However, a nearly identical difference in strain energy is also found when comparing the decomposed energy terms of the basicity (see below). This energy term is, therefore, not an important factor for downward deviation from the Brønsted‐type correlation between reactivity and basicity.

To test our proposed general model, we studied an additional set of α‐nucleophiles, including the hypohalite series (i.e., FO^−^, ClO^−^, BrO^−^, and IO^−^). We found, like Ren and Yamataka,^4l^ that FO^−^ and ClO^−^ show a profound α‐effect (see SI Figure S4). The α‐effect for these α‐nucleophiles is induced by the same intrinsic mechanism as discussed above (see SI Figures S14, S19, and Table S12), which reinforces the generality of our findings. Moreover, in line with the experimental work of Bierbaum and co‐workers, we found that BrO^−^ does not exhibit α‐effect.^[4c]^ Taken altogether, the criteria that must be satisfied for an α‐nucleophile to exhibit strong α‐effect are as follows: (i) a HOMO${}_{\mathrm{Nu}:{}^{-}}$ that has a small orbital lobe on the nucleophilic center to reduce (steric) Pauli repulsion with the substrate; and (ii) a high energy HOMO${}_{\mathrm{Nu}:{}^{-}}$ that results in a small HOMO${}_{\mathrm{Nu}:{}^{-}}$ –LUMO_substrate_ orbital energy gap to overcome the diminished favorable HOMO${}_{\mathrm{Nu}:{}^{-}}$ –LUMO_substrate_ orbital overlap. If one of these two criteria is not met, one can expect that the corresponding α‐nucleophile exhibits no α‐effect or even inverse α‐effect. Thus, one only needs to analyze the electronic structure of an α‐nucleophile to determine if it exhibits α‐effect.

**Figure 5:**
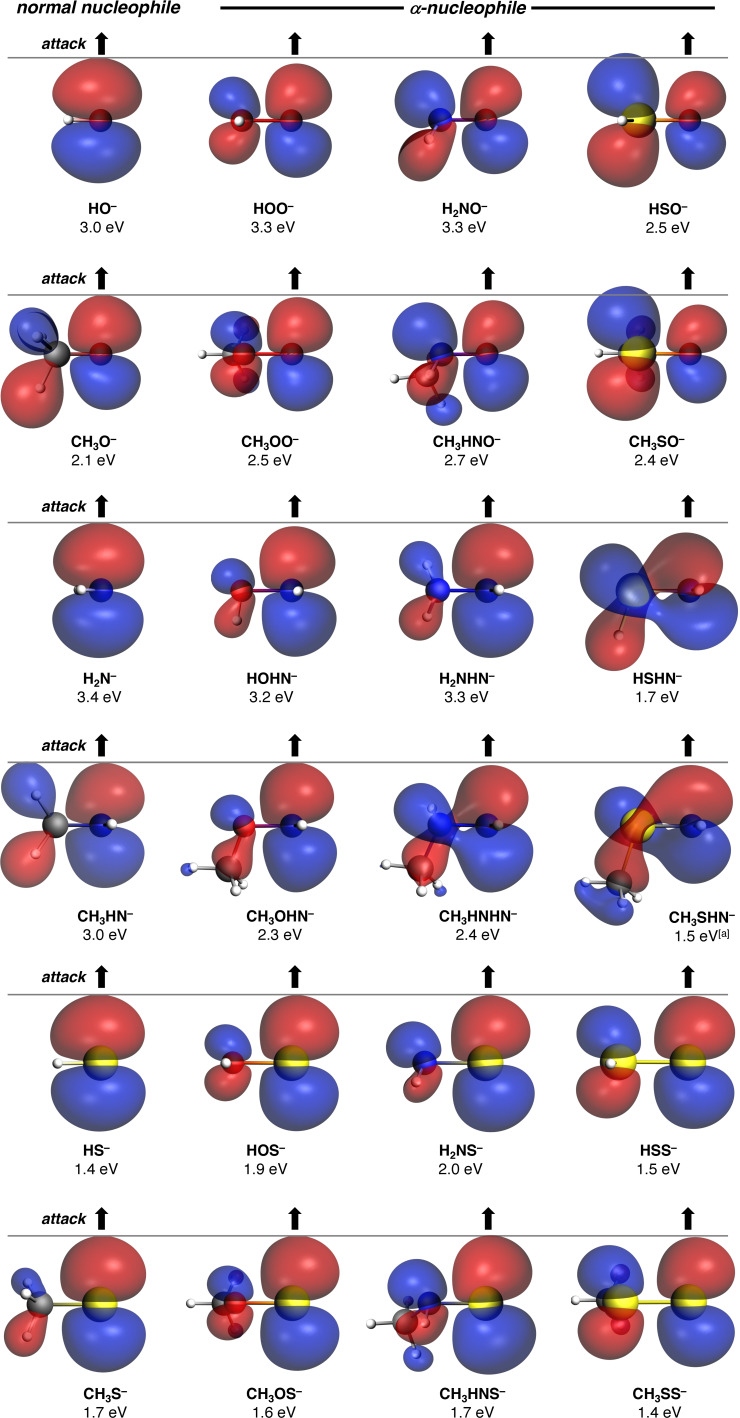
Key occupied orbitals (HOMO${}_{\mathrm{Nu}:{}^{-}}$ ; energies in eV; isovalue=0.030 Bohr^−3/2^ for *O*‐ and *N*‐nucleophiles and isovalue=0.035 Bohr^−3/2^ for *S*‐nucleophiles) computed at equilibrium geometries. Computed at ZORA‐OLYP/QZ4P. [a] HOMO−1 is the key occupied orbital.

To showcase the impact of the HOMO${}_{\mathrm{Nu}:{}^{-}}$ of the α‐nucleophile on its degree of α‐effect, we depict the HOMO${}_{\mathrm{Nu}:{}^{-}}$ of all studied nucleophiles in Figure 5. The α‐nucleophiles that exhibit large degrees of α‐effect (i.e., HOO^−^, H_2_NO^−^, CH_3_OO^−^, CH_3_HNO^−^, HOHN^−^) all share the required characteristics, namely, the HOMO${}_{\mathrm{Nu}:{}^{-}}$ has a smaller lobe on the nucleophilic center and is higher in energy compared to their parent normal nucleophile. Some α‐nucleophiles only meet one of the criteria and, therefore, have less or no α‐effect. For example, HSO^−^ and CH_3_SO^−^ feature a significantly smaller HOMO${}_{\mathrm{Nu}:{}^{-}}$ lobe at the nucleophilic center, however, the HOMO${}_{\mathrm{Nu}:{}^{-}}$ is not sufficiently high in energy to compensate for the considerable loss in favorable HOMO${}_{\mathrm{Nu}:{}^{-}}$ –LUMO${}_{C{}_{2}H{}_{5}\mathrm{Cl}}$ orbital overlap, rendering them significantly less reactive and basic, yielding in almost no α‐effect for both α‐nucleophiles. Many of the studied α‐nucleophiles, such as most *S*‐based α‐nucleophiles, do not even show any of the α‐effect characteristics, having similar‐sized HOMO${}_{\mathrm{Nu}:{}^{-}}$ lobes as their parent normal nucleophile. Moreover, the reason for inverse α‐effect (e.g., HSHN^−^ and CH_3_SHN^−^) can also be directly related to the HOMO${}_{\mathrm{Nu}:{}^{-}}$ of the α‐nucleophiles. This class of α‐nucleophiles have a relatively large HOMO${}_{\mathrm{Nu}:{}^{-}}$ lobe at the nucleophilic center compared to normal nucleophiles with a similar basicity (see SI Figures S15), while also having a lower‐lying HOMO, which results in more destabilizing (steric) Pauli repulsion and a large HOMO–LUMO orbital energy gap compared to their parent normal analog.

**Figure 6:**
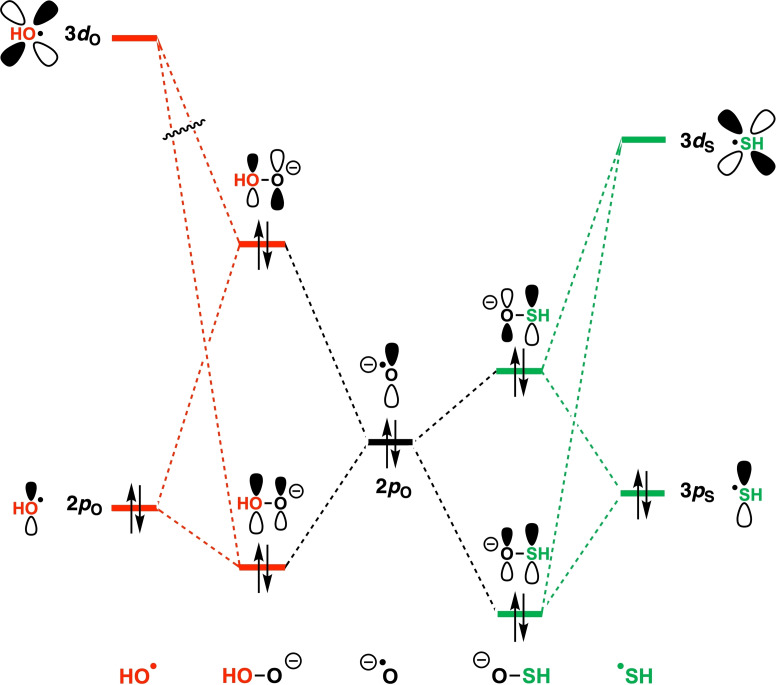
Schematic representation of the construction of the HOMO${}_{\mathrm{HOO}{}^{-}}$ (left, red) and HOMO${}_{\mathrm{HSO}{}^{-}}$ (right, green) from the interaction between the filled 2*p* atomic orbital of the nucleophilic oxygen center (middle, black, 2*p*_O_) and the filled n*p* and empty 3*d* atomic orbitals of the adjacent oxygen and sulfur atom (n*p*_X_ and 3*d*_X_).

At last, we aim to fundamentally understand how different heteroatoms adjacent to the nucleophilic center of an α‐nucleophile yield different degrees of α‐effect. In other words, why does HOO^−^ exhibit α‐effect by fulfilling the above‐mentioned requirements (i.e., small orbital lobe on the nucleophilic center and high energy HOMO), while, for example, HSO^−^ does not possess this significantly enhanced reactivity (Figure 2). As previously mentioned, introducing an electronegative heteroatom adjacent to the nucleophilic oxygen center polarizes the orbital density away from the nucleophilic oxygen center, which, ultimately, makes the filled orbital lobe of α‐nucleophile HOO^−^ on the nucleophilic oxygen center smaller than the analogous lobe on the normal nucleophile HO^−^. Figure 6 shows a schematic representation of the construction of the HOMO${}_{\mathrm{HOO}{}^{-}}$ (left side, red) and HOMO${}_{\mathrm{HSO}{}^{-}}$ (right side, green) from the interaction between the filled 2*p* atomic orbital (AO) of the nucleophilic oxygen center (2*p*_O_) and the filled n*p* and empty 3*d* AOs of the adjacent oxygen and sulfur atom (n*p*_X_ and 3*d*_X_, where X=O, S). Note that a detailed analysis of the construction of the HOMO${}_{\mathrm{HOO}{}^{-}}$ and HOMO${}_{\mathrm{HSO}{}^{-}}$ , including the orbital energies overlaps, and populations can be found in Supplementary Information Figure S16. The interplay between these two orbital interactions determines the stability and shape of the HOMO${}_{\mathrm{HXO}{}^{-}}$ . The HOMO${}_{\mathrm{HXO}{}^{-}}$ is the antibonding combination of the two‐center four‐electron orbital interaction between the 2*p*_O_ of the nucleophilic oxygen center and the n*p*_X_ of the adjacent heteroatom. As a result, the larger the 2*p*_O_‐n*p*_X_ orbital overlap, the more the HOMO${}_{\mathrm{HXO}{}^{-}}$ becomes destabilized (i.e., higher‐lying in energy). The 3*p*_S_ of HS^.^ overlaps to a larger extent with the relatively diffuse anionic oxygen center than the 2*p*_O_ of HO^.^, because they have a better match in diffuseness of atomic orbitals (see SI Figure S17). Thus, one would suggest that the HOMO${}_{\mathrm{HSO}{}^{-}}$ will be destabilized to a larger extent than the HOMO${}_{\mathrm{HOO}{}^{-}}$ . This is, however, not the case, because the HOMO${}_{\mathrm{HSO}{}^{-}}$ becomes stabilized by a favorable donor–acceptor interaction between the filled 2*p*_O_ of the nucleophilic oxygen center and the low‐lying empty 3*d_S_* AO of the adjacent sulfur atom. The formation of HOMO${}_{\mathrm{HOO}{}^{-}}$ , on the other hand, does not benefit from a stabilizing donor–acceptor interaction. This, ultimately, makes the HOMO${}_{\mathrm{HSO}{}^{-}}$ a lower‐lying orbital than the HOMO${}_{\mathrm{HOO}{}^{-}}$ (HOMO${}_{\mathrm{HSO}{}^{-}}$ =2.5 eV; HOMO${}_{\mathrm{HOO}{}^{-}}$ =3.3 eV), which, therefore, engages in a weaker orbital interaction with the substrate along the S_N_2 pathway (see SI Table S8).

The presence, or absence, of the stabilizing orbital interaction not only plays a crucial role in the stability of the HOMO${}_{\mathrm{HXO}{}^{-}}$ , but also determines the shape of this orbital. As seen in Figure 5, the HOMO${}_{\mathrm{HSO}{}^{-}}$ has a smaller orbital lobe on the nucleophilic center than HOMO${}_{\mathrm{HOO}{}^{-}}$ , which makes the former experience both less repulsive occupied–occupied orbital overlap (i.e., Pauli repulsion) and less stabilizing HOMO–LUMO orbital overlap with the substrate along the S_N_2 pathway (see SI Table S8). The strong 2*p*_O_–3*d*_S_ donor–acceptor orbital interaction polarizes the HOMO${}_{\mathrm{HSO}{}^{-}}$ orbital density away from the nucleophilic oxygen center towards the adjacent sulfur atom. This polarizing effect can be quantified by computing the 2*p*_O_ MO‐coefficient on the nucleophilic oxygen center contributing to the overall HOMO${}_{\mathrm{HXO}{}^{-}}$ orbital, which is for HOMO${}_{\mathrm{HSO}{}^{-}}$ significantly smaller than for HOMO${}_{\mathrm{HOO}{}^{-}}$ , where the donor–acceptor interaction is nearly absent, namely, 0.79 and 0.97, respectively. We have validated the role of the stabilizing 2*p*_O_–3*d*_X_ donor–acceptor orbital interaction on the stability and shape of the HOMO${}_{\mathrm{HXO}{}^{-}}$ by performing an additional bonding analysis where the empty acceptor orbitals on the HX radical fragments (3*d*_O_ and 3*d*_S_) are artificially removed (see SI Figure S18). As expected, in absence of the empty 3*d*_X_ AOs, and hence without the stabilizing 2*p*_O_–3*d*_X_ donor–acceptor orbital interaction, the HOMO${}_{\mathrm{HSO}{}^{-}}$ is higher energy than the HOMO${}_{\mathrm{HOO}{}^{-}}$ (HOMO${}_{\mathrm{HSO}{}^{-}}$ =3.7 eV; HOMO${}_{\mathrm{HOO}{}^{-}}$ =3.3 eV), due to the priory discussed larger repulsive orbital overlap. In addition, the shapes of both orbitals are nearly identical, due to the lack of polarizing effect induced by the empty 3*d*_S_ AO. These results confirm the importance of the 2*p*_O_–3*d*_X_ donor– acceptor orbital interaction on both the stability and shape of the HOMO${}_{\mathrm{HXO}{}^{-}}$ .

We find that bulk solvation, in general, stabilizes the HOMO of the (α‐)nucleophile by decreasing the electron‐donating capabilities, rendering both a significantly less reactive and basic (α‐)nucleophile (see Figures S21, S22, and Tables S16, S17). These results are in line with Bierbaum and co‐workers, which found that coordination of a single water molecule to the (α‐)nucleophile resulted in a higher reaction barrier and lower proton affinity compared to bare (α‐)nucleophiles.^[4e]^ Interestingly, they established that the reaction efficiency decreases faster as a function of proton affinity for monosolvated nucleophiles than their unsolvated counterparts. Our Brønsted‐type correlation diagrams in bulk solution (both in dichloromethane and water) reveal that the large degree of α‐effect for the strong α‐nucleophiles (i.e., HOO^−^, H_2_NO^−^), is maintained. We found that, in line with the gas‐phase results, the HOMO of the α‐effect exhibiting α‐nucleophiles (i) has a smaller lobe on the nucleophilic center and (ii) is higher‐lying in energy than the analogous HOMO of the normal nucleophile (see SI Figures S23 and S24). These findings indicate that the intrinsic properties of the α‐nucleophile may also contribute to the α‐effect in bulk solvation.

### Origin of Basicity

Lastly, we wish to establish why this (steric) Pauli repulsion reduction mechanism is not manifested in the corresponding basicity (i.e., Δ*H*_PA_; proton affinity), which ultimately leads to the observed downward deviation from the reactivity‐basicity correlation and hence the α‐effect. Table 1 shows the activation strain and energy decomposition analyses for the interaction of HO^−^ and HOO^−^ with H^+^ forming HO−H and HOO−H, respectively (see SI Table S15 for the EDA data of all nucleophiles). In analogy with our previous analysis of the reactivity (Figure 3 and SI Table S8), the Δ*V*_elstat_ is less stabilizing for the α‐nucleophile, while the Δ*E*_oi_ is nearly similar for both nucleophiles. In contrast with the S_N_2 reaction, there is no contribution of the (steric) Pauli repulsion (Δ*E*_Pauli_) in the proton affinity, because H^+^ does not have any electrons which can engage in a repulsive occupied–occupied orbital interaction with the nucleophile. This lack of destabilizing (steric) Pauli repulsion in the proton affinity, gives rise to the deviation from the classical Brønsted‐type correlation between reactivity and basicity, because a smaller HOMO${}_{\mathrm{Nu}:{}^{-}}$ lobe of α‐nucleophiles has a more significant impact on the reactivity of the nucleophile than on the basicity. In other words, the basicity of (α‐)nucleophiles is determined by the electrostatic and orbital interactions between the nucleophile and H^+^. The reactivity, on the other hand, is not only controlled by the prior mentioned stabilizing energy terms but also the destabilizing (steric) Pauli repulsion. This renders α‐nucleophiles with α‐effect to be more reactive, based on their basicity, because the electrostatic interactions are as always significantly less stabilizing for the α‐nucleophiles, while this is compensated in the reactivity by the less destabilizing (steric) Paul repulsion. To compensate for this intrinsic deviation, one could use the carbon basicity (e.g., ethyl cation affinity, Δ*H*_EtA_), instead of the proton basicity, introducing also (steric) Pauli repulsion in the basicity term.^[13]^ Even though this significantly reduces the downward deviation of α‐nucleophiles compared to the classical Brønsted‐type correlation, the α‐effect is still present for the strong α‐nucleophiles H_2_NO^−^ and HOO^−^ (see SI Figure S20).

**Table 1:** Activation strain and energy decomposition analyses (in kcal mol^−1^) and O−H distance (in Å) for the interaction between the Nu:^−^ and H^+^ in Nu−H, where Nu:^−^=HO^−^ and HOO^−^.^[a]^ 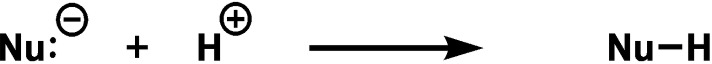

	Δ*H*_PA_	Δ*E*	Δ*E*_strain_	Δ*E*_int_	Δ*V*_elstat_	Δ*E*_Pauli_	Δ*E*_oi_	*r* (O−H)
**HO−H**	394.2	−401.5	0.0	−401.5	−224.4	0.0	−177.1	0.964
**HOO−H**	379.8	−387.4	1.0	−388.4	−211.9	0.0	−176.5	0.970

[a] Analyses at equilibrium geometries. Computed at ZORA‐OLYP/QZ4P.

## Conclusion

The present computational study on a series of *O*‐, *N*‐ and *S*‐based normal and α‐nucleophiles participating in an S_N_2 reaction with ethyl chloride identifies three distinct groups of α‐nucleophiles: (i) α‐nucleophiles with a downward deviation from the classical Brønsted‐type reactivity‐basicity correlation and hence exhibiting α‐effect, i.e., acceleration (e.g., HOO^−^, H_2_NO^−^, CH_3_OO^−^, CH_3_HNO^−^, HOHN^−^); (ii) α‐nucleophiles, primarily *S*‐ and *N*‐based, showing a minor or no degree of α‐effect and thus behaving like their parent normal counterpart; (iii) α‐nucleophiles showing a degree of inverse α‐effect, rendering them less reactive than their parent normal analog.

Our activation strain and Kohn–Sham molecular orbital analyses elucidate the underlying electronic mechanism behind the α‐effect. In contrast to the current rationales, we found that α‐nucleophiles exhibiting α‐effect are more reactive than their normal analogs due to less repulsive occupied–occupied orbital overlap between the nucleophile and substrate. The adjacent electronegative atom of α‐nucleophiles can polarize orbital density away from the nucleophilic center, resulting in a smaller HOMO lobe, and thus less (steric) Pauli repulsion between the reactants. Strikingly, the significantly smaller HOMO lobe of the α‐nucleophile can still engage in a similar orbital interaction as its parent normal nucleophile. This can be traced back to the significantly higher‐lying HOMO of the α‐nucleophiles, which results in a smaller HOMO_nucleophile_–LUMO_substrate_ orbital energy gap and hence compensates for the loss in overlap. In all, α‐nucleophiles need to fulfill the following two requirements to show strong α‐effect:

1. The HOMO of the α‐nucleophile should have a *small orbital lobe on the nucleophilic center* to reduce (steric) Pauli repulsion with the substrate.

2. The HOMO of the α‐nucleophile should be sufficiently *high in energy* (relative to the normal nucleophile) to engage in a strong orbital interaction with the substrate, by compensating for the reduced favorable HOMO–LUMO overlap.

If one of these two criteria are not fulfilled, one can expect no α‐effect or inverse α‐effect. Therefore, one only needs to analyze the electronic structure of an α‐nucleophile to determine if it will exhibit α‐effect based on its intrinsic properties.

This (steric) Pauli repulsion reduction mechanism has, however, no effect on the basicity (i.e., proton affinity) of the α‐nucleophiles since H^+^ has no electrons and, therefore, cannot engage in a repulsive occupied–occupied orbital interaction. This ultimately leads to the deviation from the classical Brønsted‐type correlation between reactivity and basicity. We believe that this is a finding which has an impact on many Brønsted‐type correlations, in which one can expect deviation as a result of the polarization of filled orbitals, leading to less (steric) Pauli repulsion.

## Conflict of interest

The authors declare no conflict of interest.

## Supporting information

As a service to our authors and readers, this journal provides supporting information supplied by the authors. Such materials are peer reviewed and may be re‐organized for online delivery, but are not copy‐edited or typeset. Technical support issues arising from supporting information (other than missing files) should be addressed to the authors.

Supporting Information
