# Supplementary material for: Origin of the α‐Effect in SN2 Reactions
Source: Angew Chem Int Ed Engl. 2021 Jul 26;60(38):20840–8. doi: 10.1002/anie.202106053 (PMC8518820; doi:10.1002/anie.202106053)
Supplement: Supplementary file 1 — Supporting Information [file ANIE-60-20840-s001.pdf]

Angewandte Chemie
Eine Zeitschrift der Gesellschaft Deutscher Chemiker

Supporting Information

Origin of the α-Effect in S_N2 Reactions

*Thomas Hansen[+], Pascal Vermeeren[+], F. Matthias Bickelhaupt, and Trevor A. Hamlin**

anie_202106053_sm_miscellaneous_information.pdf

Contents

Computational Method

Figure S1. Brønsted-type correlation between the reaction barrier (*i.e.*, $\Delta H^{\ddagger}$; kinetics) and the basicity (*i.e.*, ΔH_{PA}; thermodynamics) for the S_N2 reaction of Nu:⁻ + C_2H_5F (*i.e.*, ethyl fluoride). The normal nucleophiles (*i.e.*, R–Y:⁻) are indicated in black. The α-nucleophiles (*i.e.*, R–X–Y:⁻) have three distinct classes: class I, exhibiting α-effect, teal dots; class II, having a minor degree or no α-effect, grey dots; class III, showing inverse α-effect, red dots, where X, Y = O, HN, S and R = H, CH_3. The linear trend line (black dotted line; R^2 = 0.97) is fitted to the normal nucleophile data set. Computed at ZORA-OLYP/QZ4P.

Figure S2. Brønsted-type correlation between the reaction barrier (*i.e.*, $\Delta H^{\ddagger}$; kinetics) and the basicity (*i.e.*, ΔH_{PA}; thermodynamics) for the S_N2 reaction of (a) Nu:⁻ + CH_3F (*i.e.*, methyl fluoride) (b) Nu:⁻ + C_3H_7F (*i.e.*, isopropyl fluoride). The normal nucleophiles (*i.e.*, R–Y:⁻) are indicated in black. The α-nucleophiles (*i.e.*, R–X–Y:⁻) have three distinct classes: class I, exhibiting α-effect, teal dots; class II, having a minor degree or no α-effect, grey dots; class III, showing inverse α-effect, red dots, where X, Y = O, HN, S and R = H, CH_3. The linear trend line (black dotted line; R^2 = [a] 0.94; [b] 0.95) is fitted to the normal nucleophile data set. Computed at ZORA-OLYP/QZ4P.

Figure S3. Brønsted-type correlation between the reaction barrier (*i.e.*, $\Delta H^{\ddagger}$; kinetics) and the basicity (*i.e.*, ΔH_{PA}; thermodynamics) for the S_N2 reaction of (a) Nu:⁻ + CH_3Cl (*i.e.*, methyl chloride) (b) Nu:⁻ + C_3H_7Cl (*i.e.*, isopropyl chloride). The normal nucleophiles (*i.e.*, R–Y:⁻) are indicated in black. The α-nucleophiles (*i.e.*, R–X–Y:⁻) have three distinct classes: class I, exhibiting α-effect, teal dots; class II, having a minor degree or no α-effect, grey dots; class III, showing inverse α-effect, red dots, where X, Y = O, HN, S and R = H, CH_3. The linear trend line (black dotted line; R^2 = [a] 0.92; [b] 0.94) is fitted to the normal nucleophile data set. Computed at ZORA-OLYP/QZ4P.

Figure S4. Brønsted-type correlation between the reaction barrier (*i.e.*, $\Delta H^{\ddagger}$; kinetics) and the basicity (*i.e.*, ΔH_{PA}; thermodynamics) for the S_N2 reaction of Nu:⁻ + C_2H_5Cl (*i.e.*, ethyl chloride). The normal nucleophiles (*i.e.*, R–Y:⁻) are indicated in black. The α-nucleophiles (*i.e.*, FO⁻, ClO⁻, BrO⁻, IO⁻) have three distinct classes: class I, exhibiting α-effect, teal dots; class II, having a minor degree or no α-effect, grey dots. The linear trend line (black dotted line; R^2 = 0.93) is fitted to the normal nucleophile data set. Computed at ZORA-OLYP/QZ4P.

Figure S5. Brønsted-type correlation between the reaction barrier (*i.e.*, $\Delta H^{\ddagger}$; kinetics) and the basicity (*i.e.*, ΔH_{PA}; thermodynamics) for the S_N2 reaction of Nu:⁻ + C_2H_5Cl, computed at (a) ZORA-M06-2X/QZ4P//ZORA-OLYP/QZ4P; (b) ZORA-M06-2X-D3/QZ4P//ZORA-OLYP/QZ4P. The normal nucleophiles (*i.e.*, R–Y:⁻) are indicated in black. The α-nucleophiles (*i.e.*, R–X–Y:⁻) have three distinct classes: class I, exhibiting α-effect, teal dots; class II, having a minor degree or no α-effect, grey dots; class III, showing inverse α-effect, red dots, where X, Y = O,

HN, S and R = H, CH_3. The linear trend line (black dotted line; R^2 = [a] 0.92; [b] 0.92) is fitted to the normal nucleophile data set.

Figure S6. Brønsted-type correlation between the reaction barrier (*i.e.*, $\Delta H^{‡}$; kinetics) and the basicity (*i.e.*, ΔH_{PA}; thermodynamics) for the S_N2 reaction of Nu:$^-$ + C_2H_5Cl, computed at (a) ZORA-B3LYP-D3(BJ)/QZ4P//ZORA-OLYP/QZ4P; (b) ZORA-OLYP-D3(BJ)/QZ4P//ZORA-OLYP/QZ4P. The normal nucleophiles (*i.e.*, R–Y:$^-$) are indicated in black. The α-nucleophiles (*i.e.*, R–X–Y:$^-$) have three distinct classes: class I, exhibiting α-effect, teal dots; class II, having a minor degree or no α-effect, grey dots; class III, showing inverse α-effect, red dots, where X, Y = O, HN, S and R = H, CH_3. The linear trend line (black dotted line; R^2 = [a] 0.91; [b] 0.93) is fitted to the normal nucleophile data set.

Figure S7. (a,c,e) Activation strain analysis; and (b,d,f) energy decomposition analysis of the S_N2 reactions between the normal nucleophile (HO^-, black) and α-nucleophile (HOO^-, red; H_2NO^-, blue; HSO^-, green) + C_2H_5Cl, along the IRC projected on the C^{α}•••Cl bond stretch. Computed at ZORA-OLYP/QZ4P.

Figure S8. (a,c,e) Activation strain analysis; and (b,d,f) energy decomposition analysis of the S_N2 reactions between the normal nucleophile (CH_3O^-, black) and α-nucleophile (CH_3OO^-, red; CH_3HNO^-, blue; CH_3SO^-, green) + C_2H_5Cl, along the IRC projected on the C^{α}•••Cl bond stretch. Computed at ZORA-OLYP/QZ4P.

Figure S9. a) Activation strain analysis; and b) energy decomposition analysis of the S_N2 reactions between HO^- (black; normal nucleophile) and HOO^- (red; α-nucleophile) + C_3H_7Cl, along the IRC projected on the C^{α}•••Cl bond stretch. Computed at ZORA-OLYP/QZ4P.

Figure S10. a) Activation strain analysis; and b) energy decomposition analysis of the S_N2 reactions between HO^- (black; normal nucleophile) and HOO^- (red; α-nucleophile) + C_2H_5Cl, along the IRC projected on the C^{α}•••Cl bond stretch. Computed at ZORA-M06-2X/QZ4P//ZORA-OLYP/QZ4P.

Figure S11. a) Activation strain analysis; and b) energy decomposition analysis of the S_N2 reactions between HO^- (black; normal nucleophile) and HOO^- (red; α-nucleophile) + C_2H_5Cl, along the IRC projected on the C^{α}•••Cl bond stretch. Computed at ZORA-M06-2X-D3/QZ4P//ZORA-OLYP/QZ4P.

Figure S12. a) Activation strain analysis; and b) energy decomposition analysis of the S_N2 reactions between HO^- (black; normal nucleophile) and HOO^- (red; α-nucleophile) + C_2H_5Cl, along the IRC projected on the C^{α}•••Cl bond stretch. Computed at ZORA-B3LYP-D3(BJ)/QZ4P//ZORA-OLYP/QZ4P.

Figure S13. a) Activation strain analysis; and b) energy decomposition analysis of the S_N2 reactions between HO^- (black; normal nucleophile) and HOO^- (red; α-nucleophile) + C_2H_5Cl,

along the IRC projected on the C$^{\alpha}$•••Cl bond stretch. Computed at ZORA-OLYP-D3(BJ)/QZ4P//ZORA-OLYP/QZ4P.

Figure S14. Key occupied orbitals ($HOMO_{Nu:-}$; energies in eV; isovalue = 0.030 Bohr$^{-3/2}$) for FO^{-}, ClO^{-}, BrO^{-},and IO^{-} computed at equilibrium geometries, where the gray horizontal line indicates the maximum spatial extent of the $HOMO_{HO-}$. Computed at ZORA-OLYP/QZ4P.

Figure S15. Key occupied orbitals ($HOMO_{Nu:-}$; energies in eV; isovalue = 0.030 Bohr$^{-3/2}$) for $HSHN^{-}$, CH_3O^{-}, and CH_3HNO^{-} computed at equilibrium geometries, where the gray horizontal line indicates the maximum spatial extent of the $HOMO_{H_3CO-}$. Computed at ZORA-OLYP/QZ4P. [a] HOMO–1 is the key occupied orbital.

Figure S16. The construction of the $HOMO_{HOO-}$ (left) and $HOMO_{HSO-}$ (right) from the interaction between the filled 2*p* atomic orbital of the nucleophilic oxygen center ($2p_O$) and the filled n*p* and empty 3*d* atomic orbitals of the adjacent oxygen and sulfur atom (np_X and $3d_X$), where the orbital energies shown in red are computed in the presence of the other fragment (in eV) and the population of the empty $3d_X$ atomic orbitals are shown in blue (in electrons). Computed at ZORA-OLYP/QZ4P.

Figure S17. Contour plot of the atomic orbitals of $2p_O$ of the nucleophilic oxygen center (black lines), the $2p_O$ (red lines) and the $3p_S$ (blue lines). All contour plots contain 2 contours from 0.0195–0.2000 au.

Figure S18. The construction of the $HOMO_{HOO-}$ (left) and $HOMO_{HSO-}$ (right) from the interaction between the filled 2*p* atomic orbital of the nucleophilic oxygen center ($2p_O$) and the filled np_X, where the orbital energies shown in red are computed in the presence of the other fragment are (in eV) and the empty orbitals are artificially removed. Computed at ZORA-OLYP/QZ4P.

Figure S19. Schematic representation of the construction of the $HOMO_{FO-}$ (left, red) and $HOMO_{IO-}$ (right, green) from the interaction between the filled 2*p* atomic orbital of the nucleophilic oxygen center (middle, black, $2p_O$) and the filled n*p* and empty n*d* atomic orbitals of the adjacent fluorine and iodine atom (np_X and nd_X).

Figure S20. Brønsted-type correlation between the reaction barrier (*i.e.*, $\Delta H^{\ddagger}$; kinetics) and the carbon basicity (*i.e.*, ΔH_{EtA}; ethyl cation affinity; thermodynamics) for the S_N2 reaction of (a) $Nu:^{-}$ + C_2H_5Cl. The normal nucleophiles (*i.e.*, R–Y:$^{-}$) are indicated in black. The α-nucleophiles (*i.e.*, R–X–Y:$^{-}$) have three distinct classes: class I, exhibiting α-effect, teal dots; class II, having a minor degree or no α-effect, grey dots; class III, showing inverse α-effect, red dots, where X, Y = O, HN, S and R = H, CH_3. The linear trend line (black dotted line; R^2 = 0.90) is fitted to the normal nucleophile data set. Computed at ZORA-OLYP/QZ4P.

Figure S21. Brønsted-type correlation between the reaction barrier (*i.e.*, $\Delta H^{\ddagger}$; kinetics) and the carbon basicity (*i.e.*, ΔH_{PA}; proton affinity; thermodynamics) for the S_N2 reaction of (a) $Nu:^{-}$ + C_2H_5Cl. The normal nucleophiles (*i.e.*, R–Y:$^{-}$) are indicated in black (R = H, [a]; R = CH_3, [b]).

The α-nucleophiles (*i.e.*, R–X–Y:⁻) have three distinct classes: class I, exhibiting α-effect, teal dots; class II, having a minor degree or no α-effect, grey dots; class III, showing inverse α-effect, red dots, where X, Y = O, HN, S and R = H, CH_3. The linear trend line (black dotted line; R^2 = [a] 0.96; [b] 0.99) is fitted to the normal nucleophile data set. Computed at COSMO(DCM)ZORA-OLYP/QZ4P.

Figure S22. Brønsted-type correlation between the reaction barrier (*i.e.*, $\Delta H^{\ddagger}$; kinetics) and the carbon basicity (*i.e.*, ΔH_{PA}; proton affinity; thermodynamics) for the S_N2 reaction of (a) Nu:⁻ + C_2H_5Cl. The normal nucleophiles (*i.e.*, R–Y:⁻) are indicated in black (R = H, [a]; R = CH_3, [b]). The α-nucleophiles (*i.e.*, R–X–Y:⁻) have three distinct classes: class I, exhibiting α-effect, teal dots; class II, having a minor degree or no α-effect, grey dots; class III, showing inverse α-effect, red dots, where X, Y = O, HN, S and R = H, CH_3. The linear trend line (black dotted line; R^2 = [a] 0.90; [b] 0.99) is fitted to the normal nucleophile data set. Computed at COSMO(Water)ZORA-OLYP/QZ4P.

Figure S23. Key occupied orbitals ($HOMO_{Nu:-}$; energies in eV; isovalue = 0.030 $Bohr^{-3/2}$ for *O*- and *N*-nucleophiles and isovalue = 0.035 $Bohr^{-3/2}$ for *S*-nucleophiles) computed at equilibrium geometries. Computed at COSMO(DCM)-ZORA-OLYP/QZ4P. [a] HOMO–1 is the key occupied orbital.

Figure S24. Key occupied orbitals ($HOMO_{Nu:-}$; energies in eV; isovalue = 0.030 $Bohr^{-3/2}$ for *O*- and *N*-nucleophiles and isovalue = 0.035 $Bohr^{-3/2}$ for *S*-nucleophile) computed at equilibrium geometries. Computed at COSMO(Water)-ZORA-OLYP/QZ4P. [a] HOMO–1 is the key occupied orbital.

Figure S25. Representation of the DFT $HOMO_{Nu:-}$ (isovalue = 0.03 $Bohr^{-3/2}$) of HO^-, HOO^-, and CH_3O^-, where the grey horizontal line indicates the maximum spatial extent of the $HOMO_{HO-}$.

Table S1. Electronic energies relative to reactants (in kcal mol^{-1}) of the stationary points occurring in S_N2 reaction between Nu:⁻ + C_2H_5Cl.

Table S2. Computed proton affinities (ΔH_{PA}) and S_N2 reaction barrier enthalpies ($\Delta H^{\ddagger}$) for Nu:⁻ + C_2H_5Cl.

Table S3. Computed proton affinities (ΔH_{PA}) and S_N2 reaction barrier enthalpies ($\Delta H^{\ddagger}$) Nu:⁻ + $C_nH_{2n+1}Y$, in which n = 1, 2, 3 and Y = F, Cl.

Table S4. Computed proton affinities (ΔH_{PA}) and S_N2 reaction barrier enthalpies ($\Delta H^{\ddagger}$) for Nu:⁻ + C_2H_5Cl at ZORA-M06-2X/QZ4P//ZORA-OLYP/QZ4P.

Table S5. Computed proton affinities (ΔH_{PA}) and S_N2 reaction barrier enthalpies ($\Delta H^{\ddagger}$) for Nu:⁻ + C_2H_5Cl at ZORA-M06-2X-D3/QZ4P//ZORA-OLYP/QZ4P.

Table S6. Computed proton affinities (ΔH_{PA}) and S_N2 reaction barrier enthalpies ($\Delta H^{\ddagger}$) for $Nu{:}^-$ + C_2H_5Cl at ZORA-B3LYP-D3(BJ)/QZ4P//ZORA-OLYP/QZ4P.

Table S7. Computed proton affinities (ΔH_{PA}) and S_N2 reaction barrier enthalpies ($\Delta H^{\ddagger}$) for $Nu{:}^-$ + C_2H_5Cl at ZORA-OLYP-D3(BJ)/QZ4P//ZORA-OLYP/QZ4P.

Table S8. Activation strain and energy decomposition analyses (in kcal mol^{-1}) for the S_N2 reaction between $Nu{:}^-$ + C_2H_5Cl.

Table S9. Activation strain and energy decomposition analyses (in kcal mol^{-1}) for the S_N2 reaction between $Nu{:}^-$ + C_2H_5Cl.[a]

Table S10. Most important occupied–occupied orbital overlaps for the S_N2 reaction of $Nu{:}^-$ + C_2H_5Cl between $HOMO_{Nu:^-}$ and the filled orbitals on the substate.

Table S11. Activation strain and energy decomposition analyses (in kcal mol^{-1}) for the S_N2 reaction between $Nu{:}^-$ + C_2H_5F.

Table S12. Activation strain and energy decomposition analyses (in kcal mol^{-1}) for the S_N2 reaction between $RO{:}^-$ + C_2H_5Cl, in which R = H, F, Cl, Br, I.[a]

Table S13. Voronoi deformation density (VDD) atomic charges (Q^{VDD}; in electrons) of the nucleophilic centers of the studied normal and α-nucleophiles.

Table S14. Activation strain and energy decomposition analyses (in kcal mol^{-1}) for the S_N2 reaction between $Nu{:}^-$ + C_2H_5Cl

Table S15. Activation strain and energy decomposition analyses (in kcal mol^{-1}) for the interaction between the $Nu{:}^-$ and H^+ in Nu–H.[a]

Table S16. Computed proton affinities (ΔH_{PA}) and S_N2 reaction barrier enthalpies ($\Delta H^{\ddagger}$) at COSMO(DCM)ZORA-OLYP/QZ4P.

Table S17. Computed proton affinities (ΔH_{PA}) and S_N2 reaction barrier enthalpies ($\Delta H^{\ddagger}$) at COSMO(Water)ZORA-OLYP/QZ4P.

Table S18. Cartesian coordinates (Å), energies (in kcal mol^{-1}), and number of imaginary vibrational frequencies (N_{imag}) of the S_N2 reaction between $Nu{:}^-$ + C_2H_5Cl, computed at ZORA-OLYP/QZ4P.

Table S19. Cartesian coordinates (Å), energies (in kcal mol^{-1}), and number of imaginary vibrational frequencies (N_{imag}) of the S_N2 reaction between $Nu{:}^-$ + C_2H_5F, computed at ZORA-OLYP/QZ4P.

Table S20. Cartesian coordinates (Å), energies (in kcal mol^{-1}), and number of imaginary vibrational frequencies (N_{imag}) of the S_N2 reaction between Nu:$^-$ + CH_3F, computed at ZORA-OLYP/QZ4P.

Table S21. Cartesian coordinates (Å), energies (in kcal mol^{-1}), and number of imaginary vibrational frequencies (N_{imag}) of the S_N2 reaction between Nu:$^-$ + CH_3Cl, computed at ZORA-OLYP/QZ4P.

Table S22. Cartesian coordinates (Å), energies (in kcal mol^{-1}), and number of imaginary vibrational frequencies (N_{imag}) of the S_N2 reaction between Nu:$^-$ + C_3H_7F, computed at ZORA-OLYP/QZ4P.

Table S23. Cartesian coordinates (Å), energies (in kcal mol^{-1}), and number of imaginary vibrational frequencies (N_{imag}) of the S_N2 reaction between Nu:$^-$ + C_3H_7Cl, computed at ZORA-OLYP/QZ4P.

Table S24. Cartesian coordinates (Å), energies (in kcal mol^{-1}), and number of imaginary vibrational frequencies (N_{imag}) of the S_N2 reaction between Nu:$^-$ + C_2H_5Cl, computed at COSMO(DCM)-ZORA-OLYP/QZ4P.

Table S25. Cartesian coordinates (Å), energies (in kcal mol^{-1}), and number of imaginary vibrational frequencies (N_{imag}) of the S_N2 reaction between Nu:$^-$ + C_2H_5Cl, computed at COSMO(Water)-ZORA-OLYP/QZ4P.

Computational Method

Computational details

All density functional theory (DFT) calculations were performed using the Amsterdam Density Functional (ADF2018.105) software package.[1] The generalized gradient approximation (GGA) exchange-correlation functional OLYP was used for all computations, which consists of the optimized exchange (OPTX) functional proposed by Handy and co-workers,[2a] and the Lee–Yang–Parr (LYP) correlation functional.[2b] Our previous benchmark studies have shown that OLYP reproduces S_N2 barriers from highly correlated *ab initio* within only a few kcal mol^{-1}.[3] Scalar relativistic effects are accounted for using the zeroth-order regular approximation (ZORA).[4] The basis set used, denoted QZ4P, is of quadruple-ζ quality for all atoms and has been improved by four sets of polarization functions.[5] This large basis set is required for small anionic species such as HO^{-}.[3] Additionally, single-point energies were computed at ZORA-M06-2X[6]/QZ4P, ZORA-M06-2X-D3[6,7]/QZ4P, ZORA-OLYP-D3(BJ)[2,7]/QZ4P, and ZORA-B3LYP-D3(BJ)[8]/QZ4P on fully optimized ZORA-OLYP/QZ4P geometries to identify the effect of hybrid, meta-hybrid, and dispersion-corrected exchange-correlation functionals on the computed reactivity trends. In addition, stationary points were re-optimized at COSMO[9](DCM)-ZORA-OLYP/QZ4P and COSMO(Water)-ZORA-OLYP/QZ4P in order to assess the effect of solvation on the computed reactivity trends. These data all show the same trends in reactivity for the investigated reactions. The accuracies of the fit scheme (Zlm fit) and the integration grid (Becke

---

[1] a) G. te Velde, F. M. Bickelhaupt, E. J. Baerends, C. Fonseca Guerra, S. J. A. van Gisbergen, J. G. Snijders, T. Ziegler, *J. Comput. Chem.* **2001**, *22*, 931; b) C. Fonseca Guerra, J. G. Snijders, G. te Velde, E. J. Baerends, *Theor. Chem. Acc.* **1998**, *99*, 391; c) ADF 2018.105, SCM Theoretical Chemistry, Vrije Universiteit: Amsterdam (The Netherlands), **2018** http://www.scm.com; d) M.; Swart, F. M. Bickelhaupt, *J. Comput. Chem.* **2008**, *29*, 724.

[2] a) N. C. Handy, A. J. Cohen, *Mol. Phys.* **2001**, 99, 403; b) C. Lee, W. Yang, R. G. Parr, *Phys. Rev. B: Condens. Matter Mater. Phys.* **1988**, *37*, 785.

[3] a) A. P. Bento, F. M. Bickelhaupt, M. Solà, *J. Comp. Chem.* **2005**, *26*, 1497; b) M. Swart, M. Solà, F. M. Bickelhaupt, *J. Chem. Theory Comput.* **2010**, 6, 3145.

[4] E. van Lenthe, E. J. Baerends, J. G. Snijders, *J. Chem. Phys.* **1994**, 101, 9783.

[5] E. van Lenthe, E. J. Baerends, *J. Comput. Chem.* **2003**, *24*, 1142.

[6] a) Y. Zhao and D. G. Truhlar, *J. Chem. Phys*. **2006**, *125*, 194101; b) Y. Zhao and D. G. Truhlar, *Theor. Chem. Acc.* **2008***, 120,* 215.

[7] a) S. Grimme, J. Antony, S. Ehrlich and H. A. Krieg, *J. Chem. Phys.* **2010**, *132*, 154104; b) S. Grimme, S. Ehrlich and L. Goerigk. *J. Comput. Chem*. **2011**, *32*, 1456.

[8] a) A. D. Becke, *J. Chem. Phys.* **1993**, *98*, 1372; b) C. Lee, W. Yang, R. G. Parr, *Phys. Rev. B* **1988** *37*, 785.

[9] a) A. Klamt, G. Schüürmann, *J. Chem. Soc. Perkin Trans. 2* **1993**, 799; b) A. Klamt, *J. Phys. Chem.* **1995**, 2224; c) A. Klamt, V. Jonas, *J. Chem. Phys.* **1996**, *105*, 9972; d) C. C. Pye, T. Ziegler, *Theor. Chem. Acc.* **1999**, *101*, 396.

grid) were, for all calculations, set to VERYGOOD.[10] No symmetry constraints were used for all computations. All calculated stationary points have been verified by performing a vibrational analysis,[11] to be energy minima (no imaginary frequencies) or transition states (only one imaginary frequency). The character of the normal mode associated with the imaginary frequency of the transition state has been inspected to ensure that it is associated with the reaction of interest. The potential energy surfaces of the studied S_N2 reactions were obtained by performing intrinsic reaction coordinate (IRC) calculations,[12] which, in turn, were analyzed using the PyFrag 2019 program.[13] The optimized structures were illustrated using CYLview.[14]

Thermochemistry

Bond enthalpies, *i.e.*, proton affinities (PA), are calculated at 298.15 K and 1 atm (ΔH_{PA}) from electronic bond energies (ΔE) and vibrational frequencies using standard thermochemistry relations for an ideal gas [Eq. (1)].[15]

$$\Delta H_{PA} = \Delta E + \Delta E_{trans,298} + \Delta E_{rot,298} + \Delta E_{vib,0} + \Delta(\Delta E_{vib,0})_{298} + \Delta(pV) \tag{1}$$

Here, $\Delta E_{trans,298}$, $\Delta E_{rot,298}$, and $\Delta E_{vib,0}$ are the differences between the complex (*i.e.*, Nu–H, the protonated Lewis bases) and the separate species (*i.e.*, $Nu:^{-} + H^{+}$, the proton and the Lewis base) in translational, rotational, and zero-point vibrational energy, respectively. The last term, $\Delta(\Delta E_{vib,0})_{298}$, is the change in the vibrational energy difference when going from 0 K to 298.15 K. The molar work term $\Delta(pV)$ is (Δn)RT, where $\Delta n = +1$, for one complex (Nu–H) dissociating into two separate species, namely the $Nu:^{-} + H^{+}$.

---

[10] a) M. Franchini, P. H. T. Philipsen, E. van Lenthe, L. Visscher, *J. Chem. Theory Comput.* **2014**, *10*, 1994; b) M. Franchini, P. H. T. Philipsen, L. Visscher, *J. Comput. Chem.* **2013**, *34*, 1819.

[11] a) A. Bérces, R. M. Dickson, L. Fan, H. Jacobsen, D. Swerhone, T. Ziegler, *Comput. Phys. Commun.* **1997**, *100*, 247; b) H. Jacobsen, A. Bérces, D. P. Swerhone, T. Ziegler, *Comput. Phys. Commun.* **1997**, *100*, 263; c) S. K. Wolff, *Int. J. Quantum Chem.* **2005**, *104*, 645.

[12] a) K. Fukui, *Acc. Chem. Res.* **1981**, *14*, 363; b) L. Deng, T. Ziegler, L. A. Fan, *J. Chem. Phys.* **1993**, *99*, 3823; c) L. Deng, T. Ziegler, *Int. J. Quantum Chem.* **1994**, *52*, 731.

[13] X. Sun, T. M. Soini, J. Poater, T. A. Hamlin, F. M. Bickelhaupt, *J. Comp. Chem.* **2019**, *40*, 2227.

[14] C. Y. Legault, CYLview, 1.0b; Université de Sherbrooke, Canada, Sherbrooke, QC, 2009, www. cylview.org.

[15] a) P. W. Atkins, J. de Pauli, *Physical Chemistry*; 9th ed., Oxford University Press: Oxford, **2010**; b) F. Jensen, *Introduction to Computational Chemistry*; 2nd ed., Wiley: West Sussex, **2007**.

Activation Strain and Energy Decomposition Analysis

The activation strain model (ASM) of chemical reactivity[16] is a fragment-based approach in which the potential energy surface (PES) can be described with respect to, and understood in terms of the characteristics of, the reactants. It considers the rigidity of the reactants and to which extent they need to deform during the reaction plus their capability to interact with each other as the reaction proceeds. With the help of this model, we decompose the total energy, $\Delta E(\zeta)$, into the strain and interaction energy, $\Delta E_{\text{strain}}(\zeta)$ and $\Delta E_{\text{int}}(\zeta)$, respectively, and project these values onto the reaction coordinate ζ [Eq. (2)].

$$\Delta E(\zeta)= \Delta E_{\text{strain}}(\zeta) + \Delta E_{\text{int}}(\zeta) \qquad (2)$$

In this equation, the strain energy, $\Delta E_{\text{strain}}(\zeta)$, is the penalty that needs to be paid in order to deform the reactants from their equilibrium to the geometry they adopt during the reaction at point ζ of the reaction coordinate. On the other hand, the interaction energy, $\Delta E_{\text{int}}(\zeta)$, accounts for all the chemical interactions that occur between these two deformed reactants along the reaction coordinate.

The interaction energy between the deformed reactants can be further analyzed in terms of quantitative Kohn-Sham molecular orbital (KS-MO) theory[17a] together with a canonical energy decomposition analysis (EDA).[17b] The EDA decomposes the $\Delta E_{\text{int}}(\zeta)$ into the following three energy terms [Eq. (3)]:

$$\Delta E_{\text{int}}(\zeta) = \Delta V_{\text{elstat}}(\zeta) + \Delta E_{\text{Pauli}}(\zeta) + \Delta E_{\text{oi}}(\zeta) \qquad (3)$$

---

[16] a) P. Vermeeren, S. C. C. van der Lubbe, C. Fonseca Guerra, F. M. Bickelhaupt, T. A. Hamlin, *Nat. Protoc.* **2020**, *15*, 649; b) I. Fernández, F. M. Bickelhaupt, *Chem. Soc. Rev.* **2014**, *43*, 4953; c) F. M. Bickelhaupt, K. N. Houk, *Angew. Chem. Int. Ed.* **2017**, *56*, 10070; d) P. Vermeeren, T. A. Hamlin, F. M. Bickelhaupt, *Chem. Comm.* **2021**, DOI: 10.1039/D1CC02042K.

[17] a) R. van Meer, O. V. Gritsenko, E. J. Baerends, *J. Chem. Theory Comput.* **2014**, *10*, 4432; b) F. M. Bickelhaupt, E. J. Baerends, Kohn-Sham Density Functional Theory: Predicting and Understanding Chemistry. In *Reviews in Computational Chemistry*; Lipkowitz, K. B.; Boyd, D. B., Eds.; Wiley-VCH: New York, **2000**, Vol. *15*, pp 1–86; c) T. A. Hamlin, P. Vermeeren, C. Fonseca Guerra, F. M. Bickelhaupt, In *Complementary Bonding Analysis*, (Eds.: S. Grabowsky), De Gruyter, Berlin, **2021**, pp 199–212.

Herein, $\Delta V_{\text{elstat}}(\zeta)$ is the classical electrostatic interaction between the unperturbed charge distributions of the (deformed) reactants and is usually attractive. The Pauli repulsion, $\Delta E_{\text{Pauli}}(\zeta)$, includes the destabilizing interaction between the fully occupied orbitals of both fragments due to the Pauli principle. The orbital interaction energy, $\Delta E_{\text{oi}}(\zeta)$, accounts for, amongst others, charge transfer between the fragments, such as HOMO–LUMO interactions.

In the herein presented activation strain and accompanied energy decomposition diagrams, the intrinsic reaction coordinate (IRC) is projected onto the carbon–leaving group (C^{α}–Cl) distance. This critical reaction coordinate undergoes a well-defined change during the reaction from the reactant complex via the transition state to the product and is shown to be a valid reaction coordinate for studying S_N2 reactions.[18]

Voronoi Deformation Density

The atomic charge distribution was analyzed by using the Voronoi Deformation Density (VDD) method.[19] The VDD method partitions the space into so-called Voronoi cells, which are non-overlapping regions of space that are closer to nucleus A than to any other nucleus. The charge distribution is determined by taking a fictitious promolecule as reference point, in which the electron density is simply the superposition of the atomic densities of all atoms A:

$\rho_{\text{promolecule}}(\text{r}) = \sum_{\text{A}} \rho_{\text{A}}(\text{r})$.

The change in density in the Voronoi cell when going from this promolecule to the final molecular density ρ (r) of the interacting system is associated with the VDD atomic charge Q. The VDD atomic charge Q_A of atom A is calculated according to [Eq. (4)].

$$Q_{\text{A}}^{\text{VDD}} = -\int_{\text{Voronoi cell of A}} [\rho(r) - \rho_{\text{promolecule}}(\text{r})]d\text{r} \tag{4}$$

So, instead of computing the amount of charge contained in an atomic volume, we compute the flow of charge from one atom to the other upon formation of the molecule. The physical

---

[18] a) T. Hansen, P. Vermeeren, A. Haim, M. J. H. van Dorp, J. D. C. Codée, F. M. Bickelhaupt, T. A. Hamlin, *Eur. J. Org. Chem.* **2020**, 3822; b) P. Vermeeren, T. Hansen, P. Jansen, M. Swart, T. A. Hamlin, F . M. Bickelhaupt, *Chem. Eur. J.* **2020**, *26*, 15538; c) P. Vermeeren, T. Hansen, M. Grasser, D. R. Silva, T. A. Hamlin, F. M. Bickelhaupt, *J. Org. Chem.* **2020**, *85*, 14087; d) T. Hansen, P. Vermeeren, R. Yoshisada, D. V. Filippov, G. A. van der Marel, J. D. C. Codée, T. A. Hamlin, *J. Org. Chem.* **2021**, *86*, 3565.

[19] C. Fonseca Guerra, J. W. Handgraaf, E. J. Baerends, F. M. Bickelhaupt, J. *Comput. Chem.* **2004**, *25*, 189.

interpretation is therefore straightforward. A positive atomic charge Q_A corresponds to the loss of electrons, whereas a negative atomic charge Q_A is associated with the gain of electrons in the Voronoi cell of atom A.

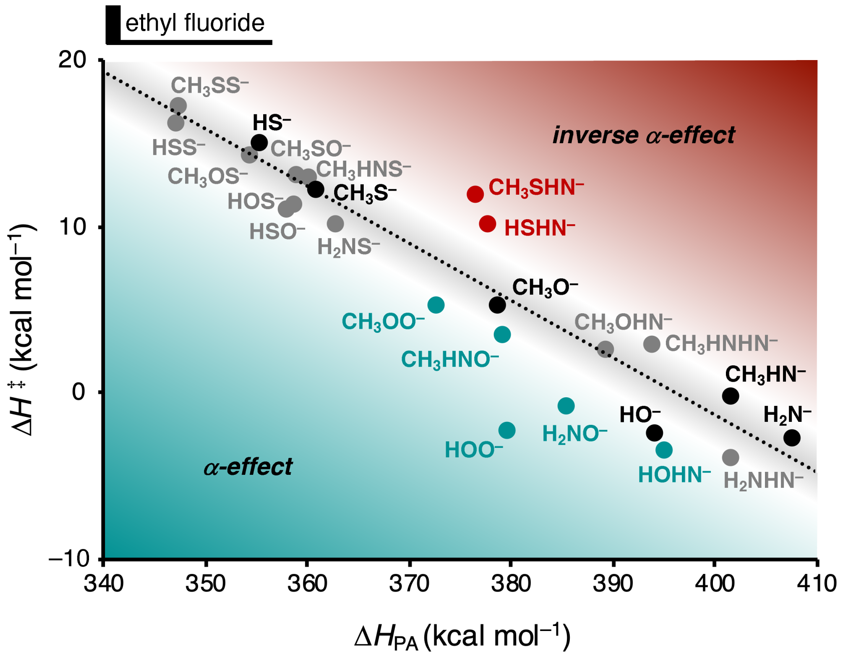

Figure S1. Brønsted-type correlation between the reaction barrier (*i.e.*, $\Delta H^{\ddagger}$; kinetics) and the basicity (*i.e.*, ΔH_{PA}; thermodynamics) for the S_N2 reaction of $Nu:^{-}$ + C_2H_5F (*i.e.*, ethyl fluoride). The normal nucleophiles (*i.e.*, R–Y:⁻) are indicated in black. The α-nucleophiles (*i.e.*, R–X–Y:⁻) have three distinct classes: class I, exhibiting α-effect, teal dots; class II, having a minor degree or no α-effect, grey dots; class III, showing inverse α-effect, red dots, where X, Y = O, HN, S and R = H, CH_3. The linear trend line (black dotted line; R^2 = 0.97) is fitted to the normal nucleophile data set. Computed at ZORA-OLYP/QZ4P.

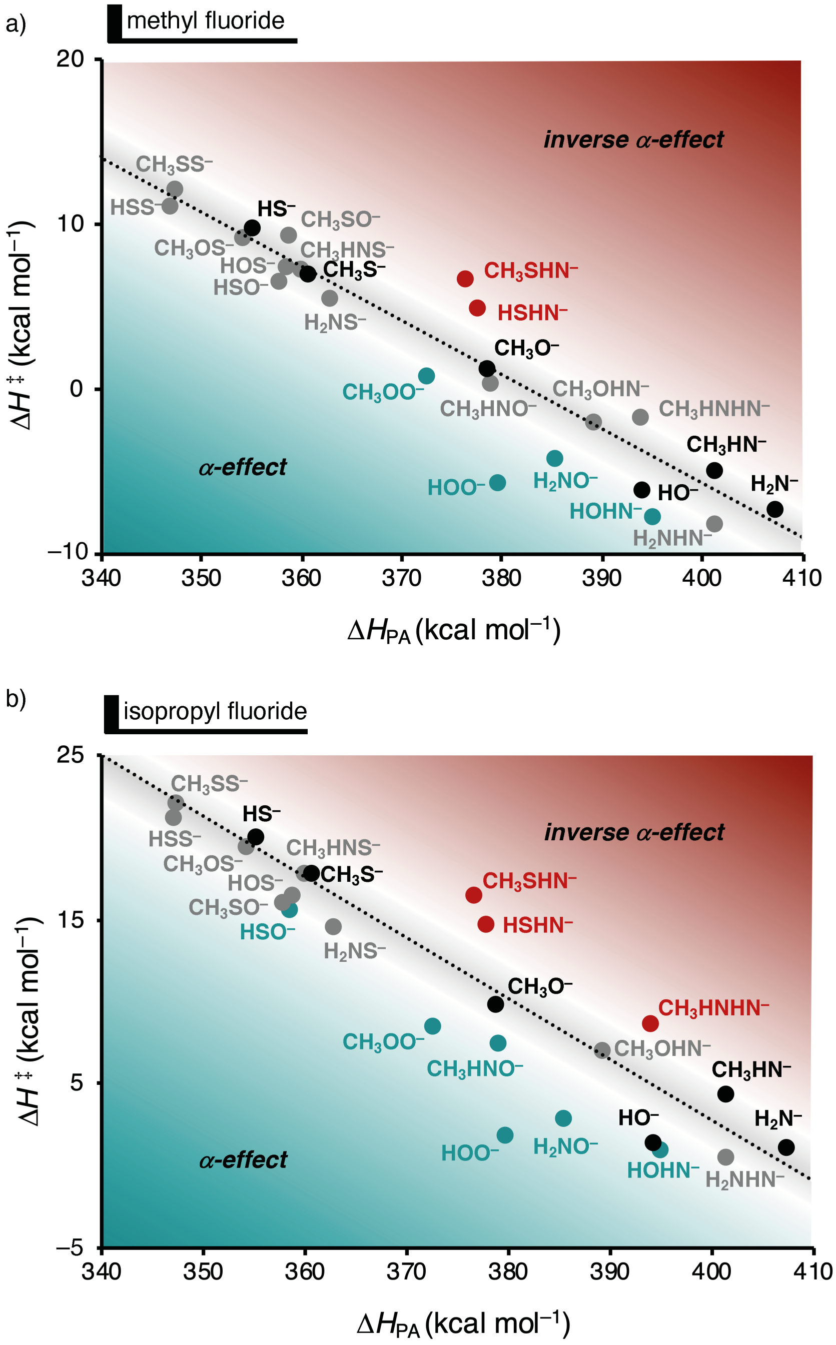

Figure S2. Brønsted-type correlation between the reaction barrier (*i.e.*, $\Delta H^{\ddagger}$; kinetics) and the basicity (*i.e.*, ΔH_{PA}; thermodynamics) for the S_N2 reaction of (a) $Nu:^{-}$ + CH_3F (*i.e.*, methyl fluoride) (b) $Nu:^{-}$ + C_3H_7F (*i.e.*, isopropyl fluoride). The normal nucleophiles (*i.e.*, R–Y:$^{-}$) are indicated in black. The α-nucleophiles (*i.e.*, R–X–Y:$^{-}$) have three distinct classes: class I, exhibiting α-effect, teal dots; class II, having a minor degree or no α-effect, grey dots; class III, showing inverse α-effect, red dots, where X, Y = O, HN, S and R = H, CH_3. The linear trend line (black dotted line; R^2 = [a] 0.94; [b] 0.95) is fitted to the normal nucleophile data set. Computed at ZORA-OLYP/QZ4P.

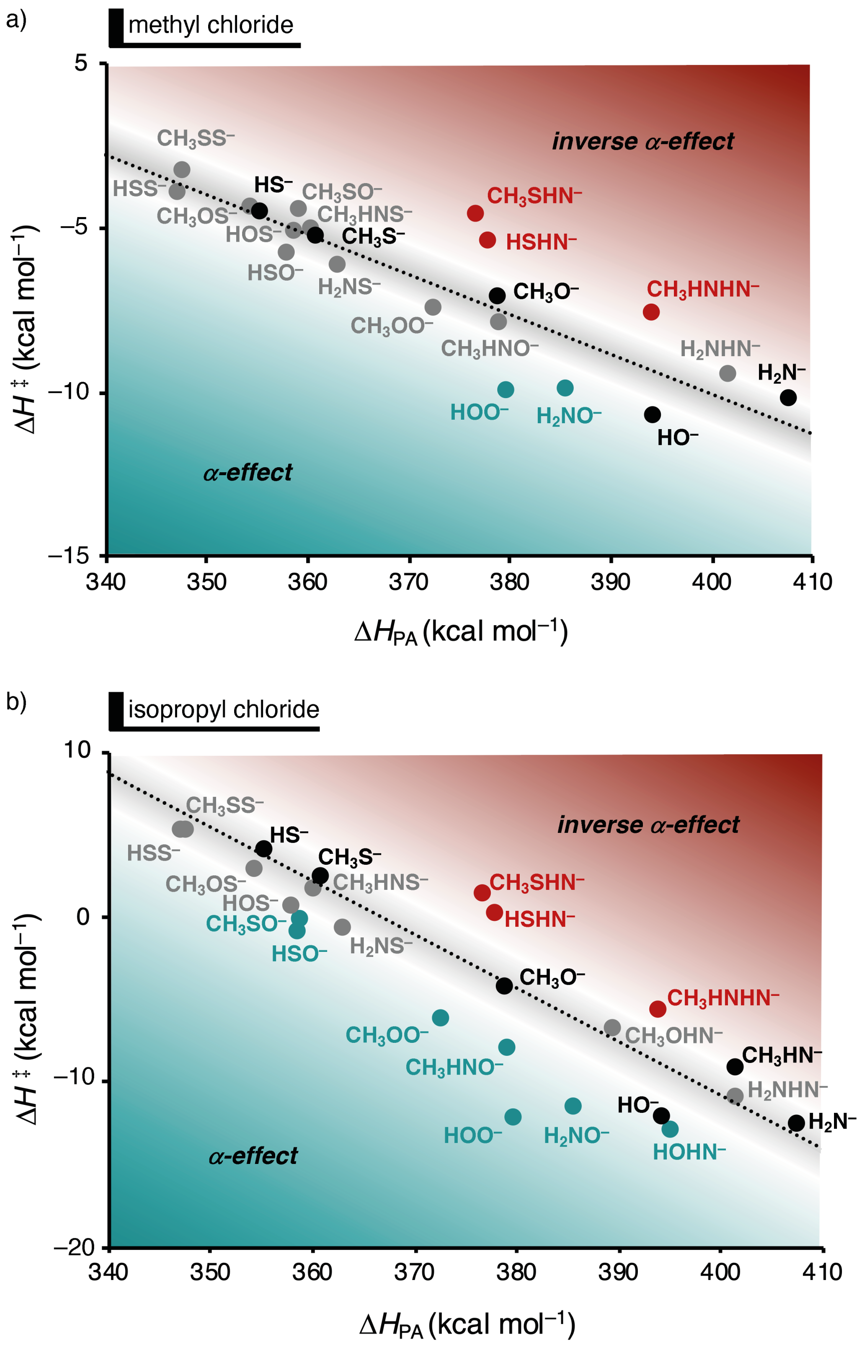

Figure S3. Brønsted-type correlation between the reaction barrier (*i.e.*, $\Delta H^{\ddagger}$; kinetics) and the basicity (*i.e.*, ΔH_{PA}; thermodynamics) for the S_N2 reaction of (a) $Nu:^{-}$ + CH_3Cl (*i.e.*, methyl chloride) (b) $Nu:^{-}$ + C_3H_7Cl (*i.e.*, isopropyl chloride). The normal nucleophiles (*i.e.*, R–Y:$^{-}$) are indicated in black. The α-nucleophiles (*i.e.*, R–X–Y:$^{-}$) have three distinct classes: class I, exhibiting α-effect, teal dots; class II, having a minor degree or no α-effect, grey dots; class III, showing inverse α-effect, red dots, where X, Y = O, HN, S and R = H, CH_3. The linear trend line (black dotted line; R^2 = [a] 0.92; [b] 0.94) is fitted to the normal nucleophile data set. Computed at ZORA-OLYP/QZ4P.

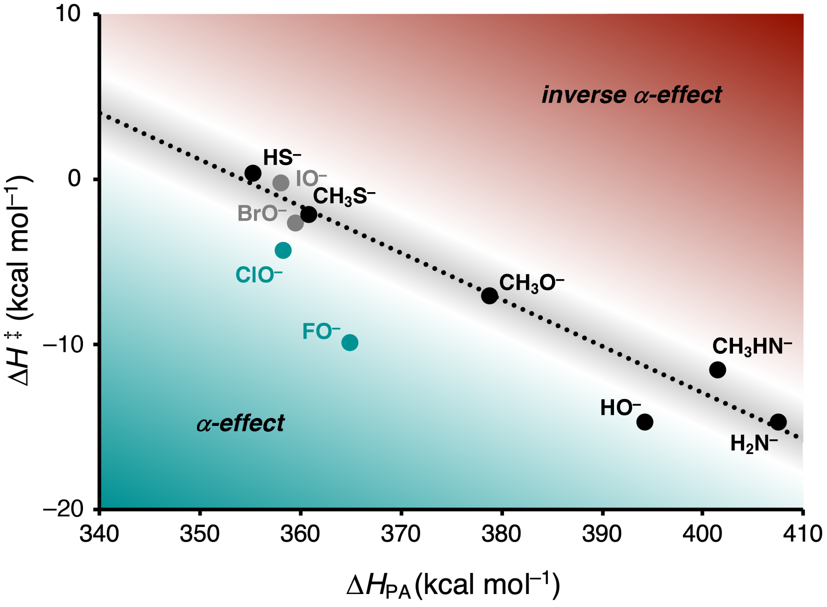

Figure S4. Brønsted-type correlation between the reaction barrier (*i.e.*, $\Delta H^{\ddagger}$; kinetics) and the basicity (*i.e.*, ΔH_{PA}; thermodynamics) for the S_N2 reaction of $Nu:^{-}$ + C_2H_5Cl (*i.e.*, ethyl chloride). The normal nucleophiles (*i.e.*, R–Y:$^{-}$) are indicated in black. The α-nucleophiles (*i.e.*, FO^{-}, ClO^{-}, BrO^{-}, IO^{-}) have three distinct classes: class I, exhibiting α-effect, teal dots; class II, having a minor degree or no α-effect, grey dots. The linear trend line (black dotted line; R^2 = 0.93) is fitted to the normal nucleophile data set. Computed at ZORA-OLYP/QZ4P.

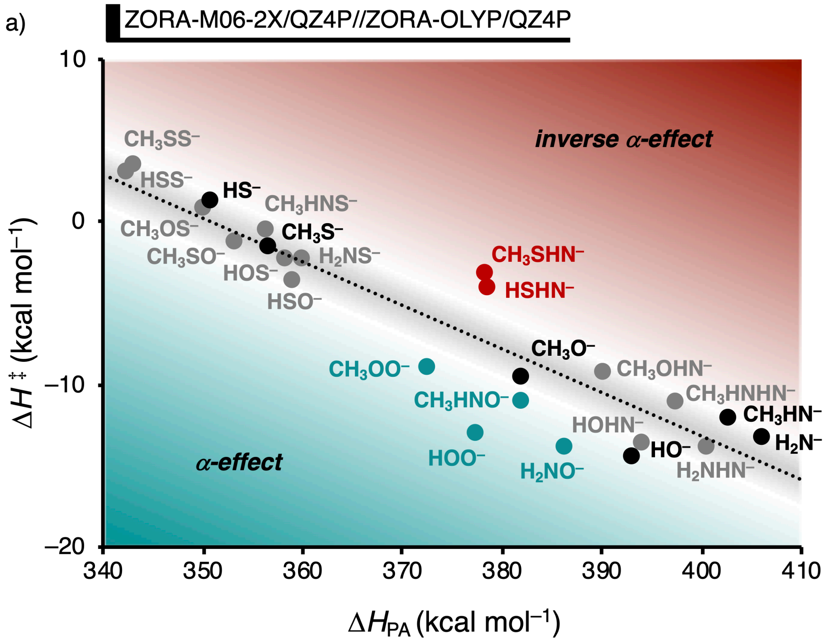

Figure S5. Brønsted-type correlation between the reaction barrier (*i.e.*, $\Delta H^{\ddagger}$; kinetics) and the basicity (*i.e.*, ΔH_{PA}; thermodynamics) for the S_N2 reaction of $Nu:^{-} + C_2H_5Cl$, computed at (a) ZORA-M06-2X/QZ4P//ZORA-OLYP/QZ4P; (b) ZORA-M06-2X-D3/QZ4P//ZORA-OLYP/QZ4P. The normal nucleophiles (*i.e.*, R–Y:$^{-}$) are indicated in black. The α-nucleophiles (*i.e.*, R–X–Y:$^{-}$) have three distinct classes: class I, exhibiting α-effect, teal dots; class II, having a minor degree or no α-effect, grey dots; class III, showing inverse α-effect, red dots, where X, Y = O, HN, S and R = H, CH_3. The linear trend line (black dotted line; R^2 = [a] 0.92; [b] 0.92) is fitted to the normal nucleophile data set.

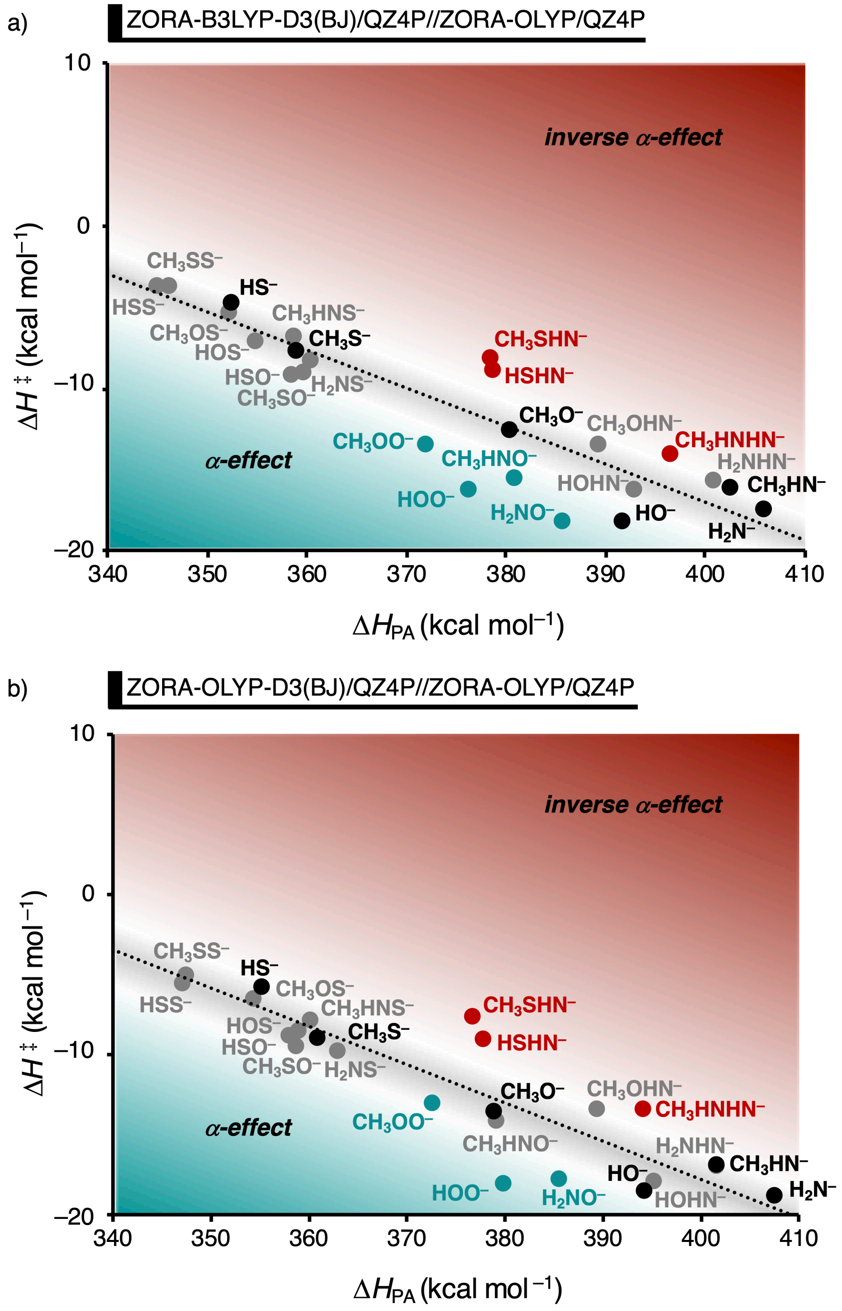

Figure S6. Brønsted-type correlation between the reaction barrier (*i.e.*, $\Delta H^{\ddagger}$; kinetics) and the basicity (*i.e.*, ΔH_{PA}; thermodynamics) for the S_N2 reaction of Nu:$^-$ + C_2H_5Cl, computed at (a) ZORA-B3LYP-D3(BJ)/QZ4P//ZORA-OLYP/QZ4P; (b) ZORA-OLYP-D3(BJ)/QZ4P//ZORA-OLYP/QZ4P. The normal nucleophiles (*i.e.*, R–Y:$^-$) are indicated in black. The α-nucleophiles (*i.e.*, R–X–Y:$^-$) have three distinct classes: class I, exhibiting α-effect, teal dots; class II, having a minor degree or no α-effect, grey dots; class III, showing inverse α-effect, red dots, where X, Y = O, HN, S and R = H, CH_3. The linear trend line (black dotted line; R^2 = [a] 0.91; [b] 0.93) is fitted to the normal nucleophile data set.

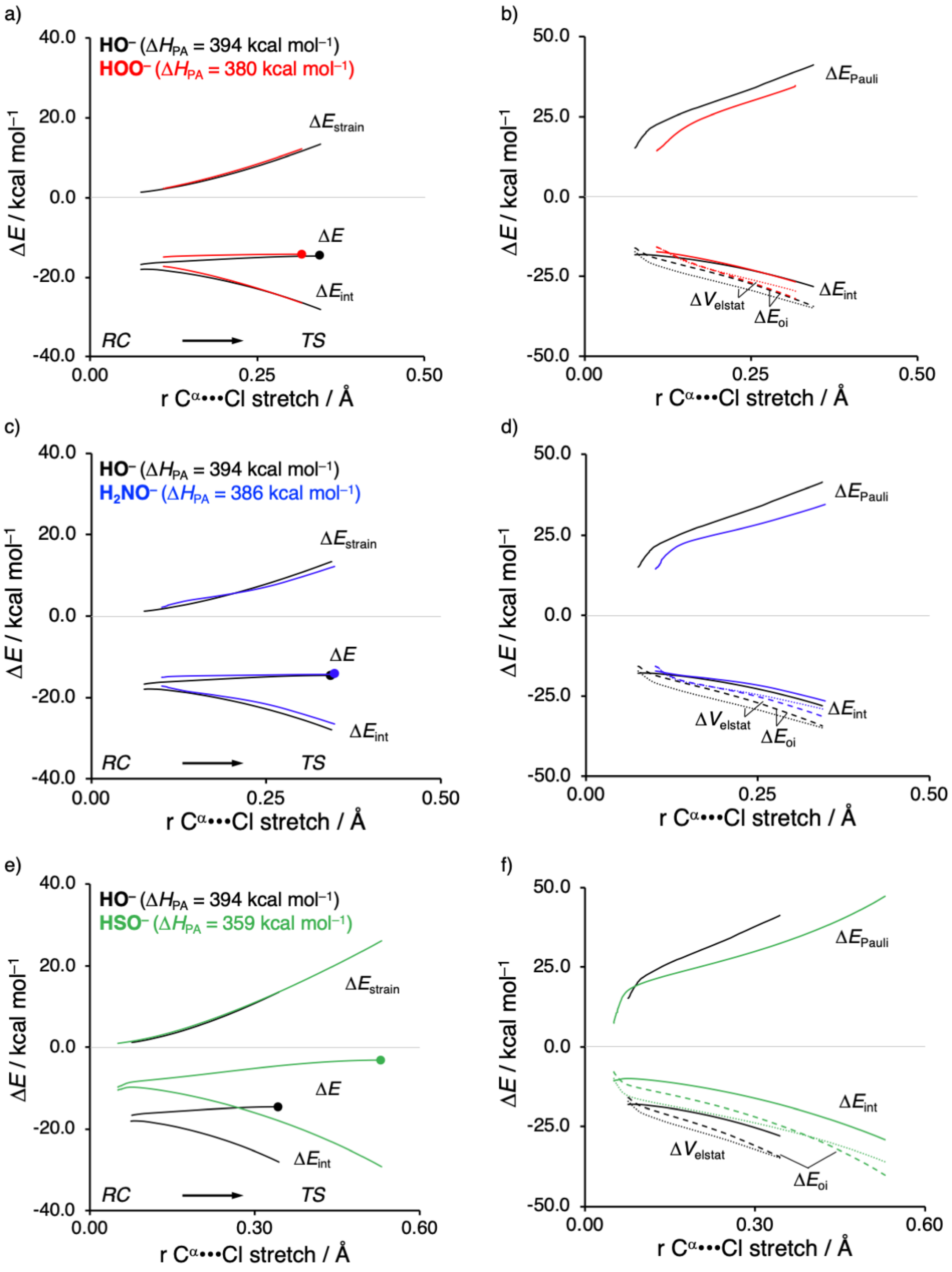

Figure S7. (a,c,e) Activation strain analysis; and (b,d,f) energy decomposition analysis of the S_N2 reactions between the normal nucleophile (HO^-, black) and α-nucleophile (HOO^-, red; H_2NO^-, blue; HSO^-, green) + C_2H_5Cl, along the IRC projected on the C^{α}•••Cl bond stretch. Computed at ZORA-OLYP/QZ4P.

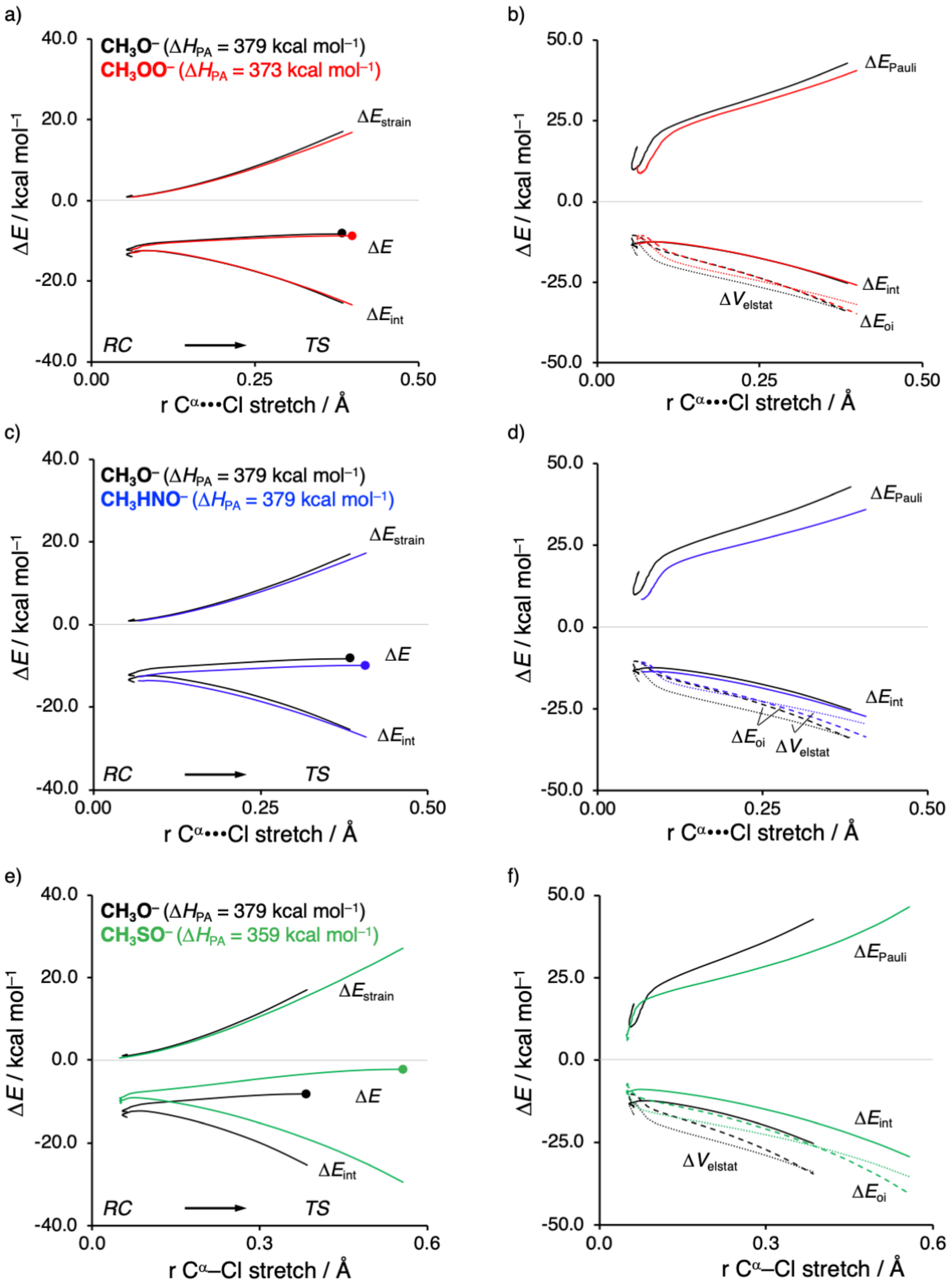

Figure S8. (a,c,e) Activation strain analysis; and (b,d,f) energy decomposition analysis of the S_N2 reactions between the normal nucleophile (CH_3O^-, black) and α-nucleophile (CH_3OO^-, red; CH_3HNO^-, blue; CH_3SO^-, green) + C_2H_5Cl, along the IRC projected on the C^{α}•••Cl bond stretch. Computed at ZORA-OLYP/QZ4P.

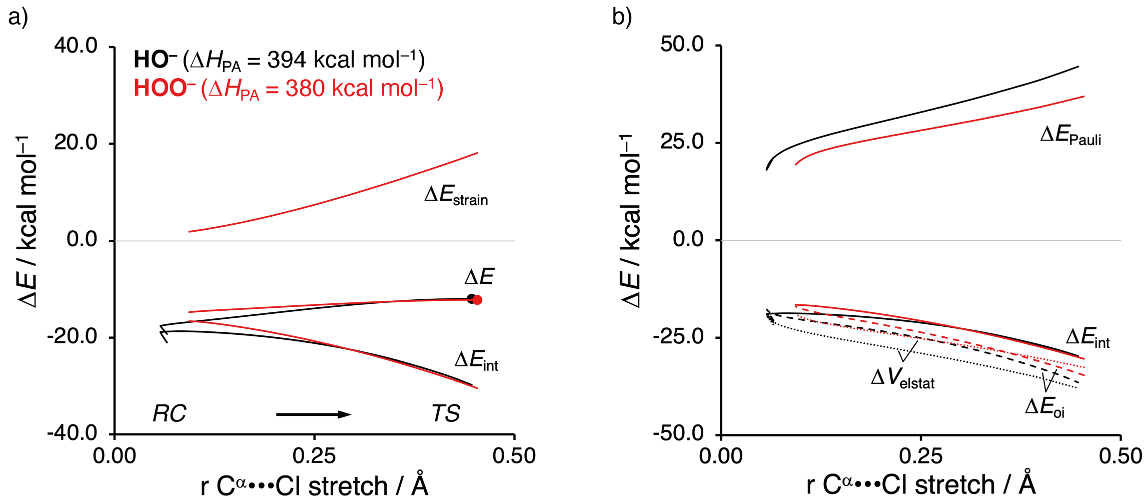

Figure S9. a) Activation strain analysis; and b) energy decomposition analysis of the S_N2 reactions between HO^- (black; normal nucleophile) and HOO^- (red; α-nucleophile) + C_3H_7Cl, along the IRC projected on the C^{α}•••Cl bond stretch. Computed at ZORA-OLYP/QZ4P.

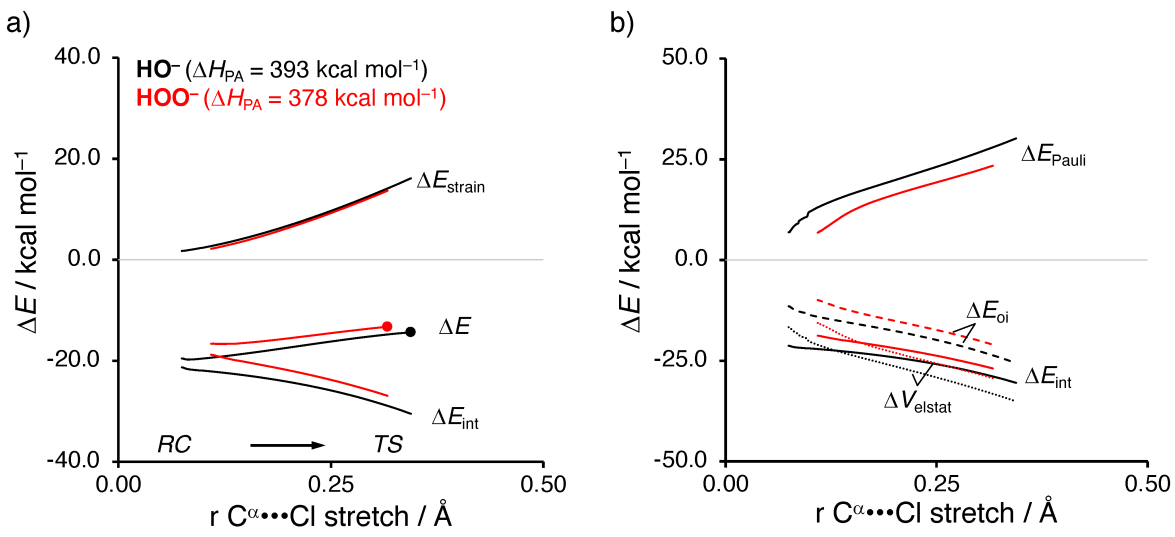

Figure S10. a) Activation strain analysis; and b) energy decomposition analysis of the S_N2 reactions between HO^- (black; normal nucleophile) and HOO^- (red; α-nucleophile) + C_2H_5Cl, along the IRC projected on the C$^{\alpha}$•••Cl bond stretch. Computed at ZORA-M06-2X/QZ4P//ZORA-OLYP/QZ4P.

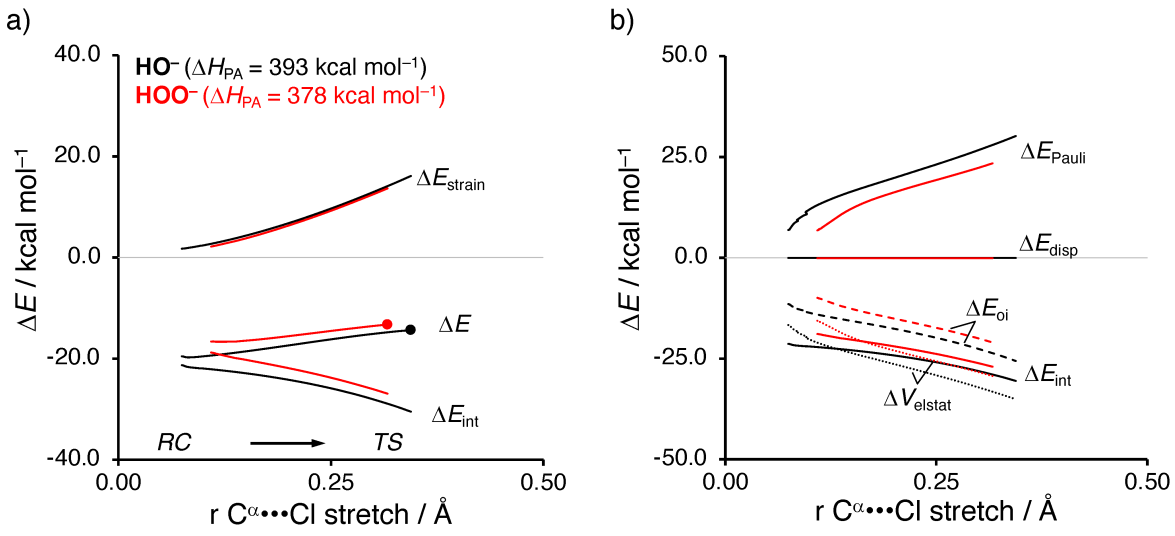

Figure S11. a) Activation strain analysis; and b) energy decomposition analysis of the S_N2 reactions between HO^- (black; normal nucleophile) and HOO^- (red; α-nucleophile) + C_2H_5Cl, along the IRC projected on the C$^{\alpha}$•••Cl bond stretch. Computed at ZORA-M06-2X-D3/QZ4P//ZORA-OLYP/QZ4P.

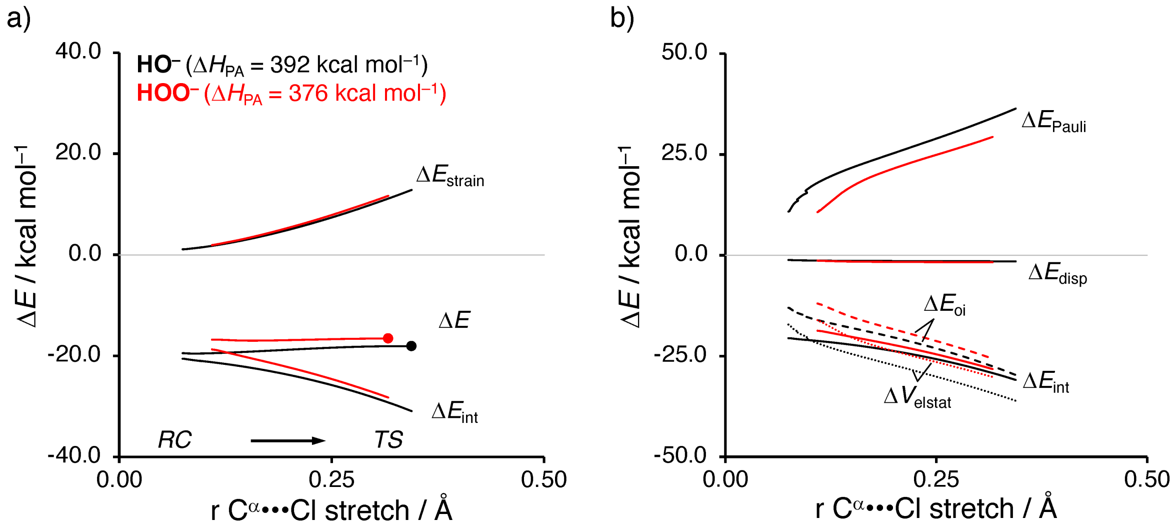

Figure S12. a) Activation strain analysis; and b) energy decomposition analysis of the S_N2 reactions between HO^- (black; normal nucleophile) and HOO^- (red; α-nucleophile) + C_2H_5Cl, along the IRC projected on the C^{α}•••Cl bond stretch. Computed at ZORA-B3LYP-D3(BJ)/QZ4P//ZORA-OLYP/QZ4P.

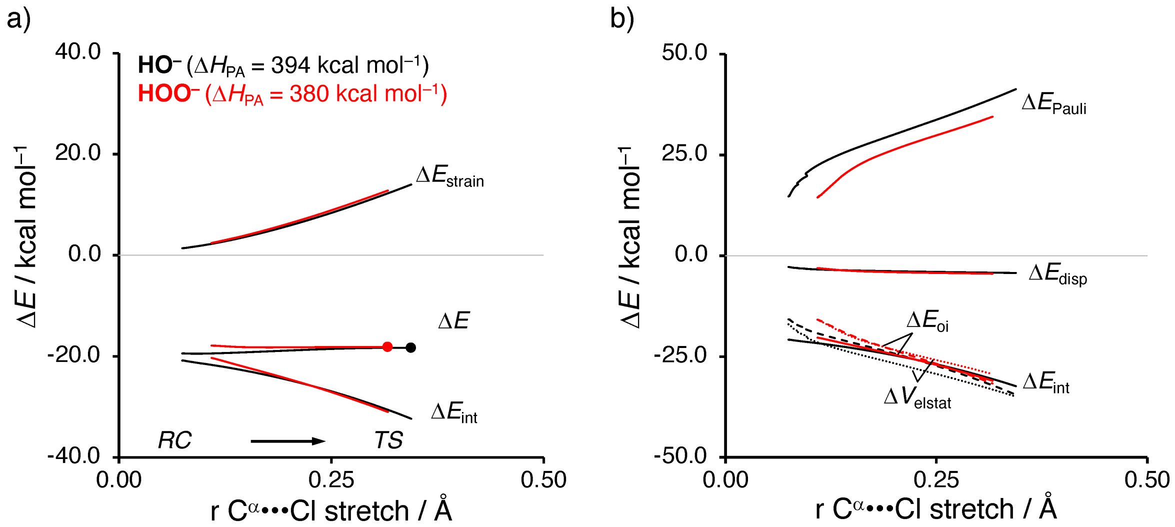

Figure S13. a) Activation strain analysis; and b) energy decomposition analysis of the S_N2 reactions between HO^- (black; normal nucleophile) and HOO^- (red; α-nucleophile) + C_2H_5Cl, along the IRC projected on the C^{α}•••Cl bond stretch. Computed at ZORA-OLYP-D3(BJ)/QZ4P//ZORA-OLYP/QZ4P.

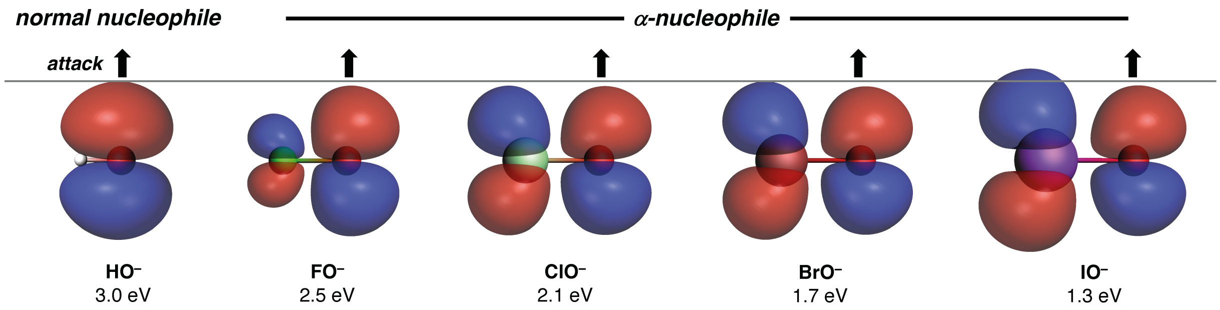

Figure S14. Key occupied orbitals ($HOMO_{Nu:^-}$; energies in eV; isovalue = 0.030 $Bohr^{-3/2}$) for FO^-, ClO^-, BrO^-,and IO^- computed at equilibrium geometries, where the gray horizontal line indicates the maximum spatial extent of the $HOMO_{HO^-}$. Computed at ZORA-OLYP/QZ4P.

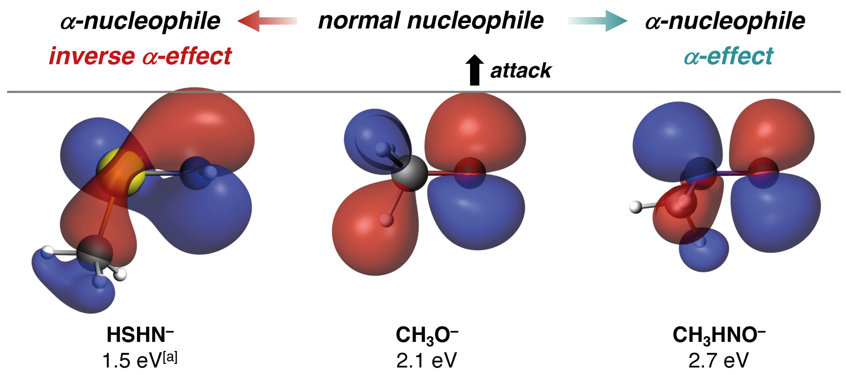

Figure S15. Key occupied orbitals ($HOMO_{Nu:^-}$; energies in eV; isovalue = 0.030 $Bohr^{-3/2}$) for $HSHN^-$, CH_3O^-, and CH_3HNO^- computed at equilibrium geometries, where the gray horizontal line indicates the maximum spatial extent of the $HOMO_{H_3CO^-}$. Computed at ZORA-OLYP/QZ4P. [a] HOMO–1 is the key occupied orbital.

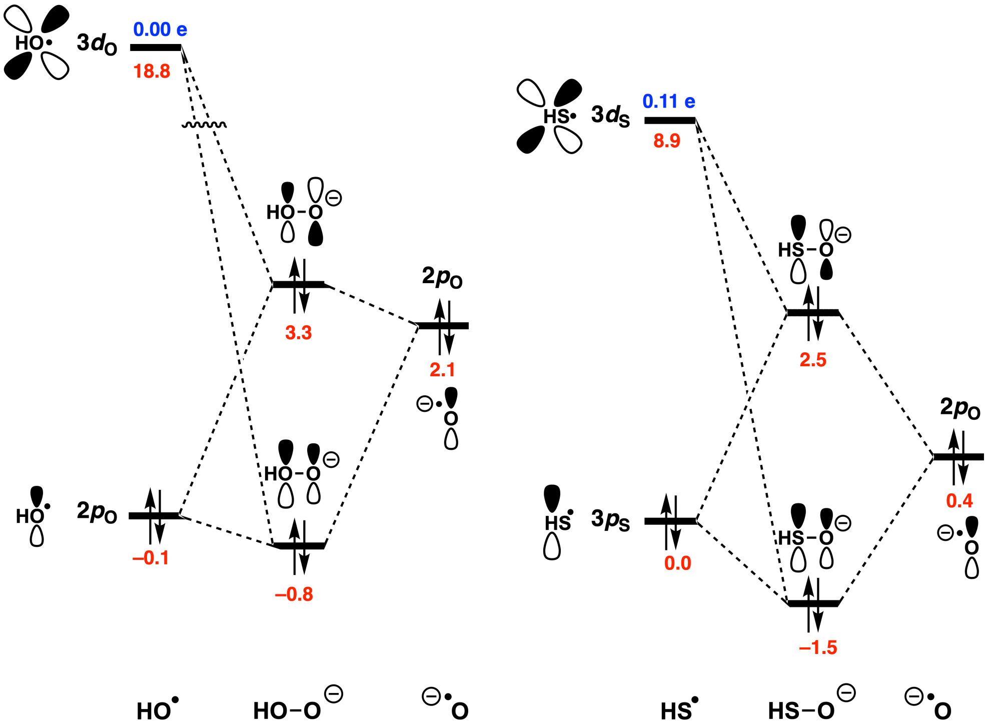

HXO⁻	ε_{HOMO,HXO^-}	2p_O-coef	⟨2p_O | np_X⟩	⟨2p_O | 3d_X⟩
HOO⁻	3.3	0.97	0.18	0.36
HSO⁻	2.5	0.79	0.26	0.44

Figure S16. The construction of the $HOMO_{HOO^-}$ (left) and $HOMO_{HSO^-}$ (right) from the interaction between the filled 2*p* atomic orbital of the nucleophilic oxygen center (2p_O) and the filled n*p* and empty 3*d* atomic orbitals of the adjacent oxygen and sulfur atom (np_X and 3d_X), where the orbital energies shown in red are computed in the presence of the other fragment (in eV) and the population of the empty 3d_X atomic orbitals are shown in blue (in electrons). Computed at ZORA-OLYP/QZ4P.

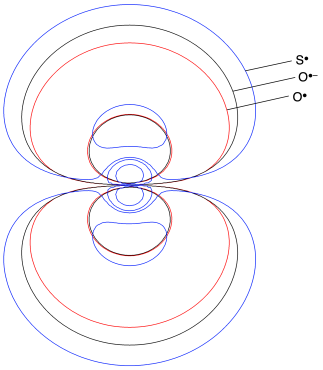

Figure S17. Contour plot of the atomic orbitals of $2p_O$ of the nucleophilic oxygen center (black lines), the $2p_O$ (red lines) and the $3p_S$ (blue lines). All contour plots contain 2 contours from 0.0195–0.2000 au.

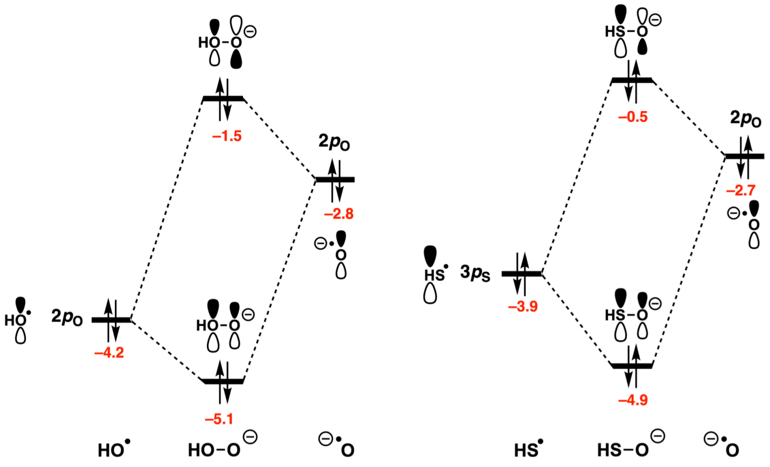

HXO⁻	ε_{HOMO,HXO^-}	$2p_O$-coef	$\langle 2p_O \mid np_X \rangle$
HOO⁻	–1.5	0.89	0.17
HSO⁻	–0.5	0.90	0.26

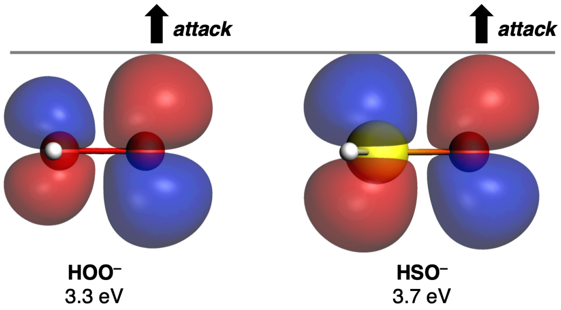

Figure S18. The construction of the $HOMO_{HOO^-}$ (left) and $HOMO_{HSO^-}$ (right) from the interaction between the filled 2*p* atomic orbital of the nucleophilic oxygen center ($2p_O$) and the filled np_X, where the orbital energies shown in red are computed in the presence of the other fragment are (in eV) and the empty orbitals are artificially removed. Computed at ZORA-OLYP/QZ4P.

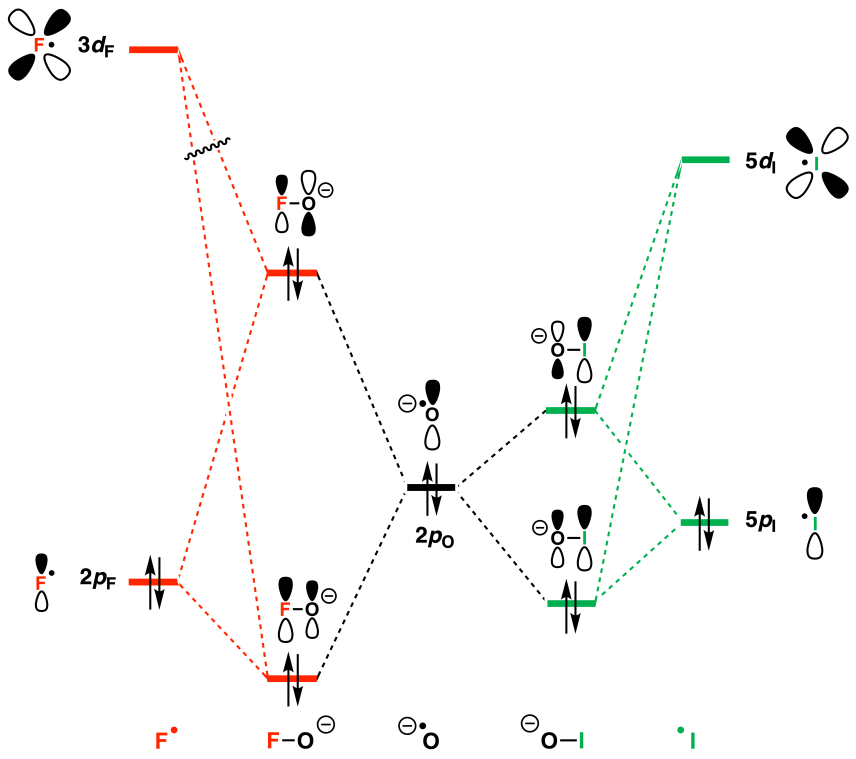

Figure S19. Schematic representation of the construction of the $HOMO_{FO-}$ (left, red) and $HOMO_{IO-}$ (right, green) from the interaction between the filled 2*p* atomic orbital of the nucleophilic oxygen center (middle, black, $2p_O$) and the filled n*p* and empty n*d* atomic orbitals of the adjacent fluorine and iodine atom (np_X and nd_X).

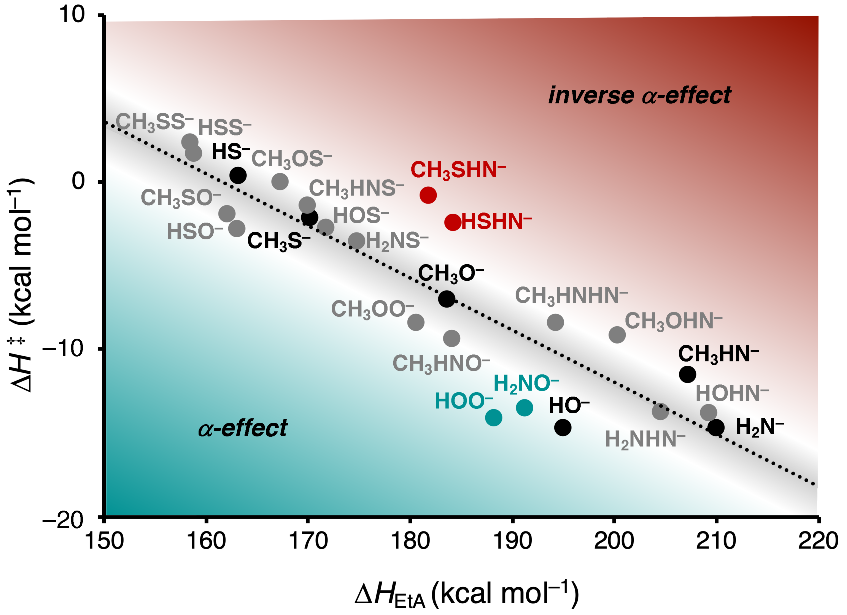

Figure S20. Brønsted-type correlation between the reaction barrier (*i.e.*, $\Delta H^{\ddagger}$; kinetics) and the carbon basicity (*i.e.*, ΔH_{EtA}; ethyl cation affinity; thermodynamics) for the S_N2 reaction of (a) Nu:$^-$ + C_2H_5Cl. The normal nucleophiles (*i.e.*, R–Y:$^-$) are indicated in black. The α-nucleophiles (*i.e.*, R–X–Y:$^-$) have three distinct classes: class I, exhibiting α-effect, teal dots; class II, having a minor degree or no α-effect, grey dots; class III, showing inverse α-effect, red dots, where X, Y = O, HN, S and R = H, CH_3. The linear trend line (black dotted line; R^2 = 0.90) is fitted to the normal nucleophile data set. Computed at ZORA-OLYP/QZ4P.

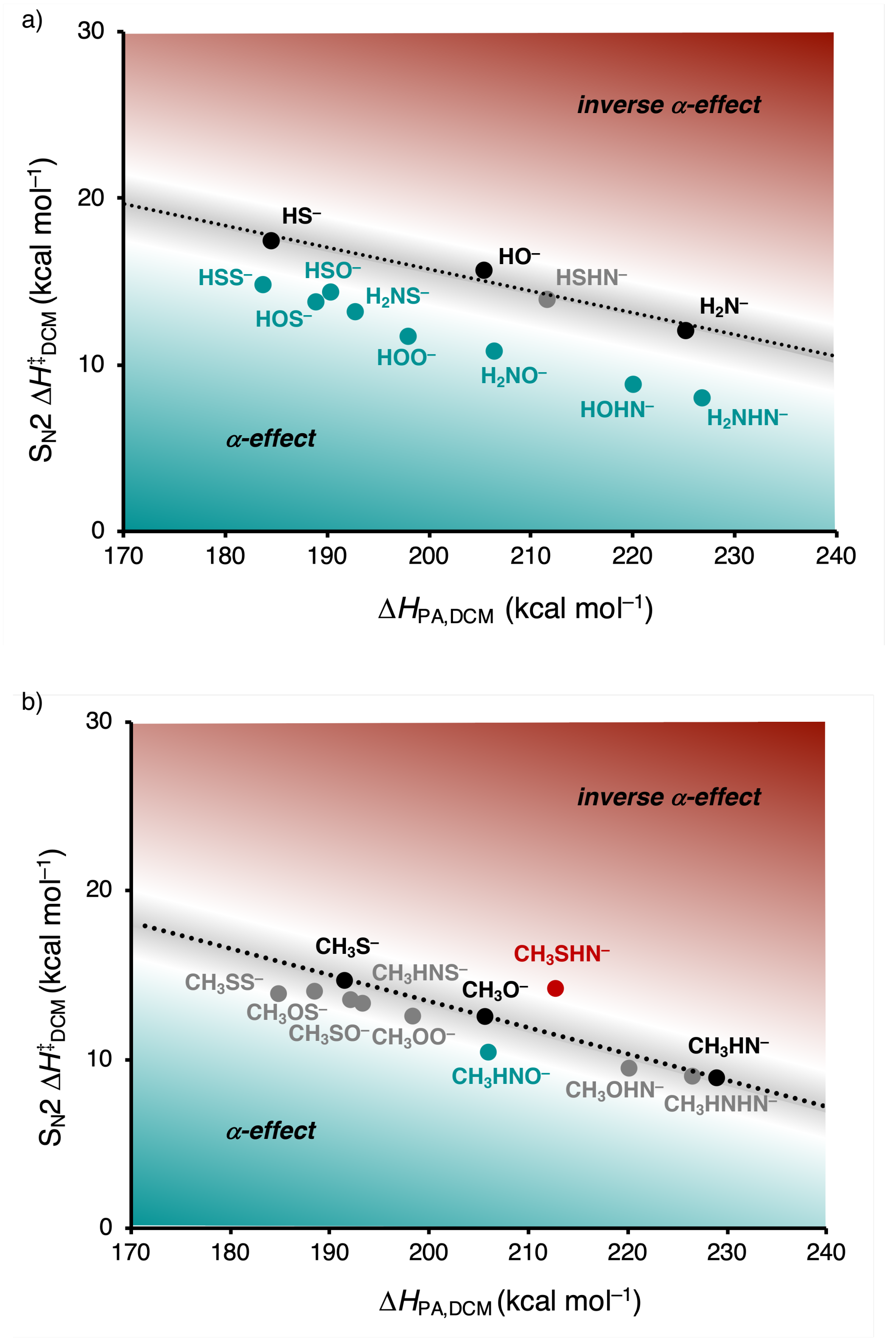

Figure S21. Brønsted-type correlation between the reaction barrier (*i.e.*, $\Delta H^{\ddagger}$; kinetics) and the carbon basicity (*i.e.*, ΔH_{PA}; proton affinity; thermodynamics) for the S_N2 reaction of (a) $Nu:^{-}$ + C_2H_5Cl. The normal nucleophiles (*i.e.*, R–Y:$^{-}$) are indicated in black (R = H, [a]; R = CH_3, [b]). The α-nucleophiles (*i.e.*, R–X–Y:$^{-}$) have three distinct classes: class I, exhibiting α-effect, teal dots; class II, having a minor degree or no α-effect, grey dots; class III, showing inverse α-effect, red dots, where X, Y = O, HN, S and R = H, CH_3. The linear trend line (black dotted line; R^2 = [a] 0.96; [b] 0.99) is fitted to the normal nucleophile data set. Computed at COSMO(DCM)ZORA-OLYP/QZ4P.

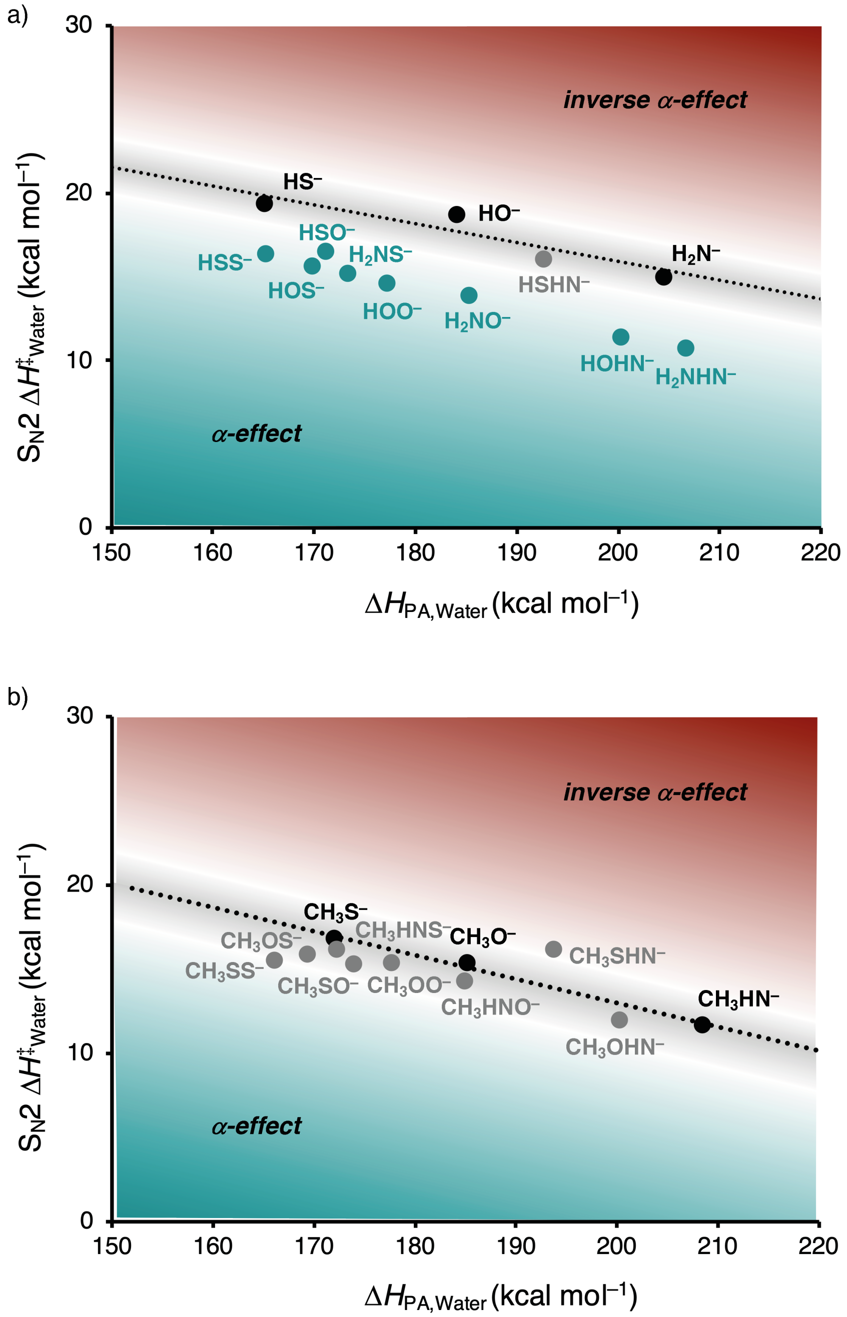

Figure S22. Brønsted-type correlation between the reaction barrier (*i.e.*, $\Delta H^{\ddagger}$; kinetics) and the carbon basicity (*i.e.*, ΔH_{PA}; proton affinity; thermodynamics) for the S_N2 reaction of (a) $Nu:^{-}$ + C_2H_5Cl. The normal nucleophiles (*i.e.*, R–Y:$^{-}$) are indicated in black (R = H, [a]; R = CH_3, [b]). The α-nucleophiles (*i.e.*, R–X–Y:$^{-}$) have three distinct classes: class I, exhibiting α-effect, teal dots; class II, having a minor degree or no α-effect, grey dots; class III, showing inverse α-effect, red dots, where X, Y = O, HN, S and R = H, CH_3. The linear trend line (black dotted line; R^2 = [a] 0.90; [b] 0.99) is fitted to the normal nucleophile data set. Computed at COSMO(Water)ZORA-OLYP/QZ4P.

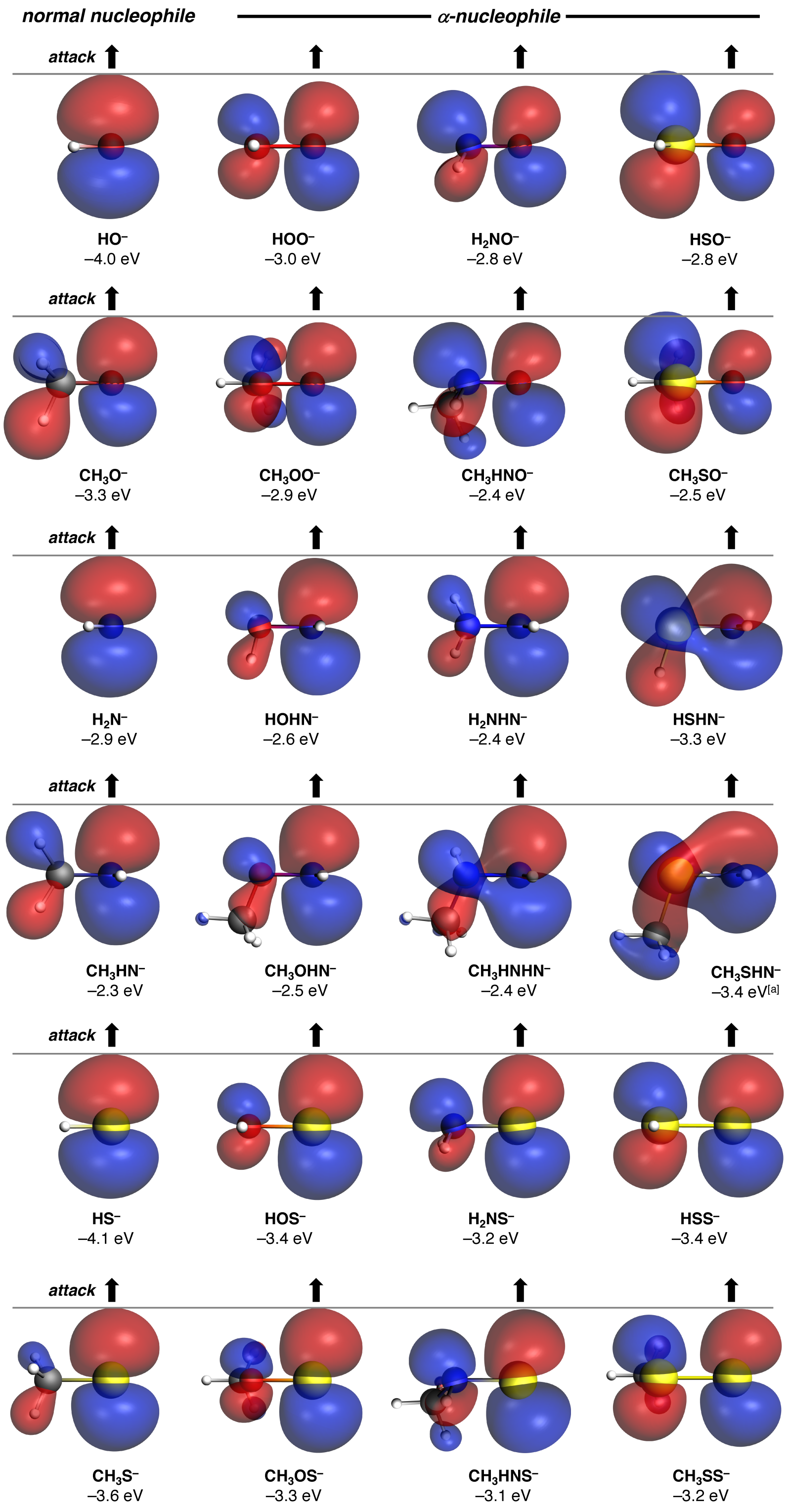

Figure S23. Key occupied orbitals ($HOMO_{Nu:^-}$; energies in eV; isovalue = 0.030 Bohr$^{-3/2}$ for *O*- and *N*-nucleophiles and isovalue = 0.035 Bohr$^{-3/2}$ for *S*-nucleophiles) computed at equilibrium geometries. Computed at COSMO(DCM)-ZORA-OLYP/QZ4P. [a] HOMO–1 is the key occupied orbital.

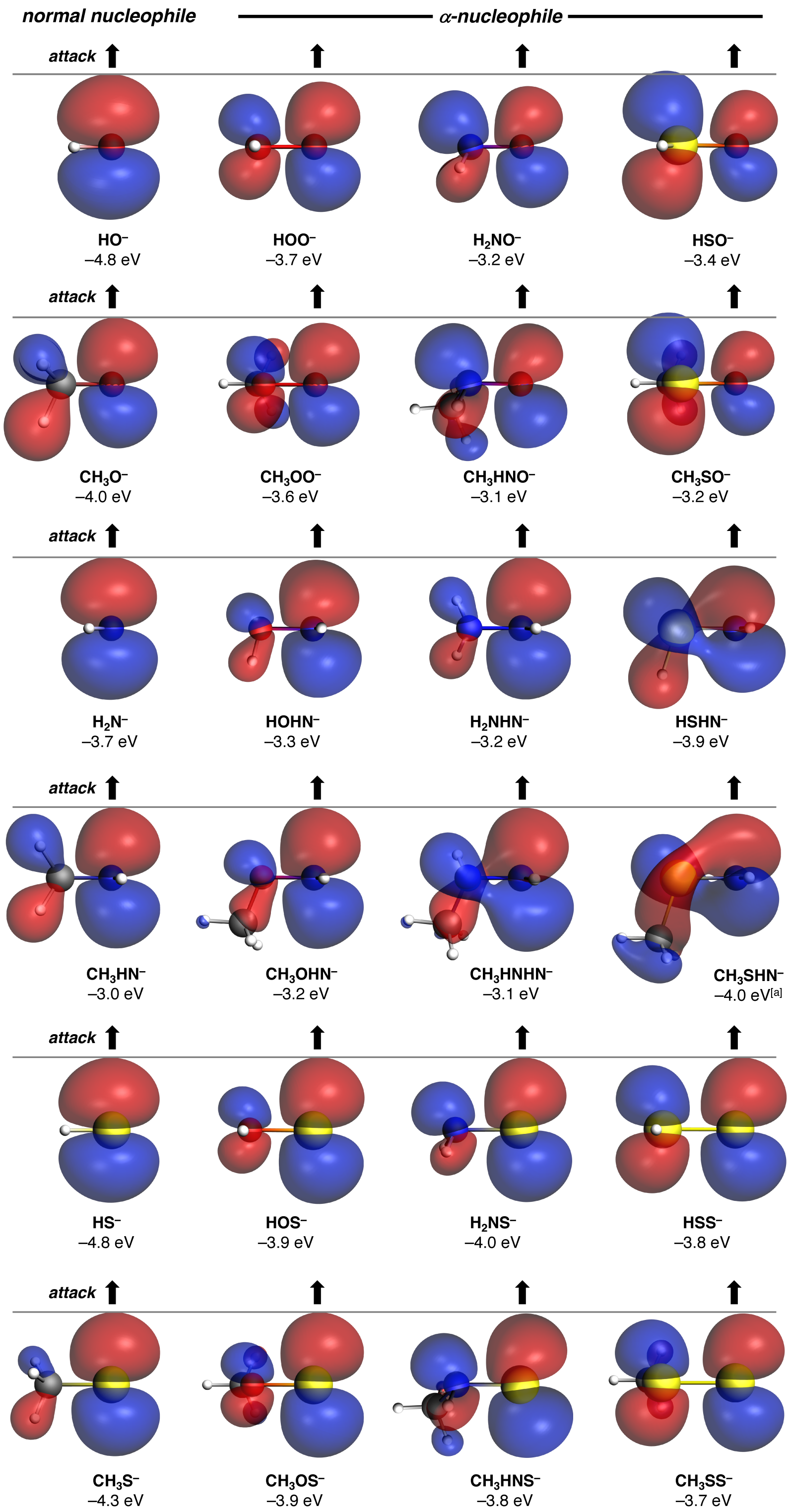

Figure S24. Key occupied orbitals ($HOMO_{Nu:^-}$; energies in eV; isovalue = 0.030 Bohr$^{-3/2}$ for *O*- and *N*-nucleophiles and isovalue = 0.035 Bohr$^{-3/2}$ for *S*-nucleophiles) computed at equilibrium geometries. Computed at COSMO(Water)-ZORA-OLYP/QZ4P. [a] HOMO–1 is the key occupied orbital.

Comparison between CH_3O^- and HOO^-

The direct comparison between CH_3O^- and HOO^- has an intrinsic complicating factor since they belong to different "chemical families". We found that the introduction of the methyl group to HO^-, *i.e.*, going from HO^- to CH_3O^-, already induces a significant change in the electronic structure of the corresponding nucleophile (SI Figure S25). The lobe of the filled orbital on the nucleophilic center of CH_3O^- is significantly smaller than the analogous lobe of HO^-, because the e-symmetric orbital of the CH_3 group mixes with the $2p_O$ atomic orbital of the nucleophilic oxygen center. This results in some density shift towards the CH_3 group, both inductively and by the accepting C–H antibonding orbitals, hence reducing the orbital lobe on the nucleophilic oxygen center of CH_3O^- compared to the analogous lobe on the nucleophilic center of HO^-. However, at the same time, the HOMO energy also decreases (*i.e.*, stabilizes) significantly, which will ultimately render no deviation from the classical Brønsted-type correlation, making CH_3O^- a pseudo α-nucleophile that does not exhibit α-effect with respect to the normal parent nucleophile HO^-. Thus, if one would compare the nucleophiles CH_3O^- and HOO^-, one is actually comparing two α-nucleophiles of the series H–Y–O^-, where Y = CH_2 or O, both belonging to the normal parent nucleophile HO. The authors, therefore, advise caution by comparing nucleophiles from different families because going from HO^- to CH_3O^- is less trivial than one would expect based on standard organic chemistry principles.

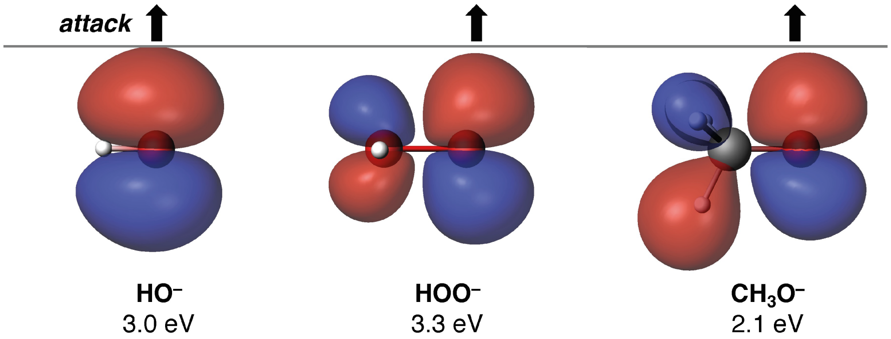

Figure S25. Representation of the DFT $HOMO_{Nu:-}$ (isovalue = 0.03 $Bohr^{-3/2}$) of HO^-, HOO^-, and CH_3O^-, where the grey horizontal line indicates the maximum spatial extent of the $HOMO_{HO-}$.

Table S1. Electronic energies relative to reactants (in kcal mol^{-1}) of the stationary points occurring in S_N2 reaction between Nu:$^-$ + C_2H_5Cl.[a]

Nu:$^-$	RC	TS	PC	P
HO^-	–17.2	–14.6	–59.0	–53.0
HOO^-	–16.7	–14.3	–53.0	–46.0
H_2NO^-	–14.9	–14.6	–54.5	–50.6
HSO^-	–10.3	–3.5	–28.6	–20.2
CH_3O^-	–12.6	–8.3	–47.4	–44.4
CH_3OO^-	–12.2	–8.8	–45.7	–39.1
CH_3HNO^-	–12.9	–10.0	–47.2	–30.4
CH_3SO^-	–9.9	–2.3	–29.6	–20.7
H_2N^-	–15.9	–15.0	–78.1	–70.4
$HOHN^-$	–15.1	–14.6	–81.3	–64.4
H_2NHN^-	–14.8	–14.4	–76.7	–70.3
$HSHN^-$	–10.0	–2.8	–53.8	–42.8
CH_3HN^-	–12.9	–12.5	–76.4	–68.7
CH_3OHN^-	–11.8	–9.7	–68.6	–54.5
CH_3HNHN^-	–10.0	–8.4	–68.9	–59.6
CH_3SHN^-	–9.2	–1.1	–51.3	–40.3
HS^-	–8.7	0.7	–27.3	–20.8
HOS^-	–9.2	–2.6	–36.9	–29.2
H_2NS^-	–9.7	–3.4	–44.9	–32.4
HSS^-	–7.9	1.7	–24.8	–16.2
CH_3S^-	–8.7	–1.6	–35.6	–28.2
CH_3OS^-	–8.2	0.0	–32.5	–25.0
CH_3HNS^-	–8.7	–1.4	–40.2	–27.7
CH_3SS^-	–7.6	2.5	–23.7	–27.7

[a] Computed at ZORA-OLYP/QZ4P.

Table S2. Computed proton affinities (ΔH_{PA}) and S_N2 reaction barrier enthalpies ($\Delta H^{‡}$) for $Nu:^-$ + C_2H_5Cl.[a]

$Nu:^-$	ΔH_{PA}	$\Delta H^{‡}$
HO^-	394.2	–14.7
HOO^-	379.8	–14.1
H_2NO^-	385.5	–13.5
HSO^-	358.6	–3.2
CH_3O^-	378.8	–7.0
CH_3OO^-	372.6	–8.4
CH_3HNO^-	379.1	–9.4
CH_3SO^-	358.9	–1.9
H_2N^-	407.5	–14.7
$HOHN^-$	395.2	–13.8
H_2NHN^-	401.5	–13.7
$HSHN^-$	377.8	–2.4
CH_3HN^-	401.5	–11.5
CH_3OHN^-	389.3	–9.1
CH_3HNHN^-	394.1	–8.4
CH_3SHN^-	376.6	–0.8
HS^-	355.2	0.4
HOS^-	357.9	–2.7
H_2NS^-	362.9	–3.5
HSS^-	347.1	1.7
CH_3S^-	360.8	–2.1
CH_3OS^-	354.3	0.0
CH_3HNS^-	360.1	–1.4
CH_3SS^-	347.4	2.4

[a] Computed at ZORA-OLYP/QZ4P.

Table S3. Computed proton affinities (ΔH_{PA}) and S_N2 reaction barrier enthalpies ($\Delta H^{\ddagger}$) Nu:$^-$ + $C_nH_{2n+1}Y$, in which n = 1, 2, 3 and Y = F, Cl.[a]

Nu:$^-$	ΔH_{PA}	$\Delta H^{\ddagger}$ (CH_3F)	$\Delta H^{\ddagger}$ (CH_3Cl)	$\Delta H^{\ddagger}$ (C_2H_5F)	$\Delta H^{\ddagger}$ (C_2H_5Cl)	$\Delta H^{\ddagger}$ (C_3H_7F)	$\Delta H^{\ddagger}$ (C_3H_7Cl)
HO^-	394.2	–6.1	–16.4	–2.5	–14.7	1.4	–12.2
HOO^-	379.8	–5.8	–15.0	–2.4	–14.1	1.7	–12.2
H_2NO^-	385.5	–4.3	–14.7	–0.8	–13.5	2.8	–11.6
HSO^-	358.6	7.4	–5.2	11.3	–3.2	15.4	–1.2
CH_3O^-	378.8	1.1	–9.2	5.2	–7.0	9.8	–4.2
CH_3OO^-	372.6	0.8	–10.0	5.3	–8.4	8.4	–6.2
CH_3HNO^-	379.1	0.2	–10.8	3.8	–9.4	7.5	–8.0
CH_3SO^-	358.9	9.3	–3.8	13.0	–1.9	16.4	–0.2
H_2N^-	407.5	–7.3	–15.4	–2.7	–14.7	1.1	–12.7
$HOHN^-$	395.2	–7.8	[b]	–3.5	–13.8	0.8	–13.0
H_2NHN^-	401.5	–8.2	–13.9	–3.9	–13.7	0.4	–10.9
$HSHN^-$	377.8	4.8	–5.7	10.1	–2.4	14.7	0.1
CH_3HN^-	401.5	–5.1	[b]	–0.2	–11.5	4.3	–9.2
CH_3OHN^-	389.3	–2.1	[b]	2.5	–9.1	7.0	–6.8
CH_3HNHN^-	394.1	–1.8	–10.2	2.7	–8.4	8.6	–5.7
CH_3SHN^-	376.6	6.6	–4.1	11.9	–0.8	16.5	1.3
HS^-	355.2	9.7	–4.0	15.0	0.4	20.0	4.1
HOS^-	357.9	6.0	–6.4	11.0	–2.7	16.0	0.7
H_2NS^-	362.9	5.4	–7.1	10.2	–3.5	14.6	–0.8
HSS^-	347.1	11.0	–2.8	16.2	1.7	21.1	5.2
CH_3S^-	360.8	6.9	–5.5	12.1	–2.1	17.8	2.3
CH_3OS^-	354.3	9.1	–3.7	14.2	0.0	19.4	2.8
CH_3HNS^-	360.1	7.2	–5.0	12.9	–1.4	17.8	1.7
CH_3SS^-	347.4	12.0	–1.4	17.2	2.4	22.0	5.1

[a] Computed at ZORA-OLYP/QZ4P. [b] Nonexistent: encounter of reactants induces S_N2 reaction without barrier.

Table S4. Computed proton affinities (ΔH_{PA}) and S_N2 reaction barrier enthalpies ($\Delta H^{‡}$) for $Nu:^-$ + C_2H_5Cl at ZORA-M06-2X/QZ4P//ZORA-OLYP/QZ4P.

$Nu:^-$	ΔH_{PA}	$\Delta H^{‡}$
HO^-	393.1	–14.5
HOO^-	377.6	–13.1
H_2NO^-	386.4	–13.9
HSO^-	359.0	–3.6
CH_3O^-	382.1	–9.6
CH_3OO^-	372.5	–9.0
CH_3HNO^-	382.0	–11.1
CH_3SO^-	359.9	–2.4
H_2N^-	406.0	–13.3
$HOHN^-$	394.1	–14.3
H_2NHN^-	400.6	–13.9
$HSHN^-$	378.6	–4.1
CH_3HN^-	402.7	–12.2
CH_3OHN^-	390.2	–9.3
CH_3HNHN^-	397.6	–11.2
CH_3SHN^-	378.3	–3.2
HS^-	350.7	1.2
HOS^-	353.3	–1.3
H_2NS^-	358.4	–2.4
HSS^-	342.3	3.0
CH_3S^-	356.5	–1.6
CH_3OS^-	350.2	0.8
CH_3HNS^-	356.4	–0.6
CH_3SS^-	343.1	3.5

Table S5. Computed proton affinities (ΔH_{PA}) and S_N2 reaction barrier enthalpies ($\Delta H^{\ddagger}$) for Nu:$^-$ + C_2H_5Cl at ZORA-M06-2X-D3/QZ4P//ZORA-OLYP/QZ4P.

Nu:$^-$	ΔH_{PA}	$\Delta H^{\ddagger}$
HO^-	391.1	–14.5
HOO^-	377.6	–13.2
H_2NO^-	386.4	–14.1
HSO^-	359.0	–3.7
CH_3O^-	382.1	–9.7
CH_3OO^-	372.5	–9.2
CH_3HNO^-	382.1	–11.3
CH_3SO^-	359.9	–2.6
H_2N^-	406.0	–13.3
$HOHN^-$	394.1	–14.5
H_2NHN^-	400.6	–14.1
$HSHN^-$	378.6	–4.2
CH_3HN^-	402.7	–12.3
CH_3OHN^-	390.2	–9.5
CH_3HNHN^-	397.6	–11.4
CH_3SHN^-	378.3	–3.4
HS^-	350.7	1.1
HOS^-	353.3	–1.4
H_2NS^-	358.4	–2.5
HSS^-	342.3	2.9
CH_3S^-	356.6	–1.7
CH_3OS^-	350.2	0.6
CH_3HNS^-	356.4	–0.7
CH_3SS^-	343.1	3.3

Table S6. Computed proton affinities (ΔH_{PA}) and S_N2 reaction barrier enthalpies ($\Delta H^{\ddagger}$) for Nu:⁻ + C_2H_5Cl at ZORA-B3LYP-D3(BJ)/QZ4P//ZORA-OLYP/QZ4P.

Nu:⁻	ΔH_{PA}	$\Delta H^{\ddagger}$
HO^-	391.7	–18.2
HOO^-	376.3	–16.4
H_2NO^-	385.7	–18.1
HSO^-	358.5	–9.6
CH_3O^-	380.4	–12.6
CH_3OO^-	371.9	–13.5
CH_3HNO^-	380.9	–15.6
CH_3SO^-	359.6	–9.0
H_2N^-	405.9	–17.5
$HOHN^-$	393.1	–16.3
H_2NHN^-	400.8	–15.7
$HSHN^-$	378.6	–8.9
CH_3HN^-	402.6	–16.1
CH_3OHN^-	389.4	–13.4
CH_3HNHN^-	396.5	–14.1
CH_3SHN^-	378.3	–8.1
HS^-	352.5	–4.8
HOS^-	354.8	7.1
H_2NS^-	360.1	–8.3
HSS^-	344.9	–3.8
CH_3S^-	359.0	–7.7
CH_3OS^-	352.3	–5.4
CH_3HNS^-	358.6	–6.8
CH_3SS^-	346.0	–3.7

Table S7. Computed proton affinities (ΔH_{PA}) and S_N2 reaction barrier enthalpies ($\Delta H^{\ddagger}$) for Nu:$^-$ + C_2H_5Cl at ZORA-OLYP-D3(BJ)/QZ4P//ZORA-OLYP/QZ4P.

Nu:$^-$	ΔH_{PA}	$\Delta H^{\ddagger}$
HO^-	395.8	–18.4
HOO^-	381.7	–18.0
H_2NO^-	387.6	–17.7
HSO^-	361.1	–9.4
CH_3O^-	381.2	–13.5
CH_3OO^-	374.7	–13.0
CH_3HNO^-	381.3	–14.1
CH_3SO^-	361.6	–8.5
H_2N^-	409.6	–18.7
$HOHN^-$	397.6	–17.9
H_2NHN^-	404.3	–16.9
$HSHN^-$	380.7	–9.0
CH_3HN^-	404.3	–16.8
CH_3OHN^-	391.3	–13.4
CH_3HNHN^-	397.2	–13.3
CH_3SHN^-	379.2	–7.6
HS^-	357.7	–5.7
HOS^-	360.6	–8.8
H_2NS^-	365.8	–9.7
HSS^-	350.3	–5.5
CH_3S^-	363.9	–8.9
CH_3OS^-	357.2	–6.5
CH_3HNS^-	363.2	–7.8
CH_3SS^-	350.8	–5.0

Numerical experiment

To assess the potential influence of the varying Nu:⁻•••C^{α} distances (*i.e.*, distance between the nucleophile and the substrate) on the EDA results, which as we shown in previous studies can influence the EDA terms,[10a] we performed a numerical experiment where we artificially constrain the HOO^{-}•••C^{α} bond length of the reaction involving HOO^{-} to the bond length of HO^{-}•••C^{α} while keeping the C^{α}•••Cl bond stretch at 0.30 Å (see SI Tables S8 and S9). The associated EDA results are entirely consistent with the EDA diagram of Figure 3b, namely, the similar reactivity of HO^{-} and HOO^{-} can again be related to the offset between the electrostatic interaction and (steric) Pauli repulsion. Thus, the conclusion obtained from the interaction energy terms shown in Figure 3b are not skewed by the difference in Nu:⁻•••C^{α} distances.

Table S8. Activation strain and energy decomposition analyses (in kcal mol^{-1}) for the S_N2 reaction between Nu:⁻ + C_2H_5Cl.[a]

Nu:⁻	ΔH_{PA}	ΔE^*	ΔE_{strain}	ΔE_{int}	ΔV_{elstat}	ΔE_{Pauli}	ΔE_{oi}
**HO^{-}**	394.2	–14.6	10.7	–25.3	–32.2	37.7	–30.8
**HOO^{-}**	379.8	–14.3	11.0	–25.3	–28.3	33.1	–30.1
**H_2NO^{-}**	385.5	–14.6	10.4	–25.0	–26.5	31.2	–29.7
**HSO^{-}**	358.6	–5.6	10.2	–15.8	–23.6	29.4	–21.6

[a] Analyses at consistent geometries with a C^{α}•••Cl bond stretch of 0.30 Å and a Nu:⁻•••C^{α} bond distance of 2.43 Å. Computed at ZORA-OLYP/QZ4P.

Table S9. Activation strain and energy decomposition analyses (in kcal mol^{-1}) for the S_N2 reaction between Nu:⁻ + C_2H_5Cl.[a]

Nu:⁻	ΔH_{PA}	ΔE^*	ΔE_{strain}	ΔE_{int}	ΔV_{elstat}	ΔE_{Pauli}	ΔE_{oi}
**CH_3O^{-}**	378.8	–8.3	17.1	–25.4	–33.8	42.7	–34.3
**CH_3OO^{-}**	372.6	–8.8	16.3	–25.1	–32.2	41.9	–34.8
**CH_3HNO^{-}**	379.1	–9.9	16.2	–26.2	–30.4	38.5	–34.2
**CH_3SO^{-}**	358.9	–3.4	15.3	–18.8	–25.9	32.7	–25.7

[a] Analyses at consistent geometries with a C^{α}•••Cl bond stretch of 0.38 Å and a Nu:⁻•••C^{α} bond distance of 2.30 Å. Computed at ZORA-OLYP/QZ4P.

Table S10. Most important occupied–occupied orbital overlaps for the S_N2 reaction of Nu:⁻ + C_2H_5Cl between $HOMO_{Nu:–}$ and the filled orbitals on the substate.[a]

	$HOMO_{Nu:–}$						
	HOMO	HOMO–1	HOMO–2	HOMO–3	HOMO–4	HOMO–5	HOMO–6
**$HO^–$**	0.00	0.01	0.06	0.01	0.03	0.08	0.02
**$HOO^–$**	0.00	0.01	0.06	0.00	0.01	0.06	0.00
**$H_2NO^–$**	0.00	0.01	0.05	0.00	0.00	0.04	0.00
**$HSO^–$**	0.00	0.01	0.03	0.00	0.00	0.04	0.00

[a] Analyses at consistent geometries with a C^{α}•••Cl bond stretch of 0.30 Å and a Nu:⁻•••C^{α} bond distance of 2.43 Å. Computed at ZORA-OLYP/QZ4P.

Table S11. Activation strain and energy decomposition analyses (in kcal mol^{-1}) for the S_N2 reaction between Nu:⁻ + C_2H_5F.[a]

**Nu:⁻**	ΔH_{PA}	ΔE^*	ΔE_{strain}	ΔE_{int}	ΔV_{elstat}	ΔE_{Pauli}	ΔE_{oi}
**$HO^–$**	394.2	–2.1	27.2	–29.3	–57.6	80.9	–52.6
**$HOO^–$**	379.8	–2.4	28.1	–30.5	–51.7	72.1	–50.9

[a] Analyses at consistent geometries with a C^{α}•••Cl bond stretch of 0.40 Å and a Nu:⁻•••C^{α} bond distance of 2.08 Å. Computed at ZORA-OLYP/QZ4P.

Table S12. Activation strain and energy decomposition analyses (in kcal mol^{-1}) for the S_N2 reaction between RO:⁻ + C_2H_5Cl, in which R = H, F, Cl, Br, I.[a]

**Nu:⁻**	ΔH_{PA}	ΔE^*	ΔE_{strain}	ΔE_{int}	ΔV_{elstat}	ΔE_{Pauli}	ΔE_{oi}
**$HO^–$**	394.2	–14.6	10.7	–25.3	–32.2	37.7	–30.8
**$FO^–$**	365.0	–8.9	13.5	–22.5	–26.8	30.9	–26.6
**$ClO^–$**	358.3	–5.8	11.0	–16.8	–24.3	29.5	–22.0
**$BrO^–$**	359.5	–5.1	11.2	–16.3	–24.1	29.3	–21.5
**$IO^–$**	358.0	–3.6	10.5	–14.1	–23.5	29.2	–19.8

[a] Analyses at consistent geometries with a C^{α}•••Cl bond stretch of 0.30 Å and a Nu:⁻•••C^{α} bond distance of 2.43 Å. Computed at ZORA-OLYP/QZ4P.

Table S13. Voronoi deformation density (VDD) atomic charges (Q^{VDD}; in electrons) of the nucleophilic centers of the studied normal and α-nucleophiles.[a]

Nu:⁻	Q^{VDD}
HO^-	–0.814
HOO^-	–0.702
H_2NO^-	–0.660
HSO^-	–0.615
CH_3O^-	–0.637
CH_3OO^-	–0.602
CH_3HNO^-	–0.599
CH_3SO^-	–0.615
H_2N^-	–0.620
$HOHN^-$	–0.518
H_2NHN^-	–0.569
$HSHN^-$	–0.519
CH_3HN^-	–0.569
CH_3OHN^-	–0.443
CH_3HNHN^-	–0.471
CH_3SHN^-	–0.494
HS^-	–0.748
HOS^-	–0.708
H_2NS^-	–0.742
HSS^-	–0.691
CH_3S^-	–0.762
CH_3OS^-	–0.628
CH_3HNS^-	–0.656
CH_3SS^-	–0.652

[a] Computed at ZORA-OLYP/QZ4P.

Table S14. Activation strain and energy decomposition analyses (in kcal mol^{-1}) for the S_N2 reaction between Nu:⁻ + C_2H_5Cl.[a]

**Nu:⁻**	ΔH_{PA}	$\Delta E*$	ΔE_{strain}	ΔE_{int}	ΔV_{elstat}	ΔE_{Pauli}	ΔE_{oi}
**CH_3HNO^-**	379.1	–10.3	11.5	–21.8	–24.9	29.5	–26.4
**CH_3O^-**	378.8	–8.7	10.7	–20.0	–27.3	32.9	–25.6
**H_2N^-**	407.5	–14.1	10.6	–24.6	–44.8	61.1	–40.9
**$HSHN^-$**	377.8	–2.7	11.9	–14.6	–31.6	44.7	–27.8
**CH_3SHN^-**	376.6	0.1	11.0	–12.2	–31.1	45.1	–26.1

[a] Analyses at consistent geometries with a C^{α}•••Cl bond stretch of 0.30 Å and a Nu:⁻•••C^{α} bond distance of 2.43 Å. Computed at ZORA-OLYP/QZ4P.

Table S15. Activation strain and energy decomposition analyses (in kcal mol^{-1}) for the interaction between the Nu:$^{-}$ and H^{+} in Nu–H.[a]

	ΔH_{PA}	ΔE	ΔE_{strain}	ΔE_{int}	ΔV_{elstat}	ΔE_{Pauli}	ΔE_{oi}	r (O–H)
**HO–H**	394.2	–401.5	0.0	–401.5	–224.4	0.0	–177.1	0.964
**HOO–H**	379.8	–387.4	1.0	–388.4	–211.9	0.0	–176.5	0.970
**H_2NO–H**	385.5	–394.7	1.6	–396.3	–206.2	0.0	–190.1	0.964
**HSO–H**	358.6	–366.0	3.6	–369.6	–180.8	0.0	–188.8	0.967
**CH_3O–H**	378.8	–388.6	5.9	–394.5	–204.8	0.0	–189.7	0.963
**CH_3OO–H**	372.6	–380.5	0.8	–381.3	–189.2	0.0	–192.1	0.970
**CH_3HNO–H**	379.1	–387.8	1.5	–389.3	–188.9	0.0	–200.4	0.965
**CH_3SO–H**	358.9	–366.4	3.5	–369.3	–174.4	0.0	–195.5	0.967
**H_2N–H**	407.5	–416.3	0.4	–416.7	–217.4	0.0	–199.3	1.017
**HOHN–H**	395.2	–404.0	5.0	–409.1	–209.7	0.0	–199.3	1.022
**H_2NHN–H**	401.5	–411.5	3.1	–414.6	–208.2	0.0	–206.4	1.020
**HSHN–H**	377.8	–386.2	4.4	–390.6	–184.7	0.0	–205.9	1.014
**CH_3HN–H**	401.5	–411.9	3.2	–415.1	–209.1	0.0	–206.0	1.016
**CH_3OHN–H**	389.3	–397.4	2.9	–400.3	–185.6	0.0	–214.7	1.025
**CH_3HNHN–H**	394.1	–403.3	1.4	–404.7	–181.2	0.0	–223.5	1.025
**CH_3SHN–H**	376.6	–384.9	3.9	–388.8	–173.8	0.0	–215.0	1.015
**HS–H**	355.2	–360.2	0.0	–360.2	–153.8	0.0	–206.3	1.345
**HOS–H**	357.9	–363.2	3.0	–366.2	–159.7	0.0	–206.5	1.356
**H_2NS–H**	362.9	–368.5	2.5	–371.1	–160.9	0.0	–210.2	1.351
**HSS–H**	347.1	–352.3	0.5	–352.3	–142.1	0.0	–210.7	1.352
**CH_3S–H**	360.8	–366.7	0.9	–367.6	–157.8	0.0	–209.8	1.345
**CH_3OS–H**	354.3	–359.8	2.6	–362.5	–149.6	0.0	–212.9	1.358
**CH_3HNS–H**	360.1	–365.8	3.1	–368.9	–153.7	0.0	–215.2	1.367
**CH_3SS–H**	347.4	–352.7	0.2	–352.9	–137.9	0.0	–215.0	1.354

[a] Analyses at equilibrium geometries. Computed at ZORA-OLYP/QZ4P.

Table S16. Computed proton affinities (ΔH_{PA}) and S_N2 reaction barrier enthalpies ($\Delta H^{\ddagger}$) at COSMO(DCM)ZORA-OLYP/QZ4P.

$Nu:^-$	ΔH_{PA}	$\Delta H^{\ddagger}$
HO^-	205.4	15.7
HOO^-	198.0	11.7
H_2NO^-	206.4	10.8
HSO^-	190.4	14.3
CH_3O^-	205.6	12.6
CH_3OO^-	198.4	12.5
CH_3HNO^-	206.0	10.4
CH_3SO^-	192.1	13.6
H_2N^-	225.2	12.1
$HOHN^-$	220.1	8.8
H_2NHN^-	226.9	8.0
$HSHN^-$	211.7	13.9
CH_3HN^-	228.9	8.9
CH_3OHN^-	220.1	9.5
CH_3HNHN^-	226.5	9.0
CH_3SHN^-	212.8	14.2
HS^-	184.5	17.5
HOS^-	188.9	13.7
H_2NS^-	192.8	13.2
HSS^-	183.7	14.8
CH_3S^-	191.4	14.7
CH_3OS^-	188.5	14.1
CH_3HNS^-	193.2	13.4
CH_3SS^-	184.8	13.9

Table S17. Computed proton affinities (ΔH_{PA}) and S_N2 reaction barrier enthalpies ($\Delta H^{‡}$) at COSMO(Water)ZORA-OLYP/QZ4P.

$Nu:^-$	ΔH_{PA}	$\Delta H^{‡}$
HO^-	184.0	18.7
HOO^-	177.1	14.6
H_2NO^-	185.2	13.9
HSO^-	171.1	16.5
CH_3O^-	185.2	15.4
CH_3OO^-	177.7	15.4
CH_3HNO^-	185.0	14.3
CH_3SO^-	172.2	16.2
H_2N^-	204.4	15.0
$HOHN^-$	200.2	11.4
H_2NHN^-	206.6	10.7
$HSHN^-$	192.7	16.0
CH_3HN^-	208.5	11.7
CH_3OHN^-	200.3	12.0
CH_3HNHN^-	206.5	11.3
CH_3SHN^-	193.7	16.2
HS^-	165.1	19.4
HOS^-	169.8	15.6
H_2NS^-	173.3	15.2
HSS^-	165.2	16.4
CH_3S^-	172.0	16.8
CH_3OS^-	169.4	15.9
CH_3HNS^-	173.9	15.3
CH_3SS^-	166.1	15.5

Table S18. Cartesian coordinates (Å), energies (in kcal mol^{-1}), and number of imaginary vibrational frequencies (N_{imag}) of the S_N2 reaction between Nu:$^-$ + C_2H_5Cl, computed at ZORA-OLYP/QZ4P.

C_2H_5Cl
E = -870.21
H = -826.18
G = -845.81
***N*imag** = 0

Cl	-0.088649	-0.035599	0.000000
C	1.714958	0.039185	-0.000000
C	2.235369	1.464762	-0.000000
H	3.332075	1.445570	0.000000
H	1.904142	2.010996	0.886967
H	1.904142	2.010996	-0.886967
H	2.035506	-0.510585	0.887482
H	2.035506	-0.510585	-0.887482

HO^-
E = -214.34
H = -206.99
G = -219.27
***N*imag** = 0

H	0.000000	0.000000	-5.543348
O	0.000000	0.000000	-4.574783

HOO^-
E = -315.28
H = -304.94
G = -321.07
***N*imag** = 0

H	-4.825960	-2.402030	0.000000
O	-4.388133	-1.543962	0.000000
O	-2.949289	-2.040958	0.000000

H_2NO^-
E = -449.35
H = -432.17
G = -448.43
***N*imag** = 0

N	-2.229808	-1.106285	-0.332407
O	-0.970711	-0.665702	0.084470
H	-2.970639	-0.500335	0.112721
H	-2.443540	-2.028149	0.135215

HSO^-
E = -309.78
H = -301.64
G = -318.53
N_{imag} = 0

H	1.834708	-1.153433	0.000000
O	-0.401240	-2.018458	0.000000
S	1.141335	-2.365666	0.000000

CH_3O^-
E = -582.82
H = -559.30
G = -575.02
N_{imag} = 0

C	-0.386506	-1.824288	0.000000
H	0.415177	-1.000891	-0.000000
O	-1.647076	-1.417029	-0.000000
H	-0.059780	-2.471036	0.891988
H	-0.059780	-2.471036	-0.891988

CH_3OO^-
E = -684.23
H = -656.28
G = -674.79
N_{imag} = 0

C	-0.796546	-1.473601	-0.729908
H	-0.033097	-0.664472	-0.732953
O	-1.096705	-1.906991	0.552461
H	-0.400592	-2.344525	-1.292375
H	-1.690506	-1.077347	-1.260321
O	-1.618556	-0.748104	1.282874

CH_3HNO^-
E = -820.72
H = -785.02
G = -803.56
N_{imag} = 0

N	-2.269605	-1.189646	-0.342980
O	-0.971782	-0.728895	-0.147255
C	-3.243846	-0.165657	0.055136
H	-2.430155	-1.965541	0.321793
H	-4.265457	-0.591385	0.023944
H	-3.047623	0.257283	1.072287
H	-3.188348	0.670353	-0.652849

CH_3SO^-

E = -681.05

H = -654.15

G = -673.73

N_{imag} = 0

S	1.105543	-2.355051	-0.423614
O	-0.130427	-2.022767	0.498767
C	2.422667	-1.179068	0.063845
H	2.110436	-0.132548	-0.092815
H	2.698546	-1.296867	1.125548
H	3.301861	-1.390955	-0.562908

H_2N^-

E = -317.17

H = -303.35

G = -316.81

N_{imag} = 0

N	1.390446	1.340195	-0.005005
H	0.749400	1.568696	0.771989
H	0.768593	0.814829	-0.640742

$HOHN^-$

E = -436.04

H = -418.65

G = -435.3

N_{imag} = 0

N	0.699083	1.648739	-0.033325
H	1.260482	1.270356	0.753587
H	-0.179800	0.049397	-0.727055
O	0.689289	0.388322	-0.950154

H_2NHN^-

E = -562.29

H = -537.69

G = -554.42

N_{imag} = 0

N	1.330490	1.103562	0.021081
H	0.836739	1.724682	0.677054
H	0.497260	0.891146	-1.832523
N	0.224770	0.670681	-0.867533
H	0.210260	-0.355886	-0.867913

$HSHN^-$
E = −414.03
H = −398.97
G = −416.20
N_{imag} = 0

N	0.780623	1.759481	0.069857
H	1.394073	1.381424	0.804567
H	-0.392512	-0.318446	-0.755790
S	0.686871	0.534355	-1.075581

CH_3HN^-
E = −680.35
H = −649.51
G = −666.44
N_{imag} = 0

N	0.786738	1.608253	-0.100644
H	1.337494	1.104937	0.606260
H	-0.415635	-0.168451	-0.627768
C	0.290454	0.642223	-1.018362
H	-0.312836	1.146632	-1.801478
H	1.042413	0.028886	-1.623966

CH_3OHN^-
E = −803.70
H = −768.76
G = −787.85
N_{imag} = 0

O	1.063819	1.431990	-0.276769
H	1.895004	2.795096	-1.408336
H	0.215973	-0.392635	-0.613388
C	0.281377	0.605761	-1.097613
H	-0.738484	1.012450	-1.236857
H	0.718580	0.477815	-2.112309
N	1.058384	2.820408	-0.790822

CH_3HNHN^-
E = −785.38
H = −751.40
G = −771.44
N_{imag} = 0

N	1.099879	1.492407	-0.277809
H	0.661912	1.501674	0.645334
H	0.135492	-0.382288	-0.551742
C	0.257576	0.585707	-1.082005
H	-0.745818	1.013158	-1.305287
H	0.744176	0.389560	-2.046559
N	1.054048	2.857157	-0.735735

H	1.908655	2.937545	-1.304879

CH_3SHN^-

E = −785.38

H = −751.40

G = −771.44

N_{imag} = 0

S	1.224640	1.539519	-0.089060
H	1.988370	3.086801	-1.423603
H	0.072468	-0.534116	-0.649934
C	0.179629	0.435212	-1.162930
H	-0.814537	0.882764	-1.308170
H	0.637907	0.269869	-2.149783
N	1.206176	3.070837	-0.752613

HS^-

E = −182.39

H = −176.60

G = −189.88

N_{imag} = 0

S	1.340628	0.777198	0.736059
H	1.443649	-0.526208	0.400654

HOS^-

E = −312.55

H = −302.32

G = −319.22

N_{imag} = 0

H	-4.874186	-2.396410	0.000000
O	-4.417835	-1.550550	0.000000
S	-2.703361	-2.098019	0.000000

H_2NS^-

E = −431.88

H = −413.89

G = −431.09

N_{imag} = 0

N	-2.380579	-1.158884	-0.368504
S	-0.709838	-0.575052	0.122950
H	-3.022748	-0.527674	0.111660
H	-2.501533	-2.038861	0.133893

HSS^-
E = −272.38
H = −264.47
G = −282.36
N_{imag} = 0

H	-5.140926	-2.553066	0.000000
S	-4.411663	-1.403405	0.000000
S	-2.442793	-2.088509	0.000000

CH_3S^-
E = −541.90
H = −517.51
G = −534.20
N_{imag} = 0

C	-0.238292	-1.872141	-0.000004
H	0.473583	-1.029456	-0.000022
S	-1.978958	-1.309894	0.000069
H	0.002885	-2.486406	0.883957
H	0.002816	-2.486383	-0.884000

CH_3OS^-
E = −673.99
H = −646.04
G = −665.43
N_{imag} = 0

C	-0.788149	-1.472704	-0.756661
H	-0.018318	-0.676303	-0.779692
O	-1.101208	-1.876384	0.544234
H	-0.397384	-2.358088	-1.292788
H	-1.670480	-1.087483	-1.304782
S	-1.743803	-0.559004	1.526115

CH_3HNS^-
E = −796.47
H = −760.63
G = −780.13
N_{imag} = 0

N	-2.357260	-1.213102	-0.339194
S	-0.640786	-0.708501	-0.170616
C	-3.293207	-0.168684	0.062475
H	-2.468175	-1.978972	0.320531
H	-4.326209	-0.564523	0.033872
H	-3.104442	0.256755	1.071781
H	-3.226736	0.663540	-0.648773

CH_3SS^-
E = −702.88
H = −669.85
G = −690.91
N N_{imag} = 0

C	-0.718654	-1.464565	-0.789360
H	0.226404	-0.923023	-0.717185
S	-1.361817	-1.879726	0.860509
H	-0.546526	-2.427233	-1.283161
H	-1.446080	-0.889302	-1.366195
S	-1.508561	-0.075257	1.818274
H	-2.757332	0.283555	1.435971

RC: HO^- + C_2H_5Cl
E = −1101.70
H = −1050.12
G = −1075.52
N_{imag} = 0

C	-1.004445	-5.149805	0.250966
C	0.283377	-4.518048	-0.237830
Cl	1.743349	-5.545826	0.242394
H	-1.052167	-5.257890	1.342490
H	-1.154394	-6.133139	-0.205424
H	-1.827057	-4.507311	-0.084559
H	0.471981	-3.558805	0.253268
H	0.257522	-4.369124	-1.352103
O	-0.527145	-3.867573	-2.927734
H	-0.045926	-3.189521	-3.419411

RC: HOO^- + C_2H_5Cl
E = −1202.19
H = −1147.69
G = −1175.41
N_{imag} = 0

C	-4.162475	-0.297707	-1.176123
C	-3.652485	0.104298	0.156631
Cl	-2.788238	1.850147	0.219984
H	-4.934510	0.387247	-1.544975
H	-4.633778	-1.350223	-1.092160
H	-3.360380	-0.358971	-1.920277
H	-2.863067	-0.542448	0.537346
H	-4.430770	0.223020	0.909520
O	-5.339360	-2.922327	-0.970469
O	-5.198398	-3.078389	0.494808
H	-6.090531	-2.839384	0.778614

RC: H_2NO^- + C_2H_5Cl
E = −1334.43
H = −1271.93
G = −1300.47
N_{imag} = 0

Cl	0.937649	2.112783	0.962246
C	0.449075	0.662318	-0.214076
N	0.120818	-2.006894	-2.914761
H	-0.845846	-2.142860	-2.549587
H	-0.000092	-1.225257	-3.591263
H	0.786022	-0.228574	0.305894
O	0.957472	-1.597330	-1.884012
C	-1.037921	0.692927	-0.468256
H	-1.607580	0.550497	0.456368
H	-1.351878	1.641623	-0.915485
H	-1.291078	-0.117048	-1.163213
H	1.054510	0.811791	-1.102048

RC: HSO^- + C_2H_5Cl

E = −1190.33
H = −1137.76
G = −1165.96
N_{imag} = 0

C	-3.940035	0.107166	-1.319609
C	-3.667591	0.059969	0.160137
Cl	-2.696016	1.511090	0.788729
H	-4.527379	0.990130	-1.592084
H	-4.516306	-0.803580	-1.572614
H	-3.011005	0.112988	-1.898989
H	-3.074087	-0.809681	0.443023
H	-4.584410	0.069402	0.749978
O	-5.527604	-2.596801	-1.268357
S	-5.989297	-3.914055	-2.021232
H	-7.352702	-3.772484	-2.238241

RC: CH_3O^- + C_2H_5Cl
E = −1465.63
H = −1397.54
G = −1424.89
N_{imag} = 0

C	-3.968039	0.171690	0.406978
C	-2.624993	0.056130	-0.242553
Cl	-1.434013	1.450839	0.147551
H	-3.889219	0.182431	1.499620
H	-4.563304	-0.738718	0.091752
H	-4.498786	1.073607	0.083150

H	-2.683845	0.071343	-1.331599
H	-2.083752	-0.836337	0.073765
C	-6.116464	-3.062604	0.153237
H	-5.862643	-4.166486	0.099705
O	-5.329167	-2.259596	-0.583755
H	-6.110539	-2.851813	1.269957
H	-7.220797	-3.037136	-0.108105

RC: CH_3OO^- + C_2H_5Cl

E = −1566.64
H = −1494.33
G = −1523.77
N_{imag} = 0

C	-4.235625	0.313020	-0.064703
C	-2.756936	0.094940	0.086297
Cl	-1.716946	1.567733	-0.402093
H	-4.586129	1.142248	0.559288
H	-4.738816	-0.623847	0.268007
H	-4.509846	0.516185	-1.105142
H	-2.388532	-0.712224	-0.546836
H	-2.470014	-0.103297	1.118965
C	-6.297397	-4.330158	0.725768
H	-5.487295	-4.976771	0.331456
O	-5.812266	-3.340926	1.577131
H	-6.998027	-4.950661	1.315375
H	-6.836912	-3.906025	-0.145670
O	-4.882807	-2.523889	0.807394

RC: CH_3HNO^- + C_2H_5Cl

E = −1703.83
H = −1623.90
G = −1652.90
N_{imag} = 0

C	-4.002005	-0.095920	-1.292432
C	-3.639196	0.068009	0.148444
Cl	-2.642680	1.625556	0.543694
H	-4.626361	0.730397	-1.648934
H	-4.587382	-1.057538	-1.397893
H	-3.112610	-0.163011	-1.928363
H	-2.999681	-0.734644	0.517565
H	-4.511352	0.162209	0.796484
O	-5.507458	-2.662692	-1.300970
N	-5.950280	-3.315659	-2.450412
H	-7.715517	-4.038331	-1.429011
H	-5.517670	-4.250921	-2.457647
C	-7.394126	-3.556042	-2.379516
H	-7.918996	-2.595449	-2.448619

H	-7.703023	-4.188484	-3.230198

RC: CH_3SO^- + C_2H_5Cl

E = −1561.16
H = −1490.32
G = −1519.10
***N*$_{imag}$** = 0

C	-3.573312	0.315846	-1.184462
C	-3.581435	0.027650	0.295038
Cl	-2.610574	1.252763	1.290307
H	-4.019810	1.290505	-1.406594
H	-4.170006	-0.474512	-1.669490
H	-2.557162	0.296722	-1.591485
H	-3.133152	-0.937852	0.528883
H	-4.586068	0.060906	0.716485
O	-5.375876	-2.282322	-1.630092
S	-6.087050	-3.484457	-2.368907
H	-7.889914	-2.048701	-3.216656
C	-7.822074	-2.959053	-2.600286
H	-8.346264	-3.779137	-3.111405
H	-8.315510	-2.762051	-1.635543

RC: H_2N^- + C_2H_5Cl

E = −1203.34
H = −1144.55
G = −1171.42
***N*$_{imag}$** = 0

C	-0.756109	-5.820845	0.277698
C	-0.341547	-4.427257	-0.126068
Cl	1.525809	-4.173188	0.007076
H	-0.490325	-6.035340	1.318879
H	-0.296012	-6.580704	-0.363883
H	-1.849475	-5.838829	0.157230
H	-0.774249	-3.656923	0.508967
H	-0.581467	-4.198503	-1.162413
N	-3.604370	-4.401792	-0.512461
H	-3.838721	-4.120345	-1.475771
H	-4.014033	-3.628926	0.032470

RC: $HOHN^-$ + C_2H_5Cl

E = −1320.99
H = −1259.03
G = −1286.76
***N*$_{imag}$** = 0

C	-0.829311	-5.759481	0.098545
C	-0.379377	-4.324019	0.126996
Cl	1.598725	-4.238428	0.271752

H	-0.486628	-6.300382	0.987219
H	-0.450673	-6.283581	-0.785483
H	-1.929753	-5.775901	0.087253
H	-0.711210	-3.767645	0.997646
H	-0.578606	-3.763046	-0.780738
N	-3.375159	-3.975984	-0.102384
H	-3.333817	-4.087562	-1.126681
O	-4.352263	-2.822504	-0.062716
H	-4.606297	-2.868559	0.863300

RC: H_2NHN^- + C_2H_5Cl

E = −1447.58
H = −1377.45
G = −1405.17
N_{imag} = 0

C	-0.878941	-5.513753	0.784029
C	-0.383099	-4.334459	-0.013432
Cl	1.523758	-4.185009	0.067171
H	-0.575811	-5.440469	1.834513
H	-0.499759	-6.460116	0.383771
H	-1.977699	-5.492666	0.723138
H	-0.742091	-3.381486	0.367504
H	-0.606949	-4.402325	-1.075207
N	-3.500318	-4.294166	-0.553079
H	-3.455497	-5.011755	-1.290776
N	-4.584058	-3.422518	-1.058357
H	-4.168433	-2.522973	-1.332625
H	-5.143205	-3.170885	-0.240383

RC: $HSHN^-$ + C_2H_5Cl

E = −1294.30
H = −1234.10
G = −1264.00
N_{imag} = 0

C	-2.370484	-6.038293	-0.063300
C	-1.210895	-5.782034	0.861695
N	-1.039248	-4.937257	-2.872726
H	-2.633974	-7.100663	-0.091351
H	-2.059315	-5.717350	-1.079063
H	-3.255343	-5.464696	0.233067
H	-0.330482	-6.364581	0.590745
H	-0.939007	-4.726485	0.897833
Cl	-1.549195	-6.229888	2.624207
S	0.423348	-5.751099	-3.050155
H	-1.376703	-4.786739	-3.830987
H	1.447136	-4.796859	-3.067646

RC: CH_3HN^- + C_2H_5Cl

E = -1563.47
H = -1487.29
G = -1516.39
N_{imag} = 0

C	-1.121033	-5.376696	-0.053226
C	-0.008727	-4.488714	0.466816
Cl	1.646571	-5.347136	0.308328
H	-1.186683	-6.328744	0.486050
H	-0.988654	-5.589866	-1.118467
H	-2.071642	-4.841159	0.058002
H	-0.100604	-4.308387	1.540867
H	0.019610	-3.516517	-0.058508
N	-0.998522	-1.651388	-0.507419
H	-2.261197	-2.122342	-2.100183
H	-1.086053	-1.193751	0.407538
C	-2.310828	-1.692354	-1.081669
H	-3.091339	-2.326545	-0.553141
H	-2.838333	-0.706798	-1.222203

RC: CH_3OHN^- + C_2H_5Cl

E = −1685.66
H = −1605.93
G = −1637.21
N_{imag} = 0

C	-2.254742	-5.883924	-0.206924
C	-1.157211	-5.869888	0.807945
N	-0.946570	-4.994141	-2.954229
H	-2.695045	-6.881451	-0.313070
H	-1.828275	-5.580523	-1.209945
H	-3.051006	-5.175282	0.046424
H	-0.371330	-6.593875	0.594088
H	-0.713494	-4.882574	0.939268
Cl	-1.703405	-6.327582	2.563100
O	0.365726	-5.536045	-2.529378
H	-1.190386	-5.673098	-3.697848
C	1.396169	-4.704796	-2.989123
H	2.343088	-5.080390	-2.557077
H	1.250327	-3.654915	-2.677716
H	1.481163	-4.709302	-4.095684

RC: CH_3HNHN^- + C_2H_5Cl

E = −1814.33
H = −1727.53
G = −1756.79
N_{imag} = 0

C	-2.168015	-6.159385	-0.126704
C	-1.478333	-5.542620	1.046345
N	-1.107646	-4.645446	-2.614947
H	-1.942448	-7.227781	-0.214967
H	-1.802371	-5.634548	-1.066589
H	-3.255160	-6.032803	-0.071587
H	-0.397848	-5.687832	1.024054
H	-1.698137	-4.479695	1.152779
Cl	-1.973895	-6.251757	2.724614
N	0.182252	-5.179715	-2.912503
C	1.013281	-4.305253	-3.740537
H	1.985334	-4.793217	-3.947263
H	1.194441	-3.369289	-3.198445
H	0.547904	-4.028506	-4.715452
H	0.120325	-6.076038	-3.414631
H	-1.652784	-4.664877	-3.502904

RC: CH_3SHN^- + C_2H_5Cl

E = −1664.80
H = −1586.29
G = −1617.15
N_{imag} = 0

C	-2.399820	-5.931997	-0.042923
C	-1.216571	-5.849136	0.885531
N	-0.977853	-5.144427	-2.967673
H	-2.837788	-6.935551	-0.043987
H	-2.043384	-5.696743	-1.064801
H	-3.176768	-5.211282	0.233267
H	-0.443110	-6.575774	0.637027
H	-0.772459	-4.853237	0.900015
Cl	-1.637524	-6.191867	2.651694
S	0.564047	-5.784893	-2.970305
H	-1.280794	-5.137238	-3.950329
C	1.758132	-4.374344	-3.140334
H	2.774020	-4.773436	-2.997636
H	1.555894	-3.612307	-2.375151
H	1.692882	-3.904553	-4.132300

RC: HS^- + C_2H_5Cl

E = −1061.34
H = −1010.77
G = −1038.89
N_{imag} = 0

C	-2.476640	-5.617715	0.213470
C	-1.251111	-6.217439	0.860770
S	-0.503625	-5.841135	-3.161151
H	-3.347816	-6.272902	0.315318
H	-2.241235	-5.496981	-0.854527
H	-2.720216	-4.639189	0.640040
H	-1.002274	-7.192002	0.441210
H	-0.380008	-5.569000	0.767400
Cl	-1.453357	-6.501884	2.664208
H	0.037108	-4.605336	-3.140172

RC: HOS^- + C_2H_5Cl

E = −1191.92
H = −1137.25
G = −1165.32
N_{imag} = 0

C	-2.506395	-5.809562	0.101475
C	-1.165215	-6.040402	0.749569
S	-1.216913	-6.020334	-3.499420
H	-3.239034	-6.562416	0.410208
H	-2.366134	-5.881619	-0.991331
H	-2.902683	-4.816888	0.337646
H	-0.763572	-7.028062	0.520824
H	-0.432731	-5.288878	0.457646
Cl	-1.220892	-5.972718	2.591396
O	0.149386	-5.321438	-2.576522
H	0.205493	-4.441267	-2.959619

RC: H_2NS^- + C_2H_5Cl

E = −1311.83
H = −1249.45
G = −1277.43
N_{imag} = 0

C	-2.468538	-5.870923	0.048990
C	-1.116058	-5.929858	0.706699
S	-1.543271	-5.450910	-3.595812
H	-3.041158	-6.787749	0.222625
H	-2.320484	-5.754367	-1.041521
H	-3.054571	-5.019248	0.409111
H	-0.525004	-6.780963	0.369830
H	-0.538009	-5.019066	0.554993
Cl	-1.201301	-6.126186	2.553096

N	0.075531	-5.445675	-2.764749
H	0.529217	-4.580725	-3.056683
H	0.620614	-6.173969	-3.224778

RC: HSS^- + C_2H_5Cl

E = −1150.47

H = −1098.07

G = −1127.06

$\boldsymbol{N}_{\mathbf{imag}}$ = 0

C	-2.535763	-2.396560	0.772377
C	-1.189109	-2.417907	1.452139
S	-1.705459	-2.158450	-3.070146
H	-3.101640	-3.311015	0.975845
H	-2.377573	-2.322384	-0.315734
H	-3.133455	-1.537757	1.093572
H	-0.586072	-3.274632	1.151375
H	-0.617936	-1.507361	1.270836
Cl	-1.306144	-2.543449	3.287918
S	0.289913	-2.171478	-2.487435
H	0.611758	-0.849590	-2.462957

RC: CH_3S^- + C_2H_5Cl

E = −1420.83

H = −1351.88

G = −1382.13

$\boldsymbol{N}_{\mathbf{imag}}$ = 0

C	-2.590925	-5.737569	0.381700
C	-1.390204	-6.321484	1.086752
S	-0.561018	-5.902063	-2.908887
H	-3.451713	-6.412516	0.424898
H	-2.302144	-5.589814	-0.670661
H	-2.879406	-4.772367	0.810537
H	-1.102741	-7.289132	0.675619
H	-0.527032	-5.657170	1.044277
Cl	-1.679446	-6.624925	2.876995
C	0.266087	-4.406608	-3.559533
H	1.359787	-4.527497	-3.604281
H	0.068656	-3.518322	-2.938766
H	-0.069135	-4.162571	-4.579808

RC: CH_3OS^- + C_2H_5Cl

E = −1552.37

H = −1479.84

G = −1510.77

$\boldsymbol{N}_{\mathbf{imag}}$ = 0

C	-2.519434	-5.761250	0.167895

C	-1.134474	-6.031864	0.703540
S	-1.211474	-5.643798	-3.523358
H	-3.218605	-6.559961	0.436045
H	-2.443588	-5.711125	-0.929396
H	-2.915183	-4.809798	0.536617
H	-0.734217	-6.979210	0.341283
H	-0.434290	-5.237267	0.448174
Cl	-1.078740	-6.161550	2.534591
O	0.083988	-4.910803	-2.580146
C	0.478915	-3.663766	-3.081472
H	1.283816	-3.286052	-2.425572
H	-0.349256	-2.929846	-3.088270
H	0.866279	-3.730262	-4.115557

RC: CH_3HNS^- + C_2H_5Cl
E = −1675.40
H = −1595.08
G = −1625.45
N_{imag} = 0

C	-2.504496	-5.919940	0.100306
C	-1.150857	-5.867743	0.760277
S	-1.557808	-5.322418	-3.580541
H	-2.982905	-6.894398	0.241400
H	-2.367183	-5.753447	-0.982687
H	-3.171371	-5.143612	0.488585
H	-0.478035	-6.640131	0.388011
H	-0.668845	-4.897406	0.644481
Cl	-1.216091	-6.147066	2.588153
N	0.012308	-5.034230	-2.770253
C	0.593210	-3.734661	-3.093129
H	1.593810	-3.651880	-2.631670
H	-0.042604	-2.944640	-2.676577
H	0.687073	-3.532114	-4.179807
H	0.639345	-5.744179	-3.139500

RC: CH_3SS^- + C_2H_5Cl
E = −1,519.47
H = −1448.06
G = −1479.77
N_{imag} = 0

C	-2.571035	-5.835036	0.227878
C	-1.251233	-6.089880	0.914395
S	-1.485486	-5.846290	-3.593192
H	-3.293082	-6.630030	0.439287
H	-2.393995	-5.806238	-0.858442
H	-3.005008	-4.878424	0.534844
H	-0.809658	-7.041590	0.617142

H	-0.528112	-5.295831	0.729049
Cl	-1.396903	-6.191096	2.745732
S	0.398296	-5.360470	-2.917064
C	0.594858	-3.592040	-3.316458
H	1.590931	-3.282238	-2.969639
H	-0.170898	-2.988663	-2.813912
H	0.518169	-3.428249	-4.398258

TS: HO^- + C_2H_5Cl
E = −1099.17
H = −1047.88
G = −1072.02
N_{imag} = -305.95

C	-1.024773	-5.651241	0.053977
C	-0.946551	-4.163169	-0.130208
O	1.427037	-4.281599	-0.205793
H	-1.518703	-5.908166	0.996498
H	-0.002845	-6.040129	0.063903
H	-1.583030	-6.122410	-0.761292
H	-0.635828	-3.558826	0.704821
H	-0.704161	-3.779319	-1.106401
Cl	-3.000430	-3.530546	-0.129721
H	1.733848	-3.372970	-0.327627

TS: HOO^- + C_2H_5Cl
E = −1199.77
H = −1145.25
G = −1171.83
N_{imag} = -204.71

C	-0.817716	-5.817447	0.251910
C	-0.663442	-4.349668	-0.018622
Cl	1.420502	-3.985150	0.140934
H	-0.455292	-6.079102	1.251289
H	-0.261541	-6.415488	-0.477083
H	-1.880422	-6.079285	0.180059
H	-0.984142	-3.659091	0.744819
H	-0.798756	-4.002005	-1.030225
O	-3.052627	-4.263045	-0.296581
O	-3.573046	-2.989297	-0.804631
H	-3.763918	-2.528222	0.023814

TS: H_2NO^- + C_2H_5Cl
E = −1334.11
H = −1271.88
G = −1298.51
N_{imag} = -226.07

Cl	1.600980	1.805684	-0.000001

C	0.058643	0.303018	0.000002
N	-0.475359	-2.879277	-0.000003
H	-0.761965	-3.450606	0.810690
H	-0.761973	-3.450602	-0.810695
H	0.359237	-0.191190	0.909295
O	-1.242363	-1.707656	0.000003
C	-1.217795	1.087592	-0.000001
H	-1.293590	1.727010	0.885399
H	-1.293591	1.727004	-0.885405
H	-2.058014	0.382117	0.000003
H	0.359238	-0.191194	-0.909288

TS: HSO^- + C_2H_5Cl

E = −1183.47

H = −1131.05

G = −1158.06

N_{imag} = 0

C	0.928234	1.640876	-0.052669
H	0.017058	2.249677	0.002487
H	1.443207	1.868870	-0.989741
H	1.586803	1.922150	0.773376
O	-1.585100	0.626580	0.096796
H	-2.758438	-1.105179	1.175630
C	0.551839	0.196006	0.021439
H	0.374211	-0.275203	0.972544
Cl	2.680335	-0.762273	-0.023732
H	0.295879	-0.358691	-0.864160
S	-2.606758	-0.581509	-0.090275

TS: CH_3O^- + C_2H_5Cl

E = −1461.30

H = −1392.52

G = −1419.51

N_{imag} = −361.608

C	-1.026470	-5.644992	0.589593
C	-0.741334	-4.438482	-0.254139
O	1.525338	-4.773234	-0.041739
H	-1.593511	-5.375595	1.485713
H	-0.070787	-6.086082	0.889218
H	-1.605685	-6.388266	0.033567
H	-0.438755	-3.521048	0.221276
H	-0.450654	-4.569681	-1.282477
Cl	-2.748282	-3.714219	-0.743396
C	2.267474	-3.841443	-0.691555
H	2.097592	-3.787473	-1.804545
H	3.370635	-4.016468	-0.589612
H	2.121251	-2.781808	-0.335937

TS: CH_3OO^- + C_2H_5Cl

E = -1563.25

H = -1490.89

G = -1519.62

N_{imag} = -274.04

C	-0.916397	-1.620951	-2.062116
C	-1.196213	-0.327799	-1.359837
O	0.393817	-0.904286	0.220102
H	-1.814781	-2.242770	-2.121372
H	-0.143686	-2.170401	-1.510021
H	-0.556590	-1.447040	-3.080605
H	-1.821552	-0.328479	-0.484017
H	-0.497686	0.486272	-1.450199
Cl	-2.724848	0.603468	-2.645193
O	0.671111	0.132755	1.184535
C	0.050467	-0.223594	2.389813
H	-1.045897	-0.310045	2.286766
H	0.281345	0.577015	3.112200
H	0.432787	-1.184992	2.780239

TS: CH_3HNO^- + C_2H_5Cl

E = -1700.91

H = -1620.63

G = -1649.37

N_{imag} = -271.75

Cl	1.527077	2.005466	0.144905
C	0.035777	0.376792	0.024906
N	-0.303841	-2.757400	0.054916
H	-0.182227	-4.524508	1.184965
H	-0.400058	-3.326521	-0.795934
H	0.320661	-0.067855	0.963003
O	-1.111996	-1.640816	-0.093534
C	-1.257818	1.122329	-0.076465
H	-1.426269	1.748751	0.804852
H	-1.276567	1.769180	-0.958891
H	-2.077239	0.396658	-0.153643
H	0.463484	-0.064925	-0.858756
C	-0.761242	-3.587002	1.169989
H	-0.583602	-3.052955	2.110803
H	-1.844015	-3.830405	1.125887

TS: CH_3SO^- + C_2H_5Cl
E = -1553.51
H = -1482.27
G = -1512.18
N**imag** = 0

C	0.926652	1.660636	-0.052888
H	-0.005938	2.235166	0.018617
H	1.425425	1.923625	-0.989537
H	1.580540	1.951712	0.773581
O	-1.559226	0.557642	0.071040
H	-1.950470	-1.663489	2.066403
C	0.603612	0.203713	-0.001361
H	0.465654	-0.291615	0.943157
Cl	2.789168	-0.687523	-0.083250
H	0.366607	-0.347574	-0.894017
S	-2.558323	-0.664828	-0.092042
C	-2.863901	-1.271814	1.602167
H	-3.278971	-0.477365	2.236423
H	-3.596644	-2.085104	1.518942

TS: H_2N^- + C_2H_5Cl
E = -1202.36
H = -1144.27
G = -1169.32
N**imag** = -237.89

C	-0.841087	-5.793819	0.177554
C	-0.551084	-4.323103	0.043398
Cl	1.530984	-4.138434	0.279821
H	-0.467047	-6.180716	1.131498
H	-0.368493	-6.367265	-0.627124
H	-1.925244	-5.931727	0.136976
H	-0.872111	-3.680167	0.848928
H	-0.645692	-3.874214	-0.933367
N	-3.164884	-3.827522	-0.308888
H	-3.202095	-3.844853	-1.338696
H	-3.272145	-2.820400	-0.118932

TS: $HOHN^-$ + C_2H_5Cl
E = -1320.62
H = -1258.58
G = -1285.53
N**imag** = -53.00

C	-0.752709	-5.837237	0.167758
C	-0.434818	-4.366574	0.056176
Cl	1.500444	-4.108778	0.343807
H	-0.465586	-6.231049	1.148756
H	-0.229918	-6.420928	-0.597911

H	-1.834738	-5.953478	0.041861
H	-0.889220	-3.743998	0.818311
H	-0.593754	-3.937201	-0.927000
N	-3.323117	-3.968954	-0.346880
H	-3.429877	-3.672560	-1.330860
O	-3.852711	-2.713766	0.321959
H	-4.438569	-3.107530	0.974994

TS: H_2NHN^- + C_2H_5Cl

E = -1447.07

H = -1377.52

G = -1405.11

N_{imag} = -25.54

C	-0.786686	-5.838106	0.233326
C	-0.424152	-4.407335	-0.076121
Cl	1.501037	-4.173790	-0.050258
H	-0.384937	-6.149959	1.203573
H	-0.407761	-6.525138	-0.530993
H	-1.882599	-5.891292	0.268244
H	-0.782720	-3.699209	0.665042
H	-0.718067	-4.078873	-1.068780
N	-3.462801	-4.171779	-0.325795
H	-3.530117	-4.569103	-1.273803
N	-4.420780	-3.051784	-0.406111
H	-3.894510	-2.169402	-0.487864
H	-4.834003	-2.981539	0.525017

TS: $HSHN^-$ + C_2H_5Cl

E = -1287.05

H = -1227.52

G = -1255.1

N_{imag} = -340.38

C	-0.836120	-5.807669	0.104981
C	-0.510936	-4.347427	-0.028797
Cl	1.703909	-4.385234	0.067650
H	-0.395167	-6.219978	1.017140
H	-0.446444	-6.379533	-0.742575
H	-1.921243	-5.944241	0.160810
H	-0.616300	-3.696149	0.821563
H	-0.519316	-3.878630	-0.997154
N	-2.832846	-3.719977	-0.132111
H	-3.166580	-4.161802	-0.996233
S	-3.146096	-2.066085	-0.352846
H	-4.138470	-1.811712	0.561913

TS: CH_3HN^- + C_2H_5Cl
E = -1563.1
H = -1487.22
G = -1514.67
N_{imag} = -190.80

C	-0.726660	-5.927223	0.257567
C	-0.510982	-4.441721	0.149319
Cl	1.524787	-4.140168	0.466121
H	-0.498224	-6.291553	1.264872
H	-0.087500	-6.468454	-0.448139
H	-1.773752	-6.141006	0.020589
H	-0.904707	-3.819828	0.939767
H	-0.612479	-4.004412	-0.832907
N	-3.138948	-4.090465	-0.311711
H	-3.382122	-4.115220	0.686055
H	-3.201209	-2.671275	-1.845298
C	-3.430158	-2.755456	-0.767384
H	-4.499206	-2.429845	-0.675857
H	-2.850733	-1.921787	-0.282810

TS: CH_3OHN^- + C_2H_5Cl
E = -1683.60
H = -1604.06
G = -1633.89
N_{imag} = -149.45

C	-0.736821	-5.804404	0.046705
C	-0.495446	-4.332824	0.232258
Cl	1.555701	-4.121692	0.609693
H	-0.389190	-6.370391	0.917414
H	-0.210297	-6.187252	-0.833862
H	-1.812097	-5.989595	-0.071804
H	-0.895734	-3.882407	1.129193
H	-0.578510	-3.697798	-0.638199
N	-3.172793	-4.024700	-0.240363
H	-3.008847	-4.153937	-1.249872
O	-3.780161	-2.678762	-0.304364
H	-3.839424	-2.551722	1.772618
H	-5.313702	-3.199143	1.004037
C	-4.486610	-2.471194	0.878613
H	-4.902383	-1.453161	0.836399

TS: CH_3HNHN^- + C_2H_5Cl
E = -1812.73
H = -1725.39
G = -1755.09
N_{imag} = -184.75

C	-0.692688	-5.823652	0.062620
C	-0.533120	-4.332104	0.194125

Cl	1.473535	-4.000793	0.571957
H	-0.332050	-6.335962	0.960880
H	-0.129930	-6.210674	-0.793520
H	-1.753663	-6.059546	-0.066804
H	-0.976653	-3.876059	1.067362
H	-0.654098	-3.743785	-0.704276
N	-3.187093	-3.999600	-0.266116
H	-3.160077	-4.252130	-1.263579
N	-3.686857	-2.646070	-0.290383
H	-4.071246	-2.739562	1.831058
H	-2.899957	-1.985751	-0.211372
C	-4.525458	-2.404605	0.875429
H	-4.745546	-1.326932	0.953432
H	-5.472277	-2.950707	0.764221

TS: CH_3SHN^- + C_2H_5Cl

E = -1656.74
H = -1578.34
G = -1608.60
N_{imag} = -337.19

C	-0.908819	-5.830383	0.380737
C	-0.550428	-4.412546	0.038021
Cl	1.677829	-4.543399	-0.018282
H	-0.523996	-6.109303	1.366171
H	-0.485383	-6.524192	-0.351166
H	-1.997587	-5.952282	0.375484
H	-0.534571	-3.657915	0.804424
H	-0.625029	-4.068021	-0.979342
N	-2.829406	-3.711584	-0.080612
H	-3.208293	-3.891862	0.855643
H	-4.570374	-1.126308	-2.090413
S	-3.158788	-2.095725	-0.405061
H	-5.364238	-2.570076	-1.385304
H	-4.046636	-2.766088	-2.575573
C	-4.411585	-2.155832	-1.741249

TS: HS^- + C_2H_5Cl

E = -1051.93
H = -1002.39
G = -1028.35
N_{imag} = -331.364

C	-1.103333	-5.734034	0.081575
C	-0.795478	-4.279169	-0.142094
S	1.847725	-4.453765	-0.209439
H	-1.656644	-5.868980	1.015013
H	-0.187983	-6.329126	0.136534
H	-1.718119	-6.124915	-0.733830

H	-0.561858	-3.636172	0.686671
H	-0.625512	-3.902564	-1.134120
Cl	-2.957180	-3.436254	-0.190040
H	2.038026	-3.134702	-0.407133

TS: HOS^- + C_2H_5Cl

E = -1185.34

H = -1131.20

G = -1159.39

N_{imag} = -288.907

C	-0.810121	-5.842773	0.240998
C	-0.780938	-4.363209	-0.022830
Cl	1.409196	-3.918367	0.255461
H	-0.445628	-6.062447	1.248627
H	-0.172150	-6.373369	-0.471692
H	-1.823625	-6.250820	0.151143
H	-1.077129	-3.675441	0.749871
H	-0.793488	-3.995682	-1.034515
S	-3.464503	-4.220964	-0.479860
O	-3.842904	-2.613321	-1.042878
H	-4.004156	-2.124147	-0.229351

TS: H_2NS^- + C_2H_5Cl

E = -1305.51

H = -1243.56

G = -1271.89

N_{imag} = -291.941

C	-0.801145	-5.852310	0.253792
C	-0.818026	-4.380768	-0.052262
Cl	1.375489	-3.891010	0.098723
H	-0.386156	-6.035538	1.249269
H	-0.184402	-6.390861	-0.471727
H	-1.810032	-6.278164	0.223124
H	-1.094859	-3.674788	0.711506
H	-0.890014	-4.043450	-1.071817
S	-3.522625	-4.233634	-0.394101
N	-3.785999	-2.525072	-0.808183
H	-4.445617	-2.141140	-0.134819
H	-4.261453	-2.494112	-1.707588

TS: HSS^- + C_2H_5Cl
E = -1140.77
H = -1088.91
G = -1117.72
N_{imag} = -309.035

C	-0.754621	-5.900533	0.260073
C	-0.821290	-4.423877	-0.007432
Cl	1.430155	-3.900526	0.237026
H	-0.317561	-6.093614	1.243476
H	-0.134203	-6.400429	-0.488594
H	-1.748598	-6.363285	0.236249
H	-1.070046	-3.735291	0.780741
H	-0.806134	-4.042567	-1.013038
S	-3.417203	-4.367374	-0.404705
S	-3.960911	-2.507515	-1.088953
H	-4.173462	-1.827261	0.067554

TS: CH_3S^- + C_2H_5Cl
E = -1413.74
H = -1345.74
G = -1373.21
N_{imag} = -315.837

C	-1.252159	-5.845125	0.088497
C	-0.902570	-4.385359	-0.002768
S	1.733540	-4.695840	-0.518572
H	-1.656052	-6.082727	1.076766
H	-0.372093	-6.471167	-0.082487
H	-2.009278	-6.106311	-0.656383
H	-0.461412	-3.882044	0.839284
H	-0.821630	-3.908818	-0.963716
Cl	-2.930184	-3.446677	0.388699
C	2.212785	-2.944832	-0.656762
H	1.977052	-2.380180	0.256254
H	1.710759	-2.443590	-1.496232
H	3.294609	-2.857175	-0.823749

TS: CH_3OS^- + C_2H_5Cl
E = -1544.23
H = -1472.20
G = -1502.80
N_{imag} = -301.234

C	-0.791043	-5.892359	0.208324
C	-0.721385	-4.406534	-0.001899
Cl	1.519086	-4.055137	0.262634
H	-0.405548	-6.159300	1.196315
H	-0.191262	-6.415476	-0.541619
H	-1.818913	-6.268962	0.135906

H	-0.956984	-3.735884	0.805291
H	-0.707851	-3.997337	-0.996848
S	-3.367413	-4.212205	-0.442119
O	-3.783039	-2.609755	-0.919804
C	-4.189201	-1.781221	0.149772
H	-3.395742	-1.657153	0.904079
H	-5.084247	-2.175530	0.659469
H	-4.426716	-0.797272	-0.283346

TS: CH_3HNS^- + C_2H_5Cl

E = -1668.07
H = -1588.18
G = -1618.83
$\boldsymbol{N}_{\mathbf{imag}}$ = -301.221

C	-0.799657	-5.854237	0.231598
C	-0.842295	-4.376574	-0.035956
Cl	1.366222	-3.858454	0.148188
H	-0.378929	-6.055367	1.221021
H	-0.175861	-6.363125	-0.508859
H	-1.800705	-6.298798	0.193340
H	-1.116934	-3.692233	0.747672
H	-0.892782	-4.008543	-1.045745
S	-3.519834	-4.274929	-0.411252
N	-3.856120	-2.594783	-0.779255
C	-4.751772	-1.923248	0.155481
H	-4.259837	-2.558260	-1.710179
H	-4.273686	-1.877946	1.139729
H	-5.732951	-2.419552	0.290104
H	-4.927681	-0.891234	-0.188380

TS: CH_3SS^- + C_2H_5Cl

E = -1509.40
H = -1438.56
G = -1470.13
$\boldsymbol{N}_{\mathbf{imag}}$ = -307.487

C	-0.763308	-5.925440	0.245577
C	-0.793065	-4.441790	0.017989
Cl	1.483999	-3.977951	0.263193
H	-0.318163	-6.156311	1.217218
H	-0.167589	-6.422261	-0.524917
H	-1.770009	-6.360384	0.224079
H	-1.013619	-3.769581	0.827982
H	-0.770253	-4.032137	-0.976204
S	-3.393741	-4.307972	-0.387202
S	-3.921974	-2.454163	-1.046191
C	-4.283222	-1.490703	0.461356
H	-3.393899	-1.401773	1.092653

H	-5.091112	-1.957157	1.034621
H	-4.596910	-0.490759	0.134967

PC: HO^- + C_2H_5Cl

E = −1143.55
H = −1089.47
G = −1115.00
N_{imag} = 0

C	-0.704050	-5.347839	-0.009870
C	-0.007807	-4.008442	0.152633
O	1.416597	-4.104748	0.396951
H	-0.586624	-5.965196	0.888385
H	-0.291363	-5.901933	-0.864543
H	-1.773359	-5.192544	-0.207929
H	-0.406011	-3.471596	1.019623
H	-0.194054	-3.380049	-0.730173
Cl	-4.293621	-4.375065	-1.040341
H	1.772277	-4.663023	-0.302927

PC: HOO^- + C_2H_5Cl

E = −1238.46
H = −1182.46
G = −1208.09
N_{imag} = 0

C	-0.702366	-5.354037	-0.129887
C	-0.079819	-4.015994	0.221964
O	1.346373	-4.210352	0.283386
H	-0.538256	-6.092182	0.662407
H	-0.292412	-5.745013	-1.067167
H	-1.780366	-5.190690	-0.269386
H	-0.443261	-3.649183	1.189644
H	-0.324462	-3.269528	-0.546336
Cl	-4.113867	-3.982451	-0.890549
O	1.976920	-2.939882	0.661750
H	2.080533	-2.526868	-0.210251

PC: H_2HO^- + C_2H_5Cl

E = −1374.02
H = −1309.02
G = −1337.28
N_{imag} = 0

C	-0.554753	-5.977708	0.399454
C	-0.125491	-4.548514	0.143643
O	1.311622	-4.469479	0.294500
H	-0.287157	-6.294159	1.413892
H	-0.083817	-6.664176	-0.313154
H	-1.644516	-6.050347	0.286380

H	-0.604064	-3.860850	0.853503
H	-0.402943	-4.230286	-0.870072
Cl	-4.403889	-5.923564	-0.075356
N	1.747524	-3.112351	0.055050
H	2.280295	-2.883383	0.897552
H	2.453499	-3.216166	-0.677809

PC: HSO^- + C_2H_5Cl
E = −1208.58
H = −1154.34
G = −1182.57
N_{imag} = 0

C	-0.746575	-5.696698	0.217106
C	-0.143666	-4.318427	0.339346
O	1.317691	-4.449307	0.422962
H	-0.532641	-6.304663	1.102895
H	-0.366984	-6.217551	-0.668552
H	-1.836469	-5.579184	0.113725
H	-0.504336	-3.797573	1.232912
H	-0.387997	-3.703652	-0.535505
Cl	-4.282904	-4.632702	-0.248787
S	2.151146	-3.043131	0.703022
H	2.143623	-2.510975	-0.549337

PC: CH_3O^- + C_2H_5Cl
E = −1500.46
H = −1428.38
G = −1456.48
N_{imag} = 0

C	-0.625975	-5.397081	0.169364
C	-0.053885	-3.987645	0.199972
O	1.362271	-3.897317	0.470122
H	-0.436068	-5.915624	1.116456
H	-0.209219	-5.998667	-0.645755
H	-1.709410	-5.326076	0.008647
H	-0.523326	-3.416058	1.006566
H	-0.280502	-3.465734	-0.741544
Cl	-4.259475	-4.361020	-0.732931
C	2.183688	-4.233404	-0.623073
H	2.109148	-5.294543	-0.910189
H	3.217999	-4.031915	-0.321611
H	1.953336	-3.623581	-1.514249

PC: CH_3OO^- + C_2H_5Cl
E = −1600.14
H = −1525.53
G = −1554.40
N_{imag} = 0

C	-1.331610	-2.229560	-1.766750
C	-0.760236	-1.089644	-0.930695
O	0.212402	-1.509163	0.038912
H	-1.767935	-3.013240	-1.136113
H	-0.556229	-2.676831	-2.394487
H	-2.111985	-1.838683	-2.430106
H	-1.537108	-0.651343	-0.287507
H	-0.365159	-0.283248	-1.561119
Cl	0.584626	1.950618	-3.001926
O	1.451213	-1.882366	-0.658686
C	2.306718	-0.740539	-0.663984
H	1.884848	0.103893	-1.221827
H	3.206975	-1.095359	-1.182319
H	2.571711	-0.441918	0.361083

PC: CH_3HNO^- + C_2H_5Cl
E = −1738.17
H = −1655.81
G = −1684.21
N_{imag} = 0

C	-0.590527	-6.020022	0.323004
C	-0.170808	-4.580053	0.115457
O	1.273449	-4.507013	0.181819
H	-0.249265	-6.393599	1.295007
H	-0.180436	-6.667498	-0.460283
H	-1.686318	-6.078592	0.293147
H	-0.605655	-3.934189	0.890433
H	-0.508273	-4.205065	-0.859180
Cl	-4.461908	-5.783807	0.253919
N	1.706136	-3.149374	-0.047822
H	2.060356	-2.858309	0.866168
H	2.489271	-3.492706	-1.958617
C	2.842123	-3.210967	-0.962022
H	3.277593	-2.205580	-1.019924
H	3.626728	-3.922738	-0.655703

PC: CH_3SO^- + C_2H_5Cl
E = −1580.88
H = −1507.52
G = −1537.40
N_{imag} = 0

C	-0.603878	-6.011046	0.374868

C	-0.090390	-4.773149	-0.338860
O	1.177438	-4.406229	0.280704
H	-0.784388	-5.801268	1.433541
H	0.098675	-6.848866	0.296126
H	-1.558063	-6.309936	-0.072418
H	-0.810367	-3.952132	-0.248932
H	0.086407	-4.975014	-1.404753
Cl	-2.750317	-1.950364	0.124084
S	1.916339	-3.051014	-0.352673
H	1.480353	-0.784727	0.001527
C	1.052552	-1.687180	0.457312
H	-0.027172	-1.702734	0.265030
H	1.253911	-1.674540	1.533325

PC: H_2N^- + C_2H_5Cl
E = −1265.48
H = −1203.52
G = −1228.25
N_{imag} = 0

C	-0.252013	-6.173113	-0.342438
C	-0.574438	-4.683852	-0.194815
N	0.396388	-3.992577	0.651071
H	-0.201301	-6.660139	0.638075
H	0.717718	-6.322488	-0.835384
H	-1.011231	-6.685010	-0.948011
H	-1.557695	-4.574032	0.279556
H	-0.677720	-4.239586	-1.203022
Cl	-0.482093	-0.648080	0.512767
H	1.284796	-3.940048	0.156786
H	0.106890	-3.008715	0.751635

PC: $HOHN^-$ + C_2H_5Cl
E = −1387.55
H = −1322.62
G = −1348.47
N_{imag} = 0

C	-0.163427	-6.137580	-0.441456
C	-0.594628	-4.715891	-0.082836
N	0.244951	-4.129443	0.971722
H	-0.187707	-6.788425	0.440380
H	0.857066	-6.139637	-0.835369
H	-0.825389	-6.562983	-1.206288
H	-1.616191	-4.729602	0.327025
H	-0.612575	-4.085516	-0.985868
Cl	0.493580	-0.908072	-0.262753
O	1.523391	-3.755168	0.419445
H	-0.180017	-3.229451	1.208566

H	1.361241	-2.835879	0.067198

PC: H_2NHN^- + C_2H_5Cl

E = −1513.96

H = −1440.57

G = −1467.06

N_{imag} = 0

C	-0.207491	-6.217472	-0.333368
C	-0.580423	-4.738560	-0.210606
N	0.406333	-3.963498	0.539047
H	-0.102409	-6.676162	0.656956
H	0.743926	-6.330332	-0.861945
H	-0.975196	-6.769078	-0.891827
H	-1.526903	-4.642189	0.338648
H	-0.756338	-4.321971	-1.219019
Cl	-0.084466	-0.618449	0.163542
N	1.557088	-3.670089	-0.298181
H	-0.014465	-3.053869	0.759521
H	2.350281	-3.639705	0.338457
H	1.434631	-2.700054	-0.610219

PC: $HSHN^-$ + C_2H_5Cl

E = −1338.08

H = −1276.04

G = −1303.15

N_{imag} = 0

C	-0.215149	-6.036491	-0.142311
C	-0.438273	-4.542487	-0.387428
N	0.542495	-3.692698	0.289998
H	-0.245765	-6.262428	0.929368
H	0.755170	-6.364817	-0.531326
H	-0.992714	-6.628347	-0.642604
H	-1.408428	-4.235060	0.017950
H	-0.477903	-4.354089	-1.474050
Cl	-1.315516	-1.154994	1.077958
S	2.021696	-3.430982	-0.511077
H	0.109186	-2.788893	0.564468
H	2.839748	-4.181679	0.270720

PC: CH_3HN^- + C_2H_5Cl

E = −1617.53

H = −1541.04

G = −1568.69

N_{imag} = 0

C	-0.704285	-5.939588	0.327532
C	-0.634267	-4.462570	0.064286
Cl	1.388133	-3.950356	0.436514

H	-0.441834	-6.174535	1.362627
H	-0.037843	-6.497170	-0.336303
H	-1.725459	-6.289168	0.150187
H	-1.073712	-3.792195	0.786837
H	-0.680862	-4.119221	-0.957428
N	-3.217140	-4.141550	-0.520820
H	-3.356159	-4.305746	0.478317
H	-3.232437	-2.469009	-1.796114
C	-3.370741	-2.711349	-0.730203
H	-4.366273	-2.294029	-0.460360
H	-2.639016	-2.071926	-0.184889

PC: CH_3OHN^- + C_2H_5Cl

E = −1737.76
H = −1657.84
G = −1688.22
N_{imag} = 0

C	-0.703238	-5.831035	0.029670
C	-0.658416	-4.346802	0.249636
Cl	1.350520	-3.910926	0.734928
H	-0.405420	-6.377175	0.929173
H	-0.052038	-6.134659	-0.794510
H	-1.725538	-6.136162	-0.219813
H	-1.119756	-3.951591	1.142456
H	-0.738614	-3.702360	-0.613972
N	-3.313986	-4.125770	-0.344903
H	-2.961938	-4.164317	-1.308433
O	-3.827378	-2.733937	-0.359701
H	-3.606438	-2.523503	1.703101
H	-5.199584	-3.120912	1.162097
C	-4.365388	-2.444675	0.907361
H	-4.739102	-1.415157	0.871377

PC: CH_3HNHN^- + C_2H_5Cl

E = −1867.31
H = −1779.39
G = −1809.54
N_{imag} = 0

C	-0.649103	-5.848301	0.080647
C	-0.676753	-4.348705	0.181855
Cl	1.290032	-3.781617	0.611112
H	-0.345427	-6.307748	1.025639
H	0.033370	-6.184348	-0.705058
H	-1.650938	-6.213733	-0.163994
H	-1.166611	-3.910056	1.038479
H	-0.803615	-3.789188	-0.733920
N	-3.352214	-4.064601	-0.373472

H	-3.108345	-4.199344	-1.359648
N	-3.690765	-2.650462	-0.307965
H	-3.962485	-2.784491	1.822671
H	-2.823938	-2.108674	-0.224169
C	-4.443001	-2.378130	0.912751
H	-4.554771	-1.294210	1.038218
H	-5.442613	-2.824323	0.841888

PC: CH_3SHN^- + C_2H_5Cl

E = −1708.06

H = −1629.47

G = −1660.08

N_{imag} = 0

C	-0.803164	-5.891042	0.459366
C	-0.513759	-4.478371	0.050417
Cl	1.683131	-4.403402	0.084778
H	-0.407900	-6.108234	1.454784
H	-0.380483	-6.606053	-0.250930
H	-1.885186	-6.051273	0.485916
H	-0.624181	-3.683767	0.769912
H	-0.583242	-4.195506	-0.986561
N	-2.901161	-3.829149	-0.275168
H	-3.245012	-3.972055	0.678988
H	-4.558100	-0.986067	-2.041793
S	-3.085904	-2.177958	-0.581979
H	-5.430832	-2.343184	-1.273962
H	-4.311608	-2.650280	-2.635678
C	-4.489903	-2.039176	-1.744612

PC: HS^- + C_2H_5Cl

E = −1079.92

H = −1028.69

G = −1055.51

N_{imag} = 0

C	-0.639076	-5.489707	0.002347
C	-0.095281	-4.071312	0.076258
S	1.693784	-3.910462	0.475288
H	-0.478002	-6.029672	0.941391
H	-0.160113	-6.057724	-0.804010
H	-1.715606	-5.429306	-0.203565
H	-0.589842	-3.512296	0.876580
H	-0.295340	-3.526034	-0.848486
Cl	-4.107564	-4.081145	-0.767111
H	2.169289	-4.569151	-0.598553

PC: HOS^- + C_2H_5Cl
E = −1219.68
H = −1164.50
G = −1192.25
N_{imag} = 0

C	-0.821176	-5.874248	-0.052331
C	-0.290187	-4.462365	-0.284525
S	1.232786	-4.035167	0.566025
H	-0.990282	-6.054785	1.013293
H	-0.134376	-6.640052	-0.429120
H	-1.783769	-5.983658	-0.564605
H	-1.017713	-3.711392	0.092639
H	-0.172461	-4.245422	-1.353563
Cl	-3.166172	-2.462267	0.375914
O	2.411277	-5.043826	-0.154068
H	2.728610	-4.551522	-0.921983

PC: H_2NS^- + C_2H_5Cl
E = −1346.99
H = −1283.73
G = −1311.14
N_{imag} = 0

C	-0.865181	-5.653396	0.879317
C	-0.164194	-4.316403	0.644278
S	1.615325	-4.403749	0.208464
H	-0.382294	-6.226275	1.678999
H	-0.861877	-6.261170	-0.030528
H	-1.916886	-5.495397	1.155263
H	-0.173412	-3.703963	1.556977
H	-0.665416	-3.749283	-0.146209
Cl	-0.553358	-3.799668	-3.474818
N	1.716714	-4.985344	-1.381963
H	1.123179	-4.451759	-2.044593
H	1.429281	-5.958107	-1.435448

PC: HSS^- + C_2H_5Cl
E = −1167.39
H = −1114.49
G = −1143.16
N_{imag} = 0

C	-0.844073	-5.874081	-0.051041
C	-0.298795	-4.476174	-0.306735
S	1.254480	-4.057619	0.542174
H	-1.005302	-6.040598	1.018082
H	-0.177031	-6.655489	-0.431068
H	-1.815108	-5.969992	-0.549369

H	-1.010045	-3.712360	0.070990
H	-0.166435	-4.278152	-1.375139
Cl	-3.132227	-2.487639	0.396330
S	2.729089	-5.244025	-0.290996
H	3.092762	-4.462850	-1.337183

PC: CH_3S^- + C_2H_5Cl

E = −1447.69
H = −1377.39
G = −1405.87
N_{imag} = 0

C	-0.668134	-5.696925	0.752663
C	-0.156496	-4.281542	0.487960
S	1.649398	-4.119311	0.276993
H	-0.201882	-6.139621	1.640143
H	-0.479037	-6.352043	-0.102091
H	-1.755136	-5.674432	0.904142
H	-0.385669	-3.626492	1.337240
H	-0.635916	-3.860396	-0.403074
Cl	-1.567280	-3.655948	-3.102012
C	1.862598	-4.634446	-1.452743
H	2.847121	-4.273243	-1.765630
H	1.086323	-4.186125	-2.080939
H	1.836447	-5.723041	-1.559997

PC: CH_3OS^- + C_2H_5Cl

E = −1576.67
H = −1503.49
G = −1533.90
N_{imag} = 0

C	-0.857851	-5.816754	-0.142409
C	-0.348503	-4.381539	-0.241318
S	1.200199	-4.030826	0.602990
H	-0.978627	-6.115305	0.902990
H	-0.182840	-6.529205	-0.629456
H	-1.840855	-5.880634	-0.622425
H	-1.064757	-3.685816	0.246591
H	-0.283859	-4.046220	-1.282726
Cl	-3.219904	-2.523627	0.745366
O	2.361044	-4.970843	-0.208161
C	2.933791	-4.347487	-1.356885
H	3.399045	-3.384705	-1.105703
H	2.192707	-4.184383	-2.151076
H	3.702629	-5.037826	-1.726039

PC: CH_3HNS^- + C_2H_5Cl
E = −1706.84
H = −1625.65
G = −1655.53
N_{imag} = 0

C	-1.055554	-5.604080	1.148278
C	-0.356453	-4.364025	0.585983
S	1.455672	-4.482459	0.267635
H	-0.569040	-5.956237	2.064897
H	-1.051569	-6.428130	0.430466
H	-2.105420	-5.380187	1.388153
H	-0.435307	-3.530285	1.297224
H	-0.813148	-4.030163	-0.350998
Cl	-0.170580	-3.588731	-3.576961
N	1.699610	-5.048199	-1.298930
H	1.228105	-4.455730	-2.007872
C	1.497784	-6.464566	-1.589134
H	1.915229	-6.668843	-2.582445
H	0.442434	-6.777835	-1.609033
H	2.031869	-7.073952	-0.851931

PC: CH_3SS^- + C_2H_5Cl
E = −1535.57
H = −1463.57
G = −1495.16
N_{imag} = 0

C	-0.893104	-5.852385	-0.065210
C	-0.299618	-4.464226	-0.260196
S	1.308004	-4.170530	0.553667
H	-1.025743	-6.075106	0.997712
H	-0.270449	-6.635548	-0.511072
H	-1.883129	-5.881304	-0.533837
H	-0.961933	-3.695109	0.181978
H	-0.198803	-4.210054	-1.319609
Cl	-3.072051	-2.376106	0.565712
S	2.705449	-5.297769	-0.436974
C	3.245428	-4.242313	-1.825030
H	3.631948	-3.288580	-1.458047
H	2.430453	-4.066267	-2.531150
H	4.047548	-4.790005	-2.333247

P: HO^- + C_2H_5Cl
E = −1050.70
H = −998.30
G = −1017.63
N_{imag} = 0

H	0.042472	-0.750600	2.778548

H	-0.833910	-1.294388	1.334443
O	2.161682	0.201422	1.473370
H	0.727185	-2.026330	1.754532
H	0.961623	-0.387891	-0.132938
H	0.275758	0.890534	0.893542
C	0.160305	-1.090475	1.745169
C	0.866909	-0.038850	0.907549
H	2.600304	0.868887	0.935339

P: HOO^- + C_2H_5Cl

E = −1143.93
H = −1088.87
G = −1110.44
N_{imag} = 0

H	0.096357	-0.735698	2.774902
H	-0.884341	-1.266603	1.396062
O	2.099468	0.181349	1.333398
H	0.691473	-2.018888	1.700938
H	0.811233	-0.365139	-0.206892
H	0.216224	0.919977	0.877730
C	0.138109	-1.076133	1.736666
C	0.774556	-0.024934	0.838672
O	2.704833	1.247769	0.538661
H	3.373959	0.739414	0.054441

P: H_2NO^- + C_2H_5Cl

E = −1282.37
H = −1218.95
G = −1240.76
N_{imag} = 0

N	-2.345989	-0.951601	-0.274184
O	-0.943945	-1.006609	0.075774
H	-2.740137	-0.327183	0.432656
H	-2.687921	-1.881105	-0.021237
H	1.518718	-0.019695	0.150301
C	-0.163168	-0.646674	-1.069589
H	-0.435347	0.365840	-1.401290
H	-0.376935	-1.338519	-1.897224
C	1.300531	-0.713635	-0.666996
H	1.577110	-1.722200	-0.345351
H	1.928657	-0.443299	-1.521771

P: HSO^- + C_2H_5Cl
E = −1112.45
H = −1059.75
G = −1081.95
N_{imag} = 0

H	2.026100	-1.955229	0.303027
O	-0.270106	-1.608384	0.218850
S	1.014635	-1.988182	1.207718
H	-0.100963	-2.277684	-2.429457
C	-0.912454	-2.714765	-0.466226
H	-1.913998	-2.326386	-0.684901
H	-1.012633	-3.560863	0.223911
C	-0.204900	-3.126671	-1.747148
H	0.786843	-3.542625	-1.547980
H	-0.791396	-3.900916	-2.254494

P: CH_3O^- + C_2H_5Cl
E = −1409.64
H = −1339.25
G = −1360.87
N_{imag} = 0

H	0.041309	-0.580129	2.794736
H	-0.708654	-1.525969	1.494512
O	1.992442	0.436335	1.283332
H	0.941345	-1.901797	2.027343
H	0.943207	-0.636965	-0.151219
H	0.042000	0.686144	0.616946
C	0.231943	-1.089099	1.845264
C	0.772359	-0.119107	0.808580
C	2.574060	1.356762	0.381004
H	2.820139	0.885752	-0.584734
H	3.497057	1.717964	0.842311
H	1.912572	2.217227	0.188470

P: CH_3OO^- + C_2H_5Cl
E = −1505.76
H = −1432.68
G = −1457.34
N_{imag} = 0

H	0.572945	-0.989804	2.737050
H	-0.782464	-1.549639	1.740689
O	1.743735	0.383868	0.817091
H	0.871710	-2.047433	1.341573
H	0.055680	-0.234691	-0.212561
H	-0.245587	0.827735	1.188736
C	0.260947	-1.220461	1.714552
C	0.374950	0.001055	0.813120

C	3.183837	1.953257	-0.042842
H	3.839430	1.165706	-0.439044
H	3.511086	2.238462	0.966391
H	3.225859	2.827844	-0.700795
O	1.825781	1.568727	-0.066717

P: CH_3HNO^- + C_2H_5Cl
E = −1646.50
H = −1565.12
G = −1589.18
N_{imag} = 0

N	-2.465122	-1.240998	-0.388107
O	-1.077094	-1.211037	0.024191
C	-3.112952	-0.057141	0.174658
H	-2.827917	-2.056604	0.108406
H	-4.189552	-0.160672	-0.002592
H	-2.939218	0.075409	1.253102
H	-2.762469	0.835211	-0.351313
H	1.431057	-0.369449	-0.226266
C	-0.239225	-1.432776	-1.115028
H	-0.449499	-0.675745	-1.883914
H	-0.454947	-2.419474	-1.551490
C	1.205397	-1.352986	-0.649186
H	1.418890	-2.110992	0.110492
H	1.875168	-1.522469	-1.498347

P: CH_3SO^- + C_2H_5Cl
E = −1484.23
H = −1412.59
G = −1437.67
N_{imag} = 0

S	1.015686	-2.053042	-0.795303
O	-0.101302	-1.670995	0.384326
C	2.529394	-1.294801	-0.169814
H	2.391622	-0.222889	-0.003112
H	2.906508	-1.780015	0.734555
H	3.262751	-1.434904	-0.972680
H	-1.118052	-1.189309	2.804662
C	-0.257025	-2.644357	1.445726
H	0.687564	-2.746252	1.996410
H	-0.502000	-3.620271	1.008119
C	-1.366509	-2.157948	2.361280
H	-2.309739	-2.057458	1.816777
H	-1.513367	-2.878653	3.172746

P: H_2N^- + C_2H_5Cl

E = −1170.05

H = −1109.66

G = −1129.10

N$_{imag}$ = 0

N	1.293259	1.350426	-0.058303
H	2.115220	2.556189	2.297218
H	0.851755	0.602701	-0.586206
H	2.209919	0.999063	0.207507
C	0.503332	1.648757	1.146328
H	0.381462	0.780883	1.818269
H	-0.504020	1.927019	0.813122
C	1.111595	2.806522	1.933537
H	1.194566	3.701624	1.309794
H	0.496027	3.046617	2.806782

P: H_2ON^- + C_2H_5Cl

E = −11282.92

H = −1219.57

G = −1240.84

$\boldsymbol{N}_{imag}$ = 0

N	0.614398	1.344554	0.353973
H	2.888476	1.494957	1.815856
H	-0.186135	0.748913	-1.328344
O	-0.216992	1.561351	-0.792740
H	0.156499	0.607710	0.893585
C	1.945531	0.885577	-0.044118
H	2.395388	1.683517	-0.644310
H	1.901546	-0.017624	-0.684703
C	2.799135	0.602835	1.189996
H	2.375112	-0.201870	1.801086
H	3.803329	0.291711	0.887615

P: H_2NHN^- + C_2H_5Cl

E = −1415.05

H = −1343.40

G = −1364.81

$\boldsymbol{N}_{imag}$ = 0

N	1.302027	0.746573	-0.035777
H	1.116887	1.280564	0.808300
H	0.135644	1.640306	-1.442838
N	0.112327	0.825652	-0.821925
H	0.090021	0.005823	-1.422500
H	3.507103	1.927493	1.141705
C	2.513024	1.261217	-0.681220
H	2.361653	2.279291	-1.092298
H	2.736027	0.609148	-1.535496

C	3.694088	1.269038	0.285619
H	3.893707	0.264123	0.668906
H	4.596053	1.631985	-0.216500

P: $HSHN^-$ + C_2H_5Cl

E = −1240.23

H = −1179.52

G = −1201.75

N_{imag} = 0

N	0.856552	1.581695	0.093211
H	-0.128331	1.071240	2.587906
H	2.666204	1.681043	-1.486791
S	1.691254	2.528615	-1.040894
H	0.341327	0.837986	-0.365887
C	1.595536	1.089895	1.261833
H	2.066302	1.958189	1.733574
H	2.410431	0.403842	0.971863
C	0.658790	0.393174	2.245812
H	0.181148	-0.484801	1.796089
H	1.221615	0.048140	3.118966

P: CH_3HN^- + C_2H_5Cl

E = −1531.50

H = −1453.11

G = −1474.80

N_{imag} = 0

1.N	0.674265	1.734729	-0.427916
H	1.511182	1.607936	0.133528
H	-0.112468	-0.267695	-0.184331
C	0.249988	0.441863	-0.952246
H	-0.560992	0.586374	-1.675236
H	1.084035	-0.027539	-1.483223
H	1.022806	3.590800	1.602775
C	-0.340531	2.411708	0.376900
H	-0.709169	1.782078	1.211318
H	-1.207746	2.595094	-0.271187
C	0.169218	3.739256	0.930816
H	0.488154	4.404509	0.122867
H	-0.615422	4.242848	1.504510

P: CH_3OHN^- + C_2H_5Cl

E = −1640.74

H = −1559.50

G = −1583.45

N_{imag} = 0

O	0.160601	3.506846	-0.983790
H	4.127945	2.478899	-0.963564

H	-0.777788	5.288183	-1.034853
C	0.255487	4.931005	-0.995956
H	0.726191	5.310105	-0.075086
H	0.796595	5.321861	-1.867666
N	1.408783	2.826042	-0.812375
H	1.884750	3.299827	-0.040580
C	2.253913	2.877842	-2.013078
H	2.532284	3.898511	-2.321359
H	1.668923	2.437276	-2.827173
C	3.520355	2.057861	-1.772686
H	3.274899	1.025123	-1.510327
H	4.138206	2.049640	-2.675753

P: CH_3HNHN^- + C_2H_5Cl
E = −1776.88
H = −1687.42
G = −1711.25
N_{imag} = 0

N	1.159045	1.057798	-0.884299
H	1.536316	0.860418	0.046033
H	-0.298990	-0.406049	-0.640464
C	-0.239279	0.648540	-0.930119
H	-0.906539	1.227052	-0.265386
H	-0.610467	0.751596	-1.953397
N	1.298373	2.441414	-1.199527
H	2.161167	4.551044	-2.683702
C	2.679925	2.769789	-1.551094
H	2.939444	2.167498	-2.427904
H	3.395472	2.489951	-0.753878
C	2.824276	4.256352	-1.864915
H	2.586258	4.880144	-0.995320
H	3.854297	4.482854	-2.156993
H	1.008097	3.021711	-0.407348

P: CH_3SHN^- + C_2H_5Cl
E = −1609.09
H = −1529.49
G = −1554.44
N_{imag} = 0

S	-0.244177	3.222073	-1.020741
H	4.177100	3.160353	-0.919357
H	-1.617845	5.084172	-1.489771
C	-0.528636	4.973626	-1.450944
H	-0.138233	5.645992	-0.680689
H	-0.118289	5.238915	-2.428790
N	1.420660	3.001730	-0.860403
H	1.795677	3.479775	-0.047306

C	2.298720	3.111797	-2.030486
H	2.376229	4.146831	-2.402385
H	1.840146	2.520190	-2.829347
C	3.696306	2.581331	-1.716183
H	3.657913	1.534997	-1.399687
H	4.334585	2.652850	-2.602896"

P: HS^- + C_2H_5Cl

E = −985.65

H = −936.11

G = −956.45

N_{imag} = 0

C	-0.643544	-5.486706	0.003150
C	-0.089547	-4.066867	0.075200
S	1.686199	-3.938822	0.494026
H	-0.483606	-6.024708	0.941424
H	-0.168768	-6.060148	-0.799014
H	-1.721573	-5.460171	-0.198624
H	-0.581197	-3.500265	0.873087
H	-0.269103	-3.522432	-0.856570
H	2.160952	-4.535544	-0.615429

P: HOS^- + C_2H_5Cl

E = −1124.24

H = −1070.50

G = −1092.80

N_{imag} = 0

C	-0.824587	-5.870431	-0.050714
C	-0.300186	-4.456150	-0.289101
S	1.243379	-4.028097	0.556239
H	-0.996214	-6.054569	1.013377
H	-0.116635	-6.619288	-0.415212
H	-1.771854	-6.016985	-0.583240
H	-0.992157	-3.702527	0.114373
H	-0.193120	-4.240773	-1.359050
H	2.751055	-4.559858	-0.904961
O	2.363028	-5.053760	-0.169940

P: H_2NS^- + C_2H_5Cl

E = −1246.70

H = −1185.10

G = −1207.58

N_{imag} = 0

C	-0.860400	-5.654937	0.872425
C	-0.181055	-4.306975	0.644014
S	1.590529	-4.358369	0.190066
H	-0.353454	-6.227107	1.654728

H	-0.870323	-6.262249	-0.038450
H	-1.904496	-5.511311	1.175767
H	-0.171849	-3.714669	1.568749
H	-0.716842	-3.711609	-0.105316
H	1.386182	-5.982181	-1.428822
N	1.698027	-5.019119	-1.369780
H	1.238921	-4.456319	-2.078825

P: HSS^- + C_2H_5Cl

E = −1071.08

H = −1019.65

G = −1042.90

N_{imag} = 0

C	-0.854174	-5.871004	-0.045976
C	-0.294065	-4.478412	-0.307730
S	1.270156	-4.068022	0.553102
H	-1.032553	-6.033909	1.020555
H	-0.170403	-6.646264	-0.401426
H	-1.806028	-5.997104	-0.575515
H	-0.971793	-3.702495	0.072887
H	-0.156077	-4.289275	-1.376601
H	3.075263	-4.433224	-1.330403
S	2.699216	-5.251630	-0.319177

P: CH_3S^- + C_2H_5Cl

E = −1352.58

H = −1284.03

G = −1306.97

N_{imag} = 0

C	-0.631132	-5.701445	0.724317
C	-0.184887	-4.258398	0.491438
S	1.598097	-3.993795	0.231205
H	-0.110192	-6.143080	1.578365
H	-0.445544	-6.333677	-0.148506
H	-1.708594	-5.732567	0.927982
H	-0.406869	-3.646375	1.372748
H	-0.727393	-3.808112	-0.348198
H	1.680783	-5.725824	-1.495425
C	1.844465	-4.646597	-1.439900
H	2.887707	-4.442435	-1.694680
H	1.203176	-4.135312	-2.164680

P: CH_3OS^- + C_2H_5Cl
E = −1481.48
H = −1409.69
G = −1434.70
N N_{imag} = 0

C	-0.866007	-5.819395	-0.130335
C	-0.366172	-4.381027	-0.246191
S	1.204799	-4.019731	0.585761
H	-0.991259	-6.112039	0.915735
H	-0.165784	-6.518307	-0.595382
H	-1.832630	-5.926063	-0.637043
H	-1.047218	-3.681351	0.259352
H	-0.311388	-4.056798	-1.291578
H	3.678654	-5.061379	-1.729648
O	2.309698	-4.966485	-0.239842
C	2.942773	-4.337432	-1.365986
H	3.451018	-3.412257	-1.071725
H	2.225639	-4.119278	-2.166746

P: CH_3HNS^- + C_2H_5Cl
E = −1606.62
H = −1526.94
G = −1552.05
N_{imag} = 0

C	-1.033356	-5.617392	1.145955
C	-0.377476	-4.355192	0.585559
S	1.429104	-4.418655	0.240026
H	-0.512777	-5.966078	2.042698
H	-1.035499	-6.435262	0.421532
H	-2.076452	-5.414645	1.420410
H	-0.449130	-3.535412	1.312695
H	-0.880455	-4.005398	-0.323628
H	1.932547	-7.073586	-0.808736
N	1.681045	-5.057679	-1.296115
H	1.366179	-4.441479	-2.036137
C	1.467479	-6.471438	-1.593219
H	1.962291	-6.706709	-2.543107
H	0.410712	-6.765763	-1.675639

P: CH_3SS^- + C_2H_5Cl
E = −1606.62
H = −1526.94
G = −1552.05
N_{imag} = 0

C	-1.033356	-5.617392	1.145955
C	-0.377476	-4.355192	0.585559
S	1.429104	-4.418655	0.240026

H	-0.512777	-5.966078	2.042698
H	-1.035499	-6.435262	0.421532
H	-2.076452	-5.414645	1.420410
H	-0.449130	-3.535412	1.312695
H	-0.880455	-4.005398	-0.323628
H	1.932547	-7.073586	-0.808736
N	1.681045	-5.057679	-1.296115
H	1.366179	-4.441479	-2.036137
C	1.467479	-6.471438	-1.593219
H	1.962291	-6.706709	-2.543107
H	0.410712	-6.765763	-1.675639

P: Cl^-

E = −87.76
H = −86.87
G = −97.79
N_{imag} = 0

Cl	0.000000	0.000000	0.000000

HOH

E = −324.38
H = −308.79
G = −322.65
N_{imag} = 0

H	-0.006268	-0.004870	-5.534813
O	-0.006176	-0.004798	-4.571236
H	-0.744627	-0.578479	-4.338726

HOOH

E = −411.25
H = −392.35
G = −408.55
N_{imag} = 0

H	-4.721571	-2.311439	-0.244475
O	-4.297515	-1.582368	0.233798
O	-2.916620	-2.050262	0.242564
H	-2.512921	-1.401434	-0.354234

H_2NOH

E = −552.64
H = −525.26
G = −542.00
N_{imag} = 0

N	-2.227638	-1.122837	-0.351120
O	-0.952472	-0.688388	0.188572
H	-2.894588	-0.472369	0.068050
H	-2.395055	-2.010546	0.125934
H	-0.436314	-0.551239	-0.614427

HSOH
E = −384.3
H = −367.85
G = −385.23
N_{imag} = 0

H	1.578644	-1.275797	-0.396905
O	-0.336504	-2.055245	0.641250
S	1.290751	-2.361616	0.362172
H	-0.824679	-2.503083	-0.062644

CH_3OH
E = −679.92
H = −645.76
G = −662.77
N_{imag} = 0

C	-0.294554	-1.832387	0.009966
H	0.419277	-0.996151	-0.019188
O	-1.648665	-1.390738	-0.056103
H	-0.096049	-2.437526	0.906690
H	-0.125577	-2.458239	-0.870218
H	-1.816974	-0.836545	0.712924

CH_3OOH
E = −773.28
H = −736.55
G = −755.80
N_{imag} = 0

C	-0.767258	-1.458850	-0.798697
H	0.002162	-0.678720	-0.744830
O	-1.148133	-1.906909	0.492046
H	-0.348156	-2.352472	-1.275054
H	-1.626324	-1.099272	-1.381426
O	-1.654930	-0.743004	1.219034
H	-2.588004	-0.993192	1.305981

CH_3HNOH
E = −917.00
H = −871.73
G = −890.81
N_{imag} = 0

N	-2.292526	-1.302354	-0.207203
O	-0.948594	-0.884251	0.156256
C	-3.192901	-0.177025	0.041048
H	-2.493808	-2.016938	0.492355
H	-4.216912	-0.550517	-0.074414
H	-3.083965	0.266670	1.041876
H	-3.022726	0.599186	-0.710139

H	-0.460517	-1.050443	-0.659080

CH_3SOH

E = −755.99
H = −720.66
G = −740.72
***N*imag** = 0

S	1.135857	-2.309006	-0.722891
O	-0.288877	-1.903810	0.071598
C	2.333259	-1.228947	0.079925
H	2.048639	-0.178177	-0.022617
H	2.485067	-1.485505	1.132616
H	3.271848	-1.395211	-0.462346
H	-0.378503	-2.517717	0.813193

H_2NH

E = −445.99
H = −422.46
G = −436.17
***N*imag** = 0

N	1.369275	1.374702	-0.023717
H	0.783977	1.557116	0.788782
H	0.803372	0.794830	-0.639744
H	2.114094	0.766950	0.310736

HOHNH

E = −555.88
H = −528.65
G = −545.37
***N*imag** = 0

N	0.773895	1.631090	-0.032367
H	1.173936	1.165944	0.784232
H	-0.040812	-0.065913	-0.586851
O	0.528310	0.595321	-1.008890
H	-0.143339	1.968658	0.264250

H_2NHNH

E = −688.13
H = −652.59
G = −669.08
***N*imag** = 0

N	1.222166	1.095316	0.116986
H	0.832606	1.941859	0.522555
H	0.327007	1.067264	-1.718723
N	0.235242	0.597701	-0.817957
H	0.467354	-0.376797	-0.989272
H	2.066539	1.386665	-0.375404

HSHNH

E = −512.61
H = −488.28
G = −505.88
N_{imag} = 0

N	0.823325	1.738555	0.142371
H	1.327938	1.275643	0.891924
H	-0.209222	-0.315936	-0.609011
S	0.539322	0.670431	-1.167364
H	-0.047565	2.105153	0.513739

CH_3HNH

E = −803.82
H = −761.72
G = −778.88
N_{imag} = 0

N	0.827468	1.587369	-0.104396
H	1.344113	1.084473	0.612711
H	-0.406499	-0.136411	-0.593204
C	0.250112	0.618556	-1.051857
H	-0.330777	1.154475	-1.809065
H	1.058260	0.092122	-1.569310
H	0.074502	2.055566	0.393500

CH_3OHNH

E = −915.15
H = −870.09
G = −889.21
N_{imag} = 0

O	0.962066	1.500391	-0.228161
H	1.708106	2.650597	-1.658496
H	0.177642	-0.330742	-0.592980
C	0.290709	0.614998	-1.130409
H	-0.701055	1.001466	-1.403031
H	0.883596	0.449655	-2.040595
N	1.188425	2.798694	-0.788395
H	0.271871	3.150265	-1.080585

CH_3HNHNH

E = −1050.68
H = −997.29
G = −1016.42
N_{imag} = 0

N	1.051670	1.493989	-0.240501
H	0.589602	1.564773	0.660993
H	0.149430	-0.362562	-0.580818
C	0.270404	0.595778	-1.096902

H	-0.730781	0.987878	-1.344726
H	0.810781	0.415152	-2.031850
N	1.148016	2.841585	-0.731323
H	1.851611	2.838314	-1.466131
H	0.273317	3.115367	-1.189220

CH_3SHNH

E = −882.75
H = −839.55
G = −859.81
N_{imag} = 0

S	1.090014	1.574775	-0.029039
H	1.849663	2.953704	-1.689353
H	0.075280	-0.467691	-0.643224
C	0.203985	0.480526	-1.175925
H	-0.780920	0.883279	-1.428038
H	0.786278	0.301972	-2.084153
N	1.297478	3.059359	-0.842558
H	0.407466	3.487082	-1.083521

HSH

E = −252.96
H = −241.31
G = −255.96
N_{imag} = 0

S	1.336296	0.810578	0.833436
H	1.437803	-0.423572	0.308248
H	1.316714	1.387338	-0.381427

HOSH

E = −388.29
H = −371.92
G = −389.35
N_{imag} = 0

H	-4.844667	-2.395246	0.033269
O	-4.306123	-1.591619	0.055683
S	-2.702241	-2.078637	-0.071501
H	-2.420212	-2.213368	1.245914

H_2NSH

E = −512.51
H = −488.08
G = −505.79
N_{imag} = 0

N	-2.321368	-1.012442	-0.293967
S	-0.811987	-0.413861	0.305779
H	-3.072339	-0.571924	0.229690

H	-2.367341	-2.010328	-0.108717
H	-0.345211	0.088599	-0.856844

HSSH

E = −335.11

H = −321.18

G = −339.19

$\boldsymbol{N}_{\mathbf{imag}}$ = 0

H	-5.034732	-2.565140	0.033038
S	-4.371680	-1.388402	0.048015
S	-2.440701	-2.104495	-0.068830
H	-2.178011	-2.219179	1.251140

CH_3SH

E = −618.95

H = −587.87

G = −606.00

$\boldsymbol{N}_{\mathbf{imag}}$ = 0

C	-0.254406	-1.900927	-0.037367
H	0.330902	-0.985881	-0.141075
S	-2.037782	-1.535971	0.032474
H	-0.006434	-2.375856	0.913998
H	-0.025478	-2.592036	-0.850148
H	-2.148822	-0.966210	-1.181159

CH_3OSH

E = −745.07

H = −710.71

G = −730.81

$\boldsymbol{N}_{\mathbf{imag}}$ = 0

C	-0.712971	-1.441582	-0.820350
H	0.253460	-0.928794	-0.784182
O	-1.226771	-1.676406	0.508285
H	-0.591845	-2.433420	-1.264227
H	-1.423195	-0.855270	-1.414192
S	-1.368389	-0.315334	1.467040
H	-2.556124	0.150357	1.005800

CH_3HNSH

E = −873.64

H = −831.29

G = −851.26

$\boldsymbol{N}_{\mathbf{imag}}$ = 0

N	-2.316579	-1.211061	-0.261222
S	-0.660561	-0.809292	-0.234023
C	-3.266557	-0.151162	0.091808
H	-2.479532	-2.044007	0.294867

H	-4.280514	-0.550357	-0.017263
H	-3.145290	0.221407	1.119937
H	-3.147310	0.687848	-0.598262
H	-0.458099	-0.439939	1.062835

CH_3SSH

E = −704.68
H = −671.73
G = −692.89
N_{imag} = 0

C	-0.716445	-1.464041	-0.791465
H	0.231334	-0.927646	-0.721185
S	-1.353264	-1.879375	0.859359
H	-0.552524	-2.428305	-1.283525
H	-1.446952	-0.886216	-1.360705
S	-1.515102	-0.073747	1.824050
H	-2.759613	0.283779	1.432324

H^+

E = 291.47
H = 292.36
G = 284.61
N_{imag} = 0

H	0.000000	0.000000	0.000000

Table S19. Cartesian coordinates (Å), energies (in kcal mol^{-1}), and number of imaginary vibrational frequencies (N_{imag}) of the S_N2 reaction between Nu:$^-$ + C_2H_5F, computed at ZORA-OLYP/QZ4P.

C_2H_5F
E = -911.12
H = -1075.90
G = -1099.24
***N*imag** = 0

F	0.316085	0.015950	0.000000
C	1.722173	0.034252	-0.000000
C	2.235888	1.459245	-0.000000
H	3.331747	1.453886	0.000000
H	1.897034	1.999191	0.888835
H	1.897035	1.999191	-0.888835
H	2.048939	-0.514681	0.892023
H	2.048939	-0.514681	-0.892023

TS: HO^- + C_2H_5F
E = -1272.72
H = -1075.90
G = -1099.24
***N*imag** = -441.942

C	-0.988709	-5.637426	0.053260
C	-0.797048	-4.157617	-0.123067
O	1.293993	-4.307240	-0.234236
H	-1.613202	-5.831770	0.931436
H	-0.023521	-6.135660	0.176101
H	-1.502446	-6.063843	-0.814883
H	-0.604503	-3.538562	0.735541
H	-0.708188	-3.734684	-1.107677
F	-2.574970	-3.703337	-0.081750
H	1.547094	-3.385693	-0.376634

TS: HOO^- + C_2H_5F
E = -1228.74
H = -1173.77
G = -1198.90
***N*imag** = -392.552

C	-0.865144	-5.795644	0.243142
C	-0.895720	-4.322659	-0.047315
F	0.912560	-4.042686	0.136534
H	-0.414433	-5.982512	1.222904
H	-0.259789	-6.319461	-0.503222
H	-1.877097	-6.219746	0.232653
H	-1.083144	-3.617902	0.746510

H	-0.862328	-3.956638	-1.059384
O	-2.927544	-4.353458	-0.303013
O	-3.376448	-3.044178	-0.780034
H	-3.467730	-2.563015	0.054977

TS: H_2NO^- + C_2H_5F
E = -1362.29
H = -1299.46
G = -1324.90
N_{imag} = -407.731

C	0.852567	1.593034	-0.048352
H	-0.013221	2.266131	-0.064548
H	1.467693	1.786044	-0.932933
H	1.462963	1.822116	0.830870
O	-1.574601	0.660065	-0.040447
H	-2.989754	-0.447724	0.782336
C	0.408828	0.159651	-0.020028
H	0.195606	-0.333289	0.913026
F	2.127723	-0.561190	0.005094
H	0.205370	-0.372989	-0.933258
N	-2.351799	-0.517586	-0.022517
H	-2.982489	-0.477024	-0.835056

TS: HSO^- + C_2H_5F
E = -1209.57
H = -1156.77
G = -1183.12
N_{imag} = -441.942

C	0.894420	1.628378	-0.062058
H	0.091331	2.356859	0.104477
H	1.316353	1.796471	-1.056961
H	1.689999	1.798993	0.667037
O	-1.480125	0.670189	0.105412
H	-2.587622	-1.161979	1.073497
C	0.374698	0.224887	0.064727
H	0.319571	-0.240129	1.035924
F	2.226739	-0.550327	0.047338
H	0.245651	-0.405910	-0.798776
S	-2.498970	-0.545204	-0.151807

TS: CH_3O^- + C_2H_5F
E = −1489.93
H = −1420.54
G = −1446.10
N_{imag} = −445.264

C	-0.97337	-5.627534	0.577049
C	-0.54767	-4.445413	-0.247315
O	1.43453	-4.799850	-0.025471
H	-1.59752	-5.299345	1.413866
H	-0.10066	-6.160200	0.966572
H	-1.57285	-6.315474	-0.027198
H	-0.36275	-3.491801	0.219093
H	-0.37722	-4.547379	-1.306232
F	-2.28011	-3.870683	-0.631269
C	2.16429	-3.856021	-0.684125
H	1.97317	-3.802770	-1.790888
H	3.26132	-4.035906	-0.589827
H	2.01024	-2.803544	-0.319166

TS: CH_3OO^- + C_2H_5F
E = −1590.27
H = −1517.47
G = −1545.21
N_{imag} = −416.806

C	-1.278798	-1.805005	-2.044221
C	-0.807438	-0.505200	-1.455373
O	0.379284	-1.313790	-0.029619
H	-2.034879	-2.274474	-1.406663
H	-0.443817	-2.503336	-2.151300
H	-1.722110	-1.621987	-3.027839
H	-1.270226	-0.079344	-0.579582
H	0.057410	-0.016601	-1.872495
F	-1.895143	0.637252	-2.469230
O	1.541556	-1.980413	-0.592233
C	2.660238	-1.187127	-0.301155
H	2.589023	-0.177816	-0.741362
H	3.531819	-1.707180	-0.732658
H	2.812259	-1.074264	0.788193

TS: CH_3HNO^- + C_2H_5F
E = −1728.48
H = −1647.65
G = −1675.33
N_{imag} = −416.552

F	1.201394	1.592603	0.087676
C	-0.039016	0.157917	-0.013217

N	-0.262961	-2.646817	0.097656
H	-0.199172	-4.415886	1.222992
H	-0.294329	-3.206971	-0.762073
H	0.367080	-0.203893	0.915123
O	-1.102846	-1.547542	-0.106171
C	-1.250921	1.042526	-0.047044
H	-1.360783	1.581858	0.898880
H	-1.152666	1.788863	-0.840743
H	-2.158801	0.452361	-0.221438
H	0.438457	-0.146368	-0.929140
C	-0.794771	-3.490513	1.166051
H	-0.705393	-2.961112	2.121600
H	-1.863592	-3.758356	1.032676

TS: CH_3SO^- + C_2H_5F

E = −1579.23

H = −1507.59

G = −1536.31

N_{imag} = −409.614

C	0.932183	1.621717	-0.225218
H	0.388024	2.425880	0.283786
H	0.763671	1.728354	-1.301783
H	2.001743	1.726288	-0.029032
O	-1.392704	0.773382	0.463037
C	-2.723629	-1.393647	1.442451
C	0.468648	0.283054	0.268055
H	0.545945	0.057806	1.318655
F	2.285348	-0.572598	0.183697
H	0.216666	-0.514482	-0.410800
S	-2.478169	-0.306245	-0.001956
H	-1.815998	-1.954319	1.692231
H	-3.514943	-2.102767	1.166042
H	-3.048946	-0.817562	2.318560

TS: H_2N^- + C_2H_5F

E = −1231.23

H = −1172.51

G = −1196.08

N_{imag} = −417.083

C	-0.850230	-5.792841	-0.053582
C	-0.871273	-4.316751	0.242477
F	0.851447	-4.062415	0.586889
H	-0.076677	-6.277159	0.553272
H	-0.609933	-5.983676	-1.105950
H	-1.820378	-6.245519	0.168209
H	-1.202319	-3.990493	1.214920

H	-0.873278	-3.596361	-0.556685
N	-3.169368	-4.177144	-0.094052
H	-3.247839	-4.358944	-1.104690
H	-3.301614	-3.156457	-0.056327

TS: HOHN⁻ + C_2H_5F

E = −1350.93
H = −1288.60
G = −1314.52
N_{imag} = −367.095

C	-0.869265	-5.767727	-0.060884
C	-0.853721	-4.293093	0.240085
F	0.851409	-4.080267	0.521266
H	-0.225067	-6.299680	0.648220
H	-0.480426	-5.968336	-1.065701
H	-1.880052	-6.187335	0.013729
H	-1.167620	-3.962596	1.217885
H	-0.902591	-3.568657	-0.556984
N	-3.151484	-4.162966	-0.044000
H	-3.200447	-4.446694	-1.034138
O	-3.514937	-2.711962	-0.214036
H	-4.006958	-2.567908	0.598518

TS: H_2NHN^- + C_2H_5F

E = −1478.15
H = −1408.05
G = −1433.79
N_{imag} = −376.367

C	-0.854435	-5.774120	-0.051126
C	-0.874747	-4.292884	0.219505
F	0.799246	-4.033766	0.528364
H	-0.278019	-6.288430	0.726396
H	-0.368757	-5.989877	-1.009652
H	-1.866974	-6.189930	-0.064866
H	-1.229702	-3.947002	1.178028
H	-0.925522	-3.595443	-0.603509
N	-3.181277	-4.127035	-0.115683
H	-3.237628	-4.386025	-1.110522
N	-3.577239	-2.724235	-0.140300
H	-3.868813	-2.511750	0.812948
H	-2.739978	-2.121863	-0.267287

TS: $HSHN^-$ + C_2H_5F
E = −1315.36
H = −1255.31
G = −1281.93
N_{imag} = −460.567

C	-0.848366	-5.778018	0.086483
C	-0.618580	-4.293588	-0.013107
F	1.239673	-4.420817	0.100770
H	-0.304921	-6.174524	0.948994
H	-0.462579	-6.288132	-0.801996
H	-1.909539	-6.023171	0.205613
H	-0.627641	-3.676405	0.868856
H	-0.494333	-3.814944	-0.968560
N	-2.691874	-3.785610	-0.119044
H	-3.062212	-4.272945	-0.941276
S	-2.991981	-2.138522	-0.418948
H	-3.915007	-1.824843	0.542843

TS: CH_3HN^- + C_2H_5F
E = −1592.73
H = −1516.14
G = −1542.01
N_{imag} = −418.951

C	-0.882250	-5.781283	0.008001
C	-0.877890	-4.277314	0.073790
F	0.874267	-4.029029	0.162011
H	-0.156953	-6.183972	0.723350
H	-0.584862	-6.131241	-0.987113
H	-1.872499	-6.181937	0.243915
H	-1.074986	-3.796964	1.018536
H	-0.963461	-3.682193	-0.820886
N	-3.160248	-4.124294	-0.053415
H	-3.266672	-4.521460	-0.993527
C	-3.497790	-2.727381	-0.161981
H	-3.404667	-2.242587	0.826238
H	-4.542317	-2.513577	-0.491789
H	-2.854582	-2.111280	-0.850297

TS: CH_3OHN^- + C_2H_5F
E = −1712.78
H = −1632.67
G = −1660.95
N_{imag} = −402.985

C	-0.874944	-5.771655	-0.067925
C	-0.884326	-4.298683	0.239312
F	0.869831	-4.092102	0.491668
H	-0.231463	-6.294010	0.647765

H	-0.468266	-5.960261	-1.067569
H	-1.878534	-6.209990	-0.009150
H	-1.140586	-3.969941	1.233004
H	-0.903983	-3.564083	-0.548358
N	-3.127535	-4.168933	-0.013144
H	-3.175774	-4.438865	-1.007665
O	-3.461196	-2.737414	-0.145108
H	-4.103495	-2.656242	1.837073
H	-5.390258	-2.928421	0.633686
C	-4.427671	-2.407870	0.811243
H	-4.588733	-1.319183	0.746581

TS: CH_3HNHN^- + C_2H_5F

E = −1842.58
H = −1754.50
G = −1782.50
N_{imag} = −417.106

C	-0.970625	-5.762530	-0.043892
C	-1.010291	-4.273143	0.170080
F	0.711351	-4.023768	0.495769
H	-0.429233	-6.240260	0.779681
H	-0.436041	-6.007416	-0.968252
H	-1.976925	-6.190214	-0.090764
H	-1.326496	-3.884570	1.124066
H	-0.997906	-3.599958	-0.672395
N	-3.247188	-4.109431	-0.192043
H	-3.282629	-4.410589	-1.174679
N	-3.602932	-2.712574	-0.266768
H	-4.167333	-2.726127	1.819871
H	-2.752077	-2.147752	-0.127956
C	-4.499139	-2.357478	0.828454
H	-4.596715	-1.260088	0.878920
H	-5.492885	-2.787521	0.643982

TS: CH_3SHN^- + C_2H_5F

E = −1684.84
H = −1605.93
G = −1635.35
N_{imag} = −459.317

C	-0.966636	-5.776085	0.364518
C	-0.718434	-4.334447	0.008624
F	1.146677	-4.533745	-0.075384
H	-0.504839	-6.015933	1.327339
H	-0.505484	-6.424710	-0.385906
H	-2.035216	-6.012984	0.411987
H	-0.567435	-3.595565	0.775835
H	-0.713082	-4.010829	-1.017786

N	-2.763740	-3.767117	-0.032996
H	-3.149923	-3.960366	0.896307
H	-4.467202	-1.175715	-2.060414
S	-3.088224	-2.146094	-0.353691
H	-5.263237	-2.633304	-1.389328
H	-3.916341	-2.804747	-2.550146
C	-4.306415	-2.207302	-1.718351

TS: HS^- + C_2H_5F

E = −1077.31

H = −1028.04

G = −1051.06

N_{imag} = −398.320

C	-1.063038	-5.712875	0.077126
C	-0.669973	-4.275621	-0.148327
S	1.716659	-4.487761	-0.202328
H	-1.643765	-5.793315	1.000220
H	-0.205455	-6.389427	0.146781
H	-1.705145	-6.050143	-0.741279
H	-0.569345	-3.602351	0.682646
H	-0.635048	-3.870383	-1.142564
F	-2.573539	-3.665971	-0.168972
H	1.939150	-3.173046	-0.393270

TS: HOS^- + C_2H_5F

E = −1212.11

H = −1157.75

G = −1184.53

N_{imag} = −404.196

C	-0.856251	-5.815406	0.197983
C	-0.974544	-4.322643	0.057326
F	0.843271	-3.883497	0.501332
H	0.085252	-6.049925	0.704288
H	-0.858657	-6.309367	-0.778556
H	-1.678641	-6.235213	0.790245
H	-1.278046	-3.727138	0.900911
H	-0.807537	-3.833560	-0.886070
S	-3.336269	-4.364042	-0.603398
O	-3.547653	-2.735504	-1.180314
H	-3.698922	-2.225696	-0.377217

TS: H_2NS^- + C_2H_5F
E = −1331.81
H = −1270.18
G = −1295.84
N_{imag} = −397.939

C	-0.853937	-5.810382	0.239879
C	-0.989035	-4.346053	-0.081517
F	0.912053	-4.010609	0.072808
H	-0.392319	-5.932512	1.224062
H	-0.196940	-6.291247	-0.490708
H	-1.811567	-6.345700	0.242640
H	-1.134982	-3.620368	0.698618
H	-0.923064	-3.994039	-1.095548
S	-3.403737	-4.301943	-0.377050
N	-3.654872	-2.591606	-0.776543
H	-4.311813	-2.206930	-0.101554
H	-4.126173	-2.547854	-1.677172

TS: HSS^- + C_2H_5F
E = −1166.74
H = −1114.72
G = −1142.31
N_{imag} = −397.056

C	-0.809990	-5.880418	0.205140
C	-0.989157	-4.393412	0.071345
F	0.894936	-3.920230	0.528008
H	0.167652	-6.070331	0.656555
H	-0.848854	-6.378062	-0.769166
H	-1.581359	-6.327254	0.844206
H	-1.240299	-3.799387	0.932975
H	-0.778244	-3.885075	-0.852661
S	-3.289746	-4.481581	-0.530171
S	-3.734848	-2.601026	-1.230144
H	-4.009886	-1.929151	-0.082109

TS: CH_3S^- + C_2H_5F
E = −1440.13
H = −1371.84
G = −1398.20
N_{imag} = −407.444

C	-1.193260	-5.804822	0.079243
C	-0.740469	-4.373600	-0.043763
S	1.623088	-4.733334	-0.495900
H	-1.627117	-5.970225	1.069670
H	-0.383404	-6.525938	-0.068893
H	-1.976334	-6.007395	-0.657152
H	-0.425270	-3.821606	0.823649

H	-0.779652	-3.866101	-0.991090
F	-2.514934	-3.652776	0.286131
C	2.106616	-2.986507	-0.626617
H	1.910231	-2.435549	0.304150
H	1.570941	-2.471908	-1.436978
H	3.180318	-2.905353	-0.837520

TS: CH_3OS^- + C_2H_5F

E = −1570.56

H = −1498.31

G = −1527.63

N_{imag} = −407.437

C	-0.840837	-5.844863	0.151712
C	-0.950049	-4.345841	0.111540
F	0.922701	-3.960344	0.486584
H	0.123143	-6.113159	0.594239
H	-0.896509	-6.277196	-0.851969
H	-1.635919	-6.295657	0.758587
H	-1.178872	-3.801937	1.010631
H	-0.802755	-3.796616	-0.801327
S	-3.319322	-4.346621	-0.449061
O	-3.580581	-2.723494	-0.961690
C	-4.038161	-1.864274	0.063403
H	-3.320918	-1.786282	0.894906
H	-5.005688	-2.195852	0.476523
H	-4.164699	-0.869739	-0.391397

TS: CH_3HNS^- + C_2H_5F

E = −1694.33

H = −1614.21

G = −1643.85

N_{imag} = −408.655

C	-0.884141	-5.803693	0.167350
C	-1.047378	-4.312199	0.072843
F	0.816617	-3.849632	0.404342
H	0.111870	-6.024614	0.562144
H	-0.983242	-6.280027	-0.813500
H	-1.630192	-6.250719	0.835145
H	-1.287120	-3.738217	0.950663
H	-0.935533	-3.792823	-0.861888
S	-3.428838	-4.370553	-0.431111
N	-3.709398	-2.681686	-0.809743
C	-4.630342	-1.993219	0.088488
H	-4.078361	-2.640792	-1.754584
H	-4.192936	-1.956012	1.091455
H	-5.626495	-2.468656	0.181694
H	-4.770705	-0.957959	-0.261919

TS: CH_3SS^- + C_2H_5F

E = −1535.14

H = −1464.11

G = −1494.32

N_{imag} = −408.655

C	-0.813177	-5.883243	0.162440
C	-0.994961	-4.391195	0.149737
F	0.915551	-3.949860	0.554740
H	0.165456	-6.106479	0.596585
H	-0.851580	-6.300420	-0.848647
H	-1.582440	-6.384048	0.763153
H	-1.200563	-3.869777	1.067924
H	-0.822672	-3.810505	-0.739004
S	-3.324891	-4.425160	-0.376493
S	-3.774299	-2.572747	-1.101076
C	-4.160742	-1.557074	0.366681
H	-3.289345	-1.461747	1.020540
H	-4.994496	-1.990812	0.928349
H	-4.446433	-0.563071	-0.000912

Table S20. Cartesian coordinates (Å), energies (in kcal mol^{-1}), and number of imaginary vibrational frequencies (N_{imag}) of the S_N2 reaction between Nu:$^-$ + CH_3F, computed at ZORA-OLYP/QZ4P.

CH_3F
E = -539.00
H = -512.56
G = -528.44
***N*imag** = 0

H	-0.844834	-5.138520	-0.015613
C	-0.807819	-4.069362	-0.137102
O	1.235157	-4.386871	-0.225490
H	-0.735305	-3.656975	-1.128920
H	1.570131	-3.489207	-0.347143
F	-2.572797	-3.857226	-0.053903
H	-0.622965	-3.450243	0.724020

TS: HO^- + CH_3F
E = -759.36
H = -725.68
G = -746.05
N_{imag} = -437.228

H	-0.844834	-5.138520	-0.015613
C	-0.807819	-4.069362	-0.137102
O	1.235157	-4.386871	-0.225490
H	-0.735305	-3.656975	-1.128920
H	1.570131	-3.489207	-0.347143
F	-2.572797	-3.857226	-0.053903
H	-0.622965	-3.450243	0.724020

TS: HOO^- + CH_3F
E = -860.12
H = -823.30
G = -846.05
N_{imag} = -406.878

H	-0.947937	-5.297029	0.145734
C	-0.872102	-4.238543	-0.062056
F	0.886041	-4.215696	0.163932
H	-3.510871	-2.624172	0.064085
O	-3.397880	-3.104480	-0.768772
O	-2.863534	-4.382037	-0.281867
H	-1.043577	-3.527225	0.730923
H	-0.824225	-3.893390	-1.082122

TS: H_2NO^- + CH_3F
E = -993.77

H = −949.06
G = −972.15
$\boldsymbol{N}_{\mathbf{imag}}$ = −426.63

H	0.611821	1.125492	-0.038016
H	-2.980766	-0.414929	-0.834753
N	-2.353136	-0.490670	-0.021531
H	0.225732	-0.466264	-0.938456
O	-1.511770	0.645302	-0.041602
H	-2.990211	-0.379335	0.780168
C	0.403054	0.065959	-0.018206
H	0.214208	-0.429315	0.920203
F	2.148110	-0.355164	0.000933

TS: HSO^- + CH_3F
E = −841.51
H = −806.81
G = −830.08
$\boldsymbol{N}_{\mathbf{imag}}$ = −434.379

H	0.681294	1.156679	0.002032
F	2.258634	-0.318499	0.055286
H	0.280084	-0.465977	-0.835146
S	-2.512239	-0.495482	-0.139942
O	-1.413177	0.657904	0.084137
H	-2.634980	-1.073803	1.101386
C	0.368680	0.126341	0.061030
H	0.320048	-0.361574	1.021094

TS: CH_3O^- + CH_3F
E = −1121.97
H = −1070.73
G = −1093.53
$\boldsymbol{N}_{\mathbf{imag}}$ = −456.345

H	-0.735816	-5.245658	0.301104
C	-0.546934	-4.370784	-0.298572
O	1.372551	-4.791373	-0.037450
H	2.008258	-3.821475	-1.785122
H	2.029448	-2.818803	-0.314668
H	3.245869	-4.095941	-0.538556
H	-0.388218	-3.416387	0.178324
H	-0.409020	-4.468898	-1.363860
F	-2.312869	-4.040238	-0.500276
C	2.158914	-3.878802	-0.670977

TS: CH_3OO^- + CH_3F
E = −1222.70
H = −1168.04
G = −1193.19

N_{imag} = −436.122

H	-0.959025	-1.388008	-1.821395
C	-0.779058	-0.415484	-1.395378
O	0.370983	-1.234315	-0.028993
H	2.637157	-0.171485	-0.662951
H	3.482945	-1.748010	-0.830308
H	2.825564	-1.234114	0.761635
H	-1.364729	-0.076718	-0.553211
H	0.039803	0.183099	-1.757331
F	-1.934008	0.377724	-2.564969
O	1.482243	-1.912213	-0.680493
C	2.650721	-1.224274	-0.330672

TS: CH_3HNO^- + CH_3F

E = −1360.07
H = −1297.37
G = −1322.62
N_{imag} = −442.401

F	0.983571	1.691798	0.055028
C	0.044531	0.118940	-0.005412
N	-0.253857	-2.648145	0.098806
H	-0.262208	-4.434217	1.200372
H	-0.325767	-3.196394	-0.766752
H	0.477783	-0.208552	0.924956
O	-1.051511	-1.509632	-0.081601
H	-0.891043	0.657168	-0.013140
C	-0.814751	-3.481219	1.161175
H	-0.693249	-2.969345	2.122639
H	-1.896020	-3.697305	1.033825
H	0.516247	-0.155538	-0.934283

TS: CH_3SO^- + CH_3F

E = −1210.94
H = −1157.39
G = −1183.50
N_{imag} = −431.067

H	0.755339	1.209320	0.093148
H	-3.570629	-2.053342	1.227292
H	-3.076596	-0.715399	2.304871
H	-1.879915	-1.940724	1.785467
O	-1.328525	0.697900	0.396139
C	-2.756336	-1.360211	1.476149
C	0.458775	0.187683	0.266116
H	0.511335	-0.209878	1.265409
F	2.361634	-0.254488	0.133485
H	0.307620	-0.481529	-0.565528
S	-2.438105	-0.379862	-0.029662

TS: H_2N^- + CH_3F
E = −863.82
H = −823.25
G = −844.18
N_{imag} = −408.154

H	-0.976153	-5.261868	0.001943
C	-0.865829	-4.219330	0.252289
F	0.830885	-4.244014	0.562417
N	-3.125407	-4.248505	-0.092356
H	-3.347065	-3.244213	-0.043407
H	-3.169578	-4.420612	-1.106533
H	-1.181342	-3.901684	1.232737
H	-0.835934	-3.493898	-0.544444

TS: $HOHN^-$ + CH_3F
E = −983.22
H = −939.05
G = −962.61
N_{imag} = −364.712

H	-0.985365	-5.241141	-0.018546
C	-0.837207	-4.203366	0.252383
F	0.830307	-4.266117	0.461529
H	-3.130800	-4.501032	-0.984313
O	-3.505189	-2.749886	-0.209937
H	-4.110325	-2.642751	0.528465
H	-1.114422	-3.901606	1.250664
H	-0.867984	-3.453540	-0.524850
N	-3.090238	-4.185926	-0.002675

TS: H_2NHN^- + CH_3F
E = −1110.39
H = −1058.46
G = −1081.78
N_{imag} = −345.295

H	-0.990385	-5.246076	-0.022134
C	-0.847665	-4.204937	0.237500
F	0.788279	-4.223078	0.481164
N	-3.598602	-2.776225	-0.159392
H	-3.914346	-2.553026	0.783693
H	-2.796288	-2.127534	-0.294712
H	-1.173194	-3.893191	1.219042
H	-0.882854	-3.471581	-0.557924
N	-3.120863	-4.152582	-0.087383
H	-3.159920	-4.446974	-1.072895

TS: $HSHN^-$ + CH_3F

E = −948.64
H = −906.71
G = −930.52
N_{imag} = −480.204

H	-0.892380	-5.230996	0.060573
C	-0.584652	-4.200801	-0.026244
F	1.158356	-4.654421	0.106494
H	-2.981006	-4.301426	-0.915956
S	-3.032982	-2.163185	-0.399671
H	-4.002808	-1.912622	0.537073
H	-0.547810	-3.575908	0.850277
H	-0.436365	-3.760602	-0.998162
N	-2.625740	-3.786253	-0.104510

TS: CH_3HN^- + CH_3F

E = −1225.55
H = −1167.13
G = −1190.38
N_{imag} = −406.589

H	-1.022036	-5.245881	-0.040888
C	-0.854273	-4.185792	0.075610
F	0.848981	-4.254707	0.162668
H	-4.572228	-2.596953	-0.463294
H	-2.917866	-2.123102	-0.890228
H	-3.404199	-2.243477	0.807583
H	-1.026377	-3.753736	1.048876
H	-0.904339	-3.543433	-0.791744
N	-3.097228	-4.129432	-0.038280
H	-3.192779	-4.549940	-0.968888
C	-3.507020	-2.755905	-0.166143

TS: CH_3OHN^- + CH_3F

E = −1345.31
H = −1283.39
G = −1309.30
N_{imag} = −414.938

H	-1.010212	-5.274306	0.003411
C	-0.838420	-4.230848	0.229491
F	0.880074	-4.363546	0.324582
H	-5.403819	-2.931807	0.452397
C	-4.481275	-2.417656	0.790214
H	-4.640421	-1.326843	0.737881
H	-0.992841	-3.889991	1.240409
H	-0.852039	-3.503784	-0.567993
N	-3.035201	-4.140911	0.122211
H	-3.147078	-4.443270	-0.859174
O	-3.377473	-2.711801	-0.019638

H	-4.312613	-2.702883	1.843295

TS: CH_3HNHN^- + CH_3F
E = −1475.08
H = −1405.12
G = −1430.66
N_{imag} = −416.984

H	-1.123814	-5.260495	0.006326
C	-0.970883	-4.201510	0.155562
F	0.725457	-4.279184	0.375322
C	-4.547442	-2.368467	0.802584
H	-4.674046	-1.272573	0.835628
H	-5.514958	-2.821554	0.547858
H	-1.206173	-3.782899	1.121349
H	-0.943806	-3.549579	-0.705319
N	-3.170231	-4.098153	-0.105322
H	-3.238972	-4.437522	-1.073147
N	-3.561466	-2.710645	-0.218918
H	-4.282196	-2.724049	1.818750
H	-2.737539	-2.128165	-0.016819

TS: CH_3SHN^- + CH_3F
E = −1318.19
H = −1257.37
G = −1283.94
N_{imag} = −479.555

H	-1.020165	-5.256945	0.174273
C	-0.679520	-4.249223	-0.001901
F	1.056942	-4.788465	0.033006
H	-5.273148	-2.681212	-1.348449
H	-3.962445	-2.821262	-2.554319
C	-4.339014	-2.232037	-1.710359
H	-0.518623	-3.579654	0.826057
H	-0.592724	-3.878295	-1.009396
N	-2.684612	-3.743425	-0.073821
H	-3.076771	-3.965482	0.846268
H	-4.538467	-1.205280	-2.047225
S	-3.075294	-2.136573	-0.387536

TS: HS^- + CH_3F
E = −711.02
H = −679.43
G = −701.00
N_{imag} = −423.342

H	-0.856478	-5.215027	-0.002984
C	-0.669454	-4.167479	-0.164123
S	1.642902	-4.563790	-0.189178

F	-2.595338	-3.871446	-0.138259
H	-0.657677	-3.779906	-1.166085
H	1.983427	-3.275021	-0.379477
H	-0.591144	-3.508105	0.680538

TS: HOS^- + CH_3F

E = −845.02
H = −808.87
G = −833.16
N_{imag} = −425.784

H	-0.947501	-5.299286	0.143650
C	-0.963537	-4.224504	0.046758
F	0.849520	-4.136797	0.527974
S	-3.259199	-4.381696	-0.570335
O	-3.585189	-2.779890	-1.178073
H	-3.797636	-2.276269	-0.385049
H	-1.228062	-3.616743	0.893817
H	-0.757698	-3.771444	-0.907844

TS: H_2NS^- + CH_3F

E = −965.01
H = −921.00
G = −945.78
N_{imag} = −427.597

H	-0.987597	-5.303823	0.099909
C	-0.973176	-4.243111	-0.096554
F	0.899604	-4.225630	0.107221
N	-3.664690	-2.618554	-0.781661
H	-4.332093	-2.256118	-0.104063
H	-4.157936	-2.623026	-1.671958
H	-1.085896	-3.545724	0.714556
H	-0.885438	-3.885937	-1.107527
S	-3.336505	-4.314165	-0.348801

TS: HSS^- + CH_3F

E = −799.888
H = −766.05
G = −790.95
N_{imag} = −417.982

H	-0.919092	-5.354639	0.178467
C	-0.977543	-4.284132	0.055051
F	0.893308	-4.223275	0.572410
S	-3.193044	-4.480326	-0.532544
H	-4.126097	-1.998696	-0.067477

S	-3.784517	-2.632513	-1.219569
H	-1.195482	-3.659978	0.903815
H	-0.705253	-3.837706	-0.885470

TS: CH_3S^- + CH_3F

E = −1073.75

H = −1023.12

G = −1047.69

***N*imag** = −428.999

H	-0.942388	-5.324591	0.016760
C	-0.746336	-4.267443	-0.044463
S	1.546075	-4.735725	-0.478621
H	1.946626	-2.446106	0.296324
H	3.193143	-2.989925	-0.839075
H	1.606662	-2.484957	-1.444698
H	-0.465662	-3.725326	0.841411
H	-0.821200	-3.765597	-0.993141
F	-2.567747	-3.890281	0.300264
C	2.116553	-3.016641	-0.62826

TS: CH_3OS^- + CH_3F

E = −1203.52

H = −1149.49

G = −1176.33

***N*imag** = −431.241

H	-0.924684	-5.331704	0.081809
C	-0.938901	-4.253334	0.115476
F	0.927800	-4.255275	0.482424
H	-3.342990	-1.738795	0.869884
H	-4.242169	-0.901019	-0.432792
H	-5.013321	-2.233370	0.488730
H	-1.114093	-3.751257	1.049334
H	-0.766141	-3.690249	-0.785753
S	-3.243533	-4.338860	-0.386912
O	-3.585358	-2.745230	-0.952618
C	-4.066554	-1.874507	0.050927

TS: CH_3HNS^- + CH_3F

E = −1327.29

H = −1265.94

G = −1291.34

***N*imag** = −431.855

H	-1.003074	-5.295920	0.095231
C	-1.034987	-4.218011	0.067556
F	0.835997	-4.172688	0.403801
H	-5.634678	-2.525144	0.183839
H	-4.855507	-0.983409	-0.292605

H	-4.231538	-1.920901	1.084094
H	-1.211956	-3.664448	0.972261
H	-0.885452	-3.699465	-0.863284
S	-3.348680	-4.331541	-0.377876
N	-3.705948	-2.663071	-0.799911
C	-4.663612	-2.002312	0.081359
H	-4.081165	-2.668503	-1.743488

TS: CH_3SS^- + CH_3F

E = −1168.19

H = −1115.35

G = −1143.13

$\boldsymbol{N}_{\mathbf{imag}}$ = −419.274

H	-0.926167	-5.370810	0.155647
C	-0.973028	-4.293124	0.148634
F	0.934143	-4.307946	0.558599
H	-5.029194	-2.028674	0.921215
H	-4.540389	-0.586893	-0.019211
H	-3.351624	-1.420833	1.020623
H	-1.121784	-3.762582	1.071925
H	-0.745760	-3.752533	-0.753980
S	-3.223415	-4.393717	-0.341115
S	-3.767889	-2.574978	-1.093573
C	-4.212095	-1.563088	0.360586

Table S21. Cartesian coordinates (Å), energies (in kcal mol^{-1}), and number of imaginary vibrational frequencies (N_{imag}) of the S_N2 reaction between Nu:$^-$ + CH_3Cl, computed at ZORA-OLYP/QZ4P.

CH_3Cl
E = −500.32
H = -474.64
G = -491.98
***N*imag** = 0

Cl	-0.081299	-0.002105	0.000065
C	1.706025	0.008092	-0.000009
H	2.046784	1.043665	-0.000153
H	2.055511	-0.506903	-0.895094
H	2.055609	-0.506633	0.895191

TS: HO^- + CH_3Cl
E = −730.92
H = −697.98
G = −720.79
***N*imag** = −109.053

C	-0.008064	0.310244	0.000068
Cl	0.118934	-1.680206	-0.000255
O	-0.272743	2.781545	0.001125
H	0.640032	3.100237	-0.001183
H	0.494119	0.594426	0.912215
H	-1.070579	0.501199	-0.000233
H	0.494634	0.594649	-0.911737

TS: HOO^- + CH_3Cl
E = −830.52
H = −794.54
G = −816.87
***N*imag** = −316.243

Cl	2.205357	-0.136743	-0.000572
C	0.174554	0.283227	0.012760
H	0.043473	0.338910	1.078824
H	-0.182798	-0.598876	-0.490871
H	0.207965	1.206413	-0.540787
O	-1.934208	0.708405	-0.004157
O	-2.515578	-0.557518	0.534721
H	-2.793809	-0.232795	1.409636

TS: H_2NO^- + CH_3Cl
E = −965.48
H = −921.51
G = −945.99

N_{imag} = −153.287

Cl	3.040007	-0.361166	0.022240
C	1.132182	0.305835	-0.072060
H	0.566847	-0.603931	0.059421
H	1.086804	0.744875	-1.057403
H	1.094642	1.002679	0.751088
O	-1.132673	1.088227	-0.160266
N	-1.367101	1.691596	-1.408527
H	-2.182251	1.223840	-1.838817
H	-1.714472	2.649943	-1.237313

TS: HSO^- + CH_3Cl

E = −815.72
H = −781.46
G = −806.27
N_{imag} = −354.987

Cl	3.132844	0.079224	-0.591940
C	1.051052	0.230697	0.179655
H	1.302180	0.995721	0.893646
H	1.016856	-0.796533	0.497511
H	0.633442	0.502766	-0.774491
O	-0.895979	0.406216	0.990535
S	-2.126615	0.139452	0.009531
H	-2.295702	-1.229146	0.026850

TS: CH_3O^- + CH_3Cl

E = −1093.52
H = −1043.10
G = −1067.87
N_{imag} = −279.961

C	0.300369	0.249153	0.000264
Cl	2.362405	-0.014232	0.000296
O	-1.952794	0.637001	0.000288
H	0.057130	-0.253729	-0.921209
H	0.256482	1.325286	0.000140
H	0.057112	-0.253520	0.921846
C	-2.662359	-0.515394	-0.000189
H	-2.487099	-1.189178	-0.890620
H	-2.487097	-1.189912	0.889685
H	-3.773722	-0.349248	-0.000119

TS: CH_3OO^- + CH_3Cl

E = −1194.99
H = −1140.88
G = −1167.95
N_{imag} = −276.095

Cl	2.914231	-0.068636	-0.034250

C	0.854166	0.379239	0.111941
H	0.688963	0.042186	1.120903
H	0.491071	-0.238893	-0.692500
H	0.906090	1.440810	-0.062507
O	-1.342492	0.944730	0.269957
O	-2.133817	0.086528	-0.590174
C	-2.824127	-0.813507	0.230136
H	-2.141953	-1.454130	0.818298
H	-3.496181	-0.293691	0.938812
H	-3.428736	-1.450789	-0.437901

TS: CH_3HNO^- + CH_3Cl
E = −1332.56
H = −1270.47
G = −1297.09
N_{imag} = −279.248

Cl	3.012778	-0.024163	0.007155
C	0.933077	0.275809	-0.149809
H	0.592300	-0.588540	0.396698
H	0.845725	0.257564	-1.223301
H	0.868671	1.233384	0.339127
O	-1.305109	0.628662	-0.317554
N	-2.007469	-0.373198	0.349504
H	-2.582208	0.086735	1.067696
C	-2.942781	-1.035318	-0.561410
H	-2.375210	-1.614895	-1.298917
H	-3.590161	-0.328061	-1.122460
H	-3.579871	-1.727741	0.013593

TS: CH_3SO^- + CH_3Cl
E = −1185.66
H = −1132.56
G = −1159.84
N_{imag} = −358.039

Cl	3.167988	0.205811	-0.668868
C	1.091457	0.232559	0.182387
H	1.279050	1.110016	0.775703
H	1.198302	-0.738691	0.631308
H	0.623417	0.328657	-0.782422
O	-0.841912	0.301536	1.046353
S	-2.096292	0.083211	0.091332
C	-2.406154	-1.716310	0.100562
H	-1.568269	-2.278590	-0.330118
H	-3.300585	-1.885385	-0.513665
H	-2.596614	-2.078499	1.119788

TS: H_2N^- + CH_3Cl

E = −833.53
H = −793.36
G = −815.23
***N*_{imag}** = −314.997

Cl	0.130374	-1.694355	0.000000
C	0.000000	0.329948	0.000000
H	-0.547725	0.511116	0.909018
N	-0.331869	2.693505	0.000000
H	0.079621	3.176727	0.813476
H	-0.547725	0.511116	-0.909018
H	1.042938	0.594123	0.000000
H	0.079621	3.176727	-0.813476

TS: H_2NHN^- + CH_3Cl
E = −1077.87
H = −1026.25
G = −1050.49
N_{imag} = −220.864

Cl	-2.247787	-0.140944	0.003331
C	-0.333015	0.322345	-0.005127
H	0.011734	-0.112505	-0.931008
N	2.657959	-0.643431	-0.106903
H	1.899256	-1.301504	-0.343638
H	3.012871	-0.971847	0.792502
N	2.071554	0.691604	0.131980
H	2.480817	1.247374	-0.631457
H	0.046084	-0.137835	0.893322
H	-0.346888	1.401090	0.018881

TS: $HSHN^-$ + CH_3Cl
E = −920.64
H = −879.31
G = −904.77
N_{imag} = −335.700

Cl	-2.247787	-0.140944	0.003331
C	-0.333015	0.322345	-0.005127
H	0.011734	-0.112505	-0.931008
N	2.657959	-0.643431	-0.106903
H	1.899256	-1.301504	-0.343638
H	3.012871	-0.971847	0.792502
N	2.071554	0.691604	0.131980
H	2.480817	1.247374	-0.631457
H	0.046084	-0.137835	0.893322
H	-0.346888	1.401090	0.018881

TS: CH_3HNHN^- + CH_3Cl
E = −1445.13
H = −1375.66

G = −1403.00
$\boldsymbol{N}_{\mathbf{imag}}$ = −132.054

Cl	3.068981	-0.262332	0.285721
C	1.158265	0.269208	0.004858
H	0.609850	-0.432380	0.617161
H	1.015124	0.158417	-1.059638
H	1.139287	1.293116	0.350792
N	-1.359367	0.926945	-0.424861
N	-2.058134	-0.334812	-0.265451
C	-3.492582	-0.220412	-0.567131
H	-1.429390	1.336995	0.516787
H	-3.700646	0.346436	-1.499024
H	-3.999157	0.305094	0.252089
H	-3.934926	-1.229871	-0.647523
H	-1.675717	-0.931506	-0.998889

TS: CH_3SHN^- + CH_3Cl

E = −1290.39
H = −1230.18
G = −1258.37
$\boldsymbol{N}_{\mathbf{imag}}$ = −346.747

Cl	3.180789	0.189338	-0.297036
C	1.065255	0.299683	-0.028954
H	1.028075	-0.138701	0.953355
H	0.771568	-0.310167	-0.866213
H	0.969773	1.367902	-0.120375
N	-1.247796	0.520668	0.201393
S	-2.155860	-0.891595	0.112426
C	-3.589177	-0.452648	-0.946338
H	-1.398233	0.897517	1.143435
H	-3.242087	-0.023521	-1.893870
H	-4.255755	0.266471	-0.451658
H	-4.144167	-1.379009	-1.150747

TS: HS^- + CH_3Cl

E = −685.50
H = −655.25
G = −678.14
$\boldsymbol{N}_{\mathbf{imag}}$ = −338.575

C	0.002108	0.153585	0.000000
Cl	-0.265252	2.364435	-0.000001
S	0.332462	-2.432591	0.000001
H	-0.992881	-2.673451	-0.000002
H	-0.512011	-0.008530	0.929956
H	1.077740	0.181906	-0.000001
H	-0.512012	-0.008531	-0.929955

TS: HOS^- + CH_3Cl
E = −819.20
H = −783.32
G = −809.05
N$_{imag}$ = −297.838

Cl	3.150648	-0.163500	-0.070076
C	1.032468	0.297380	0.034516
H	0.957156	0.365213	1.105811
H	0.661006	-0.589974	-0.451191
H	1.059441	1.209648	-0.537851
S	-1.598784	0.800877	0.143199
O	-2.247241	-0.797329	-0.157725
H	-2.308523	-1.178139	0.724366

TS: H_2NS^- + CH_3Cl
E = −932.42
H = −895.68
G = −921.49
$\boldsymbol{N_{imag}}$ = −294.957

Cl	3.150648	-0.163500	-0.070076
C	1.032468	0.297380	0.034516
H	0.957156	0.365213	1.105811
H	0.661006	-0.589974	-0.451191
H	1.059441	1.209648	-0.537851
S	-1.598784	0.800877	0.143199
O	-2.247241	-0.797329	-0.157725
H	-2.308523	-1.178139	0.724366

TS: HSS^- + CH_3Cl
E = −
H = −
G = −
$\boldsymbol{N_{imag}}$ = −

Cl	3.252742	-0.127610	-0.013648
C	1.067850	0.336604	-0.005187
H	0.956309	0.225733	1.058557
H	0.789559	-0.475911	-0.653182
H	1.196764	1.321069	-0.422260
S	-1.434672	0.928311	-0.012497
S	-2.444154	-0.866768	-0.044276
H	-2.525920	-1.173850	1.276589

TS: CH_3S^- + CH_3Cl
E = −1047.96
H = −997.66
G = −1024.00

N_{imag} = −308.232

C	0.098580	0.327343	-0.050083
Cl	0.003569	2.473036	-0.363447
S	0.199958	-2.312735	0.324214
C	-1.560847	-2.670775	0.023675
H	-0.623744	0.271096	0.745795
H	1.142359	0.270508	0.209107
H	-0.194279	0.028855	-1.041666
H	-2.215263	-2.187183	0.763414
H	-1.746913	-3.751675	0.082294
H	-1.886614	-2.338546	-0.972686

TS: CH_3OS^- + CH_3Cl

E = −1178.13
H = −1124.37
G = −1152.57
N_{imag} = −232.267

Cl	3.233212	-0.199256	-0.333029
C	1.109579	0.300142	-0.031037
H	1.150078	0.333438	1.043004
H	0.704813	-0.574132	-0.512755
H	1.127774	1.224036	-0.583799
S	-1.446501	0.889728	0.271114
O	-2.198793	-0.648250	0.033056
C	-2.462626	-1.340898	1.234175
H	-1.543520	-1.554895	1.803575
H	-2.938103	-2.293775	0.953218
H	-3.146282	-0.779242	1.893261

TS: CH_3HNS^- + CH_3Cl

E = −1301.97
H = −1240.30
G = −1268.38
N_{imag} = −316.688

Cl	3.084119	-0.189031	0.066720
C	0.968204	0.321207	-0.161000
H	0.563632	-0.520080	0.376936
H	0.950906	0.300903	-1.236975
H	1.017077	1.273556	0.338240
S	-1.610188	0.874006	-0.375097
N	-2.277768	-0.531801	0.452983
H	-2.807634	-0.181813	1.245732
C	-3.159680	-1.353670	-0.369554
H	-2.577963	-1.798906	-1.183751
H	-4.003201	-0.804469	-0.832021
H	-3.571076	-2.170877	0.245357

TS: CH_3SS^- + CH_3Cl
E = −1143.45
H = −1090.86
G = −1119.97
N_{imag} = −340.061

Cl	3.275108	-0.267077	-0.223632
C	1.123128	0.337335	-0.010485
H	1.192300	0.501427	1.049930
H	0.725886	-0.592607	-0.379087
H	1.210747	1.173541	-0.682373
S	-1.336520	1.062162	0.203664
S	-2.468843	-0.632329	0.069781
C	-2.626365	-1.239366	1.783663
H	-1.647814	-1.494800	2.201910
H	-3.251561	-2.141112	1.748231
H	-3.107122	-0.487655	2.418511

Table S22. Cartesian coordinates (Å), energies (in kcal mol^{-1}), and number of imaginary vibrational frequencies (N_{imag}) of the S_N2 reaction between Nu:$^-$ + C_3H_7F, computed at ZORA-OLYP/QZ4P.

C_3H_7F
E = -1282.39
H = -1219.76
G = -1240.71
***N*imag** = 0

H	0.172271	-0.247596	-0.840212
C	-0.407031	1.611483	0.088958
C	-0.004763	-0.620889	1.277453
F	-1.763056	-0.309213	-0.300845
H	-1.036374	1.971946	0.908406
H	-0.623694	-0.317394	2.127349
H	-0.764421	2.053474	-0.845145
H	-0.087482	-1.704365	1.155545
H	0.616426	1.960193	0.261384
H	1.038605	-0.382513	1.508414
C	-0.438599	0.093559	0.007361

TS: HO$^-$ + C_3H_7F
E = -1465.07
H = -1425.34
G = -1450.19
***N*imag** = -434.191

H	-2.476249	0.612167	-1.325275
O	-2.408309	-0.097375	-0.672839
C	-0.459344	-1.303856	0.628267
C	-0.474098	1.289704	0.635153
F	1.558576	0.014544	-0.138488
H	0.289855	-1.345003	1.425870
H	0.302101	1.355921	1.406273
H	-0.293345	-2.153976	-0.038335
H	-0.353574	2.148801	-0.032105
H	-1.461860	-1.394846	1.056831
H	-1.464551	1.346680	1.095043
C	-0.329327	-0.005747	-0.119249
H	-0.253483	-0.012125	-1.189490

TS: HOO$^-$ + C_3H_7F
E = -1595.90
H = -1523.01
G = -1550.34
***N*imag** = -376.884

C	0.420042	-0.142432	1.621786

C	-0.109242	1.591787	-0.236589
H	1.393983	0.340183	1.753414
H	0.562241	-1.219450	1.741514
H	-0.270479	0.205729	2.397088
H	0.921748	1.902411	-0.449424
H	-0.533819	2.263123	0.517326
H	-0.698953	1.697614	-1.150660
O	1.695409	-0.189919	-0.732114
O	1.963752	-1.624606	-0.821833
H	1.536926	-1.837822	-1.663557
C	-0.141907	0.176025	0.262778
H	-0.454581	-0.621380	-0.386836
F	-1.968089	0.234048	0.785361

TS: H_2NO^- + C_3H_7F
E = −1729.85
H = −1649.10
G = −1676.62
$\boldsymbol{N}_{\mathbf{imag}}$ = −384.040

F	-2.093312	0.006068	-0.065587
C	-0.175437	0.071616	-0.192749
N	1.901514	-0.236058	-2.191709
H	2.343132	0.500028	-2.759094
H	2.589507	-1.002585	-2.203923
H	-0.349486	0.127175	-1.251752
O	1.818936	0.243446	-0.873559
C	0.059972	-1.262653	0.461253
H	0.055653	-2.066456	-0.280601
H	1.031113	-1.272853	0.968775
H	-0.734250	-1.465576	1.187707
C	-0.044383	1.318597	0.633656
H	-0.356212	2.198156	0.064034
H	-0.680303	1.249263	1.522610
H	0.997416	1.462621	0.944823

TS: HSO^- + C_3H_7F
E = −1576.63
H = −1505.99
G = −1533.56
$\boldsymbol{N}_{\mathbf{imag}}$ = −371.892

C	0.446638	-0.007880	-0.124760
H	0.737655	-0.027790	-1.158824
O	-1.394586	-0.025730	-0.935810
H	-3.008241	1.243289	0.209247
S	-2.727448	-0.077135	-0.058474
F	2.573617	0.004846	-0.105488
C	0.362076	1.298760	0.597799

H	0.393827	2.139803	-0.099498
H	-0.564837	1.358939	1.183484
H	1.214372	1.385413	1.277240
C	0.375603	-1.282059	0.654755
H	0.453023	-2.151660	-0.002396
H	1.207625	-1.314349	1.363562
H	-0.568992	-1.341249	1.211118

TS: CH_3O^- + C_3H_7F

E = −1856.60

H = −1769.29

G = −1796.81

N_{imag} = −425.591

O	1.998295	0.427616	-0.065050
C	-0.318478	1.612091	0.071445
C	0.006572	-0.590513	1.398981
F	-1.903999	-0.340918	-0.262477
H	-1.236804	1.828880	0.625743
H	-0.956700	-0.504317	1.911382
H	-0.445490	1.978884	-0.950236
H	0.215072	-1.654361	1.257657
H	0.521596	2.145795	0.527461
H	0.796747	-0.163626	2.025168
C	-0.058734	0.131788	0.080037
H	0.096787	-0.397676	-0.842034
C	2.659064	-0.639846	-0.595358
H	2.852485	-1.485217	0.120948
H	2.140545	-1.118684	-1.468716
H	3.666585	-0.357336	-0.986027

TS: CH_3OO^- + C_3H_7F

E = −1958.34

H = −1867.61

G = −1897.19

N_{imag} = −389.126

C	0.503123	-0.049008	1.695342
C	-0.203522	1.498472	-0.264803
H	1.479671	0.445610	1.723806
H	0.655125	-1.107907	1.919559
H	-0.148507	0.377384	2.464259
H	0.798976	1.847794	-0.543593
H	-0.637392	2.202030	0.452847
H	-0.831363	1.498082	-1.159041
O	1.618411	-0.268843	-0.713950
O	1.879127	-1.692586	-0.729801
H	1.886041	-3.226122	-2.035162
C	-0.135495	0.128273	0.344898

H	-0.503285	-0.726542	-0.190287
F	-1.964928	0.187964	1.007753
C	1.680785	-2.143044	-2.043636
H	0.648810	-1.973985	-2.395295
H	2.369820	-1.651599	-2.755007

TS: CH_3HNO^- + C_3H_7F

E = −2096.02
H = −1997.29
G = −2026.86
N_{imag} = −390.297

F	-2.076629	-0.057042	-0.197525
C	-0.121089	-0.134981	-0.202813
N	2.073042	0.055233	-2.060014
H	2.728692	0.845550	-2.044407
H	2.969731	-0.621714	-3.831146
H	-0.263754	-0.262246	-1.260057
O	1.883586	-0.349246	-0.741495
C	-0.065752	-1.318834	0.718535
H	-0.352899	-2.234473	0.195212
H	0.950051	-1.450314	1.110948
H	-0.759009	-1.175852	1.553625
C	0.066989	1.243573	0.368071
H	0.137260	1.989781	-0.428505
H	-0.788693	1.499785	1.000846
H	0.987153	1.289528	0.961808
C	2.711870	-1.009006	-2.832463
H	3.628063	-1.415558	-2.356170
H	2.006791	-1.839257	-2.952959

TS: CH_3SO^- + C_3H_7F

E = −1947.02
H = −1857.49
G = −1888.03
N_{imag} = −376.368

C	0.449574	-0.023116	-0.164492
H	0.743706	-0.152123	-1.190813
O	-1.452638	0.110714	-0.799654
H	-0.912391	-1.994039	-2.892777
S	-1.683161	0.278116	-2.368873
F	2.517733	-0.263380	-0.052079
C	0.488732	1.344434	0.452574
H	0.580065	2.119087	-0.313299
H	-0.427318	1.532683	1.025063
H	1.355541	1.413158	1.115499
C	0.217186	-1.212894	0.720139
H	0.308992	-2.146445	0.159645

H	0.964097	-1.224733	1.518833
H	-0.786359	-1.170258	1.160860
C	-1.839083	-1.421893	-3.015192
H	-2.663089	-1.955724	-2.524180
H	-2.060334	-1.328017	-4.086472

TS: H_2N^- + C_3H_7F
E = −1598.67
H = −1521.99
G = −1547.22
N_{imag} = −421.710

H	-2.594325	-0.832557	-1.052232
N	-2.500172	-0.026280	-0.418175
C	-0.261583	-1.306605	0.746417
C	-0.263389	1.284238	0.715840
F	1.678266	-0.021155	-0.215497
H	0.560996	-1.366570	1.468904
H	0.560381	1.363315	1.435126
H	-0.182560	-2.171851	0.080997
H	-0.187829	2.133664	0.029955
H	-1.217346	-1.362149	1.275775
H	-1.218335	1.349965	1.245508
C	-0.154633	-0.020070	-0.027639
H	-0.233573	-0.032780	-1.097890
H	-2.596185	0.763592	-1.072298

TS: $HOHN^-$ + C_3H_7F
E = −1717.98
H = −1637.63
G = −1665.18
N_{imag} = −367.351

O	-3.249330	-0.710828	-0.322512
N	-2.213952	-0.008150	-1.149342
C	-0.112796	-1.372090	0.354551
C	-0.549914	1.118829	0.866745
F	1.727558	0.326391	0.114367
H	0.551161	-1.511751	1.217242
H	0.148748	1.201552	1.708176
H	0.233036	-2.042643	-0.438255
H	-0.612148	2.094367	0.377355
H	-1.128631	-1.663213	0.644354
H	-1.541600	0.867959	1.259793
C	-0.065240	0.063705	-0.088900
H	0.042540	0.305758	-1.131724
H	-1.968692	-0.788036	-1.776585
H	-3.824414	0.029942	-0.114153

TS: H_2NHN^- + C_3H_7F

E = −1845.03

H = −1757.02

G = −1784.68

N_{imag} = −371.919

N	-3.272829	-0.797839	-1.338970
N	-2.733958	0.023508	-0.266238
C	-0.449368	-1.318319	0.799147
C	-0.298438	1.249230	0.551864
F	1.411219	-0.235956	-0.488522
H	0.476137	-1.449084	1.373025
H	0.563291	1.324222	1.226713
H	-0.604835	-2.227684	0.210906
H	-0.196772	2.038192	-0.199614
H	-1.292462	-1.208258	1.488096
H	-1.211947	1.431501	1.129780
C	-0.325766	-0.110428	-0.092326
H	-0.576476	-0.199046	-1.136428
H	-2.794614	0.968305	-0.669425
H	-3.450861	-1.707613	-0.915144
H	-2.537760	-1.004172	-2.044688

TS: $HSHN^-$ + C_3H_7F

E = −1682.00

H = −1604.05

G = −1632.44

N_{imag} = −429.652

S	-2.771379	-1.132461	-1.805507
N	-2.327949	-0.151338	-0.489550
C	-0.206446	-1.328122	0.784943
C	-0.298510	1.265994	0.668961
F	1.782334	0.061033	-0.101570
H	0.752279	-1.475408	1.290718
H	0.479094	1.365509	1.432348
H	-0.396341	-2.191947	0.143053
H	-0.177495	2.094574	-0.034595
H	-1.008224	-1.277396	1.528867
H	-1.278937	1.351543	1.152677
C	-0.147926	-0.061192	-0.022562
H	-0.055520	-0.112033	-1.090748
H	-2.546405	0.800260	-0.799158
H	-3.777751	-1.895761	-1.277061

TS: CH_3HN^- + C_3H_7F
E = −1959.58
H = −1864.98
G = −1892.48
N_{imag} = −407.323

H	-1.166986	-1.535432	-2.435216
N	-2.211688	-0.782020	-0.669286
C	0.115325	-1.094250	0.929229
C	-0.851623	1.289727	0.661451
F	1.546378	0.810694	0.107135
H	0.806562	-0.811019	1.731411
H	-0.261057	1.672484	1.501614
H	0.582559	-1.901755	0.358726
H	-0.951797	2.100677	-0.066461
H	-0.815052	-1.470742	1.364940
H	-1.848653	1.014839	1.022089
C	-0.154261	0.101744	0.055375
H	-0.040252	0.026570	-1.010909
H	-2.508617	0.072416	-1.152704
C	-1.987501	-1.765464	-1.700721
H	-1.706793	-2.731591	-1.246704
H	-2.874198	-1.990743	-2.339136

TS: CH_3OHN^- + C_3H_7F
E = −2079.55
H = −1981.47
G = −2011.78
N_{imag} = −390.417

O	-2.826150	-0.881972	-1.610084
N	-2.552804	-0.047232	-0.425560
C	-0.330306	-1.347809	0.704733
C	-0.366323	1.244795	0.690447
F	1.588598	0.003876	-0.257198
H	0.597356	-1.495357	1.268877
H	0.402962	1.288755	1.469478
H	-0.453989	-2.195862	0.025854
H	-0.208788	2.099292	0.025771
H	-1.177203	-1.341255	1.397600
H	-1.349019	1.345709	1.168458
C	-0.250112	-0.053278	-0.060892
H	-0.319022	-0.067163	-1.133316
H	-2.577252	0.881928	-0.873430
H	-3.700952	-2.368896	-0.436077
C	-3.889852	-1.748619	-1.328867
H	-4.840913	-1.205317	-1.160153
H	-4.004870	-2.411635	-2.202225

TS: CH_3HNHN^- + C_3H_7F
E = −2208.37
H = −2101.96
G = −2131.75
N$_{imag}$ = -404.749

N	-3.120436	-0.886730	-1.226128
N	-2.690308	0.043239	-0.197263
C	-0.400663	-1.299396	0.758070
C	-0.318410	1.278590	0.556337
F	1.444519	-0.151666	-0.541028
H	0.537493	-1.360807	1.319995
H	0.572697	1.331140	1.191275
H	-0.477967	-2.207448	0.152571
H	-0.241114	2.079404	-0.185006
H	-1.241037	-1.271617	1.459969
H	-1.204206	1.456015	1.178489
C	-0.384820	-0.068671	-0.108579
H	-0.565240	-0.151428	-1.164782
H	-2.667697	0.933079	-0.711830
H	-5.153846	-0.879656	-0.487105
H	-2.915903	-1.803187	-0.832178
C	-4.571635	-0.855113	-1.432308
H	-4.847970	0.066469	-1.960053
H	-4.871354	-1.707029	-2.065732

TS: CH_3SHN^- + C_3H_7F
E = −2051.53
H = −1954.69
G = −1985.36
N$_{imag}$ = -426.594

S	-2.949901	-1.867678	-0.763390
N	-1.888189	-0.665960	-1.248366
C	0.492020	-1.333382	0.127610
C	-0.998642	0.566770	1.056585
F	1.440499	1.034764	0.402683
H	1.273116	-1.244530	0.889560
H	-0.379596	0.675632	1.952516
H	0.963177	-1.678652	-0.797134
H	-1.395341	1.550137	0.793603
H	-0.233148	-2.087051	0.451180
H	-1.838289	-0.100009	1.285863
C	-0.164501	0.008369	-0.057565
H	-0.046179	0.555056	-0.973905
H	-1.474553	-0.975040	-2.132127
H	-4.625299	-0.149526	-0.316360
C	-4.602859	-1.085183	-0.885434
H	-4.871534	-0.876852	-1.928930

H	-5.330049	-1.787381	-0.455334

TS: HS^- + C_3H_7F
E = −1444.07
H = −1376.32
G = −1402.42
N_{imag} = −383.973

H	2.431740	-1.142916	-1.066879
C	-0.060830	1.330171	0.738845
C	-0.069699	-1.260935	0.872701
F	-2.072415	-0.027429	-0.267443
H	-1.045061	1.459080	1.199985
H	-1.030184	-1.302364	1.397717
H	0.095247	2.145966	0.029121
H	0.008268	-2.151465	0.244007
H	0.709365	1.394315	1.515332
H	0.746843	-1.280659	1.600563
C	-0.037247	-0.004573	0.046370
H	-0.103924	-0.052807	-1.022266
S	2.423158	0.052113	-0.446061

TS: HOS^- + C_3H_7F
E = −1578.41
H = −1506.07
G = −1534.78
N_{imag} = −376.289

O	2.831306	-1.449825	-1.123710
C	-0.056989	1.330751	0.715976
C	-0.066856	-1.264522	0.917919
F	-2.019700	0.001404	-0.281771
H	-0.971693	1.438236	1.308689
H	-1.090864	-1.437486	1.266756
H	-0.046127	2.126618	-0.032254
H	0.256449	-2.139380	0.348425
H	0.803388	1.466491	1.384179
H	0.596569	-1.160260	1.782402
C	-0.055572	-0.025043	0.066333
H	-0.041224	-0.109063	-1.003506
S	2.465984	0.039013	-0.298263
H	2.711322	-1.227805	-2.053049

TS: H_2NS^- + C_3H_7F
E = −1699.18
H = −1619.08
G = −1648.03
$\boldsymbol{N_{imag}}$ = −373.481

N	3.009496	-1.186369	-1.334991
C	-0.079244	1.344878	0.749033
C	-0.093610	-1.250134	0.743708
F	-1.968098	0.067101	-0.463867
H	-1.041492	1.426994	1.266720
H	-1.103101	-1.382799	1.148970
H	0.016888	2.199428	0.075360
H	0.112241	-2.101515	0.088556
H	0.721743	1.401893	1.496500
H	0.629858	-1.264990	1.565230
C	-0.043574	0.049396	-0.011567
H	0.049533	0.054341	-1.080770
S	2.533831	0.145293	-0.274567
H	2.502397	-1.093096	-2.213978
H	2.686093	-2.059583	-0.920590

TS: HSS^- + C_3H_7F
E = −1533.10
H = −1463.09
G = −1492.50
$\boldsymbol{N_{imag}}$ = −364.759

S	2.818457	-1.741966	-1.476000
C	-0.041620	1.310742	0.682051
C	-0.140024	-1.270950	0.956990
F	-2.117077	0.036769	-0.238152
H	-0.961771	1.455105	1.256811
H	-1.170545	-1.372873	1.312379
H	0.002366	2.087265	-0.084774
H	0.127356	-2.179319	0.411990
H	0.814072	1.438567	1.358126
H	0.531213	-1.175358	1.817206
C	-0.071452	-0.061265	0.068929
H	-0.156944	-0.176348	-0.995135
S	2.372096	-0.068620	-0.372717
H	2.585724	-1.315446	-2.744385

TS: CH_3S^- + C_3H_7F
E = −1806.34
H = −1719.52
G = −1748.67
N_{imag} = −386.468

H	3.561595	-1.554725	-1.826230
C	-0.059942	1.337196	0.700923
C	-0.162512	-1.232459	1.032883
F	-2.025507	-0.026175	-0.312915
H	-1.032182	1.515046	1.172014
H	-1.151096	-1.219129	1.504715
H	0.090516	2.093705	-0.072234
H	-0.068094	-2.173175	0.484938
H	0.724690	1.455689	1.456890
H	0.610542	-1.205169	1.806950
C	-0.057630	-0.046762	0.112291
H	-0.031509	-0.179581	-0.951917
S	2.450881	-0.093468	-0.239184
C	2.600474	-1.564851	-1.296330
H	2.547345	-2.501628	-0.723997
H	1.809678	-1.593912	-2.059490

TS: CH_3OS^- + C_3H_7F
E = −1936.07
H = −1846.39
G = −1875.47
N_{imag} = −376.584

O	3.245763	-0.111779	-0.678938
C	0.133811	1.121637	0.965915
C	-0.014985	-1.286679	0.031809
F	-2.174376	0.172969	0.192276
H	-0.592456	0.939466	1.765649
H	-0.733232	-1.609060	0.793736
H	0.052416	2.167136	0.661633
H	-0.218381	-1.840775	-0.886816
H	1.143331	0.955143	1.356019
H	0.994650	-1.536088	0.375862
C	-0.175242	0.192471	-0.175673
H	-0.581808	0.572160	-1.095712
S	1.913625	0.602902	-1.510849
H	3.067132	-2.017902	-1.533414
C	3.806243	-1.223696	-1.348114
H	4.247814	-0.938525	-2.318077
H	4.602702	-1.616503	-0.697090

TS: CH_3HNS^- + C_3H_7F
E = −1936.07
H = −1846.39
G = −1875.47
N_{imag} = −376.584

N	2.969059	-0.117060	-1.450414
C	-0.057675	1.047432	0.058717
C	-0.003805	-1.000123	1.660064
F	-1.863285	-0.853831	-0.126517
H	-1.118746	1.313144	0.118255
H	-0.985278	-0.763814	2.084121
H	0.316098	1.338905	-0.925986
H	0.107510	-2.086751	1.650818
H	0.497575	1.609994	0.816962
H	0.767895	-0.577074	2.316188
C	0.072920	-0.435113	0.269779
H	0.193829	-1.085287	-0.575412
S	2.606983	-0.522048	0.213044
H	3.888274	-0.775786	-3.216553
H	3.519544	0.735951	-1.449507
C	3.684013	-1.145208	-2.198735
H	4.642429	-1.463190	-1.743669
H	3.051478	-2.035040	-2.279440

TS: CH_3SS^- + C_3H_7F
E = −1901.57
H = −1812.56
G = −1844.79
N_{imag} = −361.298

S	2.821320	-1.889812	-1.407554
C	0.026176	1.305416	0.648831
C	-0.186556	-1.262744	0.977727
F	-2.100620	0.101142	-0.275177
H	-0.895598	1.502992	1.205205
H	-1.226060	-1.314958	1.317891
H	0.116254	2.061928	-0.133848
H	0.052605	-2.192791	0.456644
H	0.876249	1.410746	1.335694
H	0.473883	-1.175969	1.847295
C	-0.055795	-0.076724	0.066299
H	-0.138534	-0.210344	-0.995069
S	2.405687	-0.208749	-0.335420
H	2.948269	-2.272386	-3.761690
C	2.698534	-1.389165	-3.159746
H	1.685010	-1.059941	-3.405397
H	3.407229	-0.584808	-3.381670

Table S23. Cartesian coordinates (Å), energies (in kcal mol^{-1}), and number of imaginary vibrational frequencies (N_{imag}) of the S_N2 reaction between Nu:$^-$ + C_3H_7Cl, computed at ZORA-OLYP/QZ4P.

C_3H_7Cl
E = −1239.37
H = −1177.25
G = −1198.91
***N*imag** = 0

H	0.188284	-0.239278	-0.830252
C	-0.375318	1.620787	0.101508
C	0.026130	-0.606759	1.287416
Cl	-2.103490	-0.440098	-0.430154
H	-1.018009	1.990382	0.905519
H	-0.600788	-0.324093	2.137810
H	-0.694590	2.081832	-0.835985
H	-0.015043	-1.692430	1.173338
H	0.651843	1.943761	0.308500
H	1.061001	-0.326372	1.516759
C	-0.418138	0.100955	0.014209

TS: HO^- + C_3H_7Cl
E = −1465.71
H = −1396.44
G = −1422.38
***N*imag** = −333.185

H	-2.747770	0.605362	-1.336874
O	-2.589147	-0.099373	-0.694068
C	-0.441210	-1.295578	0.651145
C	-0.435863	1.286420	0.660528
Cl	2.000641	0.004550	-0.354683
H	0.169023	-1.315578	1.560073
H	0.218873	1.322560	1.538218
H	-0.168190	-2.151476	0.030273
H	-0.211134	2.150032	0.029415
H	-1.499189	-1.384782	0.917800
H	-1.481481	1.347484	0.977787
C	-0.255512	-0.003742	-0.090882
H	-0.382648	-0.010991	-1.157078

TS: HOO^- + C_3H_7Cl
E = −1566.89
H = −1494.39
G = −1523.23
N_{imag} = −246.740

C	0.369213	-0.161291	1.606908
C	-0.161192	1.623717	-0.189433
H	1.454802	-0.126103	1.467698
H	0.091606	-1.172099	1.913484
H	0.085731	0.529975	2.407730
H	0.879602	1.790769	-0.499588
H	-0.417391	2.346472	0.592385
H	-0.811484	1.801010	-1.048850
O	1.720702	-0.204338	-1.012197
O	2.373417	-1.500213	-0.854713
H	1.976117	-1.997066	-1.583567
C	-0.290121	0.218191	0.312457
H	-0.472436	-0.556561	-0.410824
Cl	-2.481537	0.182848	0.936764

TS: H_2NO^- + C_3H_7Cl
E = −1701.24
H = −1621.02
G = −1650.12
N_{imag} = −235.047

Cl	-2.636235	0.012361	-0.092962
C	-0.347979	0.072900	-0.129114
N	2.180610	-0.277391	-2.247023
H	2.551962	0.427600	-2.901967
H	2.958337	-0.945567	-2.132419
H	-0.350578	0.162579	-1.200614
O	1.936325	0.339460	-1.022662
C	-0.027331	-1.265124	0.471737
H	-0.296450	-2.079981	-0.204090
H	1.051768	-1.312839	0.660019
H	-0.561881	-1.411111	1.416865
C	-0.083877	1.302433	0.682999
H	-0.479206	2.195619	0.193918
H	-0.534087	1.224952	1.678659
H	1.002481	1.424898	0.790539

TS: HSO^- + C_3H_7Cl
E = −1551.43
H = −1481.23
G = −1510.48
***N*$_{imag}$** = −239.265

C	0.552263	0.029549	-0.103688
H	0.648494	-0.010783	-1.172556
O	-1.603171	0.259283	-1.014105
H	-3.216896	0.749976	0.621704
S	-2.841273	-0.286375	-0.214015
Cl	3.084327	-0.156504	-0.299933
C	0.505184	1.350787	0.590791
H	0.681950	2.176409	-0.100624
H	-0.480320	1.488263	1.055660
H	1.258588	1.395612	1.383576
C	0.293654	-1.210689	0.660999
H	0.541012	-2.103784	0.084618
H	0.859067	-1.227407	1.598019
H	-0.782546	-1.251139	0.921510

TS: CH_3O^- + C_3H_7Cl
E = −1827.58
H = −1740.79
G = −1769.61
***N*$_{imag}$** = −311.369

O	2.113168	0.434273	-0.185828
C	-0.361820	1.631488	0.097067
C	-0.044744	-0.583512	1.390945
Cl	-2.404795	-0.459790	-0.386084
H	-1.132785	1.944163	0.808364
H	-0.865018	-0.347330	2.076227
H	-0.627848	2.011418	-0.891379
H	-0.019927	-1.665766	1.248584
H	0.599888	2.067908	0.388845
H	0.901642	-0.265594	1.841624
C	-0.215085	0.140146	0.086222
H	0.064492	-0.346618	-0.829178
C	2.819278	-0.651225	-0.593147
H	2.889634	-1.486794	0.160048
H	2.431222	-1.143569	-1.527620
H	3.886243	-0.406638	-0.835767

TS: CH_3OO^- + C_3H_7Cl
E = −1930.05
H = −1839.72
G = −1870.93
N_{imag} = −264.499

C	0.413731	-0.117662	1.649327
C	-0.223529	1.617701	-0.162435
H	1.493370	-0.034406	1.484455
H	0.188492	-1.136456	1.971238
H	0.121435	0.568047	2.451193
H	0.797482	1.806078	-0.523088
H	-0.459153	2.349527	0.616889
H	-0.916529	1.762622	-0.993615
O	1.621942	-0.205319	-0.998126
O	2.237527	-1.500267	-0.903282
H	2.362492	-3.224570	-1.942496
C	-0.295652	0.222796	0.372602
H	-0.573899	-0.569717	-0.296612
Cl	-2.506893	0.206373	1.148570
C	1.865932	-2.244785	-2.034465
H	0.774376	-2.397536	-2.092719
H	2.194275	-1.756451	-2.969546

TS: CH_3HNO^- + C_3H_7Cl
E = −2067.87
H = −1970.22
G = −1999.57
N_{imag} = −249.695

Cl	-2.629962	-0.035277	-0.241625
C	-0.299791	-0.103928	-0.123986
N	2.272066	0.034952	-2.124400
H	2.965253	0.793139	-2.083004
H	3.171407	-0.649047	-3.894633
H	-0.276225	-0.246762	-1.188715
O	1.998469	-0.373043	-0.837257
C	-0.119185	-1.289257	0.770672
H	-0.471420	-2.206568	0.293987
H	0.950881	-1.407015	0.988854
H	-0.660171	-1.159347	1.714116
C	-0.031204	1.264124	0.428658
H	-0.230933	2.040289	-0.313439
H	-0.652629	1.461635	1.308662
H	1.024729	1.325602	0.718852
C	2.861645	-1.045800	-2.914859
H	3.735244	-1.524424	-2.424490
H	2.107230	-1.824347	-3.075902

TS: CH_3SO^- + C_3H_7Cl
E = −1920.80
H = −1831.63
G = −1863.70
N_{imag} = −255.301

C	0.606591	0.027656	-0.103186
H	0.778484	-0.041031	-1.161402
O	-1.541445	0.174510	-0.957100
H	-1.069126	-2.080340	-2.888802
S	-1.800254	0.230724	-2.512669
Cl	3.084945	-0.195660	-0.046844
C	0.504774	1.367888	0.549096
H	0.824755	2.166407	-0.122887
H	-0.540517	1.550357	0.830670
H	1.119800	1.409488	1.453772
C	0.284850	-1.197799	0.688130
H	0.518586	-2.108145	0.132719
H	0.844543	-1.214598	1.628959
H	-0.788068	-1.199930	0.922077
C	-1.990424	-1.505852	-3.044137
H	-2.814652	-1.993108	-2.506569
H	-2.221588	-1.484998	-4.117046

TS: H_2N^- + C_3H_7Cl
E = −1569.42
H = −1493.25
G = −1519.86
N_{imag} = −294.470

H	-2.839374	-0.833483	-1.104399
N	-2.689170	-0.028253	-0.478937
C	-0.210999	-1.298242	0.780947
C	-0.215085	1.276591	0.751935
Cl	2.126139	-0.020793	-0.444406
H	0.491762	-1.328346	1.621050
H	0.487302	1.327672	1.591342
H	-0.027092	-2.171558	0.149428
H	-0.033526	2.136023	0.100989
H	-1.234312	-1.354441	1.166635
H	-1.238708	1.338393	1.135934
C	-0.056062	-0.019164	0.003854
H	-0.330304	-0.031322	-1.036317
H	-2.840859	0.761680	-1.123263

TS: $HOHN^-$ + C_3H_7Cl

E = −1688.74

H = −1608.94

G = −1638.59

N_{imag} = −164.397

O	-3.443064	-0.873331	-1.577361
N	-2.742786	-0.012529	-0.572433
C	-0.127672	-1.323303	0.792278
C	-0.262561	1.236389	0.712761
Cl	2.195065	0.040450	-0.344275
H	0.505314	-1.316876	1.686884
H	0.335747	1.334903	1.625772
H	0.135152	-2.200469	0.194710
H	-0.057354	2.101258	0.075941
H	-1.175503	-1.413792	1.102391
H	-1.326685	1.254301	0.992593
C	0.043710	-0.051869	0.004252
H	-0.244228	-0.099372	-1.035543
H	-2.738829	0.864050	-1.114523
H	-3.659980	-1.628017	-1.022334

TS: H_2NHN^- + C_3H_7Cl

E = −1813.48

H = −1725.88

G = −1755.45

N_{imag} = −200.393

N	-3.380007	-0.967811	-1.428608
N	-2.858142	-0.056515	-0.406980
C	-0.163788	-1.312268	0.738317
C	-0.289525	1.249024	0.651985
Cl	2.062486	0.031356	-0.645735
H	0.591106	-1.309013	1.532859
H	0.422680	1.348823	1.479067
H	0.000130	-2.196494	0.116719
H	-0.158679	2.105697	-0.014630
H	-1.157544	-1.384768	1.196150
H	-1.308071	1.278499	1.065386
C	-0.089820	-0.048790	-0.074218
H	-0.440119	-0.101563	-1.092287
H	-2.771539	0.820217	-0.937785
H	-4.386181	-0.795246	-1.570570
H	-3.368427	-1.884589	-0.981495

TS: $HSHN^-$ + C_3H_7Cl
E = −1653.56
H = −1576.09
G = −1606.18
N_{imag} = −322.131

S	-3.074481	-1.314950	-1.607991
N	-2.455061	-0.067714	-0.641433
C	-0.126676	-1.339786	0.736683
C	-0.278888	1.245661	0.739894
Cl	2.305662	0.113328	-0.224679
H	0.489890	-1.313397	1.640627
H	0.365383	1.308316	1.622382
H	0.209956	-2.171150	0.115402
H	-0.074501	2.114021	0.109293
H	-1.167149	-1.523877	1.026218
H	-1.325052	1.284455	1.066697
C	-0.038924	-0.037201	0.000821
H	-0.070044	-0.041114	-1.072287
H	-2.552560	0.775318	-1.218196
H	-4.186731	-1.718655	-0.912616

TS: CH_3HN^- + C_3H_7Cl
E = −1929.97
H = −1835.98
G = −1865.17
N_{imag} = −251.891

H	-1.432162	-1.697762	-2.622883
N	-2.309541	-0.812691	-0.827871
C	0.198023	-1.039911	0.982522
C	-0.806690	1.315617	0.741608
Cl	2.044212	1.063971	0.101226
H	0.683104	-0.787343	1.931932
H	-0.375671	1.636387	1.696449
H	0.820798	-1.774924	0.466712
H	-0.839293	2.181610	0.075194
H	-0.778541	-1.490726	1.189450
H	-1.831452	0.966039	0.915588
C	-0.004381	0.193314	0.144941
H	-0.037242	0.070640	-0.923630
H	-2.567352	0.026470	-1.360565
C	-2.165423	-1.874346	-1.792305
H	-1.819395	-2.794239	-1.287992
H	-3.106647	-2.175971	-2.319545

TS: CH_3OHN^- + C_3H_7Cl
E = −2050.52
H = −1952.82
G = −1984.47
N_{imag} = −198.673

O	-3.458271	-0.502300	-1.746993
N	-2.820876	0.054003	-0.545900
C	-0.331139	-1.309698	0.913175
C	-0.274218	1.238743	0.630626
Cl	2.105428	-0.215248	-0.373170
H	0.355549	-1.279180	1.766965
H	0.408035	1.363647	1.479082
H	-0.168009	-2.248084	0.376651
H	-0.066521	2.031749	-0.092348
H	-1.360455	-1.301715	1.290787
H	-1.301030	1.367811	1.005554
C	-0.118467	-0.121536	0.019193
H	-0.363858	-0.230554	-1.023814
H	-2.697263	1.015834	-0.891468
H	-3.128848	-2.473608	-1.157448
C	-3.938600	-1.776628	-1.439964
H	-4.665570	-1.752200	-0.604965
H	-4.434524	-2.161077	-2.342547

TS: CH_3HNHN^- + C_3H_7Cl
E = −2179.60
H = −2073.77
G = −2105.31
N_{imag} = −228.034

N	-3.385944	-1.030537	-1.134409
N	-2.854437	-0.078954	-0.201092
C	-0.154751	-1.295384	0.785014
C	-0.260299	1.267090	0.609465
Cl	1.993726	-0.026096	-0.825752
H	0.691359	-1.279081	1.480929
H	0.536876	1.385090	1.351932
H	-0.079800	-2.202953	0.180898
H	-0.183752	2.091910	-0.103660
H	-1.083948	-1.336233	1.366326
H	-1.226594	1.339477	1.128804
C	-0.160451	-0.063317	-0.074168
H	-0.535737	-0.150634	-1.078980
H	-2.804816	0.781471	-0.763244
H	-5.445628	-0.591616	-0.638193
H	-3.370325	-1.915013	-0.632330
C	-4.783887	-0.787518	-1.509204

H	-4.838871	0.089962	-2.165175
H	-5.170619	-1.652475	-2.072448

TS: CH_3SHN^- + C_3H_7Cl
E = −2023.56
H = −1927.31
G = −1959.80
***N*$_{imag}$** = −296.966

S	-2.838460	-1.722037	-1.593215
N	-2.195510	-0.206020	-1.334208
C	0.055388	-1.425301	0.356702
C	-0.668437	0.985195	0.946339
Cl	2.274162	0.657838	0.340022
H	0.385009	-1.552375	1.392909
H	-0.178602	0.940965	1.924458
H	0.749767	-1.958995	-0.295582
H	-0.601014	2.010184	0.575838
H	-0.935246	-1.896914	0.246481
H	-1.727176	0.734512	1.069032
C	-0.030191	0.016345	-0.003206
H	0.149285	0.311717	-1.019720
H	-2.010081	0.184775	-2.262723
H	-4.449409	-1.298620	0.190542
C	-4.511857	-1.636946	-0.849148
H	-5.163015	-0.954317	-1.410182
H	-4.933880	-2.650522	-0.873284

TS: HS^- + C_3H_7Cl
E = −1417.32
H = −1349.79
G = −1377.10
***N*$_{imag}$** = −307.247

H	2.639326	-1.172116	-1.025945
C	-0.084230	1.328667	0.764805
C	-0.099522	-1.255433	0.892835
Cl	-2.457171	-0.030520	-0.560685
H	-0.978157	1.436586	1.387434
H	-0.952832	-1.256109	1.579419
H	-0.059477	2.152241	0.049114
H	-0.164771	-2.145939	0.264613
H	0.793593	1.404560	1.415189
H	0.820990	-1.311333	1.482089
C	-0.103904	-0.001158	0.067149
H	0.032101	-0.049607	-0.995337
S	2.609316	0.058659	-0.478689

TS: HOS^- + C_3H_7Cl

E = −1551.07

H = −1478.91

G = −1509.39

N_{imag} = −280.633

O	3.139128	-1.460762	-0.927776
C	-0.110822	1.335136	0.777998
C	-0.117872	-1.254478	0.857867
Cl	-2.492212	0.008392	-0.503011
H	-0.933760	1.413494	1.496474
H	-0.932521	-1.276716	1.589671
H	-0.187651	2.171242	0.079852
H	-0.212893	-2.130848	0.214085
H	0.829957	1.428035	1.334558
H	0.832622	-1.323859	1.397157
C	-0.170264	0.017107	0.061316
H	-0.008124	-0.012039	-0.999272
S	2.610411	0.141181	-0.492537
H	3.069995	-1.466755	-1.888258

TS: H_2NS^- + C_3H_7Cl

E = −1671.79

H = −1591.90

G = −1622.27

N_{imag} = −268.599

N	3.262889	-1.236238	-1.314575
C	-0.126169	1.346969	0.811347
C	-0.164360	-1.237537	0.717052
Cl	-2.485827	0.127782	-0.595334
H	-0.944759	1.382874	1.538646
H	-0.991788	-1.307444	1.431611
H	-0.195831	2.230470	0.173591
H	-0.254037	-2.071050	0.016745
H	0.818607	1.393737	1.367258
H	0.775856	-1.342874	1.269997
C	-0.201449	0.084139	0.003197
H	0.015191	0.125121	-1.047788
S	2.647152	0.215216	-0.512994
H	2.827836	-1.306852	-2.232455
H	2.949648	-2.053474	-0.792551

TS: HSS^- + C_3H_7Cl
E = −1506.39
H = −1436.53
G = −1467.63
$\boldsymbol{N_{imag}}$ = −278.832

S	3.103474	-1.776986	-1.387689
C	-0.072681	1.321279	0.751548
C	-0.191127	-1.260491	0.922811
Cl	-2.604464	0.085762	-0.390616
H	-0.864407	1.449062	1.496929
H	-1.049844	-1.262701	1.602183
H	-0.148626	2.136020	0.029400
H	-0.241291	-2.157922	0.304360
H	0.889462	1.404544	1.273575
H	0.721764	-1.306278	1.526574
C	-0.200630	-0.016254	0.085339
H	-0.146975	-0.087373	-0.984043
S	2.488005	-0.001982	-0.562478
H	2.909192	-1.560376	-2.714576

TS: CH_3S^- + C_3H_7Cl
E = −1779.02
H = −1692.41
G = −1723.04
$\boldsymbol{N_{imag}}$ = −296.891

H	3.816150	-1.709728	-1.642189
C	-0.123478	1.351015	0.763644
C	-0.194678	-1.223613	1.003585
Cl	-2.542559	-0.003078	-0.424264
H	-0.963839	1.498517	1.449925
H	-1.012525	-1.175466	1.730388
H	-0.145239	2.144373	0.014490
H	-0.313330	-2.138668	0.420175
H	0.804214	1.441918	1.339581
H	0.752135	-1.276334	1.551177
C	-0.211033	-0.005281	0.124714
H	-0.070730	-0.101363	-0.934522
S	2.532758	-0.006407	-0.478455
C	2.776081	-1.610225	-1.303589
H	2.567992	-2.458089	-0.635558
H	2.135331	-1.716968	-2.189795

TS: CH_3OS^- + C_3H_7Cl
E = −1909.95
H = −1820.45
G = −1850.78
$\boldsymbol{N}_{\mathbf{imag}}$ = −278.596

O	3.447285	-0.609682	-0.489739
C	-0.186102	1.248178	0.985910
C	0.203463	-1.226913	0.328994
Cl	-2.490712	-0.072931	-0.517299
H	-0.985604	1.002735	1.693077
H	-0.452078	-1.533201	1.151024
H	-0.406992	2.224774	0.549498
H	0.047697	-1.912900	-0.505965
H	0.755202	1.321925	1.540735
H	1.242256	-1.325949	0.667061
C	-0.088992	0.185357	-0.068990
H	-0.069799	0.457489	-1.107104
S	2.572359	0.843172	-0.774281
H	2.799240	-1.661513	-2.181071
C	3.719959	-1.356047	-1.660625
H	4.340737	-0.786490	-2.371008
H	4.269090	-2.255126	-1.342001

TS: CH_3HNS^- + C_3H_7Cl
E = −2033.54
H = −1936.23
G = −1967.06
$\boldsymbol{N}_{\mathbf{imag}}$ = −277.826

N	2.899654	-0.668968	-1.933049
C	-0.283009	1.316732	0.784381
C	0.142284	-1.233473	0.859995
Cl	-2.555517	-0.391296	-0.221550
H	-1.004482	1.262937	1.606709
H	-0.620202	-1.422328	1.623646
H	-0.591362	2.115853	0.108032
H	0.205349	-2.111917	0.214819
H	0.691072	1.583399	1.215143
H	1.106354	-1.102557	1.362665
C	-0.204664	-0.001942	0.073577
H	-0.162904	-0.023675	-0.998801
S	2.421056	0.589196	-0.816875
H	4.294455	-2.185004	-2.326703
H	3.078201	-0.236085	-2.833719
C	4.064451	-1.451972	-1.537087
H	4.974718	-0.852146	-1.340524
H	3.833364	-2.001655	-0.618944

TS: CH_3SS^- + C_3H_7Cl
E = −1875.19
H = −1786.94
G = −1818.78
N_{imag} = −273.740

S	3.087039	-1.752189	-1.392882
C	-0.049077	1.339497	0.836841
C	-0.231883	-1.243571	0.875667
Cl	-2.645197	0.226602	-0.332414
H	-0.828166	1.449523	1.598427
H	-1.072687	-1.254159	1.577234
H	-0.113026	2.191970	0.158216
H	-0.328191	-2.105085	0.213204
H	0.921490	1.371537	1.348832
H	0.693625	-1.352223	1.451981
C	-0.220856	0.042330	0.106181
H	-0.193510	0.027292	-0.966207
S	2.473383	0.014684	-0.595557
H	3.228012	-2.503047	-3.656236
C	2.879768	-1.569067	-3.196823
H	1.828884	-1.407914	-3.455400
H	3.478445	-0.732349	-3.571049

Table S24. Cartesian coordinates (Å), energies (in kcal mol^{-1}), and number of imaginary vibrational frequencies (N_{imag}) of the S_N2 reaction between Nu:$^-$ + C_2H_5Cl, computed at COSMO(DCM)-ZORA-OLYP/QZ4P.

C_2H_5Cl
E = -871.67
H = -827.74
G = -847.39
***N*imag** = 0

Cl	-0.094494	-0.040131	0.000016
C	1.722478	0.040520	-0.000004
C	2.233663	1.466686	0.000001
H	3.329875	1.442307	0.000008
H	1.906384	2.012156	0.888965
H	1.906403	2.012150	-0.888973
H	2.034368	-0.509477	0.888817
H	2.034373	-0.509472	-0.888830

H^+
E = -182.18
H = -183.07
G = -175.32
***N*imag** = 0

H	0.000000	0.000000	0.000000

HO^-
E = -299.15
H = -291.67
G = -303.94
***N*imag** = 0

H	0.000000	0.000000	-5.540783
O	0.000000	0.000000	-4.577348

HOO^-
E = -393.50
H = -382.87
G = -398.95
***N*imag** = 0

H	-4.844835	-2.399370	0.000000
O	-4.371755	-1.557792	0.000000
O	-2.946792	-2.029788	0.000000

H_2NO^-
E = -525.64
H = -506.99
G = -523.27
***N*imag** = 0

N	-2.265322	-1.118600	-0.340247
O	-0.959396	-0.661738	0.095604
H	-2.955229	-0.506047	0.111208
H	-2.434750	-2.014086	0.133434

HSO^-
E = -373.01
H = -364.63
G = -381.54
***N*imag** = 0

H	1.814816	-1.161482	0.000000
O	-0.401648	-2.006277	0.000000
S	1.161635	-2.369799	0.000000

CH_3O^-
E = -651.75
H = -626.90
G = -642.65
***N*imag** = 0

C	-0.351598	-1.835547	0.000003
H	0.404434	-1.007001	0.000001
O	-1.658109	-1.413484	-0.000006
H	-0.066346	-2.464124	0.884053
H	-0.066347	-2.464124	-0.884051

CH_3OO^-
E = -753.94
H = -725.43
G = -743.95
***N*imag** = 0

C	-0.793993	-1.465017	-0.745131
H	-0.026928	-0.671748	-0.744207
O	-1.098842	-1.902039	0.553247
H	-0.403601	-2.339826	-1.285468
H	-1.685654	-1.085373	-1.272864
O	-1.626984	-0.751038	1.314201

CH_3HNO^-
E = -889.73
H = -853.13
G = -871.67
***N*_imag** = 0

N	-2.289468	-1.198458	-0.343845
O	-0.950403	-0.729308	-0.153448
C	-3.245464	-0.163342	0.054434
H	-2.420404	-1.954748	0.334851
H	-4.263127	-0.579438	0.019902
H	-3.056500	0.237335	1.068990
H	-3.191451	0.674470	-0.650808

CH_3SO^-
E = -742.95
H = -715.61
G = -735.18
***N*_imag** = 0

S	1.122802	-2.349746	-0.424748
O	-0.140293	-2.020382	0.502991
C	2.422068	-1.175876	0.067826
H	2.108454	-0.137448	-0.098470
H	2.700263	-1.303502	1.121729
H	3.295331	-1.390302	-0.560506

H_2N^-
E = -394.44
H = -380.32
G = -393.77
***N*_imag** = 0

N	1.385910	1.339133	-0.004490
H	0.751721	1.567254	0.768033
H	0.770807	0.817333	-0.637300

$HOHN^-$
E = -509.39
H = -491.60
G = -508.27
***N*_imag** = 0

N	0.730783	1.652742	-0.046363
H	1.241295	1.263334	0.757991
H	-0.174895	0.020171	-0.721664
O	0.671871	0.420566	-0.946911

H_2NHN^-
E = -634.82
H = -608.78
G = -625.65
***N*_imag** = 0

N	1.328810	1.106946	0.025238
H	0.839993	1.725398	0.678734
H	0.494943	0.889631	-1.832034
N	0.227016	0.666670	-0.872016
H	0.208757	-0.354459	-0.869756

$HSHN^-$
E = -475.12
H = -459.64
G = -476.90
***N*_imag** = 0

N	0.777623	1.753893	0.070920
H	1.385014	1.375492	0.804051
H	-0.374677	-0.298262	-0.742433
S	0.681095	0.525691	-1.089485

CH_3HN^-
E = -748.02
H = -715.90
G = -732.98
***N*_imag** = 0

N	0.790935	1.611221	-0.091319
H	1.336838	1.088034	0.598424
H	-0.404302	-0.149785	-0.618139
C	0.279530	0.630651	-1.035938
H	-0.318842	1.135964	-1.813396
H	1.044469	0.046396	-1.605589

CH_3OHN^-
E = -868.59
H = -833.03
G = -852.22
***N*_imag** = 0

O	1.042631	1.432039	-0.241949
H	1.832531	2.748384	-1.473358
H	0.215264	-0.384397	-0.632877
C	0.281126	0.610604	-1.100123
H	-0.735984	1.009136	-1.250003
H	0.753106	0.501135	-2.092813
N	1.105980	2.833984	-0.744970

CH_3HNHN^-
E = −997.71
H = −953.85
G = −973.15
$\boldsymbol{N}_{\mathbf{imag}}$ = 0

N	1.084885	1.489135	-0.288689
H	0.657127	1.515171	0.635513
H	0.153885	-0.387011	-0.559235
C	0.248780	0.578711	-1.081249
H	-0.765884	0.977275	-1.274331
H	0.720196	0.395369	-2.054317
N	1.053432	2.863977	-0.787762
H	1.963500	2.962293	-1.248614

CH_3SHN^-
E = −844.30
H = −809.85
G = −829.84
$\boldsymbol{N}_{\mathbf{imag}}$ = 0

S	1.200097	1.531410	-0.085889
H	1.989468	3.069805	-1.432833
H	0.088079	-0.528253	-0.657068
C	0.176764	0.442477	-1.161848
H	-0.823992	0.868477	-1.300717
H	0.645711	0.293627	-2.141572
N	1.218526	3.073344	-0.756166

HS^-
E = −245.74
H = −239.90
G = −253.17
$\boldsymbol{N}_{\mathbf{imag}}$ = 0

S	1.340734	0.775860	0.735715
H	1.443543	-0.524870	0.400998

HOS^-
E = −376.37
H = −366.07
G = −382.94
$\boldsymbol{N}_{\mathbf{imag}}$ = 0

H	-4.895751	-2.391936	0.000000
O	-4.398600	-1.566089	0.000000
S	-2.701032	-2.086954	0.000000

H_2NS^-
E = −496.69
H = −478.38
G = −495.54
***N*imag** = 0

N	-2.369243	-1.154744	-0.353217
S	-0.714086	-0.576614	0.117405
H	-3.026916	-0.527209	0.106762
H	-2.504453	-2.041904	0.129049

HSS^-
E = −328.61
H = −320.54
G = −338.43
***N*imag** = 0

H	-5.124222	-2.552839	0.000000
S	-4.422391	-1.396013	0.000000
S	-2.448769	-2.096128	0.000000

CH_3S^-
E = −604.50
H = −579.50
G = −596.23
***N*imag** = 0

C	-0.224778	-1.876517	-0.000007
H	0.467894	-1.027147	-0.000021
S	-1.975115	-1.311101	0.000072
H	-0.002953	-2.484767	0.884339
H	-0.003015	-2.484748	-0.884384

CH_3OS^-
E = −733.61
H = −705.29
G = −724.67
***N*imag** = 0

C	-0.785422	-1.466145	-0.770166
H	-0.013383	-0.681021	-0.786209
O	-1.105216	-1.867571	0.550703
H	-0.400261	-2.355928	-1.285919
H	-1.668466	-1.092711	-1.311837
S	-1.746595	-0.566590	1.539854

CH_3HNS^-
E = -857.46
H = -821.14
G = -840.60
N_{imag} = 0

N	-2.354711	-1.212595	-0.333620
S	-0.634565	-0.715773	-0.177311
C	-3.294364	-0.160692	0.062956
H	-2.469383	-1.978751	0.324706
H	-4.315812	-0.565479	0.030885
H	-3.113104	0.250425	1.071537
H	-3.234877	0.669378	-0.649076

CH_3SS^-
E = -697.17
H = -670.01
G = -690.57
N_{imag} = 0

C	-0.796023	-1.464887	-0.739533
H	-0.036652	-0.677793	-0.711013
S	-1.182814	-2.056920	0.935624
H	-0.405150	-2.326156	-1.293236
H	-1.694034	-1.090372	-1.239872
S	-1.917146	-0.393839	1.942299

HOH
E = -329.48
H = -313.99
G = -327.44
N_{imag} = 0

H	-0.007037	-0.005467	-5.535039
O	-0.005044	-0.003919	-4.570122
H	-0.744990	-0.578761	-4.339614

HOOH
E = -416.62
H = -397.78
G = -414.01
N_{imag} = 0

H	-4.708450	-2.294869	-0.276339
O	-4.301896	-1.599494	0.265528
O	-2.911077	-2.028629	0.271230
H	-2.527204	-1.422511	-0.382766

H_2NOH

E = -557.66

H = -530.34

G = -547.20

N_{imag} = 0

N	-2.230186	-1.123581	-0.350567
O	-0.951077	-0.687896	0.185295
H	-2.897579	-0.474967	0.069323
H	-2.398881	-2.010111	0.126952
H	-0.428345	-0.548824	-0.613993

HSOH

E = -388.29

H = -371.92

G = -389.35

N_{imag} = 0

H	1.577165	-1.277822	-0.399309
O	-0.336519	-2.059867	0.643703
S	1.294056	-2.358891	0.365232
H	-0.826490	-2.499162	-0.065753

CH_3OH

E = -683.52

H = -649.41

G = -666.44

N_{imag} = 0

C	-0.290930	-1.835447	0.007331
H	0.414147	-0.994499	-0.018070
O	-1.652379	-1.389811	-0.056253
H	-0.100579	-2.434954	0.906989
H	-0.120814	-2.461088	-0.872184
H	-1.811987	-0.835787	0.716257

CH_3OOH

E = -777.44

H = -740.77

G = -759.98

N_{imag} = 0

C	-0.771885	-1.457582	-0.791660
H	-0.016063	-0.665520	-0.747952
O	-1.114229	-1.923926	0.511391
H	-0.350777	-2.340944	-1.283444
H	-1.655185	-1.114756	-1.344058
O	-1.632654	-0.791051	1.274399
H	-2.589849	-0.938639	1.198378

CH_3HNOH
E = -921.22
H = -876.04
G = -895.22
N_{imag} = 0

N	-2.295144	-1.307207	-0.203369
O	-0.943924	-0.896995	0.156143
C	-3.192633	-0.175797	0.039081
H	-2.500658	-2.013924	0.502212
H	-4.216039	-0.548884	-0.076692
H	-3.086551	0.261612	1.041976
H	-3.018902	0.599672	-0.711786
H	-0.458097	-1.034151	-0.666867

CH_3SOH
E = -759.88
H = -724.64
G = -744.77
N_{imag} = 0

S	1.137260	-2.300136	-0.730312
O	-0.293727	-1.914727	0.067261
C	2.332543	-1.225656	0.079602
H	2.055501	-0.173472	-0.024205
H	2.471405	-1.490134	1.131148
H	3.271214	-1.401188	-0.458706
H	-0.366907	-2.513061	0.824691

H_2NH
E = -445.99
H = -422.46
G = -436.17
N_{imag} = 0

N	1.369275	1.374702	-0.023717
H	0.783977	1.557116	0.788782
H	0.803372	0.794830	-0.639744
H	2.114094	0.766950	0.310736

HOHNH
E = -555.88
H = -528.65
G = -545.37
N_{imag} = 0

N	0.773895	1.631090	-0.032367
H	1.173936	1.165944	0.784232
H	-0.040812	-0.065913	-0.586851
O	0.528310	0.595321	-1.008890
H	-0.143339	1.968658	0.264250

H_2NHNH

E = -688.13

H = -652.59

G = -669.08

N_{imag} = 0

N	1.222166	1.095316	0.116986
H	0.832606	1.941859	0.522555
H	0.327007	1.067264	-1.718723
N	0.235242	0.597701	-0.817957
H	0.467354	-0.376797	-0.989272
H	2.066539	1.386665	-0.375404

HSHNH

E = -512.61

H = -488.28

G = -505.88

N_{imag} = 0

N	0.823325	1.738555	0.142371
H	1.327938	1.275643	0.891924
H	-0.209222	-0.315936	-0.609011
S	0.539322	0.670431	-1.167364
H	-0.047565	2.105153	0.513739

CH_3HNH

E = -803.82

H = -761.72

G = -778.88

N_{imag} = 0

N	0.827468	1.587369	-0.104396
H	1.344113	1.084473	0.612711
H	-0.406499	-0.136411	-0.593204
C	0.250112	0.618556	-1.051857
H	-0.330777	1.154475	-1.809065
H	1.058260	0.092122	-1.569310
H	0.074502	2.055566	0.393500

CH_3OHNH

E = -915.15

H = -870.09

G = -889.21

N_{imag} = 0

O	0.962066	1.500391	-0.228161
H	1.708106	2.650597	-1.658496
H	0.177642	-0.330742	-0.592980
C	0.290709	0.614998	-1.130409
H	-0.701055	1.001466	-1.403031
H	0.883596	0.449655	-2.040595

N	1.188425	2.798694	-0.788395
H	0.271871	3.150265	-1.080585

CH_3HNHNH

E = −1050.68

H = −997.29

G = −1016.42

***N*imag** = 0

N	1.051670	1.493989	-0.240501
H	0.589602	1.564773	0.660993
H	0.149430	-0.362562	-0.580818
C	0.270404	0.595778	-1.096902
H	-0.730781	0.987878	-1.344726
H	0.810781	0.415152	-2.031850
N	1.148016	2.841585	-0.731323
H	1.851611	2.838314	-1.466131
H	0.273317	3.115367	-1.189220

CH_3SHNH

E = −882.75

H = −839.55

G = −859.81

***N*imag** = 0

S	1.090014	1.574775	-0.029039
H	1.849663	2.953704	-1.689353
H	0.075280	-0.467691	-0.643224
C	0.203985	0.480526	-1.175925
H	-0.780920	0.883279	-1.428038
H	0.786278	0.301972	-2.084153
N	1.297478	3.059359	-0.842558
H	0.407466	3.487082	-1.083521

HSH

E = −252.96

H = −241.31

G = −255.96

***N*imag** = 0

S	1.336296	0.810578	0.833436
H	1.437803	-0.423572	0.308248
H	1.316714	1.387338	-0.381427

HOSH
E = −388.29
H = −371.92
G = −389.35
$\boldsymbol{N}_{\mathbf{imag}}$ = 0

H	-4.844667	-2.395246	0.033269
O	-4.306123	-1.591619	0.055683
S	-2.702241	-2.078637	-0.071501
H	-2.420212	-2.213368	1.245914

H_2NSH
E = −512.51
H = −488.08
G = −505.79
$\boldsymbol{N}_{\mathbf{imag}}$ = 0

N	-2.321368	-1.012442	-0.293967
S	-0.811987	-0.413861	0.305779
H	-3.072339	-0.571924	0.229690
H	-2.367341	-2.010328	-0.108717
H	-0.345211	0.088599	-0.856844

HSSH
E = −335.11
H = −321.18
G = −339.19
$\boldsymbol{N}_{\mathbf{imag}}$ = 0

H	-5.034732	-2.565140	0.033038
S	-4.371680	-1.388402	0.048015
S	-2.440701	-2.104495	-0.068830
H	-2.178011	-2.219179	1.251140

CH_3SH
E = −618.95
H = −587.87
G = −606.00
$\boldsymbol{N}_{\mathbf{imag}}$ = 0

C	-0.254406	-1.900927	-0.037367
H	0.330902	-0.985881	-0.141075
S	-2.037782	-1.535971	0.032474
H	-0.006434	-2.375856	0.913998
H	-0.025478	-2.592036	-0.850148
H	-2.148822	-0.966210	-1.181159

CH_3OSH
E = −745.07
H = −710.71
G = −730.81
$\boldsymbol{N}_{\mathbf{imag}}$ = 0

C	-0.712971	-1.441582	-0.820350
H	0.253460	-0.928794	-0.784182
O	-1.226771	-1.676406	0.508285
H	-0.591845	-2.433420	-1.264227
H	-1.423195	-0.855270	-1.414192
S	-1.368389	-0.315334	1.467040
H	-2.556124	0.150357	1.005800

CH_3HNSH
E = −873.64
H = −831.29
G = −851.26
$\boldsymbol{N}_{\mathbf{imag}}$ = 0

N	-2.316579	-1.211061	-0.261222
S	-0.660561	-0.809292	-0.234023
C	-3.266557	-0.151162	0.091808
H	-2.479532	-2.044007	0.294867
H	-4.280514	-0.550357	-0.017263
H	-3.145290	0.221407	1.119937
H	-3.147310	0.687848	-0.598262
H	-0.458099	-0.439939	1.062835

CH_3SSH
E = −704.68
H = −671.73
G = −692.89
$\boldsymbol{N}_{\mathbf{imag}}$ = 0

C	-0.716445	-1.464041	-0.791465
H	0.231334	-0.927646	-0.721185
S	-1.353264	-1.879375	0.859359
H	-0.552524	-2.428305	-1.283525
H	-1.446952	-0.886216	-1.360705
S	-1.515102	-0.073747	1.824050
H	-2.759613	0.283779	1.432324

TS: HO^- + C_2H_5Cl
E = −1155.27
H = −1103.74
G = −1127.56
N_{imag} = −420.701

C	-1.029182	-5.670635	0.056136
C	-0.862409	-4.193793	-0.131268
O	1.451943	-4.244843	-0.213122
H	-1.527822	-5.899401	1.001172
H	-0.040538	-6.136371	0.069761
H	-1.604283	-6.113991	-0.760414
H	-0.606984	-3.571311	0.708669
H	-0.677952	-3.795176	-1.113828
Cl	-2.964984	-3.484208	-0.135229
H	1.606777	-3.298646	-0.323720

TS: HOO^- + C_2H_5Cl
E = −1253.67
H = −1198.97
G = −1225.43
N_{imag} = −359.129

C	-0.816907	-5.831316	0.260495
C	-0.757868	-4.366794	-0.046861
Cl	1.363056	-3.904006	0.147662
H	-0.461392	-6.044093	1.271843
H	-0.225587	-6.415257	-0.449238
H	-1.856668	-6.170840	0.191621
H	-1.062057	-3.656547	0.704396
H	-0.821815	-4.035304	-1.069983
O	-3.064248	-4.287365	-0.386519
O	-3.510377	-2.939605	-0.760575
H	-3.616536	-2.516673	0.102842

TS: H_2NO^- + C_2H_5Cl
E = −1386.71
H = −1323.98
G = −1350.79
N_{imag} = −335.974

Cl	1.623258	1.711257	0.001429
C	0.011756	0.282530	-0.000465
N	-0.432069	-2.881943	0.000470
H	-0.696313	-3.457877	0.807065
H	-0.693764	-3.460496	-0.805080
H	0.304858	-0.213522	0.910426
O	-1.274726	-1.737629	-0.002697
C	-1.222712	1.129879	0.000157
H	-1.277335	1.764397	0.888756

H	-1.276945	1.766362	-0.887056
H	-2.097819	0.470518	-0.000721
H	0.305258	-0.211576	-0.912287

TS: HSO^- + C_2H_5Cl
E = −1230.54
H = −1178.08
G = −1205.26
N_{imag} = −372.805

C	0.961019	1.652089	-0.042140
H	0.047938	2.258886	-0.025545
H	1.513260	1.902773	-0.950759
H	1.561841	1.930129	0.826874
O	-1.653031	0.603039	0.013730
H	-2.733021	-1.070315	1.263252
C	0.576089	0.211053	-0.009174
H	0.328178	-0.265414	0.924558
Cl	2.626406	-0.810683	-0.018819
H	0.300787	-0.303124	-0.914531
S	-2.602197	-0.687129	-0.045750

TS: CH_3O^- + C_2H_5Cl
E = −1510.65
H = −1442.03
G = −1467.63
N_{imag} = −392.714

C	-1.063504	-5.656346	0.600601
C	-0.700779	-4.469245	-0.236902
O	1.595805	-4.808503	-0.031869
H	-1.634432	-5.363753	1.485321
H	-0.140948	-6.140468	0.933748
H	-1.646518	-6.385512	0.032529
H	-0.380687	-3.558490	0.240934
H	-0.398347	-4.611640	-1.260760
Cl	-2.658393	-3.650554	-0.788245
C	2.265964	-3.813503	-0.711002
H	2.036463	-3.772289	-1.803573
H	3.372505	-3.925332	-0.653314
H	2.059685	-2.783155	-0.331500

TS: CH_3OO^- + C_2H_5Cl
E = −1613.29
H = −1540.66
G = −1569.20
N_{imag} = −351.516

C	-0.982341	-1.685047	-2.132530
C	-1.052311	-0.404593	-1.371261

O	0.451094	-0.976677	0.371096
H	-1.920021	-2.243408	-2.073346
H	-0.185765	-2.303145	-1.700891
H	-0.738833	-1.514654	-3.184561
H	-1.611610	-0.354697	-0.451411
H	-0.284072	0.338309	-1.507931
Cl	-2.571154	0.780046	-2.490802
O	1.414089	-1.994635	0.020390
C	2.580837	-1.369106	-0.478238
H	2.375569	-0.791033	-1.391050
H	3.284483	-2.178566	-0.713455
H	3.031279	-0.701639	0.271979

TS: CH_3HNO^- + C_2H_5Cl

E = −1750.04

H = −1670.02

G = −1697.50

$\boldsymbol{N_{imag}}$ = −334.374

Cl	1.549751	1.894387	0.246155
C	0.012036	0.391798	0.033178
N	-0.318605	-2.797488	0.007398
H	-0.117543	-4.501009	1.216553
H	-0.449132	-3.405543	-0.806193
H	0.224731	-0.112884	0.961372
O	-1.165460	-1.691017	-0.167006
C	-1.253577	1.181277	-0.087095
H	-1.436951	1.789701	0.802625
H	-1.241611	1.836690	-0.962012
H	-2.089665	0.481335	-0.197211
H	0.429057	-0.064812	-0.849562
C	-0.716339	-3.580501	1.180375
H	-0.516442	-3.002270	2.089230
H	-1.788125	-3.852877	1.179197

TS: CH_3SO^- + C_2H_5Cl

E = −1600.51

H = −1529.76

G = −1557.92

$\boldsymbol{N_{imag}}$ = −355.119

C	0.955865	1.658136	-0.000423
H	0.011012	2.214058	0.035531
H	1.510107	1.997713	-0.878632
H	1.525309	1.908806	0.897850
O	-1.637536	0.496067	-0.065838
H	-1.924764	-1.512862	2.155133
C	0.646924	0.201525	-0.070564
H	0.410155	-0.346345	0.825464

Cl	2.744103	-0.725924	-0.137098
H	0.388708	-0.256912	-1.010203
S	-2.562354	-0.803348	-0.095102
C	-2.855972	-1.244786	1.644517
H	-3.347321	-0.425936	2.182006
H	-3.520052	-2.117010	1.634594

TS: H_2N^- + C_2H_5Cl
E = −1254.38
H = −1196.00
G = −1221.00
N_{imag} = −385.485

C	-0.858549	-5.795206	0.134762
C	-0.654310	-4.306900	0.095871
Cl	1.480394	-4.081641	0.268586
H	-0.440191	-6.227620	1.047769
H	-0.392082	-6.283337	-0.725305
H	-1.927793	-6.019261	0.116557
H	-0.904542	-3.724190	0.967646
H	-0.737794	-3.790154	-0.846207
N	-3.178804	-3.672302	-0.193340
H	-3.176936	-4.172983	-1.087040
H	-2.988291	-2.708627	-0.488132

TS: $HOHN^-$ + C_2H_5Cl
E = −1372.39
H = −1310.51
G = −1337.95
N_{imag} = −335.893

C	-0.760365	-5.819037	0.019416
C	-0.831569	-4.334812	0.245748
Cl	1.180239	-3.736510	0.487722
H	-0.277549	-6.324786	0.860101
H	-0.207271	-6.057765	-0.892987
H	-1.771597	-6.225424	-0.077920
H	-1.198658	-3.980904	1.196783
H	-1.053469	-3.698534	-0.597693
N	-3.441312	-4.284747	0.048481
H	-3.345923	-4.392081	-0.971527
O	-3.923198	-2.864924	0.134582
H	-3.113900	-2.342532	0.218264

TS: H_2NHN^- + C_2H_5Cl
E = −1498.61
H = −1428.55
G = −1456.12
N_{imag} = −314.574

C	-0.765672	-5.843235	0.071701
C	-0.620593	-4.349076	0.175250
Cl	1.403916	-3.997780	0.409561
H	-0.406444	-6.342771	0.976083
H	-0.213787	-6.240364	-0.785113
H	-1.822311	-6.095542	-0.056005
H	-0.991743	-3.874030	1.071328
H	-0.788725	-3.773624	-0.724087
N	-3.289059	-3.816492	-0.072696
H	-3.229623	-4.105342	-1.054089
N	-3.442274	-2.356019	-0.178700
H	-3.893558	-2.061084	0.685706
H	-2.515720	-1.914407	-0.113608

TS: $HSHN^{-}$ + C_2H_5Cl

E = −1333.18

H = −1273.49

G = −1301.22

$\boldsymbol{N}_{\mathbf{imag}}$ = −412.053

C	-0.796600	-5.844438	0.077711
C	-0.521852	-4.372123	0.001440
Cl	1.677779	-4.300871	0.020563
H	-0.369913	-6.280226	0.984620
H	-0.388979	-6.371823	-0.788392
H	-1.876476	-6.018113	0.103769
H	-0.624548	-3.761410	0.882630
H	-0.595212	-3.863849	-0.945520
N	-2.904056	-3.711158	0.001584
H	-3.200062	-4.181281	-0.858954
S	-3.057822	-2.050357	-0.346861
H	-4.167866	-1.662787	0.351751

TS: CH_3HN^{-} + C_2H_5Cl

E = −1611.12

H = −1534.72

G = −1562.34

$\boldsymbol{N}_{\mathbf{imag}}$ = −327.992

C	-0.704232	-5.939985	0.321826
C	-0.615825	-4.460412	0.073124
Cl	1.397323	-3.971687	0.434759
H	-0.452447	-6.187723	1.356862
H	-0.034905	-6.496894	-0.340066
H	-1.727211	-6.274861	0.128342
H	-1.057628	-3.795440	0.799510
H	-0.679497	-4.111982	-0.946217
N	-3.214324	-4.137185	-0.497181
H	-3.369874	-4.284863	0.502998

H	-3.228506	-2.491471	-1.803100
C	-3.375394	-2.714324	-0.733536
H	-4.376631	-2.295736	-0.482127
H	-2.652742	-2.055849	-0.195013

TS: CH_3OHN^- + C_2H_5Cl

E = −1731.15
H = −1651.30
G = −1681.78
N_{imag} = −301.131

C	-0.712089	-5.826227	0.041259
C	-0.622874	-4.340925	0.244379
Cl	1.379762	-3.949046	0.723816
H	-0.424598	-6.369958	0.945869
H	-0.071991	-6.158301	-0.780970
H	-1.745113	-6.101224	-0.198963
H	-1.089269	-3.926870	1.126296
H	-0.700476	-3.708151	-0.628486
N	-3.295627	-4.103706	-0.353484
H	-2.967021	-4.134173	-1.326699
O	-3.844040	-2.724215	-0.361021
H	-3.616009	-2.524454	1.700846
H	-5.196462	-3.156931	1.164327
C	-4.380582	-2.457827	0.908171
H	-4.779924	-1.436974	0.883128

TS: CH_3HNHN^- + C_2H_5Cl

E = −1860.41
H = −1772.60
G = −1802.94
N_{imag} = −294.867

C	-0.648631	-5.846191	0.084355
C	-0.645315	-4.344739	0.179061
Cl	1.306793	-3.806547	0.604649
H	-0.349140	-6.306432	1.030587
H	0.025263	-6.200083	-0.701263
H	-1.660200	-6.187902	-0.153030
H	-1.147483	-3.901569	1.026669
H	-0.779012	-3.793072	-0.740972
N	-3.335958	-4.053986	-0.369664
H	-3.133915	-4.193354	-1.365771
N	-3.688300	-2.643838	-0.303261
H	-3.984194	-2.796749	1.822926
H	-2.827301	-2.093178	-0.206625
C	-4.455040	-2.383958	0.910013
H	-4.573519	-1.300810	1.043407
H	-5.451225	-2.835525	0.823953

TS: CH_3SHN^- + C_2H_5Cl

E = −1701.94
H = −1623.38
G = −1653.99
N_{imag} = −387.618

C	-0.822640	-5.883793	0.441002
C	-0.516161	-4.467521	0.054353
Cl	1.674098	-4.433976	0.044319
H	-0.413617	-6.126973	1.425011
H	-0.419447	-6.590666	-0.288789
H	-1.906600	-6.027118	0.478794
H	-0.602928	-3.686042	0.791087
H	-0.610532	-4.161881	-0.974088
N	-2.896206	-3.794265	-0.207007
H	-3.246837	-3.925264	0.746944
H	-4.546400	-1.021933	-2.079641
S	-3.101364	-2.153735	-0.542696
H	-5.417998	-2.381854	-1.313498
H	-4.243981	-2.693717	-2.626160
C	-4.466692	-2.066780	-1.756152

TS: HS^- + C_2H_5Cl

E = −1099.75
H = −1050.18
G = −1075.70
N_{imag} = −405.461

C	-1.119446	-5.744576	0.084496
C	-0.796563	-4.298169	-0.142813
S	1.868882	-4.424711	-0.223453
H	-1.655865	-5.881401	1.026227
H	-0.206661	-6.343475	0.135040
H	-1.728842	-6.141082	-0.730798
H	-0.556463	-3.651521	0.683344
H	-0.624538	-3.922512	-1.136585
Cl	-2.939445	-3.392542	-0.197522
H	2.038585	-3.099692	-0.394800

TS: HOS^- + C_2H_5Cl

E = −1234.30
H = −1180.09
G = −1208.28
N_{imag} = −382.568

C	-0.791832	-5.859100	0.251165
C	-0.796829	-4.385560	-0.032936
Cl	1.370253	-3.855084	0.250616
H	-0.446246	-6.061588	1.267829
H	-0.147847	-6.392348	-0.452365

H	-1.797453	-6.282693	0.158195
H	-1.115219	-3.690382	0.726154
H	-0.801523	-4.030478	-1.050260
S	-3.484368	-4.212413	-0.545677
O	-3.807870	-2.569608	-1.016146
H	-3.986512	-2.101285	-0.191602

TS: H_2NS^- + C_2H_5Cl

E = −1355.09

H = −1292.95

G = −1321.28

$\boldsymbol{N}_{\mathbf{imag}}$ = −381.762

C	-0.771795	-5.873318	0.261768
C	-0.817843	-4.405570	-0.045898
Cl	1.340419	-3.831657	0.084691
H	-0.356780	-6.054623	1.256418
H	-0.168589	-6.411697	-0.473564
H	-1.777432	-6.305195	0.241730
H	-1.114527	-3.704977	0.717269
H	-0.908198	-4.071857	-1.066477
S	-3.556819	-4.215089	-0.407216
N	-3.788953	-2.503470	-0.813169
H	-4.446433	-2.108201	-0.144786
H	-4.257888	-2.455192	-1.714849

TS: HSS^- + C_2H_5Cl

E = −1185.37

H = −1133.44

G = −1162.49

$\boldsymbol{N}_{\mathbf{imag}}$ = −382.717

C	-0.652195	-5.899037	0.191224
C	-0.931881	-4.434858	0.346610
Cl	0.964320	-3.732915	1.423295
H	0.256343	-6.066088	-0.392242
H	-1.472624	-6.400471	-0.333140
H	-0.541947	-6.384433	1.163822
H	-1.549266	-4.077906	1.153876
H	-0.747780	-3.754713	-0.468274
S	-3.358396	-4.411038	-0.840288
S	-4.117596	-2.499202	-0.656549
H	-3.622853	-1.901608	-1.765936

TS: CH_3S^- + C_2H_5Cl
E = −1461.36
H = −1392.50
G = −1421.44
N_{imag} = −396.792

C	-1.277855	-5.856612	0.094827
C	-0.893028	-4.410177	-0.007507
S	1.771990	-4.690845	-0.522084
H	-1.681467	-6.085913	1.083979
H	-0.407142	-6.498045	-0.064714
H	-2.024289	-6.117992	-0.659124
H	-0.442749	-3.907371	0.831638
H	-0.796240	-3.940953	-0.972030
Cl	-2.882807	-3.389304	0.363809
C	2.213701	-2.922015	-0.653190
H	1.991089	-2.385660	0.275873
H	1.667570	-2.433743	-1.467872
H	3.284594	-2.811214	-0.854774

TS: CH_3OS^- + C_2H_5Cl
E = −1591.16
H = −1518.95
G = −1549.79
N_{imag} = −381.839

C	-0.759814	-5.911888	0.220813
C	-0.730317	-4.430358	-0.010965
Cl	1.463906	-3.963287	0.266137
H	-0.403246	-6.159098	1.223726
H	-0.142666	-6.436609	-0.512490
H	-1.777807	-6.306909	0.131690
H	-1.017334	-3.754784	0.777451
H	-0.731893	-4.037763	-1.014278
S	-3.419559	-4.213314	-0.509237
O	-3.793740	-2.586670	-0.932878
C	-4.175029	-1.763964	0.167496
H	-3.370861	-1.683148	0.911523
H	-5.078198	-2.146188	0.663591
H	-4.383701	-0.770144	-0.246425

TS: CH_3HNS^- + C_2H_5Cl
E = −1715.66
H = −1635.53
G = −1666.33
N_{imag} = −380.473

C	-0.768354	-5.868022	0.256398
C	-0.842166	-4.402215	-0.052094
Cl	1.295878	-3.763614	0.178940

H	-0.396854	-6.039325	1.269915
H	-0.115331	-6.387583	-0.449312
H	-1.759242	-6.328656	0.188322
H	-1.191999	-3.711435	0.697298
H	-0.894325	-4.068904	-1.075467
S	-3.571838	-4.282704	-0.535973
N	-3.874467	-2.575113	-0.822361
C	-4.721310	-1.921476	0.178851
H	-4.326242	-2.498671	-1.728873
H	-4.204539	-1.914745	1.143655
H	-5.702287	-2.404588	0.324453
H	-4.889747	-0.880230	-0.126246

TS: CH_3SS^- + C_2H_5Cl

E = −1554.20

H = −1483.82

G = −1513.68

$\boldsymbol{N}_{\mathbf{imag}}$ = −375.992

C	-0.726162	-5.943228	0.273666
C	-0.767349	-4.471728	-0.004047
Cl	1.438712	-3.902929	0.232195
H	-0.336638	-6.144021	1.274678
H	-0.105739	-6.465674	-0.458462
H	-1.729937	-6.378711	0.221749
H	-1.056667	-3.780640	0.769950
H	-0.781977	-4.106105	-1.017214
S	-3.445326	-4.291494	-0.479945
S	-3.909035	-2.378891	-1.052312
C	-4.283796	-1.479632	0.485724
H	-3.413220	-1.453602	1.145855
H	-5.131893	-1.933879	1.004786
H	-4.543837	-0.457847	0.188499

Table S25. Cartesian coordinates (Å), energies (in kcal mol^{-1}), and number of imaginary vibrational frequencies (N_{imag}) of the S_N2 reaction between Nu:$^-$ + C_2H_5Cl, computed at COSMO(Water)-ZORA-OLYP/QZ4P.

C_2H_5Cl
E = -872.02
H = -828.12
G = -847.78
N_{imag} = 0

Cl	-0.095544	-0.040821	0.000017
C	1.724132	0.040790	-0.000001
C	2.233323	1.467039	0.000001
H	3.329456	1.441565	0.000017
H	1.906684	2.012258	0.889390
H	1.906714	2.012243	-0.889408
H	2.034151	-0.509175	0.889173
H	2.034133	-0.509159	-0.889190

H^+
E = -169.91
H = -170.80
G = -163.05
N_{imag} = 0

H	0.000000	0.000000	0.000000

HO^-
E = -309.08
H = -301.60
G = -313.87
N_{imag} = 0

H	0.000000	0.000000	-5.540702
O	0.000000	0.000000	-4.577429

HOO^-
E = -403.09
H = -392.44
G = -408.52
N_{imag} = 0

H	-4.847599	-2.399105	0.000000
O	-4.369812	-1.559492	0.000000
O	-2.945971	-2.028353	0.000000

H_2NO^-
E = -535.58
H = -516.82
G = -533.11
N

N	-2.266962	-1.119150	-0.339998
O	-0.958059	-0.661287	0.095912
H	-2.955053	-0.506109	0.110928
H	-2.434624	-2.013926	0.133157

HSO^-
E = -380.78
H = -372.38
G = -389.30
N_{imag} = 0

H	1.812133	-1.162313	0.000000
O	-0.402491	-2.004330	0.000000
S	1.165161	-2.370914	0.000000

CH_3O^-
E = -660.78
H = -635.75
G = -651.51
N_{imag} = 0

C	-0.346104	-1.837341	-0.000001
H	0.403104	-1.007259	0.000002
O	-1.660273	-1.412761	-0.000003
H	-0.067351	-2.463457	0.883478
H	-0.067342	-2.463462	-0.883477

CH_3OO^-
E = -763.35
H = -734.78
G = -753.30
N_{imag} = 0

C	-0.794525	-1.461335	-0.746099
H	-0.025556	-0.671673	-0.746106
O	-1.101425	-1.895257	0.557080
H	-0.405735	-2.339735	-1.279397
H	-1.685589	-1.085396	-1.274826
O	-1.630872	-0.744952	1.320272

CH_3HNO^-
E = -899.44
H = -862.73
G = -881.28
N N_{imag} = 0

N	-2.291472	-1.199450	-0.343460
O	-0.946162	-0.731053	-0.155540
C	-3.245795	-0.161876	0.054407
H	-2.419354	-1.953327	0.337017
H	-4.262012	-0.578584	0.018869
H	-3.059130	0.234939	1.069392
H	-3.192891	0.675863	-0.650609

CH_3SO^-
E = -750.97
H = -723.60
G = -743.17
N_{imag} = 0

S	1.126402	-2.349482	-0.425292
O	-0.142670	-2.019546	0.503870
C	2.421935	-1.175378	0.068580
H	2.107952	-0.137988	-0.099305
H	2.700886	-1.304654	1.121408
H	3.294122	-1.390208	-0.560438

H_2N^-
E = -403.69
H = -389.54
G = -402.99
N_{imag} = 0

N	1.385296	1.338981	-0.004436
H	0.752024	1.567270	0.767897
H	0.771120	0.817469	-0.637219

$HOHN^-$
E = -518.47
H = -500.64
G = -517.29
N_{imag} = 0

N	0.737380	1.654017	-0.049596
H	1.235523	1.260761	0.759625
H	-0.172199	0.015758	-0.717105
O	0.668351	0.426278	-0.949871

H_2NHN^-
E = -644.01
H = -617.91
G = -634.80
N$_{imag}$ = 0

N	1.328280	1.107679	0.026032
H	0.840721	1.725943	0.679727
H	0.494232	0.889178	-1.832603
N	0.228156	0.666008	-0.872586
H	0.208131	-0.354625	-0.870405

$HSHN^-$
E = -482.70
H = -467.17
G = -484.44
N_{imag} = 0

N	0.777093	1.753151	0.071404
H	1.384012	1.375036	0.804480
H	-0.372466	-0.295519	-0.740473
S	0.680416	0.524146	-1.092359

CH_3HN^-
E = -756.88
H = -724.62
G = -741.70
N_{imag} = 0

N	0.794409	1.611814	-0.091736
H	1.333371	1.085727	0.600571
H	-0.401155	-0.147215	-0.615280
C	0.278598	0.629239	-1.039162
H	-0.320892	1.134552	-1.814561
H	1.044298	0.048363	-1.605790

CH_3OHN^-
E = -877.35
H = -841.69
G = -860.83
N_{imag} = 0

O	1.040086	1.432876	-0.236635
H	1.821403	2.742718	-1.483152
H	0.214483	-0.383111	-0.635002
C	0.280640	0.610778	-1.101362
H	-0.735668	1.008764	-1.252570
H	0.758717	0.503810	-2.090016
N	1.114992	2.835051	-0.737357

CH_3HNHN^-

E = -1006.54
H = -962.59
G = -981.97
***N*$_{imag}$** = 0

N	1.111131	1.507811	-0.318348
H	0.747839	1.508507	0.632925
H	0.263020	-0.411248	-0.544435
C	0.267618	0.561193	-1.059901
H	-0.777942	0.905096	-1.170604
H	0.675001	0.410810	-2.066651
N	0.980941	2.885019	-0.802963
H	1.848313	3.027732	-1.328705

CH_3SHN^-

E = -852.02
H = -817.52
G = -837.51
***N*$_{imag}$** = 0

S	1.194904	1.529823	-0.084071
H	1.990712	3.066572	-1.434691
H	0.091702	-0.527439	-0.658547
C	0.176015	0.443872	-1.161781
H	-0.826543	0.865130	-1.298973
H	0.646988	0.298722	-2.140471
N	1.220875	3.074206	-0.757559

HS^-

E = -253.19
H = -247.34
G = -260.61
***N*$_{imag}$** = 0

S	1.340738	0.775800	0.735699
H	1.443539	-0.524810	0.401014

HOS^-

E = -384.01
H = -373.71
G = -390.58
***N*$_{imag}$** = 0

H	-4.898684	-2.391366	0.000000
O	-4.396050	-1.568129	0.000000
S	-2.700648	-2.085484	0.000000

H_2NS^-
E = −504.59
H = −486.26
G = −503.41
N N_{imag} = 0

N	-2.367755	-1.154185	-0.351406
S	-0.714412	-0.576766	0.116713
H	-3.027592	-0.527240	0.106195
H	-2.504939	-2.042280	0.128498

HSS^-
E = −335.26
H = −327.18
G = −345.07
N_{imag} = 0

H	-5.122263	-2.552949	0.000000
S	-4.423939	-1.394854	0.000000
S	-2.449180	-2.097178	0.000000

CH_3S^-
E = −612.09
H = −587.08
G = −603.77
N_{imag} = 0

C	-0.222893	-1.877126	-0.000008
H	0.467271	-1.026690	-0.000021
S	-1.974937	-1.311149	0.000075
H	-0.003676	-2.484665	0.884553
H	-0.003731	-2.484649	-0.884598

CH_3OS^-
E = −741.01
H = −712.66
G = −732.04
N_{imag} = 0

C	-0.784887	-1.464945	-0.772734
H	-0.012250	-0.681681	-0.787463
O	-1.105968	-1.865971	0.552207
H	-0.400621	-2.355957	-1.284997
H	-1.668314	-1.093532	-1.313269
S	-1.747303	-0.567880	1.542682

CH_3HNS^-
E = −865.18
H = −828.82
G = −848.28
N_{imag} = 0

N	-2.353957	-1.212701	-0.333121
S	-0.633448	-0.717141	-0.178329
C	-3.294589	-0.159117	0.062896
H	-2.469836	-1.978395	0.325724
H	-4.314229	-0.566023	0.030417
H	-3.114339	0.249204	1.071729
H	-3.236417	0.670688	-0.649240

CH_3SS^-
E = −704.01
H = −676.83
G = −697.39
N_{imag} = 0

C	-0.795809	-1.463472	-0.740422
H	-0.034657	-0.678629	-0.713008
S	-1.181309	-2.056430	0.934076
H	-0.406516	-2.326798	-1.291170
H	-1.694038	-1.090558	-1.240636
S	-1.919490	-0.394081	1.945429

HOH
E = -330.31
H = -314.83
G = -328.28
N_{imag} = 0

H	-0.007325	-0.005691	-5.534996
O	-0.004720	-0.003667	-4.569799
H	-0.745025	-0.578788	-4.339980

HOOH
E = -417.60
H = -398.78
G = -415.02
N_{imag} = 0

H	-4.704135	-2.290157	-0.284403
O	-4.303052	-1.603978	0.273515
O	-2.909624	-2.023017	0.278414
H	-2.531817	-1.428350	-0.389874

H_2NOH
E = -558.57
H = -531.26
G = -548.15
N

N	-2.230500	-1.124110	-0.350651
O	-0.950536	-0.688623	0.184813
H	-2.897595	-0.475300	0.069193
H	-2.400135	-2.010003	0.127618
H	-0.427301	-0.547344	-0.613965

HSOH
E = -389.01
H = -372.66
G = -390.10
N_{imag} = 0

H	1.576319	-1.278582	-0.400302
O	-0.336531	-2.061443	0.644897
S	1.294603	-2.358121	0.366325
H	-0.826178	-2.497594	-0.067047

CH_3OH
E = -684.22
H = -650.12
G = -667.16
N_{imag} = 0

C	-0.290290	-1.836017	0.006849
H	0.413098	-0.994144	-0.017782
O	-1.653136	-1.389718	-0.056365
H	-0.101454	-2.434431	0.907079
H	-0.119959	-2.461607	-0.872577
H	-1.810802	-0.835669	0.716867

CH_3OOH
E = -778.30
H = -741.65
G = -760.87
N_{imag} = 0

C	-0.773207	-1.457535	-0.790125
H	-0.021101	-0.662207	-0.747292
O	-1.108253	-1.928364	0.514704
H	-0.350317	-2.338133	-1.284988
H	-1.661222	-1.118716	-1.336650
O	-1.629351	-0.801864	1.284843
H	-2.587191	-0.925599	1.176562

CH_3HNOH
E = -922.09
H = -876.93
G = -896.15
***N*imag** = 0

N	-2.295809	-1.308382	-0.202673
O	-0.943044	-0.899691	0.156237
C	-3.192472	-0.175639	0.038657
H	-2.502290	-2.013286	0.504350
H	-4.215805	-0.548544	-0.077143
H	-3.086786	0.260516	1.041947
H	-3.017870	0.599619	-0.712210
H	-0.457873	-1.030265	-0.668464

CH_3SOH
E = -760.68
H = -725.47
G = -745.61
***N*imag** = 0

S	1.137695	-2.298308	-0.732375
O	-0.294942	-1.917394	0.065642
C	2.332062	-1.225161	0.079532
H	2.056404	-0.172694	-0.024232
H	2.467871	-1.491679	1.130782
H	3.270930	-1.402393	-0.457703
H	-0.362730	-2.510745	0.827831

H_2NH
E = -446.65
H = -423.13
G = -436.84
***N*imag** = 0

N	1.369716	1.375799	-0.024297
H	0.784126	1.556412	0.788561
H	0.803509	0.794624	-0.639033
H	2.113367	0.766763	0.310826

HOHNH
E = -557.25
H = -530.02
G = -546.74
***N*imag** = 0

N	0.774837	1.632268	-0.032943
H	1.172383	1.165172	0.783669
H	-0.040584	-0.064620	-0.585196
O	0.528504	0.595325	-1.009524
H	-0.143151	1.966955	0.264367

H_2NHNH
E = -689.27
H = -653.72
G = -670.22
N_{imag} = 0

N	1.219549	1.094570	0.121348
H	0.833893	1.949407	0.513487
H	0.322374	1.071624	-1.711937
N	0.232849	0.594365	-0.814880
H	0.476867	-0.375582	-0.996247
H	2.065382	1.377624	-0.373585

HSHNH
E = −513.37
H = −489.05
G = −506.66
N_{imag} = 0

N	0.825283	1.741320	0.141307
H	1.325686	1.273727	0.891129
H	-0.208632	-0.313909	-0.606740
S	0.539455	0.670578	-1.167486
H	-0.047995	2.102129	0.513449

CH_3HNH
E = −804.45
H = −762.36
G = −779.52
N_{imag} = 0

N	0.828669	1.588765	-0.104995
H	1.342269	1.083203	0.612672
H	-0.405132	-0.134183	-0.591022
C	0.250671	0.619081	-1.052691
H	-0.331381	1.154259	-1.809469
H	1.058292	0.091473	-1.569676
H	0.073790	2.053551	0.393561

CH_3OHNH
E = −916.29
H = −871.22
G = −890.37
N_{imag} = 0

O	1.296756	1.258150	-0.328285
H	0.206254	1.110119	1.319244
H	0.551373	-0.557754	-1.063977
C	0.373973	0.523284	-1.141801
H	-0.664605	0.747335	-0.863668
H	0.555445	0.850090	-2.169450

N	1.183553	0.940317	1.064719
H	1.305059	-0.073851	1.138446

CH_3HNHNH
E = −1051.72
H = −998.32
G = −1017.46
N_{imag} = 0

N	1.053535	1.492987	-0.236993
H	0.582011	1.567037	0.659529
H	0.148331	-0.361852	-0.581674
C	0.272904	0.596098	-1.097407
H	-0.725793	0.991869	-1.346708
H	0.816021	0.415746	-2.030664
N	1.152131	2.841285	-0.730823
H	1.842567	2.830006	-1.478172
H	0.272341	3.117097	-1.177565

CH_3SHNH
E = −883.60
H = −840.40
G = −860.69
N_{imag} = 0

S	1.090468	1.573890	-0.028130
H	1.846260	2.949494	-1.690323
H	0.074995	-0.466682	-0.643944
C	0.204505	0.481720	-1.175887
H	-0.779206	0.887178	-1.427496
H	0.787910	0.305242	-2.083578
N	1.298456	3.060571	-0.841050
H	0.405855	3.481592	-1.085403

HSH
E = −253.25
H = −241.60
G = −256.26
N_{imag} = 0

S	1.336319	0.810393	0.832922
H	1.437835	-0.424139	0.308751
H	1.316659	1.388090	-0.381416

HOSH
E = −389.01
H = −372.66
G = −390.10
$\boldsymbol{N_{imag}}$ = 0

H	-4.844312	-2.395615	0.035388
O	-4.306099	-1.591371	0.053679
S	-2.701551	-2.077290	-0.071425
H	-2.421281	-2.214594	1.245722

H_2NSH
E = −513.19
H = −488.77
G = −506.50
$\boldsymbol{N_{imag}}$ = 0

N	-2.322437	-1.012482	-0.296401
S	-0.812757	-0.414182	0.304807
H	-3.071721	-0.572837	0.230771
H	-2.367618	-2.009842	-0.107058
H	-0.343713	0.089388	-0.856177

HSSH
E = −335.48
H = −321.57
G = −339.60
$\boldsymbol{N_{imag}}$ = 0

H	-5.034826	-2.565059	0.033604
S	-4.371944	-1.388518	0.047366
S	-2.440191	-2.103924	-0.068650
H	-2.178162	-2.219716	1.251044

CH_3SH
E = −619.32
H = −588.26
G = −606.41
$\boldsymbol{N_{imag}}$ = 0

C	-0.253534	-1.901081	-0.036878
H	0.331168	-0.985812	-0.140567
S	-2.036693	-1.534928	0.032340
H	-0.006608	-2.376612	0.914356
H	-0.026077	-2.591374	-0.850574
H	-2.150275	-0.967074	-1.181953

CH_3OSH
E = −745.60
H = −711.26
G = −731.40
N_{imag} = 0

C	-0.712799	-1.441462	-0.820691
H	0.253531	-0.928901	-0.784453
O	-1.224979	-1.677459	0.509771
H	-0.592389	-2.432907	-1.265301
H	-1.424517	-0.854732	-1.411751
S	-1.369684	-0.315675	1.468131
H	-2.555000	0.150688	1.002469

CH_3HNSH
E = −874.26
H = −831.92
G = −851.90
N_{imag} = 0

N	-2.317386	-1.212224	-0.265361
S	-0.660405	-0.808682	-0.232923
C	-3.266205	-0.150966	0.090970
H	-2.478663	-2.041620	0.296893
H	-4.280210	-0.550445	-0.015172
H	-3.141083	0.219471	1.118995
H	-3.148681	0.688467	-0.598914
H	-0.461810	-0.440565	1.064190

CH_3SSH
E = −705.08
H = −672.16
G = −693.34
N_{imag} = 0

C	-0.716051	-1.463842	-0.791678
H	0.232303	-0.928496	-0.721974
S	-1.350997	-1.879403	0.859391
H	-0.554162	-2.428398	-1.283608
H	-1.447433	-0.885203	-1.358742
S	-1.516503	-0.073906	1.825136
H	-2.759724	0.283697	1.430328

TS: HO^- + C_2H_5Cl
E = −1162.54
H = −1111.06
G = −1135.27
N_{imag} = −438.614

C	-1.029936	-5.674075	0.056555
C	-0.848771	-4.199988	-0.131284

O	1.458802	-4.236841	-0.212906
H	-1.527562	-5.898720	1.002824
H	-0.047004	-6.152263	0.069645
H	-1.607747	-6.113064	-0.760118
H	-0.604913	-3.573834	0.709513
H	-0.675494	-3.798393	-1.114824
Cl	-2.965085	-3.473628	-0.137265
H	1.592276	-3.287573	-0.323983

TS: HOO^- + C_2H_5Cl

E = −1260.71

H = −1205.94

G = −1232.41

N_{imag} = −380.861

C	-0.815816	-5.834021	0.261542
C	-0.772398	-4.371030	-0.051390
Cl	1.360711	-3.889824	0.146164
H	-0.464193	-6.039273	1.275527
H	-0.217822	-6.415125	-0.444560
H	-1.851215	-6.186649	0.190899
H	-1.070092	-3.656921	0.698814
H	-0.822274	-4.041784	-1.076116
O	-3.068837	-4.287443	-0.393454
O	-3.507075	-2.933595	-0.753839
H	-3.601388	-2.512135	0.112095

TS: H_2NO^- + C_2H_5Cl

E = −1393.85

H = −1331.07

G = −1357.81

N_{imag} = −361.919

Cl	1.632683	1.697596	-0.000024
C	-0.001945	0.273160	0.000434
N	-0.424985	-2.881600	-0.000550
H	-0.682906	-3.460632	0.805007
H	-0.683852	-3.459715	-0.806462
H	0.296889	-0.215002	0.913668
O	-1.279541	-1.742617	0.000614
C	-1.224750	1.135883	-0.000031
H	-1.272511	1.771496	0.887855
H	-1.273137	1.769667	-0.889191
H	-2.109600	0.489498	0.000951
H	0.297103	-0.215832	-0.912272

TS: HSO^- + C_2H_5Cl
E = −1236.46
H = −1184.01
G = −1211.26
***N*imag** = −386.815

C	0.967232	1.656414	-0.043728
H	0.056813	2.267450	-0.030556
H	1.519989	1.904431	-0.952620
H	1.564963	1.935623	0.826884
O	-1.667331	0.599411	0.013702
H	-2.730521	-1.082370	1.265344
C	0.575460	0.218153	-0.008417
H	0.325337	-0.255803	0.926267
Cl	2.619572	-0.821716	-0.017797
H	0.297557	-0.295739	-0.913394
S	-2.601801	-0.704550	-0.043989

TS: CH_3O^- + C_2H_5Cl
E = −1517.19
H = −1448.49
G = −1474.09
***N*imag** = −408.954

C	-1.068954	-5.658629	0.603139
C	-0.688988	-4.477156	-0.233407
O	1.608026	-4.812945	-0.030357
H	-1.640823	-5.359660	1.484862
H	-0.154724	-6.153045	0.944566
H	-1.653316	-6.384527	0.032654
H	-0.373167	-3.564048	0.242959
H	-0.390800	-4.620300	-1.258518
Cl	-2.647501	-3.640064	-0.799371
C	2.266437	-3.807013	-0.714297
H	2.022660	-3.765141	-1.801763
H	3.372572	-3.913298	-0.668347
H	2.055394	-2.782964	-0.326152

TS: CH_3OO^- + C_2H_5Cl
E = −1602.12
H = −1547.48
G = −1576.07
***N*imag** = −368.978

C	-0.982684	-1.685772	-2.135104
C	-1.046512	-0.410315	-1.366769
O	0.446943	-0.983672	0.380831
H	-1.921518	-2.242048	-2.078899
H	-0.188656	-2.309587	-1.706919
H	-0.734210	-1.511653	-3.185119

H	-1.614759	-0.356947	-0.452403
H	-0.280053	0.334409	-1.504338
Cl	-2.566193	0.792656	-2.488446
O	1.419296	-1.995602	0.034007
C	2.579902	-1.365966	-0.480271
H	2.363386	-0.802466	-1.398612
H	3.286122	-2.173895	-0.709631
H	3.030180	-0.687986	0.259662

TS: CH_3HNO^- + C_2H_5Cl
E = −1757.25
H = −1676.57
G = −1705.78
N_{imag} = −358.542

Cl	1.558930	1.878497	0.249190
C	0.002145	0.376749	0.016118
N	-0.302856	-2.803034	0.005044
H	-0.117430	-4.476699	1.255396
H	-0.413037	-3.428473	-0.797486
H	0.233067	-0.141286	0.932392
O	-1.156353	-1.704601	-0.214657
C	-1.257106	1.179318	-0.070846
H	-1.427356	1.765285	0.836080
H	-1.250449	1.854689	-0.930159
H	-2.102285	0.491599	-0.188991
H	0.417001	-0.050736	-0.881961
C	-0.722706	-3.563189	1.186174
H	-0.549006	-2.964952	2.087285
H	-1.790433	-3.846380	1.163424

TS: CH_3SO^- + C_2H_5Cl
E = −1606.85
H = −1535.52
G = −1565.71
N_{imag} = −367.926

C	0.967006	1.662979	-0.001818
H	0.027074	2.227275	0.036354
H	1.519172	1.999465	-0.882346
H	1.538159	1.910884	0.895925
O	-1.649497	0.496240	-0.064714
H	-1.930791	-1.500290	2.166971
C	0.646132	0.209695	-0.070725
H	0.410299	-0.337680	0.826256
Cl	2.738179	-0.740482	-0.144854
H	0.384068	-0.247570	-1.010075
S	-2.560538	-0.817464	-0.091346
C	-2.861706	-1.250270	1.647327

H	-3.372063	-0.436591	2.174203
H	-3.511311	-2.133010	1.636076

TS: H_2N^- + C_2H_5Cl

E = −1261.12
H = −1202.69
G = −1227.75
N_{imag} = −411.660

C	-0.861928	-5.794715	0.131303
C	-0.671971	-4.305415	0.101599
Cl	1.480735	-4.070601	0.271449
H	-0.441610	-6.229514	1.041981
H	-0.393417	-6.273073	-0.732773
H	-1.928595	-6.031590	0.111331
H	-0.905806	-3.727895	0.981192
H	-0.744681	-3.781289	-0.837108
N	-3.180982	-3.658654	-0.192928
H	-3.167381	-4.196800	-1.063361
H	-2.963263	-2.712674	-0.521518

TS: $HOHN^-$ + C_2H_5Cl

E = −1379.25
H = −1317.32
G = −1344.69
N_{imag} = −366.212

C	-0.765985	-5.821802	0.005813
C	-0.847743	-4.344031	0.263494
Cl	1.175068	-3.746183	0.531591
H	-0.300311	-6.346016	0.844574
H	-0.195317	-6.037742	-0.901167
H	-1.772537	-6.231097	-0.123669
H	-1.208227	-4.005702	1.222374
H	-1.053727	-3.686834	-0.567791
N	-3.441935	-4.276888	0.053176
H	-3.333012	-4.381387	-0.964867
O	-3.907104	-2.853751	0.145833
H	-3.093742	-2.330621	0.161607

TS: H_2NHN^- + C_2H_5Cl

E = −1505.43
H = −1435.29
G = −1462.84
N_{imag} = −350.953

C	-0.770113	-5.843468	0.066516
C	-0.643801	-4.349450	0.178945
Cl	1.400807	-3.983327	0.391895
H	-0.400649	-6.345099	0.965317

H	-0.220382	-6.227680	-0.797222
H	-1.823563	-6.111173	-0.058108
H	-0.990194	-3.879923	1.087471
H	-0.811566	-3.765309	-0.714313
N	-3.295176	-3.818134	-0.058961
H	-3.212315	-4.115769	-1.035018
N	-3.436040	-2.357733	-0.177080
H	-3.867514	-2.048434	0.692234
H	-2.505087	-1.924267	-0.136344

TS: $HSHN^-$ + C_2H_5Cl
E = −1339.03
H = −1279.33
G = −1307.08
N_{imag} = −424.841

C	-0.791354	-5.850146	0.070316
C	-0.528199	-4.375866	0.010365
Cl	1.676949	-4.284579	0.009597
H	-0.360102	-6.294601	0.970651
H	-0.386600	-6.364110	-0.804916
H	-1.869918	-6.032185	0.097493
H	-0.621182	-3.776932	0.900830
H	-0.610462	-3.856511	-0.929974
N	-2.917488	-3.706300	0.033932
H	-3.202299	-4.190151	-0.822187
S	-3.042195	-2.049376	-0.350006
H	-4.172759	-1.637681	0.298240

TS: CH_3HN^- + C_2H_5Cl
E = −1617.53
H = −1541.04
G = −1568.69
N_{imag} = −365.453

C	-0.704285	-5.939588	0.327532
C	-0.634267	-4.462570	0.064286
Cl	1.388133	-3.950356	0.436514
H	-0.441834	-6.174535	1.362627
H	-0.037843	-6.497170	-0.336303
H	-1.725459	-6.289168	0.150187
H	-1.073712	-3.792195	0.786837
H	-0.680862	-4.119221	-0.957428
N	-3.217140	-4.141550	-0.520820
H	-3.356159	-4.305746	0.478317
H	-3.232437	-2.469009	-1.796114
C	-3.370741	-2.711349	-0.730203
H	-4.366273	-2.294029	-0.460360
H	-2.639016	-2.071926	-0.184889

TS: CH_3OHN^- + C_2H_5Cl

E = −1737.76

H = −1657.84

G = −1688.22

***N*$_{imag}$** = −338.756

C	-0.703238	-5.831035	0.029670
C	-0.658416	-4.346802	0.249636
Cl	1.350520	-3.910926	0.734928
H	-0.405420	-6.377175	0.929173
H	-0.052038	-6.134659	-0.794510
H	-1.725538	-6.136162	-0.219813
H	-1.119756	-3.951591	1.142456
H	-0.738614	-3.702360	-0.613972
N	-3.313986	-4.125770	-0.344903
H	-2.961938	-4.164317	-1.308433
O	-3.827378	-2.733937	-0.359701
H	-3.606438	-2.523503	1.703101
H	-5.199584	-3.120912	1.162097
C	-4.365388	-2.444675	0.907361
H	-4.739102	-1.415157	0.871377

TS: CH_3HNHN^- + C_2H_5Cl

E = −1867.31

H = −1779.39

G = −1809.54

***N*$_{imag}$** = −338.826

C	-0.649103	-5.848301	0.080647
C	-0.676753	-4.348705	0.181855
Cl	1.290032	-3.781617	0.611112
H	-0.345427	-6.307748	1.025639
H	0.033370	-6.184348	-0.705058
H	-1.650938	-6.213733	-0.163994
H	-1.166611	-3.910056	1.038479
H	-0.803615	-3.789188	-0.733920
N	-3.352214	-4.064601	-0.373472
H	-3.108345	-4.199344	-1.359648
N	-3.690765	-2.650462	-0.307965
H	-3.962485	-2.784491	1.822671
H	-2.823938	-2.108674	-0.224169
C	-4.443001	-2.378130	0.912751
H	-4.554771	-1.294210	1.038218
H	-5.442613	-2.824323	0.841888

TS: CH_3SHN^- + C_2H_5Cl
E = −1708.06
H = −1629.47
G = −1660.08
N_{imag} = −401.896

C	-0.803164	-5.891042	0.459366
C	-0.513759	-4.478371	0.050417
Cl	1.683131	-4.403402	0.084778
H	-0.407900	-6.108234	1.454784
H	-0.380483	-6.606053	-0.250930
H	-1.885186	-6.051273	0.485916
H	-0.624181	-3.683767	0.769912
H	-0.583242	-4.195506	-0.986561
N	-2.901161	-3.829149	-0.275168
H	-3.245012	-3.972055	0.678988
H	-4.558100	-0.986067	-2.041793
S	-3.085904	-2.177958	-0.581979
H	-5.430832	-2.343184	-1.273962
H	-4.311608	-2.650280	-2.635678
C	-4.489903	-2.039176	-1.744612

TS: HS^- + C_2H_5Cl
E = −1105.57
H = −1056.02
G = −1081.63
N_{imag} = −417.719

C	-1.122868	-5.747228	0.083649
C	-0.795536	-4.301964	-0.135892
S	1.876681	-4.417080	-0.215986
H	-1.672719	-5.886439	1.017127
H	-0.210219	-6.345039	0.150189
H	-1.715220	-6.145210	-0.743230
H	-0.558456	-3.658990	0.694493
H	-0.621169	-3.920516	-1.127356
Cl	-2.938656	-3.381075	-0.196287
H	2.037806	-3.096139	-0.423569

TS: HOS^- + C_2H_5Cl
E = −1240.42
H = −1186.23
G = −1214.51
N_{imag} = −400.666

C	-0.786694	-5.864163	0.252682
C	-0.807325	-4.392083	-0.033414
Cl	1.359567	-3.833696	0.243429
H	-0.438351	-6.062890	1.269000

H	-0.142700	-6.393742	-0.453414
H	-1.789391	-6.295286	0.163652
H	-1.127138	-3.697617	0.726081
H	-0.812933	-4.037747	-1.051234
S	-3.498612	-4.211163	-0.546055
O	-3.794489	-2.563057	-1.014440
H	-3.967381	-2.089095	-0.191313

TS: H_2NS^- + C_2H_5Cl

E = −1361.36

H = −1299.23

G = −1327.62

N_{imag} = −399.186

C	-0.766722	-5.877657	0.262275
C	-0.820697	-4.410660	-0.042933
Cl	1.339223	-3.821235	0.076825
H	-0.350387	-6.059678	1.256078
H	-0.166133	-6.413740	-0.476652
H	-1.771201	-6.312507	0.244525
H	-1.112531	-3.711321	0.723520
H	-0.913585	-4.074918	-1.062848
S	-3.565816	-4.208951	-0.398147
N	-3.790958	-2.499315	-0.815076
H	-4.450262	-2.097083	-0.152535
H	-4.255770	-2.453780	-1.719115

TS: HSS^- + C_2H_5Cl

E = −1190.78

H = −1138.87

G = −1168.04

N_{imag} = −400.398

C	-0.646062	-5.903478	0.193797
C	-0.931259	-4.441021	0.344782
Cl	0.957560	-3.717918	1.422777
H	0.263139	-6.070320	-0.388522
H	-1.464144	-6.405856	-0.333315
H	-0.540773	-6.388753	1.166888
H	-1.552018	-4.084533	1.150019
H	-0.750749	-3.762807	-0.472922
S	-3.370793	-4.406208	-0.843151
S	-4.114209	-2.486855	-0.652067
H	-3.624568	-1.894521	-1.765889

TS: CH_3S^- + C_2H_5Cl

E = −1467.24

H = −1398.38

G = −1427.29

N

C	-1.282245	-5.859153	0.098452
C	-0.890120	-4.416377	-0.012389
S	1.778643	-4.689890	-0.534839
H	-1.682396	-6.083013	1.090113
H	-0.414365	-6.505023	-0.059552
H	-2.029451	-6.122723	-0.653772
H	-0.438213	-3.909630	0.823862
H	-0.796966	-3.950557	-0.979201
Cl	-2.877120	-3.376792	0.359618
C	2.214902	-2.917354	-0.651529
H	1.993201	-2.392295	0.283622
H	1.663136	-2.425272	-1.459451
H	3.284361	-2.801766	-0.856104

TS: CH_3OS^- + C_2H_5Cl

E = −1597.13

H = −1524.92

G = −1555.81

$\boldsymbol{N_{imag}}$ = −399.546

C	-0.753962	-5.915940	0.223509
C	-0.733822	-4.435922	-0.013046
Cl	1.461061	-3.948281	0.260455
H	-0.395564	-6.158943	1.226658
H	-0.139186	-6.441228	-0.511189
H	-1.770914	-6.314620	0.139289
H	-1.022185	-3.758340	0.773569
H	-0.734414	-4.046144	-1.017696
S	-3.427585	-4.212146	-0.517601
O	-3.790339	-2.580510	-0.933458
C	-4.176111	-1.759831	0.170651
H	-3.376561	-1.688671	0.919774
H	-5.085396	-2.139995	0.655680
H	-4.375278	-0.763556	-0.240441

TS: CH_3HNS^- + C_2H_5Cl

E = −1721.81

H = −1641.67

G = −1672.50

$\boldsymbol{N_{imag}}$ = −398.617

C	-0.760578	-5.873480	0.256400
C	-0.845211	-4.408994	-0.051230
Cl	1.290511	-3.748236	0.177367

H	-0.394262	-6.044044	1.271767
H	-0.103437	-6.388830	-0.448361
H	-1.748267	-6.340374	0.183465
H	-1.196535	-3.719865	0.699242
H	-0.897526	-4.075002	-1.074649
S	-3.581547	-4.283706	-0.533681
N	-3.878202	-2.574521	-0.823609
C	-4.721031	-1.914951	0.179309
H	-4.335044	-2.499707	-1.727943
H	-4.202703	-1.906881	1.143171
H	-5.703107	-2.393903	0.325615
H	-4.885883	-0.874790	-0.129358

TS: CH_3SS^- + C_2H_5Cl

E = −1559.79

H = −1489.42

G = −1519.36

$\boldsymbol{N}_{\mathbf{imag}}$ = −391.033

C	-0.720602	-5.946926	0.279248
C	-0.768570	-4.478270	-0.006757
Cl	1.432724	-3.887244	0.230642
H	-0.339263	-6.141437	1.284527
H	-0.095610	-6.471690	-0.447153
H	-1.722718	-6.385682	0.223187
H	-1.065437	-3.783851	0.761849
H	-0.781995	-4.119108	-1.022535
S	-3.454840	-4.288037	-0.500672
S	-3.900576	-2.362910	-1.054535
C	-4.283543	-1.477640	0.488887
H	-3.419329	-1.464819	1.157367
H	-5.141312	-1.929164	0.993763
H	-4.531794	-0.451605	0.197302
